# Overall survival of triple negative breast cancer in French Caribbean women

**DOI:** 10.1371/journal.pone.0271966

**Published:** 2022-08-24

**Authors:** Murielle Murielle Beaubrun-Renard, Jacqueline Veronique-Baudin, Jonathan Macni, Stephen Ulric-Gervaise, Thierry Almont, Aude Aline-Fardin, Nathalie Grossat, Cristina Furtos, Patrick Escarmant, Vincent Vinh-Hung, Stefanos Bougas, André Cabie, Clarisse Joachim

**Affiliations:** 1 UF 1441 Registre Général des cancers de la Martinique, Pôle de Cancérologie Hématologie Urologie, CHU de Martinique, Fort-de-France, Martinique, France; 2 PCCEI, Université de Montpellier, INSERM, EFS, Université Antilles, Montpellier, France; 3 UF 3596 Recherche en cancérologie hématologie, Pôle de Cancérologie Hématologie Urologie, CHU de Martinique, Fort-de-France, Martinique, France; 4 Laboratoire d’anatomopathologie, Pôle de Biologie, CHU de Martinique, Fort-de-France, Martinique, France; 5 UF 1450 - Oncologie Médicale Hospitalisation de Semaine, Pôle de Cancérologie Hématologie Urologie, CHU de Martinique, Fort-de-France, Martinique, France; 6 Pôle de Cancérologie Hématologie Urologie, CHU de Martinique, Fort-de-France, Martinique, France; 7 UF 1401 Radiothérapie, Pôle de Cancérologie Hématologie Urologie, CHU de Martinique, Fort-de-France, Martinique, France; 8 Service des maladies infectieuses et tropicales, Martinique, CHU de Martinique, Fort-de-France, Martinique, France; 9 CIC-1424, INSERM, CHU de Martinique, Fort-de-France, Martinique, France; CNR, ITALY

## Abstract

To describe survival according to prognostic factors of women with breast cancer in French overseas territory (Martinique) during 2008–2017. We performed a Cox model for prognostic factors for OS in breast cancer patients. The cut-off date for the analysis was 13/10/2018. The main factors were demographic data, stage, hormone receptors (HR) status and HER2 status. Curves were compared with the log rank test to select candidate variables for the multivariate analysis. We included 1,708 patients; median age at diagnosis was 57 years. Triple negative breast cancer (TNBC) accounted for 20.9% (n = 332). Among the patients, 72.3% (n = 1015) had localised or local spread cancer. One-year OS was 95.2% and was 80.1% at 5 years. In TNBC, 1-year-survival was 90.4%, which fell to 70.1% at 5 years. Patients with metastatic disease at diagnosis had 1-year-survival of 74.5%, and 20.1% at 5 years. Multivariate analysis by Cox regression identified 4 factors significantly associated with an increased risk of death: metastatic disease at diagnosis (hazard ratio (HR) = 15, p<0.0001), TNBC (HR 2.84, p<0.0001), HR+/HER2- status (HR 2.05, p<0.0084) and age >75 years (HR 3.8, p<0.0001). This is the first study performed on breast cancer survival in Martinique. Our findings show that breast cancer has overall good prognosis in patients and also how prognosis factors are distributed in the population.

## Introduction

Breast cancer is the leading cancer in women worldwide. In 2018, there were an estimated 2.08 million new cases of breast cancer, and 0.68 million women died from invasive breast cancer. In France, the estimated number of new breast cancer cases in 2018 was 58,459, with 12,146 deaths [1]. Two types of factors play a role in determining survival from breast cancer, namely risk factors (factors that promote the disease), and prognostic factors (i.e. parameters that influence how the cancer will affect the patient and the response to treatment).

Risk factors identified in previous research include mainly hormonal factors and reproduction (earlymenarche, latemenopause, older age at first pregnancy). In addition, other risk factors have been shown to play a role including environmental and sanitary factors, and age, while a small percentage of breast cancers are hereditary.

According to the data from the French Public Health Authority, over the last few years, survival among persons with breast cancer has been increasing. Net survival at 5 years in mainland France was estimated at 88% between 2010 and 2015 [2].

However, survival may vary depending on the presence or absence of various prognostic factors. The stage of the cancer at diagnosis is a key prognostic factors, to which may be added other features such as hormone receptor status, HER2 status, grade, or age at diagnosis [3].

Breast cancer represents a major public health challenge that requires special attention, because it is ranked first among incident cancer cases in women in terms of frequency and mortality [4]. The healthcare pathway of patients with breast cancer has improved consistently in recent years, with the discovery of new biomarkers that are prognostic factors for the disease. These biomarkers, including hormone receptors to estrogen and/or progesterone, or transmembrane HER2 receptors, have also made it possible to develop new therapies, such as targeted therapy. However, there is a form of breast cancer that expresses neither hormone receptors nor HER2 receptors, thereby limiting the treatment options to standard therapy in these patients, i.e. surgery, radiotherapy and chemotherapy.

Martinique, a French overseas territory, is an island of 1,128 square kilometres with approximately 368,783 inhabitants. As a French overseas Department, one might expect the healthcare pathway of patients with cancer to be of the same quality in Martinique as in mainland France, and thus, the survival curves to be roughly equivalent. However, a study performed by the cancer registry of Guadeloupe, a neighbouring island that is also a French overseas territory, showed that for the period 2008–2013, incidence of breast cancer was high, albeit lower than in mainland France, with a net survival of 84% [5]. Furthermore, data from the international CONCORD-2 study published in 2015 and performed in various regions of the world from 1990 to 2015, showed that the largest increase in mortality from breast cancer was observed in the Caribbean [5–8].The indicators provided by cancer surveillance data underscore the need for reliable data about breast cancer survival in Martinique, taking account of stage at diagnosis, but also specific data regarding hormone receptor status, in view of the disparities in survival observed across the Caribbean and around the world [5–8].

In this context, this study aims to describe overall survival of women with breast cancer, from 2008 to 2017 in Martinique.

## Materials and methods

### Population

Cancer registries provide reliable epidemiological data on cancer, including data on incidence, mortality, stage at diagnosis and survival, thanks to systematic recording of all cancer cases. In the present retrospective study, we extracted data from the Martinique Cancer Registry for the period from 2008 to 2017. The Martinique Cancer Registry has been classed at category A at national level for the quality of its data and its utility for public health and research. Data have been recorded systematically since 1981 in accordance with national and international guidelines for general cancer registries. Patients included in the present analysis were all women who were diagnosed between 01/01/2008 and 31/12/2017 with invasive breast cancer of histological type 8500/3 (infiltrating duct carcinoma), 8022/3 (Pleomorphic carcinoma), 8035/3 (carcinoma with osteoclast-like giant cells) or 8140/3 (adenocarcinoma) according to the International Classification of Diseases-Oncology 3^rd^ Edition [9].

### Selected variables

We selected socio-demographic data, namely age at diagnosis, as well as incidence date and place of residence. Age was grouped into categories for analysis (<50 years, 50–74 years, and ≥75 years) to take into account the age group targeted by the national breast cancer screening programme (50 to 74 years old). Area of residence was classed in four zones based on the geographical addresses and using data from the national statistics institute (INSEE), into North Caribbean, North Atlantic, Centre, South.

Histological type was selected as well as Scarff-Bloom Richardson grade (I, II, III). Hormone receptor (HR) status was selected as either positive or negative (for estrogen receptors (ER) and progesterone receptors (PR)), and HER2 overexpression was also selected as either positive or negative. Patients were grouped into HR positive (i.e. ER and/or PR positive), and HR negative (both ER and PR negative). This yielded four groups overall, namely: HR+/HER2+, HR+/HER2-, HR-/HER2+ and triple negative breast cancer (TNBC, HR-, HER2-). We did not use the Luminal A and B classification since data regarding the Ki-67 antigen were missing.

Data regarding TNM stage at diagnosis were classified according to the recommendations of the European Network Cancer Registries (ENCR) into three condensed classes, namely: localised/local spread (T1-T4,N0M0), regional spread (Nodule +)and metastatic (Metastasis +) [10].

### Statistical analysis

Descriptive analysis was performed in the overall population, and by age category. Qualitative variables are described as number (percentage) and quantitative variables as mean ± standard deviation (SD). Univariate analysis of survival was performed using the Kaplan-Meier method to estimate average survival time for diagnosis. Curves were compared with the log rank test to select candidate variables for entry into the multivariate analysis. Variables with a p-value <0.20 were included in the multivariate model. The factors independently associated with the risk of death were identified using Cox’s proportional hazards model. The cut-off date for the analysis was 13/10/2018. The main prognostic and therapeutic factors included in the model were age, stage, and the subtypes of cancer defined above based on HR status and HER2 status. Analyses were performed using SAS version 9.4 (SAS Institute Inc., Cary, NC) and a p-value <0.05 was considered statistically significant.

### Ethics statement

This specific study was reviewed and approved by the following institutional review board: Comité de Protection des Personnes Ile de France IV Institutional Review Board Agreement of US Department of Health and Human Services, N°IRB 00003835. Our study did not involve direct patient contact.

## Results

Overall, a total of 1,708 patients were diagnosed with invasive breast cancer from 2008 to 2017 (histological type adenocarcinoma) and were included in the analysis. Average age was 58.0±14.0 years, median age at diagnosis was 57 years, and 121 (7.1%) patients were aged <40. Patients were resident mainly in the Centre and South of Martinique (43.4% and 32.1% respectively). The clinical characteristics of the study population are shown in Table 1.

**Table 1:** Characteristics of breast cancer patients according to age group in Martinique, 2008–2017.

	All		<50 years		50–74 years		≥75 years	
	n	%	n	%	n	%	n	%
	1708	100.00%	495	28.98	979	57.32	234	13.70
Zone of residence								
Center	742	43.44	217	43.84	406	41.47	119	50.85
North-Atlantic	327	19.15	98	19.80	189	19.31	40	17.09
North-Caribbean	91	5.33	20	4.04	60	6.13	11	4.70
South	548	32.08	160	32.32	324	33.09	64	27.35
Breast cancer subtypes								
HR+/Her2+	246	15.46	84	17.65	140	15.05	22	11.89
HR+/Her2-	883	55.50	250	52.52	523	56.24	110	59.46
HR-/Her2+	130	8.17	45	9.45	69	7.42	16	8.65
TNBC	332	20.87	97	20.38	198	21.29	37	20.00
Unknown	117	-	19	-	49	-	49	-
SBR grading								
Grade 1	155	10.07	39	8.57	100	11.48	16	7.51
Grade 2	800	51.98	231	50.77	444	50.98	125	58.69
Grade 3	584	37.95	185	40.66	327	37.54	72	33.80
Unknown	169	-	40	-	108	-	21	-
ENCR Condensed Staging								
Localized/ local spread	1015	72.34	297	71.39	577	72.95	141	71.94
Regional	274	19.53	86	20.67	150	18.96	38	19.39
Metastatic	114	8.13	33	7.93	64	8.09	17	8.67
Unknown	305	-	79	-	188	-	38	-

**Fig 1:**
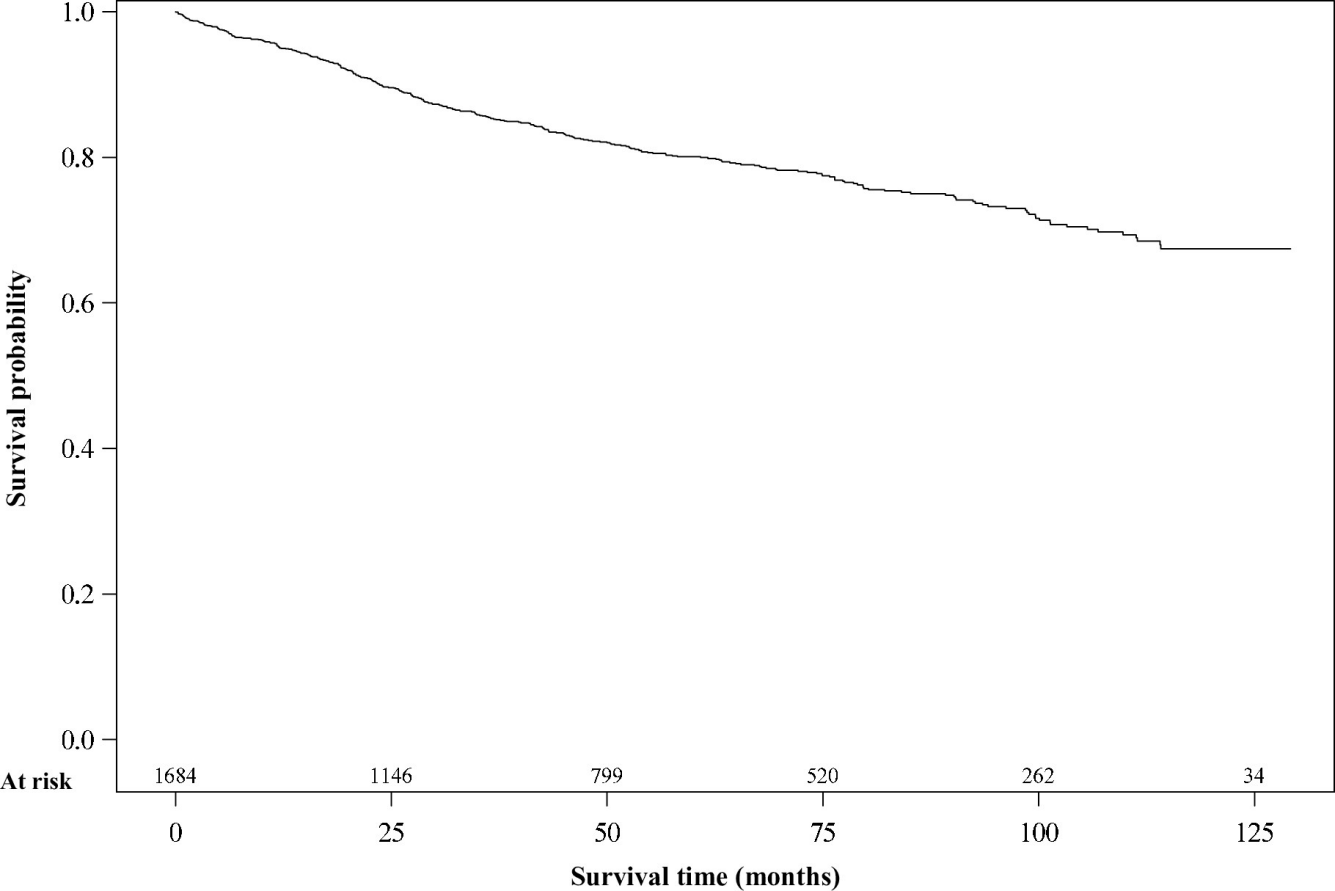
Overall survival in breast cancer patients, Martinique, 2008–2017.

The overall survival curve has been provided. The survival median hadn’t been reached at 10 years. Fig 1 shows the Kaplan Meyer curve.

There was a significant difference in survival between age categories, with shorter survival among patients aged >75 years (5-year-survival, 56% (95% confidence intervalle [CI], 48–63); 10-year-survival, 28% (95%CI, 17–40)). For the other class of age <50 years and [50–74] years, the 5-year-survival rate was 85% (95%CI, 81–88) and 83% (95%CI, 80–86) respectively and the 10-year-survival rate was 76% (95%CI, 70–81) and 72% (95%CI, 67–77) respectively.

**Fig 2:**
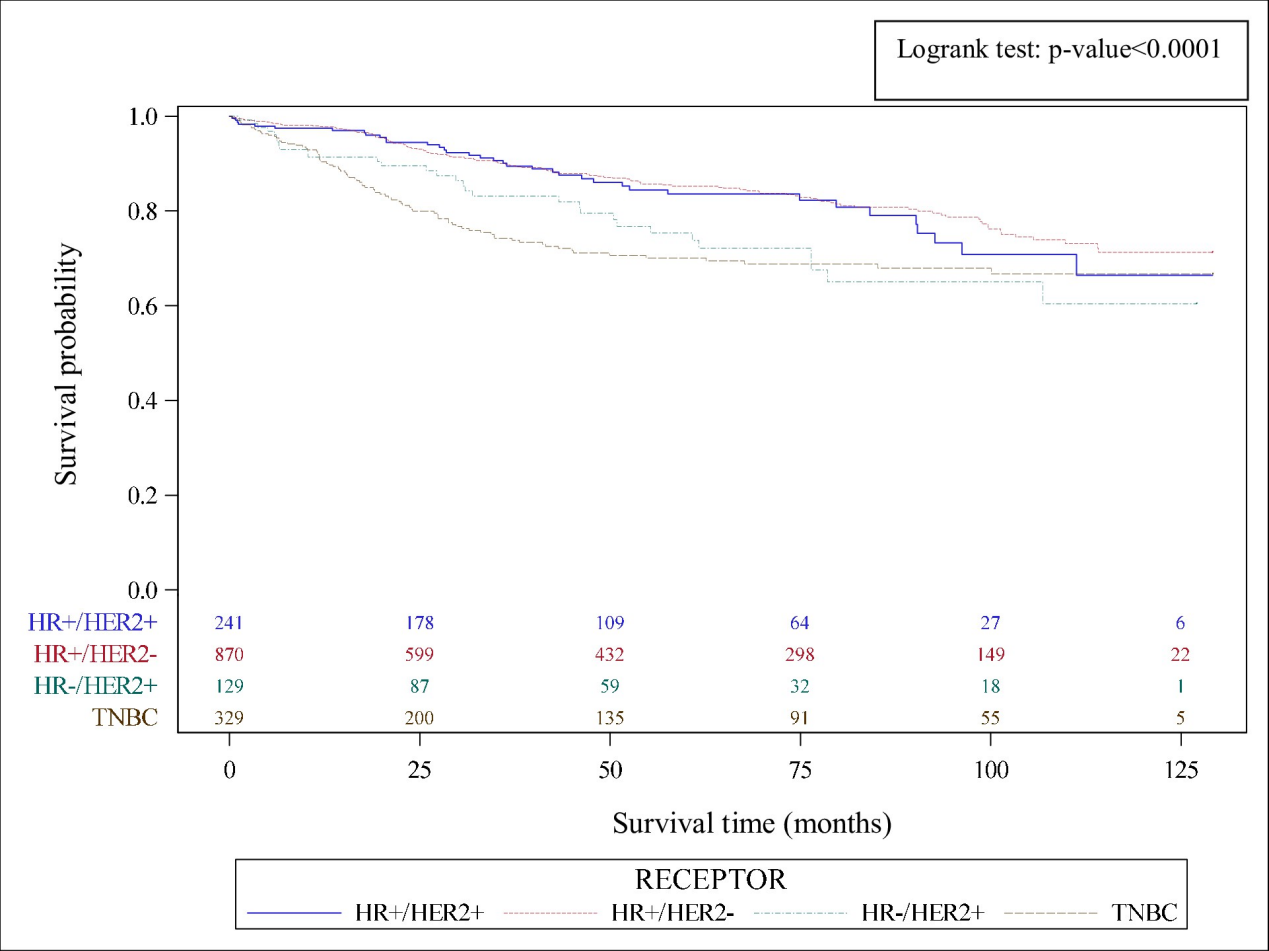
Overall survival in breast cancer patients, stratified by receptor status, Martinique, 2008–2017.

HR and HER2 status was available for 1,591 patients (93.1%). TNBC accounted for 20.9% of cases (n = 332), and there was no difference between age categories. Fig 2 shows the comparison of survival according to HR/HER2 status, with shorter survival in the TNBC group.

In total, 52.0% (n = 800) of patients had grade 2 cancer at diagnosis; 38.0% (n = 584) had grade 3. A total of 155 patients had grade 1 cancer at diagnosis (10.0%).

Regarding stage at diagnosis, 72.3% (n = 1015) had localised or local spread cancer; 19.5% (n = 274) had regional stage cancer and 8.1% (n = 114) had metastatic stage cancer. There was significantly shorter survival among patients with metastatic cancer (5-year-survival, 20% (95%CI, 12–30)); 10-year survival, 0,03%(95%CI, 0–0,11)). For the other stage, localised or local spread cancer and regional stage cancer, the 5-year-survival rate was 88% (95%CI, 85–90) and 74 (95%CI, 67–80) respectively and the 10-year-survival rate was 77% (95%CI, 72–81) and 52% (95%CI, 40–62).

Overall survival was 95.2% at 1 year, and 80.1% at 5 years. Overall survival at 1, 3 and 5 years is detailed in Table 2. Analysis of survival according to age at diagnosis shows that patients aged >75 years had shorter overall survival, with 5-year-survival of 55.7% compared to 85% in those aged <50. Analysis according to HR and HER2 status showed that 1-year-survival was 90.4% in patients with TNBC, which fell to 70.1% at 5 years. In contrast, patients with HR+/HER2+ and HR+/HER2- had overall survival of 83.6% and 85.2% respectively at 5 years. Patients with metastatic disease at diagnosis had 1-year-survival of 74.5%, and 20.1% at 5 years. Conversely, patients with localized cancer at diagnosis had longer survival, i.e. 98.0% at 1 year, and 87.5% at 5 years.

**Table 2:** Overall survival in patients with breast cancer in Martinique, 2008–2017.

	1 year	3 years	5 years
	% [95% CI]	% [95% CI]	% [95% CI]
All periods	95.16 [94.00–96.10]	85.59 [83.64–87.32]	80.08 [77.76–82.19]
2008–2012	95.63 [93.93–96.85]	85.46 [82.77–87.76]	80.54 [77.55–83.17]
2013–2017	94.77 [93.06–96.07]	85.89 [82.90–88.40]	77.60 [72.76–81.70]
Zone of residence			
Center	95.64 [93.85–96.91]	84.91 [81.82–87.52]	78.85 [75.16–82.06]
North- Atlantic	96.51 [93.79–98.05]	87.15 [82.51–90.62]	81.88 [76.43–86.17]
North-Caribbean	92.98 [85.03–96.78]	85.47 [75.08–91.76]	79.57 [67.41–87.60]
South	94.04 [91.64–95.77]	85.53 [81.94–88.46]	80.77 [76.61–84.27]
Age at diagnosis			
< 50 years	97.51 [95.66–98.58]	90.69 [87.50–93.09]	85.02 [81.02–88.24]
50–74 years	96.15 [94.70–97.20]	87.21 [84.67–89.35]	83.04 [80.07–85.60]
≥ 75 years	85.55 [80.07–89.61]	66.52 [59.18–72.85]	55.71 [47.61–63.06]
Breast cancer subtypes			
HR+/Her2+	97.44 [94.40–98.84]	90.06 [84.96–93.49]	83.57 [76.97–88.42]
HR+/Her2-	97.88 [96.65–98.66]	90.03 [87.57–92.04]	85.25 [82.21–87.81]
HR-/Her2+	91.36 [84.95–95.12]	83.15 [74.69–88.98]	75.38 [65.35–82.88]
TNBC	90.36 [86.57–93.12]	74.29 [68.73–79.01]	70.08 [64.15–75.22]
SBR grading			
Grade 1	97.94 [93.74–99.33]	94.53 [88.81–97.37]	91.57 [84.75–95.42]
Grade 2	94.80 [92.97–96.16]	87.82 [85.11–90.07]	80.63 [77.22–83.59]
Grade 3	95.34 [93.22–96.80]	81.28 [77.47–84.51]	77.49 [73.29–81.12]
ENCR Condensed Staging			
Localized/ local spread*	98.03 [96.93–98.74]	91.04 [88.83–92.83]	87.55 [84.85–89.79]
Regional	95.82 [92.57–97.66]	81.30 [75.26–86.00]	74.47 [67.46–80.20]
Metastatic	74.51 [65.23–81.66]	35.90 [25.68–46.21]	20.13 [11.58–30.38]

Analysis of survival according to both age and stage at diagnosis showed that survival was shorter in patients with metastatic disease who were aged >75 years, with 1-year-survival of 55.5% versus 81.2% in those aged <50 years with metastatic disease. The detailed results are shown in Table 2.

**Fig 3:**
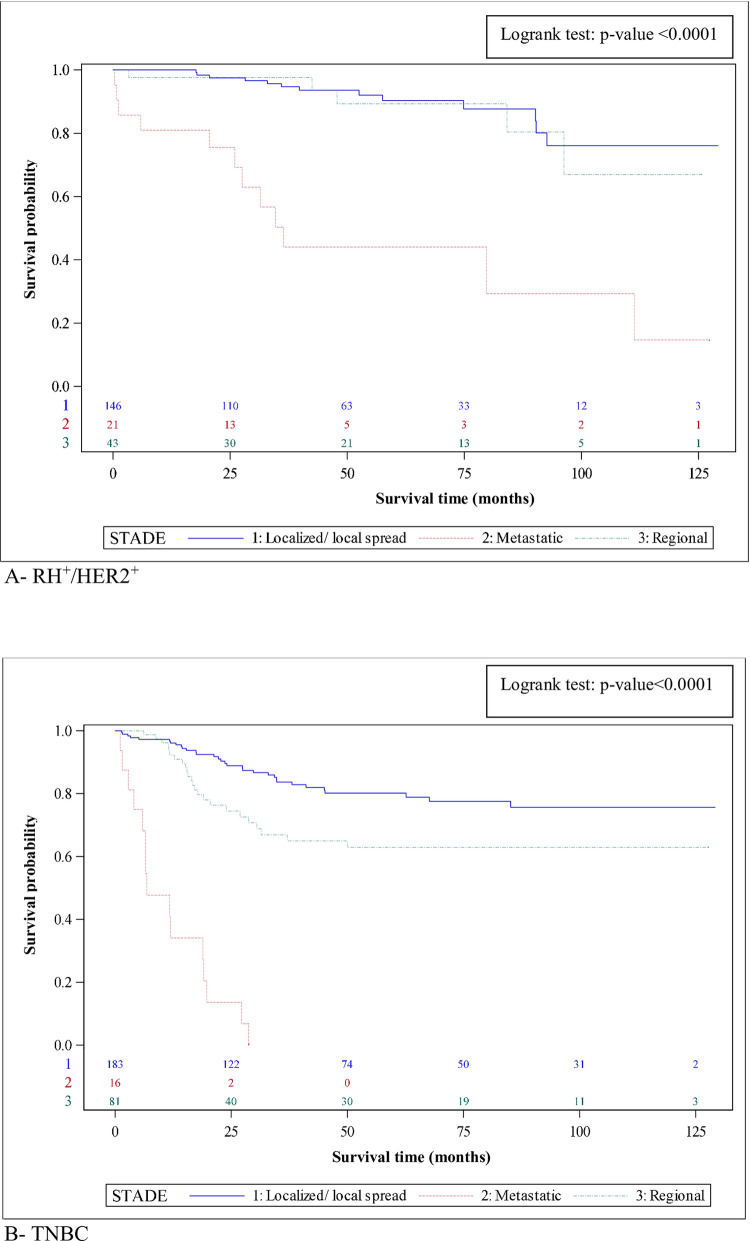
Overall survival in breast cancer patients, subtypes TNBC (A) and HR+/HER2+ (B) stratified by ENCR staging, Martinique, 2008–2017.

Analysis according to HR/HER2 status and stage at diagnosis shows that patients with metastatic triple negative disease had 1-year-survival of 34.1%, compared to 80.9% in those with metastatic HR+/HER2+ disease. Conversely, in case of localised/local spread at diagnosis, survival in patients with TNBC was 80.2% at 5 years. The corresponding survival curves are presented in Fig 3.

Univariate analysis identified 3 factors that were significantly associated with survival, namely age, HR/HER2 subtype, and staging (SBR grade and ENCR stage). Multivariate analysis by Cox regression identified 4 factors significantly associated with an increased risk of death, namely metastatic disease at diagnosis (hazard ratio (HR) = 15, p<0.0001), TNBC (HR 2.84, p<0.0001), HR+/HER2- status (HR 2.05, p<0.0084) and age >75 years (HR 3.8, p<0.0001). The results of the multivariate analysis are shown in Table 3.

**Table 3:** Prognostic factors for breast cancer survival in Martinique, 2008–2017.

	Univariate HR^a^ [95% CI]^*b*^; ^c^*p*	Multivariate HR^a^ [95% CI]^b^; *p*^*c*^
Age at diagnosis		
< 50 years	1	1
50–74 years	1.168 [0.885–1.541]; 0.2729	1.478 [1.073–2.038]; 0.0170
≥ 75 years	4.069 [3.003–5.515]; <0.0001	3.820 [2.633–5.541]; <0.0001
Breast cancer subtypes		
HR+/Her2+	1	
HR+/Her2-	0.921 [0.639–1.328]; 0.6594	1.262 [0.838–1.901]; 0.2654
HR-/Her2+	1.624 [1.008–2.617]; 0.0465	2.054 [1.202–3.510]; 0.0084
TNBC	1.815 [1.235–2.668]; 0.0024	2.848 [1.826–4.43]; <0.0001
SBR grading		
Grade 1	1	
Grade 2	2.081 [1.225–3.537]; 0.0068	
Grade 3	2.447 [1.432–4.182]. 0.0011	
Zone of residence		
Center	1	
North-Atlantic	0.990 [0.771–1.273]; 0.9395	
North-Caribbean	0.958 [0.572–1.605]; 0.8718	
South	0.817 [0.601–1.112]; 0.1989	
ENCR Condensed Staging		
Localized/ local spread*	1	1
Regional	2.365 [1.752–3.192]; <0.0001	2.227 [1.620–3.062]; <0.0001
Metastatic	11.894 [8.848–15.990]; <0.0001	15.006 [10.857–20.740]; <0.0001

^a^HR: hazard ratio

^b^95% CI: 95% confidence interval

^c^ p for Wald test.

## Discussion

This is the first study based on data from the General Cancer Registry of Martinique to report survival in breast cancer patients in Martinique. The objective was to identify prognostic factors for survival in women diagnosed between 2008 and 2017. Our study of 1,708 patients shows that in Martinique, breast cancer has good prognosis with overall survival of 80% at 5 years.

This is different to the net survival reported in mainland France, which was 88% between 2010 and 2015 [1]. We can explain this difference by the fact that the calculated method is not the same. The method used to calculate the net survival in mainland France take into account only the death by cancer. While to calculate overall survival, death all causes is included. Knowing the aging of the population, some women are dead of other cause than their cancer.

In Martinique TNBC represents 21% of breast cancer subtypes. That is more than mainland France (15%) [11]. TNBC is a subtype with a bigger prevalence in women younger than 40 years of age with African or Asian descent [12] or non-Hispanic Black or Hispanic women, as is largely the case in Caribbean populations. Indeed, prevalence ranges between 27% and 82% in Sub-Saharan African, but was reported at 17% and 14% in Puerto Rico and Guadeloupe respectively. The prevalence in Martinique is higher than in Puerto Rico and Guadeloupe, but close to the 25% reported in Barbados [7]. However, in a study performed in Haiti, the rate of TNBC was higher, at 38.5% [8]. In addition, the average age at diagnosis was lower in Haiti, at 49.1 years, which is in line with studies showing a relation between age and triple negative hormone status. We also observed significant discrepancies, with 5-year-survival of 70% in TNBC, compared to 75 to 85% for the other groups. A similar study from the Guadeloupe cancer registry investigating breast cancers diagnosed between 2008 and 2013 was the first to provide data on overall and net survival in breast cancer patients in the French West Indies. The Guadeloupe study reported net survival of 84.9% at 5 years, and 71.9% in TNBC [5]. This underlines the importance of improving management and prognosis of TNBC, which is an aggressive form of breast cancer with poor prognosis. Accordingly, depending on the Caribbean country considered, the rate of TNBC may vary widely. There is thus a compelling need to focus on this form of cancer for two main reasons. Firstly, this type of cancer rapidly progresses to metastatic. Secondly, patients with TNBC was not eligible for targeted therapies [7].Regarding stage at diagnosis, we observed that 72% of patients were diagnosed with localized disease or local spread, and only 8% had metastatic disease at diagnosis. These results show promise of successful therapeutic management with good post-operative prognosis. However, results from Guadeloupe reported only 3.7% of metastatic disease [3]. Finally, overall survival at 5 years according to stage at diagnosis was low in the metastatic group, 20%, like this one described in mainland France [13]. This result highlight the need for early diagnosis of breast cancers.

Analysis of survival according to age, stage and HR status showed that survival is affected by all these factors. Indeed, survival by stage and age at diagnosis showed 1-year-survival above 90% in patients with localized disease at diagnosis, across all age categories. However, 5-year-survival was lower in those age 75 years and older (57%), compared to other age groups, where survival remained above 90% at 5 years. Furthermore, in patients with metastatic disease at diagnosis, lower survival is observed even starting at 1 year, and this difference is most pronounced in those aged >75 years (56%). In patients aged <50 years (n = 495), of whom 33 had metastatic disease at diagnosis, overall survival varied from 81% at 1 year to 25% at 5 years. This gap, can be explain by several factors. The co-morbidity and also the difference of management of the disease. In fact, patient aged >75 years received less aggressive treatment due to co-morbidity, but also less efficient. Thus, whatever the stage at diagnosis, the presence of TNBC is associated with lower survival, with a marked difference at 5 years. Patients with metastatic disease at diagnosis had survival rates ranging from 34 to 0% at 5 years, when 5-year-survival rate is 11% in mainland France [12]. Then this difference of global survival rate can be explained by the small number of patients in the group metastatic TNBC compared to mainland France where the sample is bigger so the analysis is stronger.

We did not observe any difference in overall survival according to the area of residence, which suggests that access to care is equal across the different geographic zones of the island of Martinique. Although the main provider of care for breast cancer patients is centralized in the University Hospital, which provides surgery, radiotherapy and chemotherapy services, the whole population would appear to benefit from the healthcare services provided in an equal manner, based on the survival rates observed in this study.

Our multivariate analysis identified several independent prognostic factors, namely older age (hazard ratio (HR) = 3.8), TNBC (HR = 2.8), and metastatic disease at diagnosis (HR = 15). These factors highlight the need for management of breast cancer in older women in Martinique, taking account of possible comorbidities.

The limits of the study were the small number of patients in the metastatic TNBC group. It was difficult to compare the results with mainland France where the number of patients should be bigger. These latter were able to benefit from a more robust survival analysis.

About the missing data, in our analysis they represented less than 20% of the data in the concerned variables. It was some data that are not well completed in the source data files as breast cancer subtypes (7% of missing data), SBR grading (10% of missing data) and staging (18% of missing data). This point could be a source of bias but it was a non-informative censorship and with the large number of data, the power of the analysis was ever strong.

## Conclusions

This study is the first of its kind to be performed in Martinique, and provides novel data regarding the clinical characteristics of women with breast cancer, and the proportion of different types of breast cancer in Martinique according to age, stage, grade and hormone receptor status. The breast cancer subtype TNBC being more represented in Martinique than in mainland France. Our findings show that breast cancer has overall good prognosis in patients in Martinique when management is initiated rapidly, i.e. before disease has reached metastatic status. However, despite some data regarding TNBC, there remain some open questions. Indeed, TNBC appears to be an aggressive form, as we could see in survival analysis, and it was of prime importance to identify this factor in order to adapt management accordingly, particularly the timing. In fact, this type of breast cancer should be supported quickly to benefit from a maximum survival rate. It would be of interest to investigate the timing of management during the same period as this study, and also during COVID-19 pandemic period, and the impact that this may have had on the management and consequently, survival in breast cancer in Martinique. This would allow to set up a plan to improve delay in access to care to prevent advanced stage breast cancer.

## Supporting information

S1 Data (ZIP)
