# Supplementary material for: Overall survival of triple negative breast cancer in French Caribbean women
Source: PLoS One. 2022 Aug 24;17(8):e0271966. doi: 10.1371/journal.pone.0271966 (PMC9401158; doi:10.1371/journal.pone.0271966)
Supplement: S1 Data — (ZIP) [file pone.0271966.s001.zip › char.pdf]

GRADE	AGE_C	GEO_LOC	STADE	RECEPTOR
3	<50	NordAT		HR-/HER2+
2	<50	Sud		HR+/HER2-
3	<50	Centre		HR+/HER2-
3	<50	Centre		HR+/HER2+
2	<50	NordAT		HR+/HER2-
2	<50	Centre		HR+/HER2-
3	<50	Sud		HR+/HER2-
2	<50	Centre		
3	<50	Sud		HR+/HER2-
3	<50	Sud		HR+/HER2-
3	<50	Sud		TNBC
2	<50	Centre		HR+/HER2-
3	<50	Sud		HR+/HER2+
3	<50	NordAT		HR+/HER2-
3	<50	Centre		HR+/HER2+
3	<50	Sud		HR+/HER2-
3	<50	NordAT		HR+/HER2-
3	<50	Sud		TNBC
3	<50	Centre		HR+/HER2-
3	<50	Sud		TNBC
3	<50	Centre		TNBC
3	<50	Centre		HR+/HER2-
1	<50	Centre		HR+/HER2-
3	<50	NordAT		TNBC
3	<50	Sud		
3	<50	NordAT		
3	<50	Centre		HR+/HER2-
3	<50	Centre		HR+/HER2-
3	<50	NordAT		TNBC
3	<50	NordAT		
3	<50	NordAT		HR+/HER2-
2	<50	NordAT		HR+/HER2-
3	<50	Centre		HR+/HER2-
3	<50	nordca		TNBC
3	<50	Centre		HR+/HER2-
3	<50	Centre		HR-/HER2+
2	<50	Sud		HR+/HER2-
1	<50	Sud		HR+/HER2-
3	<50	Centre		HR+/HER2-
3	<50	Sud		HR+/HER2-
2	<50	Sud		HR+/HER2-
1	<50	Sud		HR+/HER2-
3	<50	Sud		HR+/HER2-
3	<50	Centre		TNBC
2	<50	NordAT		HR+/HER2+
3	<50	Centre		HR-/HER2+
2	<50	Centre		HR+/HER2-
3	<50	Sud		HR+/HER2+
3	<50	Sud		HR+/HER2-

3	<50	nordca		HR+/HER2-
3	<50	NordAT		HR+/HER2-
3	<50	Sud		
3	<50	NordAT		TNBC
3	<50	Centre		HR+/HER2+
9	<50	Centre		
3	<50	Centre		HR+/HER2+
3	<50	Sud		HR-/HER2+
2	<50	NordAT		HR+/HER2-
2	<50	Sud		TNBC
1	<50	NordAT		HR+/HER2-
3	<50	Sud		HR+/HER2-
2	<50	Sud		HR+/HER2+
2	<50	Sud		HR+/HER2-
3	<50	NordAT		HR+/HER2+
2	<50	Sud		HR+/HER2-
2	<50	Centre		HR+/HER2-
3	<50	Centre		TNBC
1	<50	Centre		HR+/HER2-
1	<50	Centre		HR+/HER2-
3	<50	Sud		TNBC
3	<50	Sud		HR+/HER2-
3	<50	NordAT		HR-/HER2+
3	<50	Sud		HR+/HER2+
2	<50	nordca		HR+/HER2-
3	<50	NordAT		HR+/HER2-
3	<50	Centre		TNBC
2	<50	Centre		HR+/HER2-
	<50	Centre		HR+/HER2-
3	<50	Centre		HR+/HER2-
3	<50	Centre		HR+/HER2-
2	[50-74]	Centre		HR+/HER2-
3	<50	Centre		TNBC
3	<50	Sud		HR+/HER2-
3	<50	NordAT		HR+/HER2-
3	<50	Centre		TNBC
3	<50	Sud		TNBC
3	<50	Centre		HR+/HER2-
3	<50	Sud		TNBC
3	<50	Centre		HR+/HER2+
3	<50	NordAT		HR+/HER2-
2	<50	Sud		HR+/HER2+
4	<50	Centre		
3	[50-74]	nordca		HR+/HER2-
2	[50-74]	NordAT		HR+/HER2+
2	<50	Centre		HR-/HER2+
3	<50	NordAT		HR+/HER2-
3	[50-74]	Sud		
3	[50-74]	Centre		HR+/HER2+
2	[50-74]	nordca		HR+/HER2-

3	<50	Sud		HR+/HER2-
3	[50-74]	Centre		TNBC
4	<50	Centre		HR+/HER2-
3	<50	NordAT		HR+/HER2-
3	[50-74]	Sud		HR+/HER2-
2	<50	Centre		HR+/HER2-
3	<50	Sud		HR+/HER2-
3	<50	NordAT		HR+/HER2-
3	<50	Centre		HR+/HER2+
2	<50	Sud		HR+/HER2-
3	<50	Centre		HR+/HER2+
3	<50	NordAT		HR+/HER2-
3	<50	Sud		TNBC
3	<50	Sud		HR-/HER2+
3	<50	Sud		HR+/HER2-
3	<50	NordAT		HR+/HER2-
3	<50	NordAT		HR-/HER2+
9	<50	Sud		HR+/HER2-
3	<50	NordAT		TNBC
3	<50	Sud		HR+/HER2-
2	<50	Sud		HR+/HER2-
3	<50	Centre		HR+/HER2+
3	<50	Centre		
1	[50-74]	nordca		HR+/HER2-
2	<50	Sud		HR+/HER2-
3	<50	Sud		HR+/HER2-
1	<50	NordAT		TNBC
3	<50	Centre		HR+/HER2-
2	<50	Centre		HR+/HER2-
3	<50	NordAT		HR+/HER2-
2	[50-74]	nordca		HR+/HER2-
3	<50	NordAT		HR+/HER2-
3	[50-74]	NordAT		HR+/HER2-
3	<50	Sud		TNBC
2	[50-74]	Sud		HR+/HER2-
2	[50-74]	NordAT		
2	<50	Centre		HR+/HER2-
	<50	nordca		HR+/HER2-
3	<50	Centre		HR+/HER2-
	<50	NordAT		HR+/HER2-
3	<50	nordca		HR+/HER2-
3	<50	nordca		HR+/HER2-
3	<50	Centre		HR+/HER2-
2	<50	Sud		HR+/HER2-
3	[50-74]	Sud		HR+/HER2-
	<50	nordca		HR+/HER2-
3	<50	Sud		HR+/HER2-
2	<50	nordca		HR+/HER2-
3	<50	Sud		HR+/HER2-
3	<50	NordAT		HR+/HER2-

3	<50	Centre		HR+/HER2-
9	<50	NordAT		HR+/HER2-
2	<50	NordAT		HR+/HER2-
3	<50	Centre		
2	<50	Centre		HR+/HER2-
3	<50	Centre		TNBC
3	<50	Sud		TNBC
2	[50-74]	Centre		HR+/HER2-
3	<50	Centre		HR-/HER2+
3	[50-74]	Centre		HR+/HER2-
9	<50	Sud		
3	<50	Centre		HR-/HER2+
3	<50	Centre		HR+/HER2-
3	<50	Sud		HR+/HER2-
3	<50	Centre		
3	<50	Centre		HR+/HER2-
3	<50	Centre		HR+/HER2-
3	[50-74]	NordAT		HR+/HER2-
3	<50	NordAT		
3	[50-74]	Centre		HR+/HER2-
3	<50	Centre		HR+/HER2-
2	[50-74]	Centre		HR+/HER2-
3	<50	NordAT		HR+/HER2-
3	<50	Centre		HR+/HER2-
3	<50	NordAT		TNBC
3	<50	NordAT		
3	<50	Sud		HR+/HER2-
3	<50	NordAT		HR+/HER2+
3	<50	Sud		HR+/HER2-
3	<50	Centre		HR+/HER2-
3	<50	Centre		
3	[50-74]	Sud		HR+/HER2-
3	[50-74]	Sud		HR+/HER2-
3	<50	NordAT		TNBC
3	<50	Centre		HR+/HER2-
3	[50-74]	nordca		TNBC
3	[50-74]	Sud		TNBC
3	<50	NordAT		HR-/HER2+
2	<50	Centre		HR+/HER2+
3	<50	Sud		HR+/HER2+
2	<50	Centre		HR+/HER2-
1	<50	Sud		HR+/HER2-
3	[50-74]	Centre		HR+/HER2-
	<50	Sud		HR+/HER2-
3	[50-74]	NordAT		HR+/HER2-
1	<50	NordAT		HR+/HER2-
3	[50-74]	nordca		TNBC
3	<50	Sud		TNBC
9	[50-74]	Sud		TNBC
3	<50	NordAT		HR+/HER2+

3	<50	NordAT		HR+/HER2-
3	<50	NordAT		HR-/HER2+
3	<50	NordAT		TNBC
9	<50	NordAT		TNBC
3	<50	NordAT		TNBC
	[50-74]	Centre		HR+/HER2-
3	<50	Centre		HR+/HER2+
2	<50	Sud		HR+/HER2-
3	<50	Sud		HR+/HER2-
3	[50-74]	Centre		HR-/HER2+
1	[50-74]	nordca		HR+/HER2-
3	[50-74]	NordAT		HR-/HER2+
3	<50	Centre		TNBC
3	<50	Sud		HR+/HER2-
3	<50	Centre		HR+/HER2-
1	[50-74]	Centre		HR-/HER2+
2	[50-74]	Centre		HR+/HER2-
3	<50	Centre		HR+/HER2-
	[50-74]	Sud		TNBC
2	<50	Sud		
3	[50-74]	Sud		TNBC
9	<50	Sud		TNBC
3	<50	Centre		HR+/HER2+
3	<50	NordAT		HR-/HER2+
2	<50	Sud		HR-/HER2+
3	[50-74]	Sud		HR+/HER2-
3	[50-74]	Sud		HR+/HER2-
3	[50-74]	NordAT		HR+/HER2-
9	<50	Centre		TNBC
3	<50	Sud		
3	<50	Sud		HR+/HER2-
3	<50	Sud		TNBC
3	<50	Centre		HR+/HER2+
3	<50	Sud		HR+/HER2+
3	<50	NordAT		TNBC
3	<50	NordAT		HR+/HER2-
3	<50	Sud		HR+/HER2-
3	[50-74]	Sud		HR+/HER2-
1	<50	Sud		HR+/HER2-
3	<50	nordca		HR+/HER2+
3	<50	Centre		HR+/HER2+
9	[50-74]	Centre		
2	<50	Centre		HR+/HER2+
3	<50	Centre		TNBC
2	<50	Centre		HR+/HER2+
3	[50-74]	Sud		HR+/HER2-
3	<50	NordAT		HR+/HER2-
3	<50	Sud		HR+/HER2-
	<50	Centre		HR+/HER2-
3	<50	Sud		HR+/HER2-

3	<50	Centre		HR+/HER2+
3	<50	Centre		HR+/HER2-
3	<50	NordAT		HR-/HER2+
2	[50-74]	Sud		HR+/HER2-
2	<50	NordAT		TNBC
3	<50	Centre		HR+/HER2-
3	[50-74]	nordca		HR+/HER2-
	<50	Centre		HR+/HER2-
3	[50-74]	NordAT		HR+/HER2-
2	<50	Centre		TNBC
3	[50-74]	Centre		TNBC
3	<50	Centre		TNBC
3	<50	nordca		HR+/HER2-
3	[50-74]	Sud		HR+/HER2-
3	[50-74]	Sud		HR+/HER2-
3	[50-74]	Centre		
3	[50-74]	Centre		HR-/HER2+
3	<50	Centre		HR+/HER2+
3	>=75	Centre		HR+/HER2+
3	>=75	Centre		HR+/HER2-
3	>=75	Centre		HR+/HER2-
3	>=75	Centre		HR+/HER2-
3	>=75	Sud		
3	>=75	Centre		
3	>=75	NordAT		HR+/HER2-
3	>=75	NordAT		HR+/HER2-
1	>=75	Centre		HR+/HER2-
1	>=75	Centre		HR+/HER2-
3	>=75	Centre		HR+/HER2-
3	>=75	NordAT		
2	>=75	Sud		HR+/HER2-
3	>=75	Sud		HR+/HER2-
3	>=75	Centre		HR+/HER2-
3	>=75	Sud		TNBC
9	>=75	Sud		HR+/HER2-
3	>=75	NordAT		TNBC
3	>=75	Centre		HR+/HER2-
9	>=75	Centre		HR+/HER2-
3	>=75	Sud		
3	>=75	Centre		HR-/HER2+
2	>=75	Sud		HR+/HER2-
3	>=75	nordca		
3	>=75	Centre		
2	>=75	NordAT		HR+/HER2-
3	>=75	Sud		
3	>=75	Centre		
3	>=75	Centre		
3	>=75	Centre		HR-/HER2+
2	>=75	Centre		
3	>=75	Sud		HR+/HER2+

2	>=75	Centre		TNBC
2	>=75	Centre		HR+/HER2-
3	>=75	Centre		TNBC
3	>=75	Centre		HR+/HER2-
2	>=75	NordAT		
3	>=75	Centre		
3	<50	Centre	Localized/ local spread	TNBC
1	<50	Sud	Localized/ local spread	HR+/HER2-
3	<50	Centre	Localized/ local spread	TNBC
3	<50	Centre	Localized/ local spread	HR+/HER2+
3	<50	Sud	Localized/ local spread	HR+/HER2-
3	<50	NordAT	Localized/ local spread	HR+/HER2-
3	<50	Centre	Regional	TNBC
3	<50	Centre	Localized/ local spread	HR+/HER2-
2	<50	NordAT	Localized/ local spread	HR+/HER2-
2	<50	Sud	Localized/ local spread	HR+/HER2-
3	<50	Centre	Localized/ local spread	HR+/HER2-
3	<50	Centre	Localized/ local spread	TNBC
2	<50	Centre	Localized/ local spread	HR+/HER2+
2	<50	Sud	Localized/ local spread	HR+/HER2-
3	<50	Sud	Localized/ local spread	TNBC
3	<50	NordAT	Localized/ local spread	HR+/HER2-
3	<50	Sud	Localized/ local spread	HR+/HER2-
2	<50	Sud	Localized/ local spread	HR+/HER2-
2	<50	NordAT	Localized/ local spread	HR+/HER2-
3	<50	Centre	Regional	HR+/HER2-
3	<50	Sud	Localized/ local spread	TNBC
2	<50	NordAT	Localized/ local spread	HR+/HER2+
3	<50	Sud	Localized/ local spread	HR-/HER2+
2	<50	Centre	Localized/ local spread	HR+/HER2-
	<50	Sud	Localized/ local spread	HR+/HER2+
3	<50	Centre	Localized/ local spread	HR+/HER2-
3	<50	Sud	Localized/ local spread	HR+/HER2+
3	<50	NordAT	Localized/ local spread	HR+/HER2+
3	<50	Sud	Localized/ local spread	HR+/HER2-
3	<50	NordAT	Localized/ local spread	HR+/HER2-
3	<50	NordAT	Localized/ local spread	TNBC
2	<50	Centre	Localized/ local spread	HR+/HER2-
2	<50	Sud	Localized/ local spread	HR+/HER2-
2	<50	Centre	Localized/ local spread	TNBC
2	<50	NordAT	Localized/ local spread	HR+/HER2-
1	<50	Sud	Localized/ local spread	HR+/HER2-
3	<50	NordAT	Localized/ local spread	HR+/HER2-
3	<50	Sud	Localized/ local spread	HR+/HER2-
3	<50	Centre	Localized/ local spread	HR+/HER2-
2	<50	Centre	Localized/ local spread	HR+/HER2-
3	<50	Centre	Localized/ local spread	HR+/HER2-
3	<50	Centre	Localized/ local spread	TNBC
3	<50	nordca	Localized/ local spread	HR+/HER2-
3	<50	Centre	Localized/ local spread	TNBC

3	<50	Centre	Localized/ local spread	TNBC
3	<50	Centre	Localized/ local spread	HR+/HER2+
2	<50	Centre	Localized/ local spread	HR+/HER2-
3	<50	NordAT	Regional	HR+/HER2-
3	<50	Centre	Localized/ local spread	HR+/HER2+
3	<50	Sud	Localized/ local spread	TNBC
3	<50	Centre	Regional	TNBC
3	<50	Centre	Localized/ local spread	HR+/HER2-
3	<50	NordAT	Localized/ local spread	HR+/HER2-
2	<50	Centre	Localized/ local spread	HR+/HER2-
3	<50	NordAT	Localized/ local spread	HR+/HER2-
3	<50	Sud	Localized/ local spread	HR+/HER2-
3	<50	NordAT	Localized/ local spread	HR+/HER2-
2	<50	NordAT	Localized/ local spread	HR+/HER2-
3	<50	Sud	Localized/ local spread	HR-/HER2+
3	<50	NordAT	Localized/ local spread	HR+/HER2-
3	<50	Sud	Localized/ local spread	HR+/HER2-
3	<50	Centre	Localized/ local spread	
3	<50	NordAT	Localized/ local spread	HR+/HER2-
3	<50	Centre	Localized/ local spread	TNBC
2	<50	Centre	Localized/ local spread	
3	<50	nordca	Localized/ local spread	HR+/HER2+
3	<50	Sud	Localized/ local spread	HR+/HER2+
3	<50	Centre	Localized/ local spread	HR+/HER2-
9	<50	NordAT	Localized/ local spread	
2	<50	Sud	Localized/ local spread	
3	<50	Centre	Localized/ local spread	TNBC
3	<50	Centre	Localized/ local spread	HR+/HER2-
	<50	NordAT	Localized/ local spread	HR+/HER2+
3	<50	Sud	Localized/ local spread	HR-/HER2+
3	<50	Sud	Localized/ local spread	HR+/HER2-
9	<50	NordAT	Localized/ local spread	HR+/HER2-
3	<50	Sud	Localized/ local spread	HR+/HER2-
3	<50	Sud	Localized/ local spread	HR-/HER2+
3	<50	Centre	Localized/ local spread	HR+/HER2-
3	<50	Centre	Localized/ local spread	TNBC
3	<50	Centre	Localized/ local spread	HR-/HER2+
3	<50	Sud	Localized/ local spread	TNBC
3	<50	Sud	Localized/ local spread	HR+/HER2-
3	<50	NordAT	Localized/ local spread	TNBC
3	<50	Centre	Localized/ local spread	TNBC
	<50	Sud	Localized/ local spread	HR+/HER2-
3	<50	NordAT	Localized/ local spread	HR+/HER2+
	<50	NordAT	Regional	HR+/HER2-
3	<50	Centre	Localized/ local spread	HR-/HER2+
3	<50	Centre	Localized/ local spread	HR+/HER2-
3	<50	Sud	Localized/ local spread	TNBC
3	<50	NordAT	Localized/ local spread	HR+/HER2-
3	<50	Sud	Localized/ local spread	
3	<50	Sud	Localized/ local spread	HR+/HER2-

2	<50	Centre	Localized/ local spread	
3	<50	Centre	Localized/ local spread	
3	<50	Sud	Localized/ local spread	HR+/HER2-
3	<50	Centre	Localized/ local spread	HR+/HER2+
3	<50	Sud	Localized/ local spread	HR-/HER2+
3	<50	Centre	Localized/ local spread	HR+/HER2-
2	<50	NordAT	Localized/ local spread	HR+/HER2+
9	<50	Sud	Regional	HR+/HER2+
2	<50	NordAT	Localized/ local spread	
3	<50	Sud	Localized/ local spread	HR+/HER2-
9	<50	Sud	Localized/ local spread	HR+/HER2-
3	<50	Sud	Localized/ local spread	HR+/HER2-
2	<50	Sud	Localized/ local spread	HR+/HER2+
3	<50	Centre	Localized/ local spread	HR+/HER2-
3	<50	Centre	Localized/ local spread	HR+/HER2-
2	<50	Sud	Localized/ local spread	HR+/HER2-
3	<50	Centre	Localized/ local spread	HR-/HER2+
2	<50	Sud	Localized/ local spread	HR+/HER2-
3	<50	NordAT	Localized/ local spread	HR-/HER2+
3	<50	nordca	Localized/ local spread	HR+/HER2-
3	<50	Centre	Localized/ local spread	HR+/HER2+
3	<50	Centre	Localized/ local spread	HR+/HER2-
3	<50	nordca	Localized/ local spread	HR-/HER2+
3	<50	Centre	Localized/ local spread	TNBC
3	<50	Centre	Localized/ local spread	TNBC
2	<50	Sud	Localized/ local spread	HR+/HER2-
3	<50	Centre	Localized/ local spread	HR+/HER2-
3	<50	Sud	Localized/ local spread	HR+/HER2-
3	<50	Centre	Localized/ local spread	HR-/HER2+
2	<50	Centre	Localized/ local spread	HR+/HER2-
3	<50	Centre	Regional	HR+/HER2-
3	<50	Sud	Localized/ local spread	TNBC
3	<50	Sud	Localized/ local spread	TNBC
3	<50	Sud	Localized/ local spread	HR+/HER2-
3	<50	Centre	Localized/ local spread	HR+/HER2-
3	<50	Sud	Localized/ local spread	HR+/HER2-
3	<50	Centre	Localized/ local spread	TNBC
2	<50	Centre	Localized/ local spread	HR+/HER2-
9	<50	Sud	Localized/ local spread	
3	<50	Centre	Localized/ local spread	HR+/HER2-
3	<50	Centre	Localized/ local spread	HR+/HER2+
3	<50	nordca	Localized/ local spread	HR+/HER2-
3	<50	Sud	Localized/ local spread	HR+/HER2-
9	<50	Centre	Localized/ local spread	HR-/HER2+
3	<50	Sud	Localized/ local spread	HR-/HER2+
3	<50	Centre	Localized/ local spread	HR+/HER2-
3	<50	Centre	Localized/ local spread	TNBC
3	<50	Centre	Localized/ local spread	HR+/HER2+
2	<50	NordAT	Localized/ local spread	HR+/HER2+
2	<50	Centre	Localized/ local spread	HR+/HER2-

3	<50	Centre	Localized/ local spread	TNBC
3	<50	NordAT	Localized/ local spread	TNBC
2	<50	NordAT	Localized/ local spread	
3	<50	Centre	Localized/ local spread	HR+/HER2-
1	<50	Sud	Localized/ local spread	HR+/HER2-
3	<50	Centre	Localized/ local spread	HR+/HER2-
3	<50	NordAT	Localized/ local spread	TNBC
1	<50	Centre	Localized/ local spread	HR+/HER2-
3	<50	Sud	Localized/ local spread	HR-/HER2+
2	<50	Centre	Localized/ local spread	HR+/HER2+
3	<50	Centre	Localized/ local spread	HR+/HER2-
1	<50	Sud	Localized/ local spread	HR+/HER2+
2	<50	NordAT	Regional	HR+/HER2+
3	<50	Centre	Localized/ local spread	HR+/HER2-
3	<50	Centre	Localized/ local spread	HR+/HER2-
3	<50	NordAT	Localized/ local spread	HR-/HER2+
2	<50	Sud	Regional	HR+/HER2-
2	<50	Sud	Localized/ local spread	HR+/HER2+
3	<50	Centre	Localized/ local spread	HR+/HER2+
3	<50	Sud	Localized/ local spread	HR+/HER2-
3	<50	Centre	Localized/ local spread	HR+/HER2+
3	<50	Sud	Localized/ local spread	HR+/HER2-
3	<50	NordAT	Localized/ local spread	HR+/HER2-
3	<50	NordAT	Localized/ local spread	TNBC
3	<50	NordAT	Localized/ local spread	HR+/HER2-
2	<50	Centre	Localized/ local spread	
3	<50	Centre	Localized/ local spread	HR+/HER2+
3	<50	Sud	Localized/ local spread	HR+/HER2+
3	<50	Sud	Localized/ local spread	TNBC
3	<50	Centre	Localized/ local spread	TNBC
3	<50	nordca	Localized/ local spread	HR+/HER2-
2	<50	Centre	Localized/ local spread	HR+/HER2-
3	<50	Sud	Localized/ local spread	HR+/HER2-
3	<50	Sud	Localized/ local spread	HR+/HER2+
3	<50	NordAT	Localized/ local spread	HR-/HER2+
3	<50	NordAT	Localized/ local spread	HR+/HER2-
9	<50	Centre	Localized/ local spread	HR-/HER2+
2	<50	Centre	Localized/ local spread	HR-/HER2+
3	<50	Centre	Localized/ local spread	HR+/HER2-
3	<50	Sud	Localized/ local spread	HR+/HER2+
2	<50	Sud	Localized/ local spread	HR+/HER2+
3	<50	Centre	Localized/ local spread	HR+/HER2+
3	<50	Centre	Localized/ local spread	TNBC
9	<50	Centre	Localized/ local spread	HR+/HER2-
3	<50	Sud	Localized/ local spread	HR+/HER2-
2	<50	Centre	Localized/ local spread	HR+/HER2-
3	<50	NordAT	Localized/ local spread	HR+/HER2-
3	<50	Centre	Localized/ local spread	TNBC
3	<50	Centre	Localized/ local spread	HR+/HER2+
3	<50	Sud	Localized/ local spread	HR+/HER2-

3	<50	Sud	Localized/ local spread	TNBC
2	<50	Sud	Localized/ local spread	HR+/HER2-
1	<50	Sud	Localized/ local spread	HR+/HER2-
2	<50	NordAT	Localized/ local spread	HR-/HER2+
3	<50	NordAT	Localized/ local spread	HR+/HER2-
3	<50	Centre	Localized/ local spread	HR+/HER2-
2	<50	Centre	Localized/ local spread	HR+/HER2-
9	<50	NordAT	Localized/ local spread	
3	<50	Sud	Localized/ local spread	HR+/HER2-
3	<50	NordAT	Localized/ local spread	TNBC
2	<50	Sud	Localized/ local spread	HR+/HER2+
3	<50	Centre	Localized/ local spread	HR+/HER2+
2	<50	Sud	Localized/ local spread	HR+/HER2-
3	<50	Centre	Localized/ local spread	HR+/HER2-
3	<50	Centre	Localized/ local spread	TNBC
3	<50	Sud	Localized/ local spread	HR+/HER2+
3	<50	NordAT	Localized/ local spread	HR+/HER2+
1	<50	NordAT	Localized/ local spread	HR+/HER2-
3	<50	Centre	Localized/ local spread	TNBC
3	<50	Centre	Localized/ local spread	HR+/HER2-
3	<50	Centre	Localized/ local spread	HR+/HER2-
3	<50	Sud	Localized/ local spread	HR+/HER2-
1	<50	Sud	Localized/ local spread	HR+/HER2-
1	<50	NordAT	Localized/ local spread	HR+/HER2-
2	<50	NordAT	Localized/ local spread	HR+/HER2-
3	<50	Sud	Localized/ local spread	HR-/HER2+
3	<50	Centre	Localized/ local spread	HR+/HER2-
3	<50	NordAT	Localized/ local spread	TNBC
3	<50	Centre	Localized/ local spread	HR+/HER2-
3	<50	Centre	Localized/ local spread	HR+/HER2-
3	<50	NordAT	Localized/ local spread	TNBC
2	<50	Sud	Localized/ local spread	HR+/HER2-
3	<50	Sud	Localized/ local spread	HR+/HER2-
3	<50	Centre	Localized/ local spread	HR+/HER2-
3	<50	Sud	Localized/ local spread	TNBC
3	<50	Centre	Localized/ local spread	HR+/HER2-
3	<50	Centre	Localized/ local spread	HR+/HER2-
3	<50	Centre	Localized/ local spread	HR+/HER2+
3	<50	Centre	Localized/ local spread	HR-/HER2+
3	<50	Sud	Localized/ local spread	HR+/HER2-
3	<50	Sud	Localized/ local spread	TNBC
3	<50	Sud	Localized/ local spread	HR+/HER2-
3	<50	NordAT	Localized/ local spread	TNBC
9	<50	Centre	Localized/ local spread	HR+/HER2-
1	<50	Sud	Localized/ local spread	HR+/HER2-
1	<50	Centre	Localized/ local spread	HR+/HER2-
2	<50	nordca	Localized/ local spread	HR+/HER2-
3	<50	Centre	Localized/ local spread	HR+/HER2-
3	<50	Centre	Localized/ local spread	HR+/HER2-
3	<50	Centre	Localized/ local spread	HR+/HER2+

3	<50	Centre	Localized/ local spread	HR+/HER2-
3	<50	Centre	Localized/ local spread	HR+/HER2+
3	<50	NordAT	Localized/ local spread	HR+/HER2+
2	<50	NordAT	Localized/ local spread	HR-/HER2+
3	<50	Centre	Localized/ local spread	TNBC
2	<50	NordAT	Localized/ local spread	HR+/HER2+
3	<50	nordca	Localized/ local spread	TNBC
3	<50	Centre	Localized/ local spread	HR+/HER2-
3	<50	Centre	Localized/ local spread	HR+/HER2-
1	<50	Centre	Localized/ local spread	HR+/HER2-
2	<50	Sud	Localized/ local spread	HR-/HER2+
3	<50	nordca	Localized/ local spread	HR+/HER2-
3	<50	Centre	Localized/ local spread	TNBC
3	<50	NordAT	Localized/ local spread	HR+/HER2+
3	<50	Centre	Localized/ local spread	HR+/HER2-
3	<50	Sud	Localized/ local spread	HR+/HER2-
3	<50	Centre	Localized/ local spread	HR+/HER2-
3	<50	Centre	Localized/ local spread	HR+/HER2-
3	<50	Sud	Localized/ local spread	HR+/HER2-
3	<50	nordca	Localized/ local spread	HR+/HER2-
3	<50	Centre	Localized/ local spread	HR+/HER2+
2	<50	Centre	Localized/ local spread	HR+/HER2+
2	<50	Sud	Localized/ local spread	HR+/HER2-
1	<50	Centre	Localized/ local spread	HR+/HER2-
3	<50	Sud	Localized/ local spread	TNBC
3	<50	Centre	Localized/ local spread	HR+/HER2-
3	<50	nordca	Localized/ local spread	TNBC
2	<50	NordAT	Localized/ local spread	HR+/HER2+
2	<50	NordAT	Localized/ local spread	HR+/HER2-
3	<50	Sud	Localized/ local spread	HR+/HER2+
3	<50	Centre	Localized/ local spread	TNBC
3	<50	NordAT	Localized/ local spread	HR+/HER2-
3	<50	Centre	Localized/ local spread	TNBC
3	<50	Centre	Localized/ local spread	HR+/HER2-
3	<50	Centre	Localized/ local spread	HR+/HER2-
2	<50	Centre	Localized/ local spread	HR+/HER2-
3	<50	Sud	Localized/ local spread	HR+/HER2-
3	<50	Sud	Localized/ local spread	HR+/HER2+
3	<50	Centre	Localized/ local spread	TNBC
3	<50	Centre	Localized/ local spread	TNBC
1	<50	Centre	Localized/ local spread	TNBC
3	<50	Sud	Localized/ local spread	HR+/HER2-
2	<50	NordAT	Localized/ local spread	HR+/HER2+
3	<50	Sud	Localized/ local spread	HR+/HER2+
3	<50	NordAT	Localized/ local spread	HR+/HER2-
3	<50	NordAT	Localized/ local spread	HR+/HER2-
3	<50	NordAT	Localized/ local spread	HR+/HER2-
3	<50	Centre	Localized/ local spread	HR+/HER2-
3	<50	Centre	Localized/ local spread	HR-/HER2+
3	<50	Centre	Localized/ local spread	HR+/HER2-

2	<50	Sud	Localized/ local spread	HR+/HER2+
3	<50	Centre	Localized/ local spread	HR+/HER2-
4	<50	Centre	Localized/ local spread	TNBC
3	<50	Sud	Localized/ local spread	HR+/HER2-
2	<50	Centre	Localized/ local spread	HR+/HER2-
3	<50	Centre	Localized/ local spread	HR-/HER2+
2	[50-74]	Centre	Localized/ local spread	HR+/HER2-
3	<50	Centre	Localized/ local spread	
2	[50-74]	Centre	Localized/ local spread	TNBC
3	<50	Sud	Localized/ local spread	HR+/HER2-
3	<50	nordca	Localized/ local spread	TNBC
3	[50-74]	NordAT	Localized/ local spread	HR+/HER2-
3	<50	Centre	Localized/ local spread	HR+/HER2-
9	[50-74]	NordAT	Localized/ local spread	
3	<50	NordAT	Localized/ local spread	HR-/HER2+
3	[50-74]	NordAT	Localized/ local spread	HR+/HER2-
1	[50-74]	Centre	Localized/ local spread	HR+/HER2-
3	<50	NordAT	Localized/ local spread	HR+/HER2-
	<50	Centre	Regional	TNBC
3	<50	NordAT	Regional	TNBC
3	[50-74]	Sud	Localized/ local spread	TNBC
3	<50	NordAT	Localized/ local spread	HR+/HER2+
2	<50	Centre	Localized/ local spread	HR-/HER2+
3	[50-74]	Centre	Localized/ local spread	HR+/HER2-
3	[50-74]	Centre	Localized/ local spread	HR+/HER2-
3	[50-74]	Sud	Localized/ local spread	HR+/HER2-
3	[50-74]	Sud	Localized/ local spread	HR+/HER2-
2	<50	NordAT	Localized/ local spread	HR+/HER2-
2	<50	Centre	Localized/ local spread	HR+/HER2-
3	[50-74]	Centre	Localized/ local spread	HR+/HER2+
3	<50	NordAT	Localized/ local spread	
3	<50	NordAT	Localized/ local spread	TNBC
3	<50	Sud	Localized/ local spread	TNBC
9	<50	Centre	Localized/ local spread	TNBC
3	<50	Sud	Localized/ local spread	TNBC
3	<50	nordca	Localized/ local spread	TNBC
2	<50	Sud	Localized/ local spread	HR+/HER2-
3	<50	Sud	Localized/ local spread	HR+/HER2-
1	<50	NordAT	Localized/ local spread	HR+/HER2-
3	<50	Centre	Localized/ local spread	TNBC
2	[50-74]	Centre	Localized/ local spread	HR+/HER2-
3	<50	nordca	Localized/ local spread	HR+/HER2-
	<50	Sud	Localized/ local spread	HR+/HER2-
2	<50	Sud	Localized/ local spread	HR+/HER2-
3	<50	Centre	Localized/ local spread	
3	<50	Sud	Localized/ local spread	
3	<50	Sud	Localized/ local spread	HR+/HER2+
3	<50	Sud	Localized/ local spread	HR+/HER2-
3	[50-74]	NordAT	Localized/ local spread	HR+/HER2-
3	<50	Centre	Localized/ local spread	HR+/HER2-

3	[50-74]	Centre	Localized/ local spread	HR+/HER2-
3	<50	Sud	Localized/ local spread	HR+/HER2-
3	[50-74]	NordAT	Localized/ local spread	HR+/HER2-
2	<50	Centre	Localized/ local spread	HR+/HER2-
	<50	NordAT	Localized/ local spread	HR+/HER2-
3	<50	NordAT	Localized/ local spread	HR+/HER2-
3	<50	Centre	Localized/ local spread	HR+/HER2-
3	<50	Centre	Localized/ local spread	TNBC
2	<50	Sud	Localized/ local spread	HR+/HER2-
3	<50	NordAT	Localized/ local spread	HR+/HER2-
2	<50	NordAT	Localized/ local spread	HR+/HER2-
3	[50-74]	Centre	Localized/ local spread	HR+/HER2-
3	<50	Centre	Localized/ local spread	TNBC
3	<50	Sud	Localized/ local spread	HR+/HER2+
1	<50	Sud	Localized/ local spread	HR+/HER2-
3	[50-74]	NordAT	Localized/ local spread	HR+/HER2-
3	<50	Centre	Localized/ local spread	HR+/HER2-
3	[50-74]	Sud	Localized/ local spread	HR+/HER2-
3	<50	Centre	Localized/ local spread	HR+/HER2-
3	<50	Sud	Localized/ local spread	HR+/HER2+
2	<50	Centre	Localized/ local spread	HR+/HER2-
3	[50-74]	Sud	Localized/ local spread	
2	[50-74]	Sud	Localized/ local spread	HR+/HER2-
3	<50	Centre	Localized/ local spread	HR+/HER2+
2	<50	Sud	Localized/ local spread	HR+/HER2-
3	<50	Sud	Localized/ local spread	HR+/HER2+
3	<50	NordAT	Localized/ local spread	HR+/HER2-
3	[50-74]	Centre	Localized/ local spread	HR+/HER2-
3	<50	Sud	Localized/ local spread	
9	<50	NordAT	Localized/ local spread	
3	<50	Centre	Localized/ local spread	
3	[50-74]	Sud	Localized/ local spread	TNBC
3	<50	Centre	Localized/ local spread	HR+/HER2-
3	<50	Centre	Localized/ local spread	HR+/HER2+
3	[50-74]	Sud	Localized/ local spread	HR+/HER2-
2	<50	Centre	Localized/ local spread	HR-/HER2+
3	<50	Centre	Regional	TNBC
3	<50	Sud	Localized/ local spread	HR+/HER2-
3	[50-74]	Sud	Localized/ local spread	TNBC
3	[50-74]	Sud	Localized/ local spread	HR+/HER2+
1	[50-74]	NordAT	Localized/ local spread	HR+/HER2-
2	[50-74]	Sud	Localized/ local spread	HR+/HER2-
3	[50-74]	Sud	Localized/ local spread	HR+/HER2-
3	[50-74]	nordca	Localized/ local spread	HR+/HER2+
3	[50-74]	Centre	Localized/ local spread	HR+/HER2-
3	<50	NordAT	Localized/ local spread	HR+/HER2-
3	<50	Centre	Localized/ local spread	HR+/HER2-
3	<50	NordAT	Localized/ local spread	HR-/HER2+
3	[50-74]	Centre	Localized/ local spread	HR+/HER2+
3	<50	NordAT	Localized/ local spread	TNBC

3	<50	Centre	Localized/ local spread	TNBC
4	<50	Centre	Regional	HR+/HER2+
2	<50	Sud	Localized/ local spread	
3	<50	Centre	Regional	HR+/HER2+
4	<50	NordAT	Localized/ local spread	TNBC
3	[50-74]	Centre	Localized/ local spread	HR+/HER2-
3	[50-74]	Centre	Localized/ local spread	HR+/HER2-
2	[50-74]	Sud	Localized/ local spread	HR+/HER2-
2	<50	Centre	Localized/ local spread	HR+/HER2-
3	<50	Sud	Localized/ local spread	HR+/HER2-
3	<50	Centre	Localized/ local spread	TNBC
3	[50-74]	NordAT	Localized/ local spread	HR+/HER2+
3	[50-74]	Sud	Regional	HR-/HER2+
3	[50-74]	NordAT	Localized/ local spread	HR+/HER2-
3	[50-74]	Centre	Localized/ local spread	HR+/HER2-
	<50	NordAT	Localized/ local spread	
3	<50	Centre	Localized/ local spread	HR+/HER2-
3	<50	nordca	Localized/ local spread	TNBC
2	<50	Sud	Localized/ local spread	HR+/HER2+
3	<50	Sud	Localized/ local spread	
3	<50	nordca	Localized/ local spread	HR+/HER2-
3	[50-74]	Centre	Regional	TNBC
3	<50	Sud	Localized/ local spread	HR+/HER2+
3	[50-74]	Centre	Localized/ local spread	HR+/HER2-
3	<50	Centre	Regional	TNBC
1	<50	Centre	Localized/ local spread	HR+/HER2-
3	<50	Centre	Localized/ local spread	HR-/HER2+
2	<50	NordAT	Localized/ local spread	HR+/HER2-
3	<50	Sud	Localized/ local spread	TNBC
3	<50	nordca	Localized/ local spread	TNBC
3	<50	Centre	Localized/ local spread	TNBC
3	[50-74]	Centre	Regional	HR+/HER2-
2	[50-74]	Sud	Localized/ local spread	HR-/HER2+
2	<50	Centre	Localized/ local spread	HR+/HER2+
3	<50	Centre	Localized/ local spread	HR+/HER2+
3	[50-74]	NordAT	Localized/ local spread	TNBC
3	[50-74]	NordAT	Localized/ local spread	HR+/HER2+
9	<50	Centre	Localized/ local spread	
3	[50-74]	nordca	Localized/ local spread	HR+/HER2-
	<50	Centre	Localized/ local spread	HR+/HER2-
3	[50-74]	Sud	Localized/ local spread	TNBC
2	<50	NordAT	Localized/ local spread	HR+/HER2+
3	<50	NordAT	Localized/ local spread	TNBC
2	<50	Centre	Localized/ local spread	HR+/HER2-
3	<50	Sud	Localized/ local spread	HR+/HER2-
3	<50	NordAT	Localized/ local spread	HR+/HER2-
3	[50-74]	Centre	Localized/ local spread	HR+/HER2+
3	<50	Sud	Localized/ local spread	HR+/HER2-
3	<50	Centre	Localized/ local spread	
3	<50	Centre	Localized/ local spread	TNBC

2	[50-74]	Centre	Localized/ local spread	HR+/HER2-
10	<50	Centre	Localized/ local spread	HR+/HER2-
2	<50	Sud	Localized/ local spread	HR+/HER2+
2	[50-74]	Centre	Localized/ local spread	HR+/HER2-
3	<50	NordAT	Localized/ local spread	
3	<50	NordAT	Localized/ local spread	HR+/HER2-
3	[50-74]	Centre	Localized/ local spread	HR+/HER2+
9	<50	Centre	Localized/ local spread	HR+/HER2-
3	<50	Sud	Localized/ local spread	HR-/HER2+
2	<50	nordca	Localized/ local spread	HR+/HER2+
	<50	Centre	Localized/ local spread	TNBC
2	[50-74]	Sud	Localized/ local spread	HR+/HER2-
3	<50	Centre	Localized/ local spread	HR+/HER2+
3	[50-74]	Centre	Regional	HR+/HER2+
3	[50-74]	Centre	Localized/ local spread	HR+/HER2-
3	<50	Centre	Localized/ local spread	TNBC
2	<50	Sud	Localized/ local spread	HR+/HER2+
3	[50-74]	Centre	Localized/ local spread	HR+/HER2-
	<50	Sud	Localized/ local spread	HR+/HER2-
3	<50	Centre	Localized/ local spread	HR+/HER2-
3	<50	Centre	Localized/ local spread	TNBC
2	<50	Centre	Localized/ local spread	HR+/HER2-
3	<50	Sud	Localized/ local spread	HR-/HER2+
3	<50	Sud	Localized/ local spread	HR+/HER2+
3	[50-74]	Centre	Localized/ local spread	HR+/HER2-
3	[50-74]	Centre	Localized/ local spread	TNBC
2	<50	Centre	Localized/ local spread	HR+/HER2-
2	[50-74]	Sud	Localized/ local spread	HR+/HER2-
2	<50	Sud	Localized/ local spread	HR+/HER2-
3	[50-74]	nordca	Localized/ local spread	HR+/HER2+
3	[50-74]	Centre	Localized/ local spread	HR+/HER2+
3	<50	Sud	Localized/ local spread	TNBC
3	<50	Centre	Localized/ local spread	HR+/HER2+
3	<50	Sud	Localized/ local spread	HR+/HER2-
	<50	Sud	Localized/ local spread	HR+/HER2-
9	[50-74]	Centre	Localized/ local spread	
3	[50-74]	Centre	Localized/ local spread	TNBC
3	<50	Centre	Localized/ local spread	HR+/HER2+
2	<50	Sud	Localized/ local spread	HR+/HER2-
2	[50-74]	Sud	Localized/ local spread	HR+/HER2-
3	<50	Sud	Localized/ local spread	HR+/HER2-
3	<50	Sud	Localized/ local spread	HR-/HER2+
3	<50	Centre	Localized/ local spread	HR+/HER2+
3	[50-74]	Centre	Localized/ local spread	HR+/HER2-
3	[50-74]	Centre	Localized/ local spread	HR+/HER2-
9	<50	Sud	Localized/ local spread	HR+/HER2-
2	<50	Sud	Localized/ local spread	HR+/HER2-
	<50	nordca	Localized/ local spread	HR+/HER2-
9	<50	Sud	Localized/ local spread	
9	[50-74]	Centre	Localized/ local spread	

2	<50	Sud	Localized/ local spread	HR+/HER2-
2	[50-74]	NordAT	Localized/ local spread	HR+/HER2-
3	<50	nordca	Localized/ local spread	TNBC
3	<50	Sud	Localized/ local spread	HR+/HER2+
2	<50	nordca	Localized/ local spread	HR+/HER2+
3	<50	Sud	Localized/ local spread	HR+/HER2+
2	<50	Sud	Localized/ local spread	HR+/HER2+
3	<50	nordca	Localized/ local spread	HR+/HER2+
1	<50	Sud	Localized/ local spread	HR+/HER2+
2	<50	Sud	Localized/ local spread	HR+/HER2+
3	[50-74]	Sud	Localized/ local spread	TNBC
3	<50	Centre	Localized/ local spread	HR+/HER2-
2	<50	Centre	Localized/ local spread	
2	<50	Centre	Localized/ local spread	HR+/HER2+
3	<50	Sud	Localized/ local spread	TNBC
3	<50	Sud	Localized/ local spread	HR+/HER2-
3	<50	Sud	Localized/ local spread	HR+/HER2-
3	<50	NordAT	Localized/ local spread	HR+/HER2-
2	<50	Centre	Localized/ local spread	HR+/HER2-
3	<50	Centre	Localized/ local spread	TNBC
3	<50	Sud	Localized/ local spread	TNBC
3	<50	NordAT	Localized/ local spread	HR-/HER2+
3	<50	Centre	Localized/ local spread	TNBC
3	<50	Centre	Localized/ local spread	
2	<50	Sud	Localized/ local spread	HR+/HER2-
2	<50	Centre	Localized/ local spread	HR+/HER2-
3	[50-74]	Centre	Localized/ local spread	TNBC
3	[50-74]	nordca	Localized/ local spread	TNBC
3	<50	Sud	Localized/ local spread	
3	<50	Centre	Localized/ local spread	HR+/HER2-
2	<50	Centre	Localized/ local spread	TNBC
2	<50	NordAT	Localized/ local spread	HR+/HER2+
3	<50	Centre	Localized/ local spread	HR+/HER2-
3	<50	Centre	Localized/ local spread	TNBC
3	<50	Centre	Localized/ local spread	HR+/HER2-
3	[50-74]	NordAT	Localized/ local spread	TNBC
1	<50	NordAT	Localized/ local spread	HR+/HER2-
3	<50	Centre	Localized/ local spread	HR+/HER2-
2	<50	Centre	Localized/ local spread	HR+/HER2-
3	<50	nordca	Localized/ local spread	HR+/HER2-
3	<50	NordAT	Localized/ local spread	HR+/HER2-
2	<50	Sud	Localized/ local spread	TNBC
2	<50	Centre	Localized/ local spread	HR+/HER2-
3	<50	Centre	Localized/ local spread	HR+/HER2-
9	[50-74]	Centre	Localized/ local spread	TNBC
2	[50-74]	NordAT	Localized/ local spread	HR+/HER2-
9	<50	NordAT	Localized/ local spread	
9	[50-74]	Sud	Localized/ local spread	HR+/HER2-
3	<50	Centre	Localized/ local spread	TNBC
2	[50-74]	Sud	Localized/ local spread	HR+/HER2-

2	<50	Sud	Localized/ local spread	HR+/HER2+
3	<50	Centre	Localized/ local spread	TNBC
3	<50	Centre	Localized/ local spread	TNBC
3	<50	NordAT	Localized/ local spread	TNBC
1	[50-74]	Centre	Localized/ local spread	HR+/HER2-
3	<50	nordca	Localized/ local spread	TNBC
3	[50-74]	Sud	Localized/ local spread	HR+/HER2-
2	<50	Sud	Localized/ local spread	HR+/HER2-
3	<50	nordca	Localized/ local spread	HR+/HER2-
3	<50	NordAT	Localized/ local spread	HR+/HER2-
3	[50-74]	Centre	Localized/ local spread	HR+/HER2+
3	<50	Centre	Localized/ local spread	HR-/HER2+
3	[50-74]	Sud	Localized/ local spread	TNBC
1	<50	Centre	Localized/ local spread	HR+/HER2-
2	[50-74]	Sud	Localized/ local spread	TNBC
3	<50	Centre	Localized/ local spread	
3	<50	Centre	Localized/ local spread	HR-/HER2+
3	<50	Centre	Localized/ local spread	HR+/HER2+
3	<50	Sud	Localized/ local spread	HR+/HER2-
3	<50	Sud	Localized/ local spread	HR+/HER2-
2	<50	Sud	Localized/ local spread	HR+/HER2-
3	[50-74]	Sud	Localized/ local spread	HR+/HER2-
2	<50	Centre	Localized/ local spread	HR+/HER2+
3	<50	Centre	Localized/ local spread	HR+/HER2+
3	<50	Sud	Localized/ local spread	TNBC
2	<50	Sud	Localized/ local spread	HR+/HER2-
3	<50	Sud	Localized/ local spread	HR+/HER2-
3	[50-74]	Centre	Localized/ local spread	TNBC
3	<50	NordAT	Localized/ local spread	HR+/HER2-
2	[50-74]	Sud	Localized/ local spread	HR+/HER2+
3	<50	Centre	Localized/ local spread	TNBC
2	[50-74]	nordca	Localized/ local spread	TNBC
3	<50	NordAT	Localized/ local spread	HR+/HER2-
2	<50	nordca	Localized/ local spread	HR+/HER2-
3	[50-74]	Centre	Localized/ local spread	HR+/HER2-
3	[50-74]	Sud	Localized/ local spread	HR+/HER2-
3	<50	Centre	Localized/ local spread	HR+/HER2-
3	[50-74]	Centre	Localized/ local spread	HR+/HER2-
2	[50-74]	Centre	Localized/ local spread	HR+/HER2-
3	<50	Sud	Localized/ local spread	HR+/HER2+
3	<50	Sud	Localized/ local spread	HR+/HER2+
3	[50-74]	Centre	Localized/ local spread	HR+/HER2+
1	<50	Sud	Localized/ local spread	HR+/HER2-
3	<50	Sud	Localized/ local spread	HR+/HER2+
2	[50-74]	Sud	Localized/ local spread	HR+/HER2-
3	[50-74]	NordAT	Localized/ local spread	TNBC
3	<50	Sud	Localized/ local spread	HR+/HER2-
3	<50	Centre	Localized/ local spread	HR-/HER2+
3	<50	NordAT	Localized/ local spread	HR+/HER2-
3	<50	Centre	Localized/ local spread	HR+/HER2-

3	<50	Centre	Localized/ local spread	HR+/HER2-
2	<50	Sud	Localized/ local spread	TNBC
3	<50	Sud	Localized/ local spread	HR+/HER2-
3	<50	Centre	Localized/ local spread	HR+/HER2-
3	<50	Centre	Localized/ local spread	HR+/HER2+
3	<50	Sud	Localized/ local spread	TNBC
3	<50	Sud	Localized/ local spread	HR+/HER2-
1	<50	Sud	Localized/ local spread	HR+/HER2-
2	<50	Centre	Localized/ local spread	HR+/HER2-
3	<50	Sud	Localized/ local spread	HR+/HER2-
3	<50	Sud	Localized/ local spread	TNBC
3	<50	Centre	Localized/ local spread	HR+/HER2+
3	<50	Centre	Localized/ local spread	TNBC
3	<50	Sud	Localized/ local spread	HR+/HER2+
2	<50	nordca	Localized/ local spread	HR+/HER2-
3	<50	Centre	Localized/ local spread	HR+/HER2-
2	<50	NordAT	Regional	HR+/HER2+
2	[50-74]	Sud	Localized/ local spread	HR+/HER2-
3	<50	NordAT	Localized/ local spread	TNBC
2	<50	Sud	Localized/ local spread	HR+/HER2-
3	[50-74]	nordca	Localized/ local spread	HR+/HER2-
2	[50-74]	Sud	Localized/ local spread	HR+/HER2-
3	<50	Centre	Localized/ local spread	HR+/HER2-
3	<50	Centre	Localized/ local spread	HR+/HER2-
3	[50-74]	Centre	Localized/ local spread	HR+/HER2-
1	<50	Centre	Localized/ local spread	HR+/HER2-
3	<50	Centre	Localized/ local spread	HR+/HER2-
3	<50	Sud	Localized/ local spread	HR+/HER2+
3	<50	Sud	Localized/ local spread	HR+/HER2-
3	<50	Centre	Localized/ local spread	HR+/HER2-
3	<50	NordAT	Localized/ local spread	HR-/HER2+
3	<50	Centre	Localized/ local spread	HR+/HER2+
3	<50	Centre	Localized/ local spread	HR+/HER2-
3	[50-74]	Centre	Localized/ local spread	HR+/HER2-
9	<50	NordAT	Localized/ local spread	HR+/HER2-
2	<50	NordAT	Localized/ local spread	HR+/HER2-
3	[50-74]	NordAT	Localized/ local spread	HR+/HER2-
3	[50-74]	Centre	Localized/ local spread	HR+/HER2+
2	[50-74]	nordca	Localized/ local spread	HR+/HER2-
3	<50	Centre	Localized/ local spread	HR+/HER2-
3	[50-74]	Sud	Localized/ local spread	HR+/HER2+
3	<50	Centre	Localized/ local spread	TNBC
3	[50-74]	Centre	Localized/ local spread	HR+/HER2-
3	<50	Sud	Localized/ local spread	HR+/HER2-
3	<50	NordAT	Localized/ local spread	HR+/HER2-
3	<50	NordAT	Localized/ local spread	TNBC
2	<50	NordAT	Localized/ local spread	HR+/HER2-
9	<50	Centre	Localized/ local spread	
2	[50-74]	Centre	Localized/ local spread	HR-/HER2+
3	<50	Centre	Localized/ local spread	HR+/HER2+

3	<50	Sud	Localized/ local spread	TNBC
3	<50	Centre	Localized/ local spread	HR-/HER2+
3	[50-74]	Sud	Localized/ local spread	HR-/HER2+
3	<50	Centre	Localized/ local spread	HR+/HER2-
3	<50	Sud	Localized/ local spread	
3	<50	Sud	Localized/ local spread	TNBC
2	[50-74]	Sud	Localized/ local spread	HR+/HER2-
3	<50	nordca	Localized/ local spread	HR+/HER2+
3	<50	Centre	Localized/ local spread	HR+/HER2-
3	<50	Sud	Localized/ local spread	HR+/HER2+
3	<50	Sud	Localized/ local spread	HR+/HER2-
2	[50-74]	Centre	Localized/ local spread	HR+/HER2-
2	<50	Sud	Localized/ local spread	TNBC
3	<50	Centre	Localized/ local spread	HR+/HER2-
3	<50	NordAT	Localized/ local spread	TNBC
3	[50-74]	Centre	Localized/ local spread	HR-/HER2+
2	<50	Sud	Localized/ local spread	HR+/HER2-
3	[50-74]	Centre	Localized/ local spread	HR+/HER2-
3	<50	Sud	Localized/ local spread	HR+/HER2-
3	<50	nordca	Localized/ local spread	HR+/HER2-
3	<50	Centre	Localized/ local spread	TNBC
3	[50-74]	NordAT	Localized/ local spread	HR+/HER2-
2	[50-74]	Centre	Localized/ local spread	HR+/HER2-
3	[50-74]	Centre	Localized/ local spread	HR+/HER2-
3	<50	NordAT	Localized/ local spread	TNBC
3	<50	Centre	Localized/ local spread	TNBC
3	[50-74]	NordAT	Localized/ local spread	HR+/HER2-
3	<50	nordca	Localized/ local spread	HR+/HER2+
3	<50	Sud	Localized/ local spread	TNBC
3	<50	NordAT	Localized/ local spread	HR+/HER2-
3	<50	Sud	Localized/ local spread	HR+/HER2-
3	[50-74]	Centre	Localized/ local spread	TNBC
2	<50	Centre	Localized/ local spread	HR+/HER2-
1	<50	Sud	Localized/ local spread	HR+/HER2-
3	<50	Centre	Localized/ local spread	HR+/HER2-
3	<50	Sud	Localized/ local spread	HR+/HER2-
3	<50	Centre	Localized/ local spread	HR+/HER2+
2	<50	Sud	Localized/ local spread	HR+/HER2+
3	<50	Centre	Localized/ local spread	TNBC
3	<50	Centre	Localized/ local spread	HR+/HER2+
2	<50	Centre	Localized/ local spread	HR+/HER2-
2	<50	Sud	Localized/ local spread	HR+/HER2+
3	<50	Sud	Localized/ local spread	HR+/HER2-
1	<50	NordAT	Localized/ local spread	HR+/HER2-
2	<50	Centre	Localized/ local spread	HR+/HER2-
3	<50	Centre	Localized/ local spread	HR+/HER2-
2	<50	nordca	Localized/ local spread	HR+/HER2-
3	<50	Centre	Localized/ local spread	HR+/HER2-
2	[50-74]	Sud	Localized/ local spread	HR+/HER2-
3	<50	Centre	Localized/ local spread	HR+/HER2-

2	<50	nordca	Localized/ local spread	HR+/HER2-
3	[50-74]	Centre	Localized/ local spread	HR+/HER2-
3	<50	Centre	Localized/ local spread	HR+/HER2-
3	<50	NordAT	Localized/ local spread	HR+/HER2-
3	[50-74]	Centre	Localized/ local spread	HR+/HER2+
3	[50-74]	Centre	Regional	TNBC
3	[50-74]	Sud	Localized/ local spread	HR+/HER2-
3	<50	NordAT	Localized/ local spread	HR+/HER2-
3	<50	Centre	Localized/ local spread	TNBC
2	<50	Sud	Localized/ local spread	HR+/HER2+
3	[50-74]	Sud	Localized/ local spread	HR+/HER2-
3	[50-74]	NordAT	Localized/ local spread	HR+/HER2-
3	<50	nordca	Localized/ local spread	HR+/HER2+
3	<50	Sud	Localized/ local spread	HR+/HER2-
1	[50-74]	Centre	Localized/ local spread	HR+/HER2+
3	<50	nordca	Localized/ local spread	HR-/HER2+
3	<50	Centre	Localized/ local spread	HR+/HER2-
2	<50	Sud	Localized/ local spread	HR+/HER2-
2	<50	Sud	Localized/ local spread	HR+/HER2-
3	[50-74]	Centre	Localized/ local spread	HR+/HER2-
3	[50-74]	Centre	Localized/ local spread	HR+/HER2-
1	<50	Sud	Localized/ local spread	TNBC
1	<50	Centre	Localized/ local spread	HR+/HER2-
3	<50	Sud	Localized/ local spread	HR+/HER2-
3	[50-74]	Sud	Localized/ local spread	HR+/HER2-
3	<50	Centre	Localized/ local spread	HR+/HER2-
3	<50	nordca	Localized/ local spread	HR+/HER2-
3	<50	Centre	Localized/ local spread	HR+/HER2-
2	[50-74]	Centre	Localized/ local spread	HR+/HER2-
3	<50	Centre	Localized/ local spread	HR+/HER2-
3	[50-74]	Sud	Localized/ local spread	HR+/HER2+
3	<50	Centre	Localized/ local spread	HR+/HER2-
3	[50-74]	nordca	Regional	HR+/HER2-
3	[50-74]	NordAT	Localized/ local spread	HR+/HER2-
3	<50	NordAT	Localized/ local spread	HR+/HER2-
2	[50-74]	Sud	Localized/ local spread	HR+/HER2-
3	<50	Sud	Localized/ local spread	TNBC
2	<50	Sud	Localized/ local spread	HR+/HER2-
3	<50	Centre	Localized/ local spread	HR+/HER2-
3	[50-74]	NordAT	Localized/ local spread	HR+/HER2-
3	<50	Centre	Localized/ local spread	HR-/HER2+
2	[50-74]	NordAT	Localized/ local spread	HR+/HER2-
3	<50	NordAT	Localized/ local spread	HR+/HER2-
3	<50	Sud	Localized/ local spread	HR+/HER2+
2	<50	Sud	Localized/ local spread	HR+/HER2-
3	[50-74]	Sud	Localized/ local spread	
3	[50-74]	NordAT	Localized/ local spread	TNBC
3	[50-74]	Centre	Localized/ local spread	HR+/HER2-
3	<50	NordAT	Localized/ local spread	TNBC
3	[50-74]	Sud	Localized/ local spread	HR+/HER2-

3	[50-74]	Sud	Localized/ local spread	HR+/HER2-
3	[50-74]	nordca	Localized/ local spread	TNBC
2	[50-74]	Centre	Localized/ local spread	HR+/HER2-
3	[50-74]	Sud	Localized/ local spread	HR+/HER2-
1	<50	Centre	Localized/ local spread	HR+/HER2-
3	<50	NordAT	Localized/ local spread	TNBC
3	<50	Sud	Localized/ local spread	HR+/HER2-
1	<50	Sud	Localized/ local spread	HR+/HER2-
2	<50	NordAT	Localized/ local spread	HR+/HER2-
3	[50-74]	Centre	Localized/ local spread	HR+/HER2-
3	<50	NordAT	Localized/ local spread	TNBC
3	<50	Centre	Localized/ local spread	HR+/HER2-
3	<50	Sud	Localized/ local spread	HR+/HER2-
2	[50-74]	Centre	Localized/ local spread	HR+/HER2-
3	[50-74]	Sud	Localized/ local spread	HR+/HER2-
1	<50	Centre	Localized/ local spread	HR+/HER2-
2	<50	Centre	Localized/ local spread	HR+/HER2+
3	[50-74]	Centre	Localized/ local spread	HR+/HER2-
2	<50	Centre	Localized/ local spread	HR+/HER2-
3	<50	NordAT	Localized/ local spread	HR+/HER2-
3	<50	Sud	Localized/ local spread	HR-/HER2+
2	[50-74]	Sud	Localized/ local spread	HR+/HER2-
3	<50	Sud	Localized/ local spread	HR+/HER2-
3	<50	NordAT	Localized/ local spread	HR+/HER2-
3	<50	Sud	Localized/ local spread	HR+/HER2-
2	<50	NordAT	Localized/ local spread	HR+/HER2-
3	<50	Sud	Localized/ local spread	HR+/HER2-
2	[50-74]	Centre	Localized/ local spread	HR+/HER2+
2	<50	Sud	Localized/ local spread	HR+/HER2-
3	<50	Sud	Localized/ local spread	HR+/HER2-
3	[50-74]	NordAT	Localized/ local spread	HR+/HER2+
3	<50	NordAT	Localized/ local spread	HR+/HER2-
3	<50	Sud	Localized/ local spread	HR+/HER2-
3	<50	NordAT	Localized/ local spread	HR+/HER2-
3	<50	Centre	Localized/ local spread	HR+/HER2+
3	[50-74]	Centre	Localized/ local spread	TNBC
3	<50	Centre	Localized/ local spread	HR-/HER2+
3	<50	Centre	Localized/ local spread	HR+/HER2-
3	[50-74]	Centre	Localized/ local spread	TNBC
3	<50	Centre	Localized/ local spread	TNBC
3	[50-74]	Sud	Localized/ local spread	TNBC
3	<50	Sud	Localized/ local spread	HR-/HER2+
9	<50	Sud	Localized/ local spread	
2	<50	Centre	Localized/ local spread	HR+/HER2+
3	[50-74]	Centre	Localized/ local spread	TNBC
3	[50-74]	NordAT	Localized/ local spread	HR+/HER2-
2	[50-74]	Sud	Localized/ local spread	TNBC
2	[50-74]	Sud	Localized/ local spread	HR+/HER2-
2	<50	Sud	Localized/ local spread	HR+/HER2+
2	<50	Centre	Localized/ local spread	HR-/HER2+

3	<50	NordAT	Localized/ local spread	HR+/HER2-
3	[50-74]	NordAT	Localized/ local spread	HR+/HER2-
3	<50	NordAT	Localized/ local spread	HR-/HER2+
2	<50	NordAT	Localized/ local spread	HR+/HER2-
2	<50	NordAT	Localized/ local spread	HR+/HER2-
3	<50	Centre	Localized/ local spread	TNBC
3	<50	Centre	Localized/ local spread	HR+/HER2-
3	<50	Sud	Localized/ local spread	HR+/HER2-
3	<50	Centre	Localized/ local spread	HR+/HER2-
3	<50	Centre	Localized/ local spread	HR+/HER2-
3	[50-74]	Sud	Localized/ local spread	HR+/HER2-
3	<50	NordAT	Localized/ local spread	TNBC
3	<50	NordAT	Regional	TNBC
3	[50-74]	nordca	Localized/ local spread	HR+/HER2-
2	[50-74]	Sud	Localized/ local spread	HR+/HER2-
1	<50	Sud	Localized/ local spread	HR+/HER2-
2	<50	Centre	Localized/ local spread	HR+/HER2-
3	<50	NordAT	Localized/ local spread	HR+/HER2-
3	[50-74]	NordAT	Localized/ local spread	HR+/HER2-
3	[50-74]	Sud	Localized/ local spread	TNBC
3	<50	Sud	Localized/ local spread	HR+/HER2-
3	[50-74]	Centre	Localized/ local spread	HR+/HER2+
3	[50-74]	Centre	Localized/ local spread	HR+/HER2-
3	[50-74]	Centre	Localized/ local spread	HR+/HER2-
3	<50	Sud	Localized/ local spread	HR-/HER2+
3	[50-74]	Sud	Localized/ local spread	HR+/HER2-
3	[50-74]	Sud	Localized/ local spread	HR+/HER2-
3	<50	nordca	Localized/ local spread	HR+/HER2-
3	[50-74]	Centre	Localized/ local spread	HR+/HER2+
2	<50	Sud	Localized/ local spread	HR+/HER2-
3	<50	Sud	Localized/ local spread	HR+/HER2-
3	<50	Sud	Localized/ local spread	HR-/HER2+
3	[50-74]	Centre	Localized/ local spread	HR+/HER2-
2	[50-74]	Centre	Localized/ local spread	HR-/HER2+
3	[50-74]	Sud	Localized/ local spread	HR+/HER2-
2	<50	NordAT	Localized/ local spread	HR+/HER2-
2	<50	Centre	Localized/ local spread	HR+/HER2-
3	<50	Centre	Localized/ local spread	TNBC
3	<50	NordAT	Localized/ local spread	HR+/HER2-
3	<50	Centre	Localized/ local spread	HR+/HER2-
1	<50	Centre	Localized/ local spread	HR+/HER2-
1	<50	NordAT	Localized/ local spread	HR+/HER2-
3	<50	Centre	Localized/ local spread	HR+/HER2-
1	[50-74]	NordAT	Localized/ local spread	HR+/HER2-
3	<50	Sud	Localized/ local spread	HR+/HER2-
2	<50	Centre	Localized/ local spread	HR+/HER2-
3	<50	Centre	Localized/ local spread	HR-/HER2+
3	[50-74]	Centre	Localized/ local spread	HR+/HER2-
3	<50	Centre	Localized/ local spread	HR+/HER2-
3	[50-74]	Sud	Localized/ local spread	HR+/HER2-

2	<50	NordAT	Localized/ local spread	HR+/HER2-
9	<50	Sud	Localized/ local spread	TNBC
3	<50	Sud	Localized/ local spread	HR-/HER2+
3	[50-74]	Centre	Localized/ local spread	
3	<50	NordAT	Localized/ local spread	HR+/HER2-
3	<50	Centre	Localized/ local spread	HR+/HER2-
2	<50	Centre	Localized/ local spread	HR+/HER2-
	<50	Centre	Localized/ local spread	TNBC
3	<50	Sud	Localized/ local spread	HR+/HER2-
3	<50	Centre	Localized/ local spread	HR+/HER2-
2	<50	Sud	Localized/ local spread	TNBC
3	<50	NordAT	Localized/ local spread	HR+/HER2-
9	<50	NordAT	Localized/ local spread	HR-/HER2+
3	<50	Centre	Regional	HR+/HER2-
3	[50-74]	Centre	Localized/ local spread	HR+/HER2-
3	<50	NordAT	Localized/ local spread	HR+/HER2-
	<50	Sud	Localized/ local spread	HR+/HER2-
2	<50	Centre	Localized/ local spread	HR+/HER2-
2	<50	NordAT	Localized/ local spread	HR+/HER2-
3	<50	Centre	Localized/ local spread	TNBC
3	<50	Sud	Localized/ local spread	TNBC
3	<50	Centre	Localized/ local spread	TNBC
3	<50	Centre	Localized/ local spread	TNBC
3	<50	nordca	Localized/ local spread	TNBC
3	[50-74]	Centre	Localized/ local spread	HR+/HER2-
2	[50-74]	Centre	Localized/ local spread	TNBC
3	[50-74]	Centre	Localized/ local spread	HR+/HER2-
3	<50	NordAT	Localized/ local spread	HR+/HER2-
2	<50	NordAT	Localized/ local spread	HR+/HER2-
3	<50	Centre	Localized/ local spread	HR+/HER2-
3	>=75	Centre	Localized/ local spread	HR+/HER2-
3	>=75	nordca	Localized/ local spread	HR+/HER2-
2	>=75	Centre	Localized/ local spread	HR+/HER2-
2	>=75	Centre	Localized/ local spread	
2	>=75	Centre	Localized/ local spread	HR+/HER2-
3	>=75	NordAT	Localized/ local spread	TNBC
3	>=75	Sud	Localized/ local spread	HR-/HER2+
3	>=75	NordAT	Regional	
3	>=75	Sud	Localized/ local spread	HR+/HER2-
1	>=75	Sud	Localized/ local spread	HR+/HER2-
3	>=75	NordAT	Localized/ local spread	
3	>=75	Centre	Localized/ local spread	HR+/HER2-
3	>=75	NordAT	Localized/ local spread	TNBC
2	>=75	Centre	Localized/ local spread	
3	>=75	Centre	Localized/ local spread	TNBC
3	>=75	Centre	Regional	HR+/HER2+
3	>=75	Centre	Localized/ local spread	HR+/HER2-
3	>=75	Sud	Localized/ local spread	
3	>=75	Centre	Localized/ local spread	HR+/HER2+
3	>=75	Centre	Localized/ local spread	TNBC

3	>=75	NordAT	Localized/ local spread	HR-/HER2+
	>=75	Centre	Localized/ local spread	HR+/HER2-
3	>=75	Centre	Localized/ local spread	HR+/HER2-
3	>=75	Centre	Localized/ local spread	HR+/HER2-
3	>=75	Centre	Localized/ local spread	HR+/HER2-
3	>=75	Sud	Localized/ local spread	HR+/HER2-
2	>=75	Centre	Localized/ local spread	
3	>=75	Sud	Localized/ local spread	HR+/HER2-
3	>=75	NordAT	Localized/ local spread	HR+/HER2-
3	>=75	Centre	Regional	HR+/HER2-
3	>=75	nordca	Localized/ local spread	HR+/HER2+
3	>=75	Centre	Localized/ local spread	HR-/HER2+
1	>=75	Sud	Localized/ local spread	HR+/HER2+
3	>=75	Sud	Localized/ local spread	
3	>=75	Centre	Localized/ local spread	TNBC
3	>=75	Sud	Localized/ local spread	HR+/HER2-
10	>=75	Sud	Localized/ local spread	
3	>=75	NordAT	Localized/ local spread	HR+/HER2-
3	>=75	Centre	Localized/ local spread	HR-/HER2+
3	>=75	Centre	Localized/ local spread	
3	>=75	NordAT	Localized/ local spread	TNBC
3	>=75	NordAT	Localized/ local spread	
2	>=75	Sud	Localized/ local spread	HR+/HER2-
3	>=75	Centre	Localized/ local spread	
3	>=75	Sud	Localized/ local spread	HR-/HER2+
2	>=75	NordAT	Localized/ local spread	
3	>=75	Centre	Localized/ local spread	HR+/HER2-
3	>=75	NordAT	Localized/ local spread	HR+/HER2-
3	>=75	Sud	Localized/ local spread	HR+/HER2-
	>=75	Sud	Localized/ local spread	HR+/HER2+
2	>=75	Centre	Localized/ local spread	
2	>=75	Sud	Localized/ local spread	HR+/HER2-
	>=75	Centre	Localized/ local spread	TNBC
3	>=75	Sud	Regional	HR+/HER2-
3	>=75	Centre	Localized/ local spread	HR+/HER2+
3	>=75	Sud	Localized/ local spread	
3	>=75	Centre	Localized/ local spread	
2	>=75	Centre	Localized/ local spread	HR-/HER2+
3	>=75	Centre	Localized/ local spread	TNBC
3	>=75	Sud	Localized/ local spread	HR+/HER2-
3	>=75	Sud	Localized/ local spread	TNBC
3	>=75	Centre	Localized/ local spread	TNBC
3	>=75	NordAT	Localized/ local spread	HR+/HER2+
3	>=75	Sud	Localized/ local spread	HR+/HER2-
3	>=75	Centre	Localized/ local spread	
3	>=75	Centre	Localized/ local spread	
3	>=75	Sud	Localized/ local spread	HR+/HER2-
3	>=75	NordAT	Localized/ local spread	HR+/HER2-
3	>=75	Sud	Localized/ local spread	
2	>=75	NordAT	Localized/ local spread	

3	>=75	Centre	Localized/ local spread	HR+/HER2-
3	>=75	Sud	Localized/ local spread	HR+/HER2-
3	>=75	Sud	Localized/ local spread	HR+/HER2+
3	>=75	Sud	Localized/ local spread	HR-/HER2+
3	>=75	Centre	Localized/ local spread	HR+/HER2-
2	>=75	Sud	Localized/ local spread	HR+/HER2-
3	>=75	Sud	Localized/ local spread	HR+/HER2-
3	>=75	Centre	Localized/ local spread	HR+/HER2+
3	>=75	Sud	Localized/ local spread	HR+/HER2+
2	>=75	Centre	Localized/ local spread	TNBC
2	>=75	Centre	Localized/ local spread	HR+/HER2-
3	>=75	Centre	Localized/ local spread	TNBC
3	>=75	Sud	Localized/ local spread	HR+/HER2-
3	>=75	Centre	Localized/ local spread	HR+/HER2-
2	>=75	Centre	Localized/ local spread	HR+/HER2-
2	>=75	Sud	Localized/ local spread	HR+/HER2-
2	>=75	Centre	Localized/ local spread	HR+/HER2-
3	>=75	Centre	Localized/ local spread	TNBC
3	>=75	NordAT	Localized/ local spread	TNBC
2	>=75	Centre	Localized/ local spread	HR+/HER2-
3	>=75	Centre	Regional	TNBC
3	>=75	nordca	Localized/ local spread	HR+/HER2+
3	>=75	Centre	Localized/ local spread	HR+/HER2-
2	>=75	Sud	Localized/ local spread	HR+/HER2+
2	>=75	NordAT	Localized/ local spread	HR-/HER2+
3	>=75	Centre	Localized/ local spread	TNBC
3	>=75	Sud	Localized/ local spread	
3	>=75	Centre	Localized/ local spread	HR+/HER2-
3	>=75	nordca	Localized/ local spread	
3	>=75	Sud	Localized/ local spread	HR+/HER2-
3	>=75	Sud	Localized/ local spread	HR-/HER2+
3	>=75	Centre	Localized/ local spread	HR+/HER2+
3	>=75	Centre	Regional	
3	>=75	Sud	Localized/ local spread	HR+/HER2+
3	>=75	Centre	Localized/ local spread	TNBC
3	>=75	Centre	Localized/ local spread	HR+/HER2-
3	>=75	Centre	Localized/ local spread	HR+/HER2-
3	>=75	Sud	Localized/ local spread	HR+/HER2-
3	>=75	Sud	Localized/ local spread	HR+/HER2-
3	>=75	NordAT	Localized/ local spread	HR+/HER2-
3	>=75	Sud	Localized/ local spread	HR+/HER2-
3	>=75	Centre	Localized/ local spread	HR+/HER2-
3	>=75	NordAT	Localized/ local spread	HR+/HER2-
3	>=75	Centre	Localized/ local spread	HR+/HER2+
3	>=75	NordAT	Localized/ local spread	TNBC
9	>=75	Centre	Localized/ local spread	
3	>=75	Centre	Localized/ local spread	TNBC
2	>=75	Centre	Localized/ local spread	HR+/HER2-
3	>=75	Centre	Localized/ local spread	HR+/HER2-
3	>=75	Centre	Localized/ local spread	HR+/HER2-

3	>=75	Centre	Localized/ local spread	HR+/HER2+
3	>=75	Sud	Localized/ local spread	HR+/HER2-
3	>=75	Centre	Localized/ local spread	HR+/HER2-
3	>=75	Centre	Localized/ local spread	
3	>=75	nordca	Localized/ local spread	TNBC
3	>=75	NordAT	Localized/ local spread	HR+/HER2-
3	>=75	Centre	Localized/ local spread	TNBC
3	>=75	NordAT	Localized/ local spread	TNBC
3	>=75	nordca	Localized/ local spread	HR+/HER2-
3	>=75	NordAT	Localized/ local spread	
3	>=75	Sud	Localized/ local spread	
3	>=75	NordAT	Localized/ local spread	
3	>=75	Centre	Localized/ local spread	HR+/HER2-
2	>=75	NordAT	Localized/ local spread	TNBC
3	>=75	Centre	Localized/ local spread	
3	>=75	NordAT	Localized/ local spread	HR-/HER2+
3	>=75	Centre	Localized/ local spread	TNBC
3	>=75	Sud	Localized/ local spread	HR+/HER2-
3	>=75	Sud	Localized/ local spread	HR+/HER2-
9	>=75	Centre	Localized/ local spread	HR+/HER2-
4	>=75	Centre	Localized/ local spread	HR+/HER2-
3	<50	Centre	Metastatic	HR+/HER2+
3	<50	Centre	Metastatic	TNBC
3	<50	Sud	Metastatic	HR-/HER2+
3	<50	Sud	Metastatic	HR+/HER2-
3	<50	Sud	Metastatic	HR+/HER2+
3	<50	NordAT	Metastatic	TNBC
3	<50	Centre	Metastatic	HR+/HER2-
3	<50	Centre	Metastatic	HR+/HER2+
3	<50	Sud	Metastatic	TNBC
3	<50	Sud	Metastatic	HR+/HER2+
3	<50	Centre	Metastatic	HR+/HER2+
3	<50	Centre	Metastatic	HR+/HER2-
3	<50	Centre	Metastatic	HR+/HER2-
9	<50	Centre	Metastatic	HR-/HER2+
3	<50	Sud	Metastatic	HR-/HER2+
2	<50	Centre	Metastatic	HR+/HER2-
3	<50	Sud	Metastatic	
9	<50	NordAT	Metastatic	HR+/HER2-
3	<50	Sud	Metastatic	TNBC
3	<50	Centre	Metastatic	TNBC
3	<50	Centre	Metastatic	HR-/HER2+
3	<50	Sud	Metastatic	HR+/HER2-
3	<50	Sud	Metastatic	HR+/HER2-
3	<50	Centre	Metastatic	TNBC
3	<50	NordAT	Metastatic	HR+/HER2-
3	<50	Centre	Metastatic	TNBC
3	<50	Centre	Metastatic	HR+/HER2-
2	<50	Sud	Metastatic	TNBC
2	<50	Centre	Metastatic	HR+/HER2+

9	<50	Centre	Metastatic	TNBC
2	<50	nordca	Metastatic	TNBC
3	<50	Centre	Metastatic	HR+/HER2-
3	<50	Centre	Metastatic	TNBC
3	<50	Sud	Metastatic	HR+/HER2+
3	<50	Centre	Metastatic	HR+/HER2+
2	<50	Sud	Metastatic	HR+/HER2-
2	<50	NordAT	Metastatic	HR+/HER2+
3	<50	NordAT	Metastatic	TNBC
3	<50	Sud	Metastatic	TNBC
3	<50	NordAT	Metastatic	HR-/HER2+
3	<50	NordAT	Metastatic	HR-/HER2+
3	<50	nordca	Metastatic	HR+/HER2-
3	<50	Centre	Metastatic	HR+/HER2+
3	<50	nordca	Metastatic	HR+/HER2-
3	<50	NordAT	Metastatic	HR+/HER2-
3	<50	Sud	Metastatic	HR+/HER2-
3	<50	NordAT	Metastatic	HR+/HER2-
3	<50	NordAT	Metastatic	HR+/HER2-
3	<50	NordAT	Metastatic	HR+/HER2-
3	<50	Centre	Metastatic	TNBC
1	<50	Centre	Metastatic	TNBC
3	<50	Sud	Metastatic	HR+/HER2-
3	<50	Sud	Metastatic	HR-/HER2+
3	<50	Sud	Metastatic	HR-/HER2+
3	<50	nordca	Metastatic	HR+/HER2+
3	<50	Sud	Metastatic	HR+/HER2-
3	<50	Centre	Metastatic	HR+/HER2-
3	<50	Centre	Metastatic	HR+/HER2-
3	<50	Centre	Metastatic	HR+/HER2+
3	<50	Centre	Metastatic	HR+/HER2-
3	<50	Centre	Metastatic	TNBC
2	<50	Centre	Metastatic	HR+/HER2+
3	<50	Centre	Metastatic	HR+/HER2-
3	<50	Sud	Metastatic	HR-/HER2+
3	<50	Centre	Metastatic	HR-/HER2+
3	<50	Sud	Metastatic	TNBC
3	<50	Centre	Metastatic	HR+/HER2+
2	<50	Centre	Metastatic	HR+/HER2-
3	<50	Sud	Metastatic	HR+/HER2-
3	<50	Sud	Metastatic	HR+/HER2+
3	<50	Sud	Metastatic	HR+/HER2-
3	<50	Sud	Metastatic	HR+/HER2-
3	<50	Sud	Metastatic	TNBC
3	<50	Sud	Metastatic	HR+/HER2+
9	<50	NordAT	Metastatic	TNBC
3	<50	Centre	Metastatic	TNBC
3	<50	Centre	Metastatic	HR+/HER2+
3	<50	Centre	Metastatic	HR+/HER2+
3	<50	Centre	Metastatic	TNBC

3	<50	Sud	Metastatic	HR+/HER2-
2	<50	nordca	Metastatic	HR+/HER2-
2	<50	NordAT	Metastatic	HR+/HER2-
3	<50	Centre	Metastatic	TNBC
3	<50	Sud	Metastatic	HR+/HER2-
3	<50	Centre	Metastatic	TNBC
3	<50	Sud	Metastatic	TNBC
3	<50	Centre	Metastatic	HR+/HER2-
3	<50	Sud	Metastatic	HR-/HER2+
2	<50	Centre	Metastatic	TNBC
3	<50	NordAT	Metastatic	HR+/HER2-
3	[50-74]	nordca	Metastatic	HR+/HER2-
2	<50	NordAT	Metastatic	HR+/HER2-
3	<50	Centre	Metastatic	HR+/HER2-
3	<50	NordAT	Metastatic	TNBC
	<50	Centre	Metastatic	TNBC
3	<50	Centre	Metastatic	HR+/HER2-
3	<50	Sud	Metastatic	TNBC
2	<50	Sud	Metastatic	HR+/HER2-
2	<50	Centre	Metastatic	TNBC
3	<50	Sud	Metastatic	TNBC
3	<50	NordAT	Metastatic	HR+/HER2-
3	<50	Sud	Metastatic	HR+/HER2+
3	<50	Sud	Metastatic	TNBC
	<50	Sud	Metastatic	TNBC
3	<50	Sud	Metastatic	HR+/HER2-
3	[50-74]	Centre	Metastatic	HR+/HER2-
3	<50	NordAT	Metastatic	HR+/HER2-
3	<50	Centre	Metastatic	HR+/HER2-
3	<50	NordAT	Metastatic	HR+/HER2+
2	[50-74]	Centre	Metastatic	HR+/HER2-
3	<50	nordca	Metastatic	TNBC
	[50-74]	Sud	Metastatic	TNBC
3	<50	Centre	Metastatic	TNBC
9	[50-74]	NordAT	Metastatic	HR+/HER2-
3	<50	Sud	Metastatic	HR+/HER2+
2	<50	Centre	Metastatic	TNBC
3	<50	Sud	Metastatic	HR+/HER2+
3	<50	Centre	Metastatic	HR+/HER2+
9	[50-74]	Centre	Metastatic	TNBC
3	<50	nordca	Metastatic	HR-/HER2+
3	<50	Centre	Metastatic	HR+/HER2-
3	<50	NordAT	Metastatic	TNBC
3	<50	Sud	Metastatic	TNBC
3	[50-74]	NordAT	Metastatic	HR+/HER2+
3	<50	Sud	Metastatic	HR+/HER2+
3	[50-74]	Sud	Metastatic	HR+/HER2-
	<50	Centre	Metastatic	HR+/HER2-
3	[50-74]	Centre	Metastatic	HR+/HER2-
3	<50	Centre	Metastatic	TNBC

3	<50	Centre	Metastatic	TNBC
1	<50	Sud	Metastatic	HR+/HER2-
3	<50	Sud	Metastatic	HR+/HER2+
3	[50-74]	Centre	Metastatic	TNBC
2	<50	Sud	Metastatic	HR+/HER2-
3	<50	Sud	Metastatic	TNBC
3	<50	Centre	Metastatic	HR+/HER2-
3	<50	Sud	Metastatic	HR+/HER2+
3	<50	Sud	Metastatic	HR+/HER2-
2	<50	Centre	Metastatic	HR+/HER2-
3	<50	Sud	Metastatic	HR+/HER2-
3	<50	Sud	Metastatic	HR+/HER2-
3	<50	Sud	Metastatic	TNBC
3	<50	Centre	Metastatic	HR+/HER2+
3	<50	Centre	Metastatic	TNBC
3	<50	Sud	Metastatic	TNBC
3	<50	Sud	Metastatic	HR+/HER2-
3	<50	Centre	Metastatic	TNBC
3	<50	Centre	Metastatic	
3	[50-74]	Centre	Metastatic	TNBC
3	[50-74]	NordAT	Metastatic	HR-/HER2+
3	[50-74]	Sud	Metastatic	HR-/HER2+
2	<50	NordAT	Metastatic	HR+/HER2-
3	<50	Centre	Metastatic	TNBC
3	<50	Centre	Metastatic	TNBC
2	<50	Centre	Metastatic	HR+/HER2+
3	<50	Centre	Metastatic	HR+/HER2-
3	[50-74]	Sud	Metastatic	HR+/HER2-
3	[50-74]	NordAT	Metastatic	HR+/HER2-
2	<50	Centre	Metastatic	HR+/HER2-
3	<50	Sud	Metastatic	HR+/HER2-
9	<50	Centre	Metastatic	HR-/HER2+
3	[50-74]	Sud	Metastatic	HR+/HER2+
2	[50-74]	Sud	Metastatic	
3	[50-74]	Sud	Metastatic	HR+/HER2+
3	<50	NordAT	Metastatic	HR+/HER2+
9	<50	NordAT	Metastatic	HR-/HER2+
3	<50	Sud	Metastatic	TNBC
3	<50	NordAT	Metastatic	HR+/HER2-
3	<50	Centre	Metastatic	HR+/HER2-
2	<50	Sud	Metastatic	HR+/HER2-
3	<50	Centre	Metastatic	HR-/HER2+
3	[50-74]	Centre	Metastatic	HR+/HER2-
3	[50-74]	Centre	Metastatic	HR+/HER2-
3	<50	Sud	Metastatic	HR+/HER2-
3	<50	Centre	Metastatic	HR-/HER2+
2	[50-74]	Centre	Metastatic	TNBC
3	<50	NordAT	Metastatic	HR+/HER2-
3	<50	NordAT	Metastatic	HR+/HER2-
3	<50	nordca	Metastatic	HR+/HER2-

3	<50	Sud	Metastatic	HR+/HER2+
3	[50-74]	Centre	Metastatic	HR+/HER2-
3	[50-74]	NordAT	Metastatic	TNBC
3	[50-74]	Centre	Metastatic	HR+/HER2+
3	<50	Centre	Metastatic	HR+/HER2+
3	<50	Centre	Metastatic	TNBC
3	<50	NordAT	Metastatic	TNBC
3	<50	NordAT	Metastatic	TNBC
3	<50	Centre	Metastatic	HR+/HER2+
3	<50	Sud	Metastatic	HR+/HER2-
3	[50-74]	Sud	Metastatic	TNBC
3	<50	NordAT	Metastatic	TNBC
3	<50	Centre	Metastatic	HR+/HER2-
9	<50	Centre	Metastatic	HR-/HER2+
3	<50	NordAT	Metastatic	HR+/HER2-
3	[50-74]	Centre	Metastatic	TNBC
2	<50	NordAT	Metastatic	HR+/HER2-
3	<50	Sud	Metastatic	HR-/HER2+
3	[50-74]	Sud	Metastatic	HR-/HER2+
3	<50	Centre	Metastatic	HR+/HER2-
3	<50	NordAT	Metastatic	TNBC
3	<50	Centre	Metastatic	HR+/HER2-
3	<50	Centre	Metastatic	HR+/HER2-
3	[50-74]	Centre	Metastatic	TNBC
2	<50	Centre	Metastatic	HR+/HER2+
3	[50-74]	Centre	Metastatic	TNBC
3	[50-74]	Sud	Metastatic	HR+/HER2+
3	[50-74]	Centre	Metastatic	HR-/HER2+
2	<50	Sud	Metastatic	HR+/HER2-
3	<50	Sud	Metastatic	HR+/HER2-
3	<50	Centre	Metastatic	HR+/HER2-
3	<50	NordAT	Metastatic	TNBC
3	<50	nordca	Metastatic	TNBC
3	<50	NordAT	Metastatic	HR-/HER2+
9	<50	Sud	Metastatic	HR-/HER2+
3	[50-74]	Sud	Metastatic	TNBC
2	<50	Sud	Metastatic	HR+/HER2+
3	<50	Sud	Metastatic	TNBC
3	[50-74]	NordAT	Metastatic	TNBC
3	<50	Centre	Metastatic	TNBC
3	<50	NordAT	Metastatic	HR+/HER2-
9	[50-74]	Sud	Metastatic	HR-/HER2+
2	[50-74]	NordAT	Metastatic	HR-/HER2+
9	<50	Sud	Metastatic	TNBC
10	<50	Sud	Metastatic	HR+/HER2-
2	<50	Centre	Metastatic	HR+/HER2-
3	[50-74]	Centre	Metastatic	TNBC
3	<50	Centre	Metastatic	TNBC
2	<50	Centre	Metastatic	TNBC
3	[50-74]	Centre	Metastatic	HR+/HER2+

3	[50-74]	Centre	Metastatic	HR+/HER2-
2	<50	Sud	Metastatic	TNBC
3	[50-74]	Centre	Metastatic	HR+/HER2-
2	[50-74]	Centre	Metastatic	HR+/HER2-
3	[50-74]	Centre	Metastatic	
	<50	Centre	Metastatic	HR-/HER2+
3	<50	Centre	Metastatic	HR+/HER2+
3	>=75	Centre	Metastatic	
3	>=75	Centre	Metastatic	
3	>=75	Sud	Metastatic	HR+/HER2-
3	>=75	Centre	Metastatic	HR+/HER2-
	>=75	Centre	Metastatic	TNBC
3	>=75	Centre	Metastatic	HR+/HER2-
3	>=75	NordAT	Metastatic	TNBC
3	>=75	nordca	Metastatic	HR+/HER2-
9	>=75	Centre	Metastatic	
9	>=75	Centre	Metastatic	TNBC
3	>=75	Centre	Metastatic	
3	>=75	Sud	Metastatic	TNBC
2	>=75	Centre	Metastatic	HR+/HER2-
3	>=75	Sud	Metastatic	HR+/HER2+
3	>=75	Sud	Metastatic	HR+/HER2+
2	>=75	Sud	Metastatic	HR+/HER2-
2	>=75	nordca	Metastatic	HR+/HER2-
3	>=75	Centre	Metastatic	HR+/HER2-
2	>=75	Centre	Metastatic	HR+/HER2-
3	>=75	NordAT	Metastatic	HR-/HER2+
3	>=75	NordAT	Metastatic	HR+/HER2-
3	>=75	Centre	Metastatic	TNBC
3	>=75	Centre	Metastatic	HR-/HER2+
3	>=75	Sud	Metastatic	HR+/HER2-
3	>=75	Sud	Metastatic	HR+/HER2-
3	>=75	Centre	Metastatic	HR+/HER2-
3	>=75	Sud	Metastatic	HR+/HER2-
3	>=75	Sud	Metastatic	HR+/HER2-
3	>=75	NordAT	Metastatic	
3	>=75	Centre	Metastatic	HR+/HER2-
1	>=75	Centre	Metastatic	HR+/HER2-
3	>=75	Centre	Metastatic	TNBC
2	>=75	Centre	Metastatic	HR+/HER2-
3	>=75	Centre	Metastatic	HR+/HER2-
2	>=75	NordAT	Metastatic	HR+/HER2-
3	>=75	NordAT	Metastatic	TNBC
3	>=75	Sud	Metastatic	HR+/HER2-
2	>=75	Centre	Metastatic	TNBC
3	<50	Sud	4	HR+/HER2-
3	<50	Centre	4	HR+/HER2-
2	<50	Sud	4	HR+/HER2-
3	<50	NordAT	4	HR+/HER2-
2	<50	nordca	4	HR+/HER2-

2	<50	Centre	4	HR-/HER2+
2	<50	Centre	4	HR+/HER2+
3	<50	Centre	4	HR+/HER2-
3	<50	Centre	4	HR+/HER2-
3	<50	Sud	4	HR-/HER2+
3	<50	Centre	4	TNBC
3	<50	Sud	4	HR+/HER2+
3	<50	NordAT	4	TNBC
3	<50	Sud	4	HR+/HER2-
2	<50	Sud	4	HR+/HER2-
3	<50	Centre	4	HR+/HER2-
3	<50	Centre	4	TNBC
3	<50	Centre	4	HR-/HER2+
3	<50	NordAT	4	HR+/HER2-
3	<50	Centre	4	HR+/HER2-
3	<50	Sud	4	HR-/HER2+
9	<50	Centre	4	TNBC
3	<50	NordAT	4	HR+/HER2+
3	<50	NordAT	4	TNBC
3	<50	Centre	4	TNBC
3	<50	Centre	4	HR+/HER2-
3	<50	NordAT	4	HR-/HER2+
3	<50	Centre	4	HR+/HER2-
3	<50	Centre	4	HR+/HER2-
3	<50	Sud	4	HR+/HER2-
3	<50	Sud	4	HR+/HER2+
3	<50	Centre	4	HR+/HER2+
3	<50	Sud	4	HR-/HER2+
2	<50	Sud	4	HR+/HER2+
3	[50-74]	Centre	4	HR+/HER2-
1	[50-74]	Centre	4	
9	<50	Centre	4	
3	<50	nordca	4	
	[50-74]	Centre	4	HR+/HER2-
3	[50-74]	Centre	4	TNBC
9	<50	Centre	4	HR+/HER2-
3	[50-74]	Sud	4	HR+/HER2+
3	[50-74]	Sud	4	HR+/HER2-
3	<50	Sud	4	HR+/HER2+
3	<50	Sud	4	TNBC
2	<50	Sud	4	HR+/HER2-
3	<50	Sud	4	HR-/HER2+
3	<50	Sud	4	TNBC
3	<50	Centre	4	HR+/HER2-
3	<50	Sud	4	TNBC
2	<50	Sud	4	HR+/HER2-
3	[50-74]	Centre	4	HR+/HER2-
3	<50	Centre	4	HR+/HER2-
3	<50	Sud	4	HR+/HER2-
3	[50-74]	Sud	4	HR+/HER2-

3	<50	NordAT	4	HR+/HER2-
1	[50-74]	Centre	4	HR+/HER2-
3	<50	Sud	4	TNBC
9	<50	Centre	4	HR+/HER2-
2	<50	Centre	4	TNBC
3	[50-74]	Sud	4	HR+/HER2-
3	[50-74]	Sud	4	HR+/HER2+
3	[50-74]	Sud	4	HR+/HER2-
9	<50	Centre	4	HR+/HER2-
3	<50	NordAT	4	HR+/HER2-
3	[50-74]	Sud	4	
3	<50	Centre	4	HR-/HER2+
2	<50	Centre	4	HR+/HER2+
3	<50	NordAT	4	HR+/HER2+
2	<50	NordAT	4	HR+/HER2-
3	[50-74]	NordAT	4	HR+/HER2+
3	<50	Sud	4	HR-/HER2+
3	<50	nordca	4	HR+/HER2+
9	<50	Sud	4	HR+/HER2+
3	[50-74]	Centre	4	TNBC
2	[50-74]	Sud	4	HR+/HER2-
3	<50	NordAT	4	HR+/HER2+
3	<50	Centre	4	TNBC
3	<50	Centre	4	HR+/HER2-
3	[50-74]	Sud	4	HR+/HER2-
3	<50	Centre	4	HR-/HER2+
2	[50-74]	Centre	4	TNBC
3	<50	Centre	4	HR+/HER2-
3	[50-74]	Centre	4	HR+/HER2-
3	<50	Centre	4	HR+/HER2+
2	[50-74]	Sud	4	HR+/HER2-
3	<50	Sud	4	HR+/HER2+
3	<50	nordca	4	HR+/HER2+
3	[50-74]	Centre	4	HR+/HER2-
3	[50-74]	nordca	4	HR+/HER2+
3	[50-74]	NordAT	4	HR+/HER2-
3	<50	Centre	4	HR+/HER2-
3	<50	Sud	4	HR+/HER2-
3	<50	NordAT	4	HR+/HER2-
3	<50	Sud	4	HR-/HER2+
3	[50-74]	NordAT	4	HR+/HER2-
3	<50	Centre	4	HR+/HER2-
3	>=75	Sud	4	HR+/HER2-
3	>=75	NordAT	4	HR+/HER2-
3	>=75	Centre	4	HR+/HER2+
2	>=75	Centre	4	
3	>=75	Centre	4	TNBC
3	>=75	Sud	4	HR-/HER2+
3	>=75	NordAT	4	
3	>=75	Centre	4	HR+/HER2+

3	>=75	Centre	4	HR+/HER2-
3	>=75	Centre	4	HR+/HER2-
10	>=75	Centre	4	
3	>=75	Sud	4	HR+/HER2-
3	>=75	Centre	4	HR+/HER2-
3	>=75	nordca	4	HR-/HER2+
3	>=75	NordAT	4	
3	>=75	nordca	4	HR+/HER2-
9	>=75	Centre	4	HR+/HER2-
