# Supplementary material for: Overall survival of triple negative breast cancer in French Caribbean women
Source: PLoS One. 2022 Aug 24;17(8):e0271966. doi: 10.1371/journal.pone.0271966 (PMC9401158; doi:10.1371/journal.pone.0271966)
Supplement: S1 Data — (ZIP) [file pone.0271966.s001.zip › survie_global.pdf]

Time (month)	CENSOR	SURVIVAL	Survival LCL	Survival UCL
0		1	1	1
0	1	1		
0	1	1		
0	1	1		
0	1	1		
0	1	1		
0	1	1		
0	1	1		
0.0328542094	0	0.9994036971	0.9957744929	0.9999159812
0.2628336756	0	0.9988073942	0.9952399582	0.9997015986
0.2628336756	1	0.9988073942		
0.295687885	0	0.9976140759	0.993655537	0.9991038523
0.295687885	1	0.9976140759		
0.3285420945	0	0.9970170597	0.9928483752	0.9987573355
0.3613963039	1	0.9970170597		
0.3942505133	1	0.9970170597		
0.4599589322	1	0.9970170597		
0.4599589322	1	0.9970170597		
0.4599589322	1	0.9970170597		
0.5585215606	0	0.9964182506	0.9920449392	0.9983892744
0.59137577	1	0.9964182506		
0.6899383984	1	0.9964182506		
0.6899383984	1	0.9964182506		
0.7227926078	0	0.9958183601	0.9912486583	0.9980042927
0.7885010267	1	0.9958183601		
0.7885010267	1	0.9958183601		
0.7885010267	1	0.9958183601		
0.8213552361	0	0.9952173834	0.9904594377	0.9976053729
0.887063655	0	0.99401543	0.9889058927	0.9967755346
0.9856262834	0	0.9934144533	0.9881399094	0.9963475769
1.0513347023	0	0.9928134767	0.9873801707	0.9959123788
1.0841889117	0	0.9922125	0.9866260823	0.9954707658
1.0841889117	1	0.9922125		
1.0841889117	1	0.9922125		
1.1498973306	0	0.9916107944	0.9858759023	0.9950229851
1.2484599589	0	0.9910090888	0.9851304581	0.994570018
1.2813141684	1	0.9910090888		
1.3798767967	0	0.9904070177	0.9843887593	0.9941121205
1.4127310062	1	0.9904070177		
1.4127310062	1	0.9904070177		
1.5441478439	0	0.9898042137	0.983649898	0.9936494635
1.5770020534	0	0.9892014096	0.9829147781	0.9931828433
1.6098562628	0	0.9885986056	0.9821831263	0.9927125683
1.7412731006	1	0.9885986056		
1.7741273101	0	0.9879954338	0.9814541317	0.9922386713
1.839835729	1	0.9879954338		
2.0041067762	0	0.9873918935	0.9807275963	0.9917613822
2.0698151951	1	0.9873918935		
2.3983572895	1	0.9873918935		

2.4640657084	1	0.9873918935		
2.5297741273	1	0.9873918935		
2.5626283368	0	0.986786874	0.9800016588	0.9912801743
2.6283367556	0	0.9861818544	0.9792784291	0.9907961915
2.6283367556	1	0.9861818544		
2.6611909651	1	0.9861818544		
2.726899384	0	0.9855760916	0.9785566372	0.9903091068
2.8583162218	0	0.9849703288	0.9778372623	0.9898195635
2.8583162218	1	0.9849703288		
2.8583162218	1	0.9849703288		
2.8911704312	1	0.9849703288		
3.022587269	1	0.9849703288		
3.1211498973	0	0.9843630721	0.9771179779	0.9893266855
3.2525667351	0	0.9831485588	0.9756858701	0.9883344239
3.318275154	0	0.9819340454	0.974261658	0.9873342166
3.4496919918	0	0.9813267887	0.973552276	0.986831373
3.4825462012	1	0.9813267887		
3.6796714579	0	0.9807191561	0.9728440678	0.9863265498
3.7125256674	1	0.9807191561		
3.9753593429	1	0.9807191561		
4.0410677618	0	0.9801107695	0.9721364359	0.9858195506
4.1396303901	1	0.9801107695		
4.2381930185	0	0.979502005	0.9714298514	0.9853107067
4.3367556468	1	0.979502005		
4.7967145791	0	0.978283719	0.9700201157	0.9842880006
4.8952772074	0	0.9770654329	0.96861598	0.9832597135
5.0266940452	0	0.9764562898	0.967915878	0.9827436102
5.0595482546	0	0.9758471468	0.9672170219	0.9822262646
5.0924024641	0	0.9752380038	0.9665193669	0.9817077212
5.158110883	1	0.9752380038		
5.3223819302	1	0.9752380038		
5.3552361396	1	0.9752380038		
5.4209445585	0	0.9746277172	0.9658213273	0.9811871769
5.5852156057	1	0.9746277172		
5.6837782341	0	0.9740170482	0.9651239041	0.9806652262
5.8151950719	1	0.9740170482		
5.8151950719	1	0.9740170482		
5.8809034908	0	0.9734056125	0.9644265494	0.9801416177
5.8809034908	1	0.9734056125		
5.9137577002	0	0.9727937925	0.9637297488	0.9796166664
5.9794661191	0	0.9721819725	0.963033985	0.979090691
6.0780287474	1	0.9721819725		
6.1437371663	0	0.9709575619	0.9616444177	0.9780352135
6.1765913758	0	0.9703453567	0.9609510685	0.9775060581
6.2094455852	0	0.9697331514	0.9602586376	0.9769759954
6.4394250513	0	0.9691209461	0.9595670981	0.9764450515
6.4722792608	0	0.9685087408	0.9588764248	0.9759132512
6.4722792608	1	0.9685087408		
6.5051334702	0	0.9678961483	0.9581860878	0.9753803222
6.5051334702	1	0.9678961483		

6.5708418891	0	0.9666701874	0.9568068487	0.9743114544
6.5708418891	1	0.9666701874		
6.5708418891	1	0.9666701874		
6.6694045175	1	0.9666701874		
6.7679671458	0	0.9660560386	0.9561168969	0.9737749542
6.8336755647	0	0.9654418899	0.9554277133	0.9732376981
6.932238193	0	0.9648277411	0.9547392781	0.9726997052
6.9650924025	1	0.9648277411		
7.2936344969	1	0.9648277411		
7.3264887064	1	0.9648277411		
7.3264887064	1	0.9648277411		
7.5893223819	1	0.9648277411		
7.6550308008	0	0.9642116314	0.9540490476	0.9721594746
7.8521560575	0	0.9635955217	0.9533595404	0.9716185359
8.0492813142	1	0.9635955217		
8.0492813142	1	0.9635955217		
8.0492813142	1	0.9635955217		
8.1149897331	1	0.9635955217		
8.2792607803	1	0.9635955217		
8.5749486653	1	0.9635955217		
8.6078028747	1	0.9635955217		
8.6735112936	0	0.9629766421	0.9526671848	0.9710747525
8.772073922	0	0.9623577626	0.9519755338	0.9705302846
8.9691991786	1	0.9623577626		
9.1334702259	1	0.9623577626		
9.2320328542	1	0.9623577626		
9.5934291581	0	0.9617376867	0.9512830409	0.9699842147
9.7577002053	1	0.9617376867		
9.8234086242	1	0.9617376867		
9.9219712526	0	0.9611168102	0.9505902041	0.9694368619
9.9219712526	1	0.9611168102		
9.954825462	1	0.9611168102		
10.020533881	1	0.9611168102		
10.05338809	0	0.9604947281	0.9498964975	0.9688879203
10.05338809	1	0.9604947281		
10.184804928	0	0.9598722429	0.9492029355	0.9683380279
10.184804928	1	0.9598722429		
10.184804928	1	0.9598722429		
10.184804928	1	0.9598722429		
10.283367556	1	0.9598722429		
10.283367556	1	0.9598722429		
10.349075975	0	0.9586232224	0.9478125968	0.9672332039
10.349075975	1	0.9586232224		
10.349075975	1	0.9586232224		
10.414784394	1	0.9586232224		
10.447638604	1	0.9586232224		
10.480492813	1	0.9586232224		
10.480492813	1	0.9586232224		
10.480492813	1	0.9586232224		
10.513347023	1	0.9586232224		

10.513347023	1	0.9586232224		
10.513347023	1	0.9586232224		
10.710472279	1	0.9586232224		
10.710472279	1	0.9586232224		
10.710472279	1	0.9586232224		
10.743326489	1	0.9586232224		
10.743326489	1	0.9586232224		
10.743326489	1	0.9586232224		
10.776180698	1	0.9586232224		
10.907597536	0	0.9579917183	0.947109509	0.9666743791
10.907597536	1	0.9579917183		
10.940451745	1	0.9579917183		
10.940451745	1	0.9579917183		
10.973305955	0	0.9573589628	0.9464054829	0.9661139585
10.973305955	1	0.9573589628		
11.039014374	1	0.9573589628		
11.071868583	1	0.9573589628		
11.071868583	1	0.9573589628		
11.071868583	1	0.9573589628		
11.104722793	1	0.9573589628		
11.170431211	1	0.9573589628		
11.170431211	1	0.9573589628		
11.433264887	1	0.9573589628		
11.466119097	0	0.956722421	0.9456973026	0.9655499529
11.498973306	1	0.956722421		
11.531827515	0	0.9560854553	0.94498923	0.9649850122
11.531827515	1	0.9560854553		
11.564681725	1	0.9560854553		
11.696098563	0	0.9554476398	0.9442807156	0.9644188104
11.728952772	0	0.9548098243	0.9435728227	0.9638520285
11.761806982	0	0.9528963778	0.9414527405	0.9621483171
11.761806982	1	0.9528963778		
11.761806982	1	0.9528963778		
11.794661191	1	0.9528963778		
11.86036961	1	0.9528963778		
11.893223819	0	0.95225685	0.9407450598	0.961577964
11.893223819	1	0.95225685		
11.926078029	0	0.9516168924	0.9400374079	0.961006738
12.024640657	0	0.9509769349	0.9393303126	0.9604349908
12.024640657	1	0.9509769349		
12.024640657	1	0.9509769349		
12.057494867	0	0.9503361148	0.9386226876	0.9598620421
12.221765914	0	0.9496952948	0.9379156009	0.9592885891
12.254620123	1	0.9496952948		
12.254620123	1	0.9496952948		
12.320328542	1	0.9496952948		
12.353182752	1	0.9496952948		
12.353182752	1	0.9496952948		
12.353182752	1	0.9496952948		
12.517453799	1	0.9496952948		

12.583162218	1	0.9496952948		
12.583162218	1	0.9496952948		
12.681724846	1	0.9496952948		
12.714579055	1	0.9496952948		
12.747433265	0	0.9490496828	0.9372030798	0.9587107998
12.813141684	1	0.9490496828		
12.813141684	1	0.9490496828		
12.944558522	1	0.9490496828		
12.977412731	1	0.9490496828		
13.01026694	1	0.9490496828		
13.01026694	1	0.9490496828		
13.01026694	1	0.9490496828		
13.04312115	1	0.9490496828		
13.108829569	0	0.948400538	0.9364867004	0.9581296801
13.108829569	1	0.948400538		
13.207392197	1	0.948400538		
13.273100616	1	0.948400538		
13.273100616	1	0.948400538		
13.437371663	1	0.948400538		
13.503080082	0	0.947749164	0.9357680894	0.9575462738
13.568788501	0	0.94709779	0.9350500171	0.956962372
13.700205339	1	0.94709779		
13.733059548	1	0.94709779		
13.733059548	1	0.94709779		
13.765913758	0	0.9464450692	0.9343308027	0.9563769008
13.995893224	0	0.9457923485	0.9336121118	0.9557909479
14.028747433	1	0.9457923485		
14.061601643	0	0.945139177	0.9328933769	0.9552041584
14.061601643	1	0.945139177		
14.094455852	1	0.945139177		
14.094455852	1	0.945139177		
14.19301848	1	0.945139177		
14.258726899	0	0.9444841949	0.93217291	0.9546154418
14.291581109	1	0.9444841949		
14.291581109	1	0.9444841949		
14.291581109	1	0.9444841949		
14.324435318	1	0.9444841949		
14.324435318	1	0.9444841949		
14.422997947	0	0.9438269338	0.9314501314	0.9540244224
14.422997947	1	0.9438269338		
14.488706366	1	0.9438269338		
14.488706366	1	0.9438269338		
14.488706366	1	0.9438269338		
14.521560575	0	0.9431678368	0.9307255892	0.9534314593
14.521560575	1	0.9431678368		
14.521560575	1	0.9431678368		
14.587268994	1	0.9431678368		
14.718685832	1	0.9431678368		
14.718685832	1	0.9431678368		
14.751540041	1	0.9431678368		

14.784394251	1	0.9431678368		
14.784394251	1	0.9431678368		
14.784394251	1	0.9431678368		
14.850102669	1	0.9431678368		
14.850102669	1	0.9431678368		
14.882956879	1	0.9431678368		
14.981519507	0	0.942503166	0.9299946685	0.9528335353
15.014373717	1	0.942503166		
15.047227926	1	0.942503166		
15.112936345	1	0.942503166		
15.145790554	1	0.942503166		
15.178644764	0	0.941836615	0.9292619384	0.9522336293
15.244353183	1	0.941836615		
15.277207392	1	0.941836615		
15.277207392	1	0.941836615		
15.277207392	1	0.941836615		
15.310061602	0	0.9411681717	0.9285273834	0.9516317334
15.310061602	1	0.9411681717		
15.310061602	1	0.9411681717		
15.310061602	1	0.9411681717		
15.342915811	1	0.9411681717		
15.375770021	1	0.9411681717		
15.40862423	1	0.9411681717		
15.441478439	0	0.9404968677	0.9277898091	0.9510270665
15.441478439	1	0.9404968677		
15.474332649	0	0.9398250842	0.9270521509	0.9504215557
15.474332649	1	0.9398250842		
15.540041068	1	0.9398250842		
15.638603696	0	0.939152339	0.926313809	0.9498148159
15.671457906	0	0.9384795937	0.9255759606	0.9492076319
15.704312115	1	0.9384795937		
15.770020534	1	0.9384795937		
15.868583162	0	0.9378058826	0.9248374119	0.9485992257
15.868583162	1	0.9378058826		
15.934291581	1	0.9378058826		
15.934291581	1	0.9378058826		
15.967145791	1	0.9378058826		
16.164271047	1	0.9378058826		
16.229979466	1	0.9378058826		
16.229979466	1	0.9378058826		
16.229979466	1	0.9378058826		
16.295687885	1	0.9378058826		
16.295687885	1	0.9378058826		
16.328542094	1	0.9378058826		
16.328542094	1	0.9378058826		
16.361396304	1	0.9378058826		
16.361396304	1	0.9378058826		
16.459958932	0	0.936444771	0.9233449724	0.9473699837
16.459958932	1	0.936444771		
16.492813142	1	0.936444771		

16.492813142	1	0.936444771		
16.492813142	1	0.936444771		
16.492813142	1	0.936444771		
16.525667351	0	0.9357617333	0.9225964434	0.9467526892
16.525667351	1	0.9357617333		
16.657084189	1	0.9357617333		
16.657084189	1	0.9357617333		
16.689938398	1	0.9357617333		
16.689938398	1	0.9357617333		
16.722792608	0	0.9350761935	0.9218453349	0.9461329258
16.755646817	0	0.9343906538	0.9210947115	0.9455127308
16.854209446	1	0.9343906538		
16.854209446	1	0.9343906538		
16.854209446	1	0.9343906538		
16.887063655	1	0.9343906538		
16.887063655	1	0.9343906538		
16.952772074	1	0.9343906538		
16.952772074	1	0.9343906538		
17.084188912	0	0.9337015752	0.9203402368	0.9448892262
17.084188912	1	0.9337015752		
17.084188912	1	0.9337015752		
17.084188912	1	0.9337015752		
17.117043121	1	0.9337015752		
17.149897331	1	0.9337015752		
17.248459959	1	0.9337015752		
17.347022587	1	0.9337015752		
17.347022587	1	0.9337015752		
17.347022587	1	0.9337015752		
17.379876797	1	0.9337015752		
17.379876797	1	0.9337015752		
17.412731006	0	0.9330068567	0.9195793434	0.9442606997
17.412731006	1	0.9330068567		
17.445585216	0	0.9323116206	0.918818307	0.9436313192
17.445585216	1	0.9323116206		
17.478439425	1	0.9323116206		
17.511293634	1	0.9323116206		
17.577002053	1	0.9323116206		
17.642710472	1	0.9323116206		
17.741273101	0	0.9316137826	0.9180545745	0.9429993899
17.741273101	1	0.9316137826		
17.77412731	0	0.9309154214	0.9172906868	0.9423666078
17.80698152	1	0.9309154214		
17.839835729	1	0.9309154214		
17.839835729	1	0.9309154214		
17.839835729	1	0.9309154214		
17.839835729	1	0.9309154214		
17.872689938	1	0.9309154214		
17.905544148	0	0.9302139026	0.9165234231	0.941730826
17.905544148	1	0.9302139026		
18.004106776	1	0.9302139026		

18.036960986	1	0.9302139026		
18.036960986	1	0.9302139026		
18.036960986	1	0.9302139026		
18.036960986	1	0.9302139026		
18.069815195	1	0.9302139026		
18.069815195	1	0.9302139026		
18.102669405	1	0.9302139026		
18.201232033	1	0.9302139026		
18.234086242	0	0.9295070532	0.9157501333	0.9410902695
18.234086242	1	0.9295070532		
18.299794661	1	0.9295070532		
18.299794661	1	0.9295070532		
18.36550308	0	0.9287985874	0.9149753591	0.9404479668
18.36550308	1	0.9287985874		
18.39835729	1	0.9287985874		
18.464065708	1	0.9287985874		
18.496919918	1	0.9287985874		
18.496919918	1	0.9287985874		
18.529774127	1	0.9287985874		
18.661190965	1	0.9287985874		
18.661190965	1	0.9287985874		
18.661190965	1	0.9287985874		
18.694045175	1	0.9287985874		
18.694045175	1	0.9287985874		
18.726899384	1	0.9287985874		
18.792607803	1	0.9287985874		
18.825462012	0	0.9280830261	0.9141924116	0.9397994458
18.858316222	0	0.9273674648	0.9134099624	0.939150493
18.858316222	1	0.9273674648		
18.891170431	0	0.9266513509	0.9126273299	0.9385006629
18.924024641	1	0.9266513509		
18.924024641	1	0.9266513509		
19.022587269	1	0.9266513509		
19.022587269	1	0.9266513509		
19.055441478	0	0.9259330165	0.9118424773	0.9378485951
19.121149897	0	0.9244963478	0.9102742117	0.9365432086
19.154004107	0	0.9237780134	0.9094907833	0.9358899017
19.154004107	1	0.9237780134		
19.186858316	0	0.92305912	0.9087071368	0.9352357339
19.186858316	1	0.92305912		
19.219712526	1	0.92305912		
19.219712526	1	0.92305912		
19.252566735	1	0.92305912		
19.351129363	1	0.92305912		
19.351129363	1	0.92305912		
19.383983573	1	0.92305912		
19.416837782	0	0.9223362859	0.9079191681	0.9345779291
19.416837782	1	0.9223362859		
19.482546201	1	0.9223362859		
19.482546201	1	0.9223362859		

19.54825462	0	0.9216117484	0.9071295937	0.9339183242
19.58110883	1	0.9216117484		
19.58110883	1	0.9216117484		
19.646817248	1	0.9216117484		
19.712525667	1	0.9216117484		
19.745379877	0	0.9208849252	0.9063377074	0.9332564415
19.778234086	0	0.9201581021	0.9055462764	0.9325941627
19.811088296	1	0.9201581021		
19.843942505	1	0.9201581021		
19.843942505	1	0.9201581021		
19.942505133	0	0.9194295525	0.9047532063	0.9319300703
19.942505133	1	0.9194295525		
20.008213552	1	0.9194295525		
20.041067762	1	0.9194295525		
20.106776181	1	0.9194295525		
20.1724846	1	0.9194295525		
20.271047228	1	0.9194295525		
20.271047228	1	0.9194295525		
20.271047228	1	0.9194295525		
20.271047228	1	0.9194295525		
20.303901437	1	0.9194295525		
20.336755647	0	0.9186951839	0.9039535471	0.9312607936
20.369609856	1	0.9186951839		
20.369609856	1	0.9186951839		
20.369609856	1	0.9186951839		
20.369609856	1	0.9186951839		
20.402464066	1	0.9186951839		
20.435318275	1	0.9186951839		
20.468172485	0	0.9179572761	0.9031500634	0.9305882063
20.501026694	0	0.9172193683	0.902347038	0.9299152229
20.501026694	1	0.9172193683		
20.533880903	1	0.9172193683		
20.566735113	0	0.9164802713	0.9015430281	0.9292408675
20.599589322	0	0.9157411743	0.9007394659	0.9285661241
20.599589322	1	0.9157411743		
20.599589322	1	0.9157411743		
20.599589322	1	0.9157411743		
20.632443532	0	0.9150002834	0.8999341823	0.9278895152
20.632443532	1	0.9150002834		
20.632443532	1	0.9150002834		
20.632443532	1	0.9150002834		
20.632443532	1	0.9150002834		
20.698151951	1	0.9150002834		
20.796714579	1	0.9150002834		
20.829568789	1	0.9150002834		
20.862422998	0	0.9142551692	0.899124248	0.927209034
20.862422998	1	0.9142551692		
20.862422998	1	0.9142551692		
20.862422998	1	0.9142551692		
20.895277207	0	0.9135082287	0.8983125572	0.9265266589

20.895277207	1	0.9135082287		
20.928131417	0	0.9127606769	0.8975005727	0.9258433957
20.993839836	1	0.9127606769		
21.158110883	1	0.9127606769		
21.158110883	1	0.9127606769		
21.190965092	0	0.9120112839	0.8966868101	0.9251582295
21.190965092	1	0.9120112839		
21.256673511	0	0.9112612747	0.8958727423	0.9244721757
21.256673511	1	0.9112612747		
21.256673511	1	0.9112612747		
21.256673511	1	0.9112612747		
21.388090349	1	0.9112612747		
21.420944559	0	0.9105087881	0.8950561284	0.9237836948
21.420944559	1	0.9105087881		
21.420944559	1	0.9105087881		
21.453798768	1	0.9105087881		
21.486652977	0	0.9097544312	0.8942376991	0.9230932898
21.585215606	1	0.9097544312		
21.650924025	1	0.9097544312		
21.683778234	1	0.9097544312		
21.683778234	1	0.9097544312		
21.848049281	0	0.9089975639	0.8934166853	0.9224004305
21.880903491	1	0.9089975639		
21.880903491	1	0.9089975639		
21.880903491	1	0.9089975639		
21.9137577	1	0.9089975639		
21.9137577	1	0.9089975639		
21.9137577	1	0.9089975639		
21.979466119	1	0.9089975639		
22.078028747	1	0.9089975639		
22.143737166	1	0.9089975639		
22.242299795	0	0.9082349821	0.8925892388	0.9217024596
22.308008214	1	0.9082349821		
22.308008214	1	0.9082349821		
22.308008214	1	0.9082349821		
22.340862423	1	0.9082349821		
22.340862423	1	0.9082349821		
22.373716632	1	0.9082349821		
22.439425051	1	0.9082349821		
22.439425051	1	0.9082349821		
22.570841889	0	0.9074672433	0.8917560351	0.9209998361
22.570841889	1	0.9074672433		
22.603696099	1	0.9074672433		
22.603696099	1	0.9074672433		
22.669404517	1	0.9074672433		
22.702258727	0	0.9066968976	0.8909201446	0.9202946699
22.735112936	0	0.9059265519	0.8900846997	0.9195891221
22.767967146	1	0.9059265519		
22.800821355	0	0.9051555505	0.8892489074	0.9188826532
22.866529774	1	0.9051555505		

22.899383984	0	0.9043838919	0.888412762	0.9181752648
22.965092402	1	0.9043838919		
23.12936345	1	0.9043838919		
23.162217659	0	0.9036109142	0.8875754669	0.9174664096
23.227926078	0	0.9028379366	0.8867385993	0.9167571867
23.293634497	0	0.9020649589	0.8859021534	0.9160476006
23.359342916	1	0.9020649589		
23.359342916	1	0.9020649589		
23.392197125	0	0.9012906542	0.885064538	0.9153365493
23.425051335	1	0.9012906542		
23.457905544	1	0.9012906542		
23.490759754	0	0.9005150168	0.8842257445	0.9146240295
23.490759754	1	0.9005150168		
23.523613963	1	0.9005150168		
23.622176591	0	0.8997380409	0.8833857648	0.9139100378
23.655030801	0	0.8989610651	0.8825461939	0.913195693
23.655030801	1	0.8989610651		
23.917864476	0	0.8974057691	0.8808666568	0.9117648358
24.049281314	0	0.8966281211	0.8800274828	0.911048892
24.246406571	1	0.8966281211		
24.344969199	1	0.8966281211		
24.443531828	1	0.8966281211		
24.542094456	1	0.8966281211		
24.574948665	1	0.8966281211		
24.640657084	0	0.8958470862	0.8791846705	0.9103297732
24.640657084	1	0.8958470862		
25.297741273	0	0.8950653697	0.87834144	0.9096097435
25.297741273	1	0.8950653697		
25.297741273	1	0.8950653697		
25.363449692	1	0.8950653697		
25.49486653	0	0.8942815997	0.8774961582	0.9088876553
25.527720739	1	0.8942815997		
25.560574949	1	0.8942815997		
25.626283368	1	0.8942815997		
25.823408624	0	0.8934957635	0.8766488103	0.9081634979
25.856262834	0	0.8927099273	0.8758018503	0.9074390044
25.856262834	1	0.8927099273		
25.889117043	0	0.8919233987	0.874954452	0.9067135971
25.889117043	1	0.8919233987		
25.954825462	1	0.8919233987		
25.987679671	0	0.8911354805	0.874105786	0.9059866925
26.184804928	0	0.8903475623	0.873257498	0.9052594593
26.184804928	1	0.8903475623		
26.217659138	0	0.8895589462	0.8724087573	0.9045313142
26.414784394	1	0.8895589462		
26.447638604	0	0.8887696304	0.8715595591	0.9038022574
26.874743326	0	0.8879803146	0.8707107284	0.9030728805
27.071868583	1	0.8879803146		
27.23613963	0	0.8871902965	0.8698614311	0.9023425947
27.26899384	0	0.8864002784	0.8690124941	0.9016119944

27.26899384	1	0.8864002784		
27.301848049	0	0.8856095556	0.8681630816	0.9008804879
27.400410678	0	0.8848188328	0.8673140225	0.9001486722
27.433264887	0	0.88402811	0.8664653128	0.8994165504
27.433264887	1	0.88402811		
27.466119097	1	0.88402811		
27.531827515	1	0.88402811		
27.564681725	0	0.8832352596	0.8656144428	0.8986823264
27.794661191	0	0.8824424093	0.8647639185	0.8979477995
27.86036961	1	0.8824424093		
27.893223819	1	0.8824424093		
28.090349076	0	0.8816481317	0.8639120567	0.8972117644
28.123203285	1	0.8816481317		
28.123203285	1	0.8816481317		
28.156057495	1	0.8816481317		
28.221765914	0	0.8808517016	0.8630580038	0.8964736074
28.45174538	1	0.8808517016		
28.484599589	0	0.8792573999	0.8613492236	0.8949951775
28.714579055	0	0.878460249	0.8604953375	0.8942555198
28.747433265	0	0.8768659473	0.8587885552	0.8927753334
28.878850103	0	0.8760687964	0.8579356521	0.8920348102
29.207392197	0	0.8752716456	0.8570830697	0.8912940037
29.273100616	0	0.8744744947	0.8562308048	0.8905529168
29.404517454	1	0.8744744947		
29.535934292	1	0.8744744947		
29.60164271	1	0.8744744947		
29.700205339	0	0.8736751579	0.8553762971	0.8898096904
29.765913758	0	0.872875821	0.8545221041	0.8890661858
29.897330595	1	0.872875821		
29.963039014	1	0.872875821		
30.521560575	0	0.8720750175	0.8536665086	0.8883211555
30.652977413	0	0.871274214	0.8528112238	0.8875758503
30.652977413	1	0.871274214		
30.751540041	1	0.871274214		
30.850102669	0	0.8704719358	0.851954525	0.8868290142
30.981519507	0	0.8696696575	0.8510981331	0.8860819063
31.441478439	0	0.8688673792	0.8502420453	0.8853345289
31.474332649	0	0.8680651009	0.8493862584	0.8845868844
31.901437372	0	0.8672628226	0.8485307695	0.8838389751
32.098562628	0	0.8656582661	0.8468206744	0.8823423712
32.394250513	0	0.8648559878	0.8459660625	0.8815936812
32.427104723	1	0.8648559878		
32.755646817	1	0.8648559878		
32.952772074	0	0.8640522183	0.8451100044	0.8808434572
32.985626283	0	0.8632484488	0.8442542327	0.8800929777
33.051334702	1	0.8632484488		
33.412731006	1	0.8632484488		
33.741273101	1	0.8632484488		
33.872689938	1	0.8632484488		
33.905544148	1	0.8632484488		

33.938398357	1	0.8632484488		
33.938398357	1	0.8632484488		
34.036960986	1	0.8632484488		
34.135523614	1	0.8632484488		
34.135523614	1	0.8632484488		
34.299794661	0	0.862437125	0.8433899679	0.8793357689
34.332648871	1	0.862437125		
34.39835729	1	0.862437125		
34.39835729	1	0.862437125		
34.496919918	1	0.862437125		
34.529774127	1	0.862437125		
34.562628337	1	0.862437125		
34.562628337	1	0.862437125		
34.595482546	0	0.8616204232	0.8425197427	0.8785736933
34.694045175	0	0.8608037214	0.841649816	0.8778113537
34.726899384	0	0.8599870196	0.8407801848	0.8770487522
34.759753593	1	0.8599870196		
34.792607803	0	0.8591695414	0.8399099449	0.8762852253
34.858316222	1	0.8591695414		
34.891170431	0	0.8583512847	0.8390390925	0.8755207721
35.022587269	1	0.8583512847		
35.186858316	1	0.8583512847		
35.219712526	0	0.857531465	0.838166716	0.8747547204
35.219712526	1	0.857531465		
35.219712526	1	0.857531465		
35.252566735	1	0.857531465		
35.515400411	0	0.8567092871	0.8372918931	0.8739863878
35.515400411	1	0.8567092871		
35.515400411	1	0.8567092871		
35.515400411	1	0.8567092871		
35.515400411	1	0.8567092871		
35.515400411	1	0.8567092871		
35.58110883	1	0.8567092871		
35.679671458	1	0.8567092871		
35.679671458	1	0.8567092871		
35.712525667	1	0.8567092871		
35.909650924	1	0.8567092871		
35.909650924	1	0.8567092871		
35.942505133	0	0.8558783373	0.8364071774	0.8732102728
36.008213552	1	0.8558783373		
36.008213552	1	0.8558783373		
36.041067762	1	0.8558783373		
36.1724846	1	0.8558783373		
36.205338809	0	0.8550441479	0.8355189999	0.8724311153
36.205338809	1	0.8550441479		
36.205338809	1	0.8550441479		
36.271047228	1	0.8550441479		
36.402464066	1	0.8550441479		
36.402464066	1	0.8550441479		
36.435318275	0	0.8542058693	0.8346263754	0.871648186

36.435318275	1	0.8542058693		
36.468172485	1	0.8542058693		
36.501026694	0	0.8533659422	0.8337321428	0.870863574
36.665297741	1	0.8533659422		
36.698151951	1	0.8533659422		
36.829568789	1	0.8533659422		
36.862422998	0	0.8525235276	0.8328353277	0.8700765604
36.862422998	1	0.8525235276		
36.895277207	1	0.8525235276		
37.092402464	0	0.8516794449	0.8319368832	0.8692878464
37.158110883	1	0.8516794449		
37.190965092	1	0.8516794449		
37.519507187	1	0.8516794449		
37.519507187	1	0.8516794449		
37.585215606	1	0.8516794449		
37.618069815	0	0.8508311586	0.8310338648	0.8684952588
37.650924025	1	0.8508311586		
37.782340862	1	0.8508311586		
37.782340862	1	0.8508311586		
37.815195072	1	0.8508311586		
37.815195072	1	0.8508311586		
37.848049281	1	0.8508311586		
37.880903491	1	0.8508311586		
37.9137577	1	0.8508311586		
38.078028747	0	0.8499760519	0.8301232333	0.8676965544
38.373716632	0	0.8491209453	0.8292129209	0.8668975716
38.50513347	1	0.8491209453		
38.702258727	1	0.8491209453		
38.702258727	1	0.8491209453		
38.899383984	1	0.8491209453		
38.899383984	1	0.8491209453		
38.932238193	1	0.8491209453		
39.162217659	1	0.8491209453		
39.195071869	1	0.8491209453		
39.227926078	1	0.8491209453		
39.359342916	1	0.8491209453		
39.392197125	1	0.8491209453		
39.392197125	1	0.8491209453		
39.490759754	1	0.8491209453		
39.490759754	1	0.8491209453		
39.589322382	1	0.8491209453		
39.622176591	1	0.8491209453		
39.68788501	1	0.8491209453		
39.72073922	1	0.8491209453		
39.72073922	1	0.8491209453		
39.753593429	0	0.8482491578	0.8282835233	0.8660840376
39.852156057	1	0.8482491578		
39.852156057	1	0.8482491578		
39.852156057	1	0.8482491578		
39.917864476	1	0.8482491578		

39.917864476	1	0.8482491578		
39.983572895	0	0.8473728674	0.8273492207	0.8652663619
40.082135524	1	0.8473728674		
40.114989733	1	0.8473728674		
40.147843943	1	0.8473728674		
40.180698152	1	0.8473728674		
40.180698152	1	0.8473728674		
40.344969199	1	0.8473728674		
40.509240246	1	0.8473728674		
40.574948665	1	0.8473728674		
40.772073922	1	0.8473728674		
40.772073922	1	0.8473728674		
40.804928131	1	0.8473728674		
40.837782341	1	0.8473728674		
40.837782341	1	0.8473728674		
40.837782341	1	0.8473728674		
40.87063655	1	0.8473728674		
40.936344969	0	0.8464827698	0.8263991623	0.8644366066
41.100616016	0	0.8455926723	0.825449468	0.8636065389
41.133470226	0	0.8447025747	0.8245001343	0.8627761613
41.264887064	1	0.8447025747		
41.297741273	1	0.8447025747		
41.330595483	1	0.8447025747		
41.49486653	0	0.8438096545	0.8235478672	0.8619430673
41.527720739	1	0.8438096545		
41.527720739	1	0.8438096545		
41.593429158	0	0.8429148404	0.8225937512	0.8611080486
41.691991786	1	0.8429148404		
41.691991786	1	0.8429148404		
41.691991786	1	0.8429148404		
41.691991786	1	0.8429148404		
41.757700205	1	0.8429148404		
41.790554415	1	0.8429148404		
41.790554415	1	0.8429148404		
41.823408624	0	0.8420133272	0.8216321843	0.8602670045
41.889117043	1	0.8420133272		
41.954825462	1	0.8420133272		
42.020533881	1	0.8420133272		
42.151950719	1	0.8420133272		
42.151950719	1	0.8420133272		
42.184804928	1	0.8420133272		
42.184804928	1	0.8420133272		
42.217659138	1	0.8420133272		
42.381930185	1	0.8420133272		
42.381930185	1	0.8420133272		
42.414784394	1	0.8420133272		
42.447638604	0	0.8411010701	0.820658447	0.8594164871
42.447638604	1	0.8411010701		
42.447638604	1	0.8411010701		
42.480492813	1	0.8411010701		

42.546201232	0	0.840185835	0.8196816134	0.8585631076
42.611909651	0	0.8392705999	0.818705162	0.8577094018
42.64476386	1	0.8392705999		
42.710472279	1	0.8392705999		
42.743326489	0	0.8383533642	0.8177267552	0.8568536671
42.874743326	1	0.8383533642		
42.940451745	1	0.8383533642		
43.006160164	1	0.8383533642		
43.104722793	1	0.8383533642		
43.104722793	1	0.8383533642		
43.137577002	1	0.8383533642		
43.170431211	0	0.8374300676	0.8167416552	0.8559924422
43.203285421	0	0.8365067709	0.8157569399	0.8551308891
43.23613963	0	0.8355834743	0.8147726055	0.8542690105
43.26899384	0	0.8346601776	0.8137886483	0.8534068094
43.367556468	1	0.8346601776		
43.400410678	1	0.8346601776		
43.400410678	1	0.8346601776		
43.400410678	1	0.8346601776		
43.400410678	1	0.8346601776		
43.531827515	1	0.8346601776		
43.597535934	1	0.8346601776		
43.597535934	1	0.8346601776		
43.630390144	1	0.8346601776		
43.761806982	1	0.8346601776		
44.090349076	1	0.8346601776		
44.090349076	1	0.8346601776		
44.123203285	0	0.8337244599	0.8127905863	0.852533691
44.320328542	1	0.8337244599		
44.320328542	1	0.8337244599		
44.714579055	1	0.8337244599		
44.911704312	0	0.832785581	0.8117892279	0.8516575446
44.911704312	1	0.832785581		
44.911704312	1	0.832785581		
44.911704312	1	0.832785581		
44.911704312	1	0.832785581		
44.977412731	1	0.832785581		
45.01026694	1	0.832785581		
45.04312115	0	0.8318403079	0.8107808019	0.8507756144
45.108829569	1	0.8318403079		
45.174537988	0	0.8308939595	0.8097715213	0.8498924275
45.174537988	1	0.8308939595		
45.207392197	1	0.8308939595		
45.240246407	0	0.8299454504	0.8087601187	0.8490070601
45.404517454	1	0.8299454504		
45.568788501	1	0.8299454504		
45.667351129	0	0.8289947683	0.8077465803	0.8481195012
45.667351129	1	0.8289947683		
45.667351129	1	0.8289947683		
45.831622177	1	0.8289947683		

45.831622177	1	0.8289947683		
45.930184805	1	0.8289947683		
45.930184805	1	0.8289947683		
45.930184805	1	0.8289947683		
45.930184805	1	0.8289947683		
45.963039014	0	0.8280352836	0.8067231721	0.847224101
45.963039014	1	0.8280352836		
46.061601643	1	0.8280352836		
46.127310062	0	0.8270735702	0.8056975727	0.8463264545
46.127310062	1	0.8270735702		
46.127310062	1	0.8270735702		
46.160164271	1	0.8270735702		
46.258726899	0	0.8261084902	0.8046684552	0.8454255918
46.258726899	1	0.8261084902		
46.291581109	1	0.8261084902		
46.291581109	1	0.8261084902		
46.357289528	1	0.8261084902		
46.357289528	1	0.8261084902		
46.390143737	1	0.8261084902		
46.488706366	1	0.8261084902		
46.718685832	1	0.8261084902		
46.718685832	1	0.8261084902		
46.751540041	1	0.8261084902		
46.850102669	1	0.8261084902		
46.915811088	0	0.825130847	0.8036250816	0.8445136845
46.948665298	1	0.825130847		
46.981519507	1	0.825130847		
47.014373717	1	0.825130847		
47.014373717	1	0.825130847		
47.145790554	1	0.825130847		
47.178644764	1	0.825130847		
47.211498973	1	0.825130847		
47.211498973	1	0.825130847		
47.277207392	0	0.8241438484	0.8025712019	0.8435934607
47.441478439	1	0.8241438484		
47.441478439	1	0.8241438484		
47.474332649	1	0.8241438484		
47.507186858	1	0.8241438484		
47.507186858	1	0.8241438484		
47.704312115	1	0.8241438484		
47.737166324	1	0.8241438484		
47.835728953	0	0.8231485055	0.8015080025	0.8426657772
47.868583162	1	0.8231485055		
47.967145791	1	0.8231485055		
47.967145791	1	0.8231485055		
47.967145791	1	0.8231485055		
48	1	0.8231485055		
48.131416838	1	0.8231485055		
48.131416838	1	0.8231485055		
48.164271047	1	0.8231485055		

48.229979466	1	0.8231485055		
48.262833676	0	0.8221422115	0.8004324319	0.8417284221
48.328542094	1	0.8221422115		
48.361396304	1	0.8221422115		
48.459958932	1	0.8221422115		
48.459958932	1	0.8221422115		
48.558521561	1	0.8221422115		
48.624229979	1	0.8221422115		
48.689938398	1	0.8221422115		
48.788501027	1	0.8221422115		
48.919917864	1	0.8221422115		
49.117043121	1	0.8221422115		
49.18275154	0	0.8211234479	0.7993427091	0.840780111
49.248459959	1	0.8211234479		
49.347022587	1	0.8211234479		
49.379876797	1	0.8211234479		
49.478439425	1	0.8211234479		
49.609856263	1	0.8211234479		
49.80698152	1	0.8211234479		
49.971252567	0	0.8200970436	0.7982445068	0.8398249264
50.234086242	1	0.8200970436		
50.234086242	1	0.8200970436		
50.266940452	1	0.8200970436		
50.299794661	1	0.8200970436		
50.332648871	0	0.819065475	0.7971407398	0.8388649575
50.39835729	1	0.819065475		
50.464065708	0	0.8180326056	0.7960359498	0.837903467
50.759753593	1	0.8180326056		
50.858316222	1	0.8180326056		
50.891170431	0	0.8169971213	0.7949285926	0.8369393449
50.891170431	1	0.8169971213		
50.924024641	1	0.8169971213		
51.383983573	1	0.8169971213		
51.449691992	1	0.8169971213		
51.58110883	0	0.8159563606	0.7938155452	0.8359703392
51.613963039	1	0.8159563606		
51.876796715	1	0.8159563606		
52.041067762	1	0.8159563606		
52.205338809	0	0.8149116021	0.7926983128	0.8349975278
52.238193018	1	0.8149116021		
52.271047228	1	0.8149116021		
52.369609856	1	0.8149116021		
52.468172485	1	0.8149116021		
52.533880903	0	0.8138614583	0.7915752694	0.8340197353
52.533880903	1	0.8138614583		
52.566735113	0	0.8128099577	0.7904511484	0.833040365
52.566735113	1	0.8128099577		
52.599589322	1	0.8128099577		
52.73100616	0	0.8117557295	0.7893243398	0.8320582582
52.76386037	1	0.8117557295		

52.928131417	1	0.8117557295		
52.928131417	1	0.8117557295		
52.960985626	1	0.8117557295		
52.960985626	1	0.8117557295		
52.993839836	1	0.8117557295		
52.993839836	1	0.8117557295		
53.190965092	0	0.8106918295	0.7881866958	0.8310675405
53.388090349	1	0.8106918295		
53.453798768	1	0.8106918295		
53.453798768	1	0.8106918295		
53.519507187	1	0.8106918295		
53.618069815	1	0.8106918295		
53.683778234	0	0.8096209023	0.7870413278	0.8300704432
53.979466119	0	0.8074790481	0.7847521886	0.8280749153
53.979466119	1	0.8074790481		
54.110882957	1	0.8074790481		
54.439425051	1	0.8074790481		
54.439425051	1	0.8074790481		
54.53798768	1	0.8074790481		
54.636550308	1	0.8074790481		
54.669404517	1	0.8074790481		
54.669404517	1	0.8074790481		
54.702258727	0	0.8063966365	0.7835949336	0.827066778
55.063655031	1	0.8063966365		
55.293634497	0	0.8053127701	0.7824365119	0.8260569591
55.359342916	1	0.8053127701		
55.556468172	1	0.8053127701		
55.589322382	1	0.8053127701		
55.72073922	1	0.8053127701		
55.917864476	1	0.8053127701		
55.950718686	1	0.8053127701		
55.950718686	1	0.8053127701		
55.950718686	1	0.8053127701		
56.016427105	1	0.8053127701		
56.049281314	1	0.8053127701		
56.049281314	1	0.8053127701		
56.180698152	1	0.8053127701		
56.213552361	1	0.8053127701		
56.27926078	1	0.8053127701		
56.27926078	1	0.8053127701		
56.607802875	1	0.8053127701		
56.640657084	1	0.8053127701		
56.772073922	0	0.8042035238	0.7812488032	0.8250252659
56.837782341	0	0.8030942776	0.7800616795	0.8239930864
56.87063655	1	0.8030942776		
56.87063655	1	0.8030942776		
57.067761807	1	0.8030942776		
57.100616016	1	0.8030942776		
57.199178645	1	0.8030942776		
57.297741273	1	0.8030942776		

57.330595483	1	0.8030942776		
57.330595483	1	0.8030942776		
57.429158111	1	0.8030942776		
57.560574949	0	0.8019710688	0.7788587002	0.8229486617
57.823408624	1	0.8019710688		
57.856262834	1	0.8019710688		
58.0862423	1	0.8019710688		
58.0862423	1	0.8019710688		
58.119096509	1	0.8019710688		
58.119096509	1	0.8019710688		
58.151950719	0	0.8008383413	0.7776451074	0.8218957277
58.316221766	1	0.8008383413		
58.447638604	1	0.8008383413		
58.480492813	1	0.8008383413		
58.480492813	1	0.8008383413		
58.513347023	1	0.8008383413		
58.513347023	1	0.8008383413		
58.67761807	1	0.8008383413		
58.743326489	1	0.8008383413		
58.841889117	1	0.8008383413		
58.907597536	1	0.8008383413		
58.940451745	1	0.8008383413		
58.940451745	1	0.8008383413		
58.973305955	1	0.8008383413		
59.137577002	1	0.8008383413		
59.203285421	1	0.8008383413		
59.630390144	1	0.8008383413		
59.630390144	1	0.8008383413		
59.696098563	1	0.8008383413		
60.090349076	1	0.8008383413		
60.090349076	1	0.8008383413		
60.188911704	1	0.8008383413		
60.287474333	1	0.8008383413		
60.353182752	1	0.8008383413		
60.353182752	1	0.8008383413		
60.41889117	1	0.8008383413		
60.616016427	1	0.8008383413		
60.780287474	1	0.8008383413		
60.813141684	0	0.7996606379	0.776378949	0.8208045947
60.813141684	1	0.7996606379		
60.878850103	1	0.7996606379		
61.273100616	1	0.7996606379		
61.273100616	1	0.7996606379		
61.437371663	1	0.7996606379		
61.535934292	1	0.7996606379		
61.667351129	0	0.7984724348	0.7751010468	0.819704112
61.700205339	1	0.7984724348		
61.733059548	1	0.7984724348		
61.963039014	1	0.7984724348		
62.028747433	1	0.7984724348		

62.160164271	1	0.7984724348		
62.22587269	1	0.7984724348		
62.422997947	1	0.7984724348		
62.587268994	1	0.7984724348		
62.587268994	1	0.7984724348		
62.620123203	0	0.7972681024	0.7738047152	0.8185895871
62.620123203	1	0.7972681024		
62.718685832	1	0.7972681024		
62.718685832	1	0.7972681024		
62.915811088	1	0.7972681024		
62.981519507	0	0.7960564487	0.7725004423	0.8174683415
63.047227926	1	0.7960564487		
63.047227926	1	0.7960564487		
63.244353183	0	0.7948410954	0.7711925439	0.8163433819
63.310061602	0	0.793625742	0.7698854185	0.8152177877
63.310061602	1	0.793625742		
63.540041068	1	0.793625742		
63.835728953	1	0.793625742		
63.967145791	1	0.793625742		
64	1	0.793625742		
64.032854209	1	0.793625742		
64.164271047	1	0.793625742		
64.197125257	0	0.7923972192	0.7685634077	0.8140805985
64.229979466	1	0.7923972192		
64.295687885	1	0.7923972192		
64.328542094	1	0.7923972192		
64.525667351	1	0.7923972192		
64.689938398	1	0.7923972192		
64.887063655	0	0.7911590985	0.7672307707	0.8129347774
64.919917864	1	0.7911590985		
64.952772074	1	0.7911590985		
64.985626283	1	0.7911590985		
65.117043121	1	0.7911590985		
65.215605749	1	0.7911590985		
65.248459959	1	0.7911590985		
65.379876797	1	0.7911590985		
65.478439425	0	0.7899072645	0.765882615	0.8117769003
65.609856263	1	0.7899072645		
65.642710472	1	0.7899072645		
65.642710472	1	0.7899072645		
65.708418891	1	0.7899072645		
65.708418891	1	0.7899072645		
65.839835729	1	0.7899072645		
66.036960986	1	0.7899072645		
66.102669405	1	0.7899072645		
66.135523614	1	0.7899072645		
66.135523614	1	0.7899072645		
66.266940452	1	0.7899072645		
66.529774127	1	0.7899072645		
66.759753593	1	0.7899072645		

66.759753593	1	0.7899072645		
66.825462012	1	0.7899072645		
66.858316222	1	0.7899072645		
66.98973306	1	0.7899072645		
67.022587269	1	0.7899072645		
67.055441478	0	0.7886186719	0.7644914138	0.8105878992
67.285420945	1	0.7886186719		
67.318275154	1	0.7886186719		
67.449691992	1	0.7886186719		
67.449691992	1	0.7886186719		
67.54825462	1	0.7886186719		
67.646817248	0	0.7873194649	0.7630884316	0.8093893825
67.646817248	1	0.7873194649		
67.646817248	1	0.7873194649		
67.646817248	1	0.7873194649		
67.778234086	1	0.7873194649		
67.876796715	0	0.7860116252	0.7616760543	0.8081829759
67.876796715	1	0.7860116252		
68.106776181	1	0.7860116252		
68.13963039	1	0.7860116252		
68.13963039	1	0.7860116252		
68.13963039	1	0.7860116252		
68.1724846	1	0.7860116252		
68.238193018	1	0.7860116252		
68.369609856	1	0.7860116252		
68.435318275	0	0.7846861419	0.7602434886	0.8069612496
68.566735113	1	0.7846861419		
68.665297741	1	0.7846861419		
68.665297741	1	0.7846861419		
68.698151951	1	0.7846861419		
68.698151951	1	0.7846861419		
69.289527721	1	0.7846861419		
69.519507187	0	0.783347087	0.7587956331	0.8057275464
69.519507187	1	0.783347087		
69.552361396	1	0.783347087		
69.683778234	0	0.7820034385	0.7573432835	0.8044892345
69.749486653	1	0.7820034385		
69.782340862	1	0.7820034385		
69.815195072	1	0.7820034385		
70.176591376	1	0.7820034385		
70.242299795	1	0.7820034385		
70.275154004	1	0.7820034385		
70.275154004	1	0.7820034385		
70.439425051	1	0.7820034385		
70.472279261	1	0.7820034385		
70.669404517	1	0.7820034385		
70.702258727	1	0.7820034385		
71.425051335	1	0.7820034385		
71.425051335	1	0.7820034385		
71.425051335	1	0.7820034385		

71.457905544	1	0.7820034385		
71.655030801	1	0.7820034385		
71.655030801	1	0.7820034385		
71.655030801	1	0.7820034385		
71.786447639	1	0.7820034385		
72.016427105	1	0.7820034385		
72.049281314	1	0.7820034385		
72.114989733	0	0.780609493	0.7558314271	0.8032088627
72.147843943	1	0.780609493		
72.246406571	1	0.780609493		
72.27926078	1	0.780609493		
72.27926078	1	0.780609493		
72.344969199	1	0.780609493		
72.377823409	1	0.780609493		
72.377823409	1	0.780609493		
72.509240246	1	0.780609493		
72.509240246	1	0.780609493		
72.542094456	1	0.780609493		
72.574948665	1	0.780609493		
72.574948665	1	0.780609493		
72.706365503	1	0.780609493		
72.739219713	1	0.780609493		
72.772073922	1	0.780609493		
72.772073922	1	0.780609493		
72.969199179	1	0.780609493		
73.002053388	1	0.780609493		
73.002053388	1	0.780609493		
73.034907598	1	0.780609493		
73.034907598	1	0.780609493		
73.199178645	0	0.7791612379	0.7542549654	0.8018833764
73.199178645	1	0.7791612379		
73.297741273	1	0.7791612379		
73.297741273	1	0.7791612379		
73.396303901	1	0.7791612379		
73.46201232	1	0.7791612379		
73.691991786	1	0.7791612379		
73.724845996	1	0.7791612379		
73.757700205	1	0.7791612379		
73.856262834	1	0.7791612379		
74.283367556	0	0.7776883433	0.7526498728	0.8005368779
74.316221766	1	0.7776883433		
74.316221766	1	0.7776883433		
74.414784394	1	0.7776883433		
74.579055441	1	0.7776883433		
74.67761807	1	0.7776883433		
74.874743326	0	0.7762013675	0.7510290378	0.7991778714
74.874743326	1	0.7762013675		
74.907597536	0	0.7747115376	0.7494061654	0.7978153963
75.137577002	1	0.7747115376		
75.301848049	1	0.7747115376		

75.498973306	1	0.7747115376		
75.531827515	1	0.7747115376		
75.564681725	1	0.7747115376		
75.696098563	1	0.7747115376		
75.696098563	1	0.7747115376		
75.696098563	1	0.7747115376		
75.728952772	1	0.7747115376		
75.794661191	0	0.7731954681	0.7477527463	0.7964305977
75.8275154	1	0.7731954681		
75.893223819	1	0.7731954681		
75.893223819	1	0.7731954681		
75.893223819	1	0.7731954681		
76.221765914	1	0.7731954681		
76.254620123	1	0.7731954681		
76.287474333	1	0.7731954681		
76.353182752	0	0.7716583001	0.7460750752	0.7950276025
76.386036961	0	0.7685839643	0.7427243952	0.7922178393
76.484599589	1	0.7685839643		
76.484599589	1	0.7685839643		
76.484599589	1	0.7685839643		
76.648870637	1	0.7685839643		
76.648870637	1	0.7685839643		
76.747433265	1	0.7685839643		
76.944558522	1	0.7685839643		
76.977412731	1	0.7685839643		
77.108829569	1	0.7685839643		
77.141683778	1	0.7685839643		
77.305954825	0	0.7670154256	0.7410130651	0.7907858506
77.305954825	1	0.7670154256		
77.371663244	1	0.7670154256		
77.503080082	0	0.7654404452	0.7392954796	0.7893473784
77.535934292	1	0.7654404452		
77.60164271	1	0.7654404452		
77.63449692	1	0.7654404452		
77.63449692	1	0.7654404452		
77.765913758	1	0.7654404452		
77.798767967	1	0.7654404452		
77.864476386	1	0.7654404452		
77.995893224	1	0.7654404452		
78.061601643	1	0.7654404452		
78.291581109	1	0.7654404452		
78.324435318	1	0.7654404452		
78.324435318	1	0.7654404452		
78.488706366	1	0.7654404452		
78.554414784	0	0.7638221779	0.7375265407	0.787872885
78.81724846	1	0.7638221779		
78.81724846	1	0.7638221779		
78.948665298	1	0.7638221779		
78.981519507	0	0.7621935592	0.7357466751	0.7863886848
79.014373717	1	0.7621935592		

79.047227926	1	0.7621935592		
79.047227926	1	0.7621935592		
79.178644764	1	0.7621935592		
79.40862423	1	0.7621935592		
79.474332649	1	0.7621935592		
79.507186858	1	0.7621935592		
79.507186858	1	0.7621935592		
79.638603696	0	0.7588796742	0.7321228498	0.7833706474
79.704312115	0	0.7572227316	0.7303137112	0.7818593845
79.868583162	1	0.7572227316		
79.934291581	1	0.7572227316		
79.967145791	1	0.7572227316		
79.967145791	1	0.7572227316		
80.098562628	1	0.7572227316		
80.131416838	1	0.7572227316		
80.164271047	1	0.7572227316		
80.164271047	1	0.7572227316		
80.197125257	1	0.7572227316		
80.197125257	1	0.7572227316		
80.262833676	0	0.7555287211	0.7284609054	0.7803170457
80.328542094	1	0.7555287211		
80.394250513	1	0.7555287211		
80.558521561	1	0.7555287211		
80.755646817	1	0.7555287211		
80.788501027	1	0.7555287211		
81.018480493	1	0.7555287211		
81.018480493	1	0.7555287211		
81.051334702	1	0.7555287211		
81.117043121	1	0.7555287211		
81.314168378	1	0.7555287211		
81.708418891	1	0.7555287211		
81.77412731	1	0.7555287211		
81.77412731	1	0.7555287211		
81.80698152	1	0.7555287211		
81.80698152	1	0.7555287211		
81.938398357	1	0.7555287211		
81.971252567	1	0.7555287211		
81.971252567	1	0.7555287211		
82.135523614	0	0.753763467	0.7265221726	0.7787166213
82.464065708	1	0.753763467		
82.464065708	1	0.753763467		
82.496919918	1	0.753763467		
82.595482546	1	0.753763467		
82.661190965	1	0.753763467		
82.924024641	1	0.753763467		
83.121149897	1	0.753763467		
83.154004107	1	0.753763467		
83.186858316	1	0.753763467		
83.285420945	1	0.753763467		
83.318275154	1	0.753763467		

83.318275154	1	0.753763467		
83.515400411	1	0.753763467		
83.58110883	1	0.753763467		
83.646817248	1	0.753763467		
83.811088296	1	0.753763467		
83.843942505	1	0.753763467		
84.041067762	1	0.753763467		
84.106776181	0	0.7519205246	0.7244891327	0.7770533798
84.106776181	1	0.7519205246		
84.205338809	1	0.7519205246		
84.238193018	1	0.7519205246		
84.336755647	1	0.7519205246		
84.336755647	1	0.7519205246		
84.501026694	1	0.7519205246		
84.501026694	1	0.7519205246		
84.566735113	1	0.7519205246		
84.895277207	1	0.7519205246		
84.895277207	1	0.7519205246		
84.928131417	1	0.7519205246		
85.125256674	1	0.7519205246		
85.125256674	1	0.7519205246		
85.158110883	0	0.7500169283	0.7223828197	0.7753408314
85.158110883	1	0.7500169283		
85.223819302	1	0.7500169283		
85.256673511	1	0.7500169283		
85.388090349	1	0.7500169283		
85.453798768	1	0.7500169283		
85.486652977	1	0.7500169283		
85.650924025	1	0.7500169283		
86.110882957	1	0.7500169283		
86.308008214	1	0.7500169283		
86.340862423	1	0.7500169283		
86.340862423	1	0.7500169283		
86.406570842	1	0.7500169283		
86.50513347	1	0.7500169283		
86.636550308	1	0.7500169283		
86.735112936	1	0.7500169283		
86.800821355	1	0.7500169283		
86.833675565	1	0.7500169283		
87.030800821	1	0.7500169283		
87.030800821	1	0.7500169283		
87.063655031	1	0.7500169283		
87.260780287	1	0.7500169283		
87.293634497	1	0.7500169283		
87.425051335	1	0.7500169283		
87.753593429	1	0.7500169283		
88.114989733	1	0.7500169283		
88.410677618	1	0.7500169283		
88.410677618	1	0.7500169283		
88.410677618	1	0.7500169283		

88.607802875	1	0.7500169283		
88.673511294	1	0.7500169283		
88.706365503	1	0.7500169283		
88.837782341	1	0.7500169283		
89.034907598	1	0.7500169283		
89.034907598	1	0.7500169283		
89.034907598	1	0.7500169283		
89.166324435	1	0.7500169283		
89.199178645	0	0.747921909	0.7200367284	0.7734796812
89.527720739	1	0.747921909		
89.527720739	1	0.747921909		
89.560574949	1	0.747921909		
89.560574949	1	0.747921909		
89.790554415	1	0.747921909		
90.184804928	0	0.7457971308	0.7176566567	0.7715927833
90.316221766	1	0.7457971308		
90.349075975	0	0.7436662819	0.7152730807	0.7696978658
90.414784394	0	0.7415354329	0.7128937078	0.7677995558
90.447638604	1	0.7415354329		
90.64476386	1	0.7415354329		
90.67761807	1	0.7415354329		
90.743326489	1	0.7415354329		
90.907597536	1	0.7415354329		
90.973305955	1	0.7415354329		
91.23613963	1	0.7415354329		
91.301848049	1	0.7415354329		
91.433264887	1	0.7415354329		
91.498973306	1	0.7415354329		
91.630390144	1	0.7415354329		
91.8275154	1	0.7415354329		
91.86036961	1	0.7415354329		
92.123203285	1	0.7415354329		
92.156057495	1	0.7415354329		
92.254620123	1	0.7415354329		
92.254620123	1	0.7415354329		
92.287474333	1	0.7415354329		
92.287474333	1	0.7415354329		
92.287474333	1	0.7415354329		
92.386036961	0	0.7392746542	0.7103516372	0.7658004656
92.484599589	1	0.7392746542		
92.648870637	0	0.7370069405	0.7078056563	0.7637921436
92.714579055	1	0.7370069405		
92.845995893	1	0.7370069405		
93.04312115	1	0.7370069405		
93.174537988	1	0.7370069405		
93.207392197	1	0.7370069405		
93.207392197	1	0.7370069405		
93.273100616	1	0.7370069405		
93.437371663	1	0.7370069405		
93.503080082	0	0.7346819975	0.7051907603	0.7617372414

93.63449692	1	0.7346819975		
93.930184805	1	0.7346819975		
93.963039014	1	0.7346819975		
93.963039014	1	0.7346819975		
93.963039014	1	0.7346819975		
94.127310062	0	0.7323196759	0.7025328238	0.7596503341
94.160164271	1	0.7323196759		
94.422997947	1	0.7323196759		
94.554414784	1	0.7323196759		
94.784394251	1	0.7323196759		
94.784394251	1	0.7323196759		
94.882956879	1	0.7323196759		
94.915811088	1	0.7323196759		
94.915811088	1	0.7323196759		
94.915811088	1	0.7323196759		
94.981519507	1	0.7323196759		
95.014373717	1	0.7323196759		
95.112936345	1	0.7323196759		
95.112936345	1	0.7323196759		
95.277207392	1	0.7323196759		
95.310061602	1	0.7323196759		
95.507186858	1	0.7323196759		
95.540041068	1	0.7323196759		
95.605749487	1	0.7323196759		
95.704312115	1	0.7323196759		
95.802874743	1	0.7323196759		
95.802874743	1	0.7323196759		
95.802874743	1	0.7323196759		
95.835728953	1	0.7323196759		
96.032854209	1	0.7323196759		
96.065708419	1	0.7323196759		
96.229979466	0	0.7297501332	0.6996095943	0.7574076516
96.295687885	1	0.7297501332		
96.394250513	1	0.7297501332		
96.525667351	1	0.7297501332		
96.755646817	1	0.7297501332		
96.854209446	1	0.7297501332		
96.854209446	1	0.7297501332		
96.985626283	1	0.7297501332		
97.117043121	1	0.7297501332		
97.577002053	1	0.7297501332		
97.77412731	1	0.7297501332		
98.102669405	1	0.7297501332		
98.135523614	1	0.7297501332		
98.464065708	0	0.7270672283	0.696543675	0.7550778493
98.628336756	0	0.7243843234	0.6934862347	0.7527411838
98.759753593	1	0.7243843234		
98.825462012	0	0.7216914449	0.6904237894	0.7503907557
98.825462012	1	0.7216914449		
99.186858316	1	0.7216914449		

99.252566735	1	0.7216914449		
99.613963039	0	0.7189680809	0.6873291331	0.7480119384
99.646817248	0	0.716244717	0.6842425686	0.7456265965
99.942505133	1	0.716244717		
100.07392197	0	0.7135109585	0.6811501865	0.7432274209
100.07392197	1	0.7135109585		
100.10677618	1	0.7135109585		
100.33675565	1	0.7135109585		
100.40246407	1	0.7135109585		
100.56673511	1	0.7135109585		
100.56673511	1	0.7135109585		
100.76386037	1	0.7135109585		
100.82956879	1	0.7135109585		
100.862423	1	0.7135109585		
100.89527721	1	0.7135109585		
100.89527721	1	0.7135109585		
101.09240246	1	0.7135109585		
101.12525667	1	0.7135109585		
101.25667351	1	0.7135109585		
101.25667351	1	0.7135109585		
101.25667351	1	0.7135109585		
101.28952772	1	0.7135109585		
101.32238193	0	0.7105867333	0.6778140241	0.7406853186
101.35523614	0	0.7076625081	0.6744875345	0.7381354215
101.35523614	1	0.7076625081		
101.68377823	1	0.7076625081		
102.04517454	1	0.7076625081		
102.83367556	1	0.7076625081		
102.89938398	1	0.7076625081		
102.89938398	1	0.7076625081		
102.9650924	1	0.7076625081		
103.12936345	1	0.7076625081		
103.16221766	1	0.7076625081		
103.19507187	1	0.7076625081		
103.26078029	0	0.7046122386	0.671002836	0.7354885211
103.2936345	1	0.7046122386		
103.32648871	1	0.7046122386		
103.78644764	1	0.7046122386		
103.78644764	1	0.7046122386		
103.88501027	1	0.7046122386		
104.04928131	1	0.7046122386		
104.24640657	1	0.7046122386		
104.3449692	1	0.7046122386		
104.50924025	1	0.7046122386		
104.67351129	1	0.7046122386		
104.7063655	1	0.7046122386		
104.7063655	1	0.7046122386		
104.73921971	1	0.7046122386		
104.93634497	1	0.7046122386		
105.00205339	1	0.7046122386		

105.00205339	1	0.7046122386		
105.00205339	1	0.7046122386		
105.0349076	1	0.7046122386		
105.0349076	1	0.7046122386		
105.13347023	1	0.7046122386		
105.19917864	1	0.7046122386		
105.26488706	1	0.7046122386		
105.62628337	0	0.7012408882	0.6670950472	0.7326109954
105.65913758	1	0.7012408882		
105.95482546	1	0.7012408882		
106.18480493	1	0.7012408882		
106.28336756	1	0.7012408882		
106.34907598	1	0.7012408882		
106.34907598	1	0.7012408882		
106.38193018	1	0.7012408882		
106.41478439	1	0.7012408882		
106.54620123	1	0.7012408882		
106.61190965	1	0.7012408882		
106.61190965	1	0.7012408882		
106.80903491	1	0.7012408882		
106.87474333	0	0.6976631286	0.662918516	0.7295827804
106.87474333	1	0.6976631286		
107.00616016	1	0.6976631286		
107.00616016	1	0.6976631286		
107.20328542	1	0.6976631286		
107.20328542	1	0.6976631286		
107.30184805	1	0.6976631286		
107.53182752	1	0.6976631286		
107.66324435	1	0.6976631286		
107.79466119	1	0.6976631286		
107.99178645	1	0.6976631286		
108.15605749	1	0.6976631286		
108.22176591	1	0.6976631286		
108.45174538	1	0.6976631286		
108.81314168	1	0.6976631286		
108.84599589	1	0.6976631286		
108.94455852	1	0.6976631286		
109.04312115	1	0.6976631286		
109.07597536	1	0.6976631286		
109.10882957	1	0.6976631286		
109.14168378	1	0.6976631286		
109.27310062	1	0.6976631286		
109.73305955	1	0.6976631286		
109.73305955	1	0.6976631286		
109.76591376	0	0.6936069476	0.6580871958	0.7262316597
109.83162218	1	0.6936069476		
109.99589322	1	0.6936069476		
110.19301848	1	0.6936069476		
110.52156057	1	0.6936069476		
110.55441478	1	0.6936069476		

110.55441478	1	0.6936069476		
110.68583162	1	0.6936069476		
110.91581109	1	0.6936069476		
110.91581109	1	0.6936069476		
110.9486653	1	0.6936069476		
111.14579055	1	0.6936069476		
111.17864476	1	0.6936069476		
111.27720739	0	0.6892446397	0.6528431334	0.7226691137
111.40862423	1	0.6892446397		
111.44147844	0	0.6848545465	0.6475937282	0.7190612095
111.44147844	1	0.6848545465		
111.44147844	1	0.6848545465		
111.67145791	1	0.6848545465		
111.80287474	1	0.6848545465		
111.86858316	1	0.6848545465		
111.90143737	1	0.6848545465		
111.93429158	1	0.6848545465		
111.93429158	1	0.6848545465		
112.13141684	1	0.6848545465		
112.13141684	1	0.6848545465		
112.59137577	1	0.6848545465		
112.59137577	1	0.6848545465		
112.62422998	1	0.6848545465		
112.85420945	1	0.6848545465		
112.85420945	1	0.6848545465		
112.95277207	1	0.6848545465		
113.08418891	1	0.6848545465		
113.11704312	1	0.6848545465		
113.21560575	1	0.6848545465		
113.51129363	1	0.6848545465		
113.54414784	1	0.6848545465		
113.54414784	1	0.6848545465		
113.67556468	1	0.6848545465		
113.93839836	1	0.6848545465		
114.03696099	0	0.6796662545	0.6411997258	0.7149588554
114.13552361	0	0.6744779625	0.634864737	0.7108082526
114.13552361	1			
114.16837782	1			
114.20123203	1			
114.46406571	1			
114.46406571	1			
114.66119097	1			
114.66119097	1			
114.82546201	1			
114.92402464	1			
115.02258727	1			
115.05544148	1			
115.25256674	1			
115.35112936	1			
115.38398357	1			

115.4825462	1			
115.84394251	1			
116.04106776	1			
116.23819302	1			
116.40246407	1			
116.43531828	1			
116.50102669	1			
116.5338809	1			
116.66529774	1			
116.66529774	1			
116.66529774	1			
116.89527721	1			
117.09240246	1			
117.38809035	1			
117.65092402	1			
117.81519507	1			
117.84804928	1			
117.88090349	1			
117.88090349	1			
117.9137577	1			
118.04517454	1			
118.07802875	1			
118.07802875	1			
118.11088296	1			
118.11088296	1			
118.14373717	1			
118.14373717	1			
118.275154	1			
118.34086242	1			
118.50513347	1			
118.50513347	1			
118.83367556	1			
118.9650924	1			
119.26078029	1			
119.42505133	1			
119.52361396	1			
119.75359343	1			
119.85215606	1			
119.85215606	1			
119.95071869	1			
119.9835729	1			
120.08213552	1			
120.11498973	1			
120.31211499	1			
120.41067762	1			
120.41067762	1			
120.44353183	1			
121.00205339	1			
121.0349076	1			
121.10061602	1			

121.13347023	1			
121.13347023	1			
121.13347023	1			
121.33059548	1			
121.75770021	1			
121.82340862	1			
122.02053388	1			
122.18480493	1			
122.41478439	1			
122.64476386	1			
122.71047228	1			
122.71047228	1			
122.74332649	1			
122.84188912	1			
122.84188912	1			
123.33470226	1			
123.33470226	1			
123.33470226	1			
123.40041068	1			
123.43326489	1			
123.56468172	1			
123.56468172	1			
123.76180698	1			
123.8275154	1			
123.89322382	1			
123.89322382	1			
124.09034908	1			
124.22176591	1			
124.25462012	1			
124.28747433	1			
124.48459959	1			
124.97741273	1			
125.37166324	1			
125.63449692	1			
125.73305955	1			
125.73305955	1			
125.73305955	1			
125.83162218	1			
126.12731006	1			
126.19301848	1			
126.55441478	1			
126.55441478	1			
126.58726899	1			
126.81724846	1			
126.88295688	1			
127.01437372	1			
127.11293634	1			
127.27720739	1			
127.34291581	1			
127.34291581	1			

127.54004107	1			
127.70431211	1			
127.70431211	1			
127.90143737	1			
127.93429158	1			
128.13141684	1			
128.13141684	1			
128.42710472	1			
128.45995893	1			
128.65708419	1			
128.82135524	1			
128.85420945	1			
129.0513347	1			
129.08418891	1			
129.14989733	1			
129.18275154	1			
