# Supplementary material for: Overall survival of triple negative breast cancer in French Caribbean women
Source: PLoS One. 2022 Aug 24;17(8):e0271966. doi: 10.1371/journal.pone.0271966 (PMC9401158; doi:10.1371/journal.pone.0271966)
Supplement: S1 Data — (ZIP) [file pone.0271966.s001.zip › survie_recept.pdf]

RECEPTOR	Time (month)	CENSOR	SURVIVAL	Survival LCL	Survival UCL	STRATUM
HR+/HER2+	0		1	1	1	1
HR+/HER2+	0	1	1			1
HR+/HER2+	0	1	1			1
HR+/HER2+	0	1	1			1
HR+/HER2+	0.3285420945	0	0.9957983193	0.970551401	0.9994070664	1
HR+/HER2+	0.59137577	1	0.9957983193			1
HR+/HER2+	0.7885010267	1	0.9957983193			1
HR+/HER2+	0.8213552361	0	0.9915608797	0.9666802766	0.9978827456	1
HR+/HER2+	1.0513347023	0	0.98732344	0.9612151677	0.99589398	1
HR+/HER2+	1.1498973306	0	0.9830860004	0.9555647896	0.9936182275	1
HR+/HER2+	1.2813141684	1	0.9830860004			1
HR+/HER2+	1.839835729	1	0.9830860004			1
HR+/HER2+	2.8583162218	1	0.9830860004			1
HR+/HER2+	3.318275154	0	0.9787930484	0.9498017779	0.991118438	1
HR+/HER2+	3.9753593429	1	0.9787930484			1
HR+/HER2+	4.3367556468	1	0.9787930484			1
HR+/HER2+	5.9794661191	0	0.9744621057	0.9440373963	0.9884464166	1
HR+/HER2+	10.184804928	1	0.9744621057			1
HR+/HER2+	10.513347023	1	0.9744621057			1
HR+/HER2+	10.710472279	1	0.9744621057			1
HR+/HER2+	10.710472279	1	0.9744621057			1
HR+/HER2+	10.776180698	1	0.9744621057			1
HR+/HER2+	10.940451745	1	0.9744621057			1
HR+/HER2+	11.531827515	1	0.9744621057			1
HR+/HER2+	11.564681725	1	0.9744621057			1
HR+/HER2+	11.86036961	1	0.9744621057			1
HR+/HER2+	12.320328542	1	0.9744621057			1
HR+/HER2+	12.353182752	1	0.9744621057			1
HR+/HER2+	12.353182752	1	0.9744621057			1
HR+/HER2+	12.517453799	1	0.9744621057			1
HR+/HER2+	12.583162218	1	0.9744621057			1
HR+/HER2+	13.437371663	1	0.9744621057			1
HR+/HER2+	13.503080082	0	0.96982181	0.937709524	0.9855062303	1
HR+/HER2+	14.488706366	1	0.96982181			1
HR+/HER2+	14.587268994	1	0.96982181			1
HR+/HER2+	14.784394251	1	0.96982181			1
HR+/HER2+	15.277207392	1	0.96982181			1
HR+/HER2+	16.164271047	1	0.96982181			1
HR+/HER2+	16.229979466	1	0.96982181			1
HR+/HER2+	16.854209446	1	0.96982181			1
HR+/HER2+	16.952772074	1	0.96982181			1
HR+/HER2+	17.347022587	1	0.96982181			1
HR+/HER2+	17.741273101	0	0.9649727009	0.931103018	0.9823484977	1
HR+/HER2+	17.80698152	1	0.9649727009			1
HR+/HER2+	17.905544148	0	0.9600991014	0.9246110224	0.9790691477	1
HR+/HER2+	17.905544148	1	0.9600991014			1
HR+/HER2+	18.39835729	1	0.9600991014			1
HR+/HER2+	18.496919918	1	0.9600991014			1
HR+/HER2+	18.661190965	1	0.9600991014			1

HR+/HER2+	19.712525667	1	0.9600991014			1
HR+/HER2+	19.745379877	0	0.9550985853	0.9180071937	0.9756314828	1
HR+/HER2+	19.843942505	1	0.9550985853			1
HR+/HER2+	19.942505133	1	0.9550985853			1
HR+/HER2+	20.501026694	1	0.9550985853			1
HR+/HER2+	20.566735113	0	0.9500182736	0.9113801362	0.9720660518	1
HR+/HER2+	20.599589322	0	0.944937962	0.9048667394	0.9684225531	1
HR+/HER2+	20.632443532	1	0.944937962			1
HR+/HER2+	20.698151951	1	0.944937962			1
HR+/HER2+	21.388090349	1	0.944937962			1
HR+/HER2+	21.420944559	1	0.944937962			1
HR+/HER2+	21.453798768	1	0.944937962			1
HR+/HER2+	23.425051335	1	0.944937962			1
HR+/HER2+	24.344969199	1	0.944937962			1
HR+/HER2+	24.574948665	1	0.944937962			1
HR+/HER2+	25.297741273	1	0.944937962			1
HR+/HER2+	25.889117043	1	0.944937962			1
HR+/HER2+	25.954825462	1	0.944937962			1
HR+/HER2+	25.987679671	0	0.9395383165	0.8978777124	0.9645361343	1
HR+/HER2+	26.414784394	1	0.9395383165			1
HR+/HER2+	27.466119097	1	0.9395383165			1
HR+/HER2+	27.564681725	0	0.9340758844	0.890892316	0.9605429472	1
HR+/HER2+	27.86036961	1	0.9340758844			1
HR+/HER2+	28.123203285	1	0.9340758844			1
HR+/HER2+	28.221765914	0	0.9285488082	0.8838981424	0.9564468907	1
HR+/HER2+	28.484599589	0	0.923021732	0.8769978949	0.9522890359	1
HR+/HER2+	31.474332649	0	0.9174946557	0.8701790619	0.9480752325	1
HR+/HER2+	32.952772074	0	0.9119675795	0.8634317933	0.9438103929	1
HR+/HER2+	33.412731006	1	0.9119675795			1
HR+/HER2+	33.905544148	1	0.9119675795			1
HR+/HER2+	34.332648871	1	0.9119675795			1
HR+/HER2+	34.694045175	0	0.90633815	0.8565844914	0.9394363473	1
HR+/HER2+	35.186858316	1	0.90633815			1
HR+/HER2+	35.515400411	1	0.90633815			1
HR+/HER2+	35.515400411	1	0.90633815			1
HR+/HER2+	35.942505133	0	0.9006018326	0.8496308268	0.9349510468	1
HR+/HER2+	36.1724846	1	0.9006018326			1
HR+/HER2+	36.205338809	1	0.9006018326			1
HR+/HER2+	36.402464066	1	0.9006018326			1
HR+/HER2+	36.402464066	1	0.9006018326			1
HR+/HER2+	36.435318275	0	0.8947155461	0.8425036958	0.9303285161	1
HR+/HER2+	37.190965092	1	0.8947155461			1
HR+/HER2+	37.519507187	1	0.8947155461			1
HR+/HER2+	37.782340862	1	0.8947155461			1
HR+/HER2+	37.848049281	1	0.8947155461			1
HR+/HER2+	37.880903491	1	0.8947155461			1
HR+/HER2+	38.50513347	1	0.8947155461			1
HR+/HER2+	38.899383984	1	0.8947155461			1
HR+/HER2+	39.753593429	0	0.888545094	0.8349936996	0.9254862463	1
HR+/HER2+	39.852156057	1	0.888545094			1

HR+/HER2+	40.772073922	1	0.888545094			1
HR+/HER2+	41.297741273	1	0.888545094			1
HR+/HER2+	41.527720739	1	0.888545094			1
HR+/HER2+	41.691991786	1	0.888545094			1
HR+/HER2+	41.691991786	1	0.888545094			1
HR+/HER2+	41.790554415	1	0.888545094			1
HR+/HER2+	42.217659138	1	0.888545094			1
HR+/HER2+	42.447638604	0	0.8820116742	0.8269822945	0.9203747713	1
HR+/HER2+	42.64476386	1	0.8820116742			1
HR+/HER2+	42.940451745	1	0.8820116742			1
HR+/HER2+	43.006160164	1	0.8820116742			1
HR+/HER2+	43.26899384	0	0.8753297676	0.8188227735	0.9151195527	1
HR+/HER2+	43.597535934	1	0.8753297676			1
HR+/HER2+	44.090349076	1	0.8753297676			1
HR+/HER2+	44.911704312	1	0.8753297676			1
HR+/HER2+	44.977412731	1	0.8753297676			1
HR+/HER2+	45.01026694	1	0.8753297676			1
HR+/HER2+	45.174537988	1	0.8753297676			1
HR+/HER2+	45.207392197	1	0.8753297676			1
HR+/HER2+	45.568788501	1	0.8753297676			1
HR+/HER2+	46.127310062	1	0.8753297676			1
HR+/HER2+	46.258726899	0	0.8681549335	0.8099661662	0.909514138	1
HR+/HER2+	46.291581109	1	0.8681549335			1
HR+/HER2+	46.291581109	1	0.8681549335			1
HR+/HER2+	46.718685832	1	0.8681549335			1
HR+/HER2+	47.014373717	1	0.8681549335			1
HR+/HER2+	47.014373717	1	0.8681549335			1
HR+/HER2+	47.211498973	1	0.8681549335			1
HR+/HER2+	47.474332649	1	0.8681549335			1
HR+/HER2+	47.835728953	0	0.8605395393	0.8005094319	0.9035855555	1
HR+/HER2+	47.967145791	1	0.8605395393			1
HR+/HER2+	48.164271047	1	0.8605395393			1
HR+/HER2+	48.229979466	1	0.8605395393			1
HR+/HER2+	49.248459959	1	0.8605395393			1
HR+/HER2+	50.266940452	1	0.8605395393			1
HR+/HER2+	51.449691992	1	0.8605395393			1
HR+/HER2+	51.58110883	0	0.8524971137	0.7904874916	0.8973369828	1
HR+/HER2+	52.271047228	1	0.8524971137			1
HR+/HER2+	52.533880903	0	0.8443780936	0.7804821368	0.8909656268	1
HR+/HER2+	54.110882957	1	0.8443780936			1
HR+/HER2+	55.950718686	1	0.8443780936			1
HR+/HER2+	56.016427105	1	0.8443780936			1
HR+/HER2+	56.213552361	1	0.8443780936			1
HR+/HER2+	57.100616016	1	0.8443780936			1
HR+/HER2+	57.297741273	1	0.8443780936			1
HR+/HER2+	57.429158111	1	0.8443780936			1
HR+/HER2+	57.560574949	0	0.8356731648	0.7696672565	0.884177292	1
HR+/HER2+	58.0862423	1	0.8356731648			1
HR+/HER2+	58.119096509	1	0.8356731648			1
HR+/HER2+	58.316221766	1	0.8356731648			1

HR+/HER2+	58.447638604	1	0.8356731648			1
HR+/HER2+	60.188911704	1	0.8356731648			1
HR+/HER2+	60.813141684	1	0.8356731648			1
HR+/HER2+	61.733059548	1	0.8356731648			1
HR+/HER2+	61.963039014	1	0.8356731648			1
HR+/HER2+	62.620123203	1	0.8356731648			1
HR+/HER2+	62.718685832	1	0.8356731648			1
HR+/HER2+	62.915811088	1	0.8356731648			1
HR+/HER2+	64.229979466	1	0.8356731648			1
HR+/HER2+	65.642710472	1	0.8356731648			1
HR+/HER2+	65.708418891	1	0.8356731648			1
HR+/HER2+	66.529774127	1	0.8356731648			1
HR+/HER2+	66.759753593	1	0.8356731648			1
HR+/HER2+	66.759753593	1	0.8356731648			1
HR+/HER2+	67.022587269	1	0.8356731648			1
HR+/HER2+	67.318275154	1	0.8356731648			1
HR+/HER2+	67.54825462	1	0.8356731648			1
HR+/HER2+	67.646817248	1	0.8356731648			1
HR+/HER2+	67.646817248	1	0.8356731648			1
HR+/HER2+	68.13963039	1	0.8356731648			1
HR+/HER2+	68.238193018	1	0.8356731648			1
HR+/HER2+	70.275154004	1	0.8356731648			1
HR+/HER2+	70.669404517	1	0.8356731648			1
HR+/HER2+	72.016427105	1	0.8356731648			1
HR+/HER2+	72.27926078	1	0.8356731648			1
HR+/HER2+	72.574948665	1	0.8356731648			1
HR+/HER2+	73.724845996	1	0.8356731648			1
HR+/HER2+	73.856262834	1	0.8356731648			1
HR+/HER2+	74.874743326	0	0.8228166545	0.7517406398	0.8752192846	1
HR+/HER2+	76.221765914	1	0.8228166545			1
HR+/HER2+	76.254620123	1	0.8228166545			1
HR+/HER2+	77.305954825	1	0.8228166545			1
HR+/HER2+	77.60164271	1	0.8228166545			1
HR+/HER2+	78.291581109	1	0.8228166545			1
HR+/HER2+	78.81724846	1	0.8228166545			1
HR+/HER2+	78.948665298	1	0.8228166545			1
HR+/HER2+	79.014373717	1	0.8228166545			1
HR+/HER2+	79.047227926	1	0.8228166545			1
HR+/HER2+	79.704312115	0	0.8078563517	0.7304647682	0.8650596925	1
HR+/HER2+	81.018480493	1	0.8078563517			1
HR+/HER2+	81.77412731	1	0.8078563517			1
HR+/HER2+	81.971252567	1	0.8078563517			1
HR+/HER2+	82.464065708	1	0.8078563517			1
HR+/HER2+	83.515400411	1	0.8078563517			1
HR+/HER2+	83.58110883	1	0.8078563517			1
HR+/HER2+	83.811088296	1	0.8078563517			1
HR+/HER2+	83.843942505	1	0.8078563517			1
HR+/HER2+	84.106776181	0	0.7902942571	0.7050452806	0.8534353998	1
HR+/HER2+	85.125256674	1	0.7902942571			1
HR+/HER2+	85.486652977	1	0.7902942571			1

HR+/HER2+	88.410677618	1	0.7902942571			1
HR+/HER2+	90.184804928	0	0.7714777272	0.678468303	0.8406969869	1
HR+/HER2+	90.349075975	0	0.7526611973	0.6531483024	0.8273380193	1
HR+/HER2+	91.23613963	1	0.7526611973			1
HR+/HER2+	92.254620123	1	0.7526611973			1
HR+/HER2+	92.648870637	0	0.7328543237	0.6270607455	0.8130327725	1
HR+/HER2+	93.207392197	1	0.7328543237			1
HR+/HER2+	93.273100616	1	0.7328543237			1
HR+/HER2+	94.160164271	1	0.7328543237			1
HR+/HER2+	95.112936345	1	0.7328543237			1
HR+/HER2+	95.802874743	1	0.7328543237			1
HR+/HER2+	95.802874743	1	0.7328543237			1
HR+/HER2+	96.032854209	1	0.7328543237			1
HR+/HER2+	96.229979466	0	0.7084258462	0.593584589	0.7962682384	1
HR+/HER2+	96.295687885	1	0.7084258462			1
HR+/HER2+	96.854209446	1	0.7084258462			1
HR+/HER2+	100.40246407	1	0.7084258462			1
HR+/HER2+	100.82956879	1	0.7084258462			1
HR+/HER2+	100.862423	1	0.7084258462			1
HR+/HER2+	101.12525667	1	0.7084258462			1
HR+/HER2+	106.38193018	1	0.7084258462			1
HR+/HER2+	106.61190965	1	0.7084258462			1
HR+/HER2+	108.84599589	1	0.7084258462			1
HR+/HER2+	108.94455852	1	0.7084258462			1
HR+/HER2+	109.99589322	1	0.7084258462			1
HR+/HER2+	110.19301848	1	0.7084258462			1
HR+/HER2+	110.9486653	1	0.7084258462			1
HR+/HER2+	111.27720739	0	0.6641492308	0.5208717283	0.7735394075	1
HR+/HER2+	112.95277207	1				1
HR+/HER2+	113.54414784	1				1
HR+/HER2+	114.66119097	1				1
HR+/HER2+	114.66119097	1				1
HR+/HER2+	123.40041068	1				1
HR+/HER2+	123.56468172	1				1
HR+/HER2+	123.8275154	1				1
HR+/HER2+	124.09034908	1				1
HR+/HER2+	124.22176591	1				1
HR+/HER2+	125.63449692	1				1
HR+/HER2+	126.12731006	1				1
HR+/HER2+	127.27720739	1				1
HR+/HER2+	127.34291581	1				1
HR+/HER2+	128.65708419	1				1
HR+/HER2+	129.14989733	1				1
HR+/HER2-	0		1	1	1	2
HR+/HER2-	0	1	1			2
HR+/HER2-	0	1	1			2
HR+/HER2-	0	1	1			2
HR+/HER2-	0.2628336756	0	0.9988465975	0.9918406561	0.9998374471	2
HR+/HER2-	0.3613963039	1	0.9988465975			2
HR+/HER2-	0.3942505133	1	0.9988465975			2

HR+/HER2-	0.4599589322	1	0.9988465975			2
HR+/HER2-	0.4599589322	1	0.9988465975			2
HR+/HER2-	0.6899383984	1	0.9988465975			2
HR+/HER2-	0.6899383984	1	0.9988465975			2
HR+/HER2-	0.7227926078	0	0.9976851479	0.9907761743	0.9994205644	2
HR+/HER2-	0.7885010267	1	0.9976851479			2
HR+/HER2-	0.887063655	0	0.9953595415	0.9876834994	0.9982558399	2
HR+/HER2-	1.0841889117	1	0.9953595415			2
HR+/HER2-	1.0841889117	1	0.9953595415			2
HR+/HER2-	1.4127310062	1	0.9953595415			2
HR+/HER2-	1.6098562628	0	0.9941926487	0.9861042647	0.9975787394	2
HR+/HER2-	1.7741273101	0	0.993025756	0.9845422985	0.9968607487	2
HR+/HER2-	2.0041067762	0	0.9918588632	0.9829991528	0.9961105948	2
HR+/HER2-	2.0698151951	1	0.9918588632			2
HR+/HER2-	2.4640657084	1	0.9918588632			2
HR+/HER2-	2.5297741273	1	0.9918588632			2
HR+/HER2-	2.5626283368	0	0.9906878374	0.9814656823	0.9953321938	2
HR+/HER2-	2.6283367556	1	0.9906878374			2
HR+/HER2-	2.6611909651	1	0.9906878374			2
HR+/HER2-	3.022587269	1	0.9906878374			2
HR+/HER2-	3.2525667351	0	0.9895126442	0.9799412644	0.9945295893	2
HR+/HER2-	3.7125256674	1	0.9895126442			2
HR+/HER2-	4.1396303901	1	0.9895126442			2
HR+/HER2-	4.8952772074	0	0.9871566617	0.9769281546	0.9928670524	2
HR+/HER2-	5.158110883	1	0.9871566617			2
HR+/HER2-	5.4209445585	0	0.9859772631	0.9754392634	0.9920123124	2
HR+/HER2-	5.6837782341	0	0.9847978644	0.9739621121	0.9911447372	2
HR+/HER2-	5.8151950719	1	0.9847978644			2
HR+/HER2-	5.8809034908	1	0.9847978644			2
HR+/HER2-	6.0780287474	1	0.9847978644			2
HR+/HER2-	6.4394250513	0	0.9836142132	0.9724884179	0.9902631673	2
HR+/HER2-	6.4722792608	1	0.9836142132			2
HR+/HER2-	6.5708418891	1	0.9836142132			2
HR+/HER2-	6.7679671458	0	0.9824277063	0.9710199247	0.9893694324	2
HR+/HER2-	6.932238193	0	0.9812411994	0.9695606538	0.9884661531	2
HR+/HER2-	6.9650924025	1	0.9812411994			2
HR+/HER2-	7.2936344969	1	0.9812411994			2
HR+/HER2-	7.3264887064	1	0.9812411994			2
HR+/HER2-	7.3264887064	1	0.9812411994			2
HR+/HER2-	7.5893223819	1	0.9812411994			2
HR+/HER2-	8.0492813142	1	0.9812411994			2
HR+/HER2-	8.0492813142	1	0.9812411994			2
HR+/HER2-	8.5749486653	1	0.9812411994			2
HR+/HER2-	8.6078028747	1	0.9812411994			2
HR+/HER2-	9.2320328542	1	0.9812411994			2
HR+/HER2-	9.7577002053	1	0.9812411994			2
HR+/HER2-	9.8234086242	1	0.9812411994			2
HR+/HER2-	9.9219712526	1	0.9812411994			2
HR+/HER2-	9.954825462	1	0.9812411994			2
HR+/HER2-	10.020533881	1	0.9812411994			2

HR+/HER2-	10.05338809	1	0.9812411994			2
HR+/HER2-	10.184804928	1	0.9812411994			2
HR+/HER2-	10.349075975	1	0.9812411994			2
HR+/HER2-	10.480492813	1	0.9812411994			2
HR+/HER2-	10.480492813	1	0.9812411994			2
HR+/HER2-	10.480492813	1	0.9812411994			2
HR+/HER2-	10.513347023	1	0.9812411994			2
HR+/HER2-	10.513347023	1	0.9812411994			2
HR+/HER2-	10.710472279	1	0.9812411994			2
HR+/HER2-	10.743326489	1	0.9812411994			2
HR+/HER2-	10.743326489	1	0.9812411994			2
HR+/HER2-	10.907597536	0	0.9800161792	0.9680475776	0.9875304493	2
HR+/HER2-	10.907597536	1	0.9800161792			2
HR+/HER2-	10.940451745	1	0.9800161792			2
HR+/HER2-	11.071868583	1	0.9800161792			2
HR+/HER2-	11.071868583	1	0.9800161792			2
HR+/HER2-	11.071868583	1	0.9800161792			2
HR+/HER2-	11.104722793	1	0.9800161792			2
HR+/HER2-	11.170431211	1	0.9800161792			2
HR+/HER2-	11.170431211	1	0.9800161792			2
HR+/HER2-	11.433264887	1	0.9800161792			2
HR+/HER2-	11.498973306	1	0.9800161792			2
HR+/HER2-	11.728952772	0	0.9787756523	0.9665191837	0.9865764387	2
HR+/HER2-	11.761806982	1	0.9787756523			2
HR+/HER2-	11.794661191	1	0.9787756523			2
HR+/HER2-	11.893223819	1	0.9787756523			2
HR+/HER2-	12.024640657	1	0.9787756523			2
HR+/HER2-	12.221765914	0	0.9775288044	0.964990215	0.9856101759	2
HR+/HER2-	12.254620123	1	0.9775288044			2
HR+/HER2-	12.353182752	1	0.9775288044			2
HR+/HER2-	12.583162218	1	0.9775288044			2
HR+/HER2-	12.681724846	1	0.9775288044			2
HR+/HER2-	12.714579055	1	0.9775288044			2
HR+/HER2-	12.813141684	1	0.9775288044			2
HR+/HER2-	13.01026694	1	0.9775288044			2
HR+/HER2-	13.01026694	1	0.9775288044			2
HR+/HER2-	13.04312115	1	0.9775288044			2
HR+/HER2-	13.108829569	1	0.9775288044			2
HR+/HER2-	13.207392197	1	0.9775288044			2
HR+/HER2-	13.273100616	1	0.9775288044			2
HR+/HER2-	13.273100616	1	0.9775288044			2
HR+/HER2-	13.700205339	1	0.9775288044			2
HR+/HER2-	13.733059548	1	0.9775288044			2
HR+/HER2-	13.733059548	1	0.9775288044			2
HR+/HER2-	13.765913758	0	0.9762559804	0.9634291618	0.9846197782	2
HR+/HER2-	13.995893224	0	0.9749831565	0.9618773476	0.9836217403	2
HR+/HER2-	14.028747433	1	0.9749831565			2
HR+/HER2-	14.061601643	0	0.9737086687	0.9603314173	0.9826155346	2
HR+/HER2-	14.061601643	1	0.9737086687			2
HR+/HER2-	14.094455852	1	0.9737086687			2

HR+/HER2-	14.094455852	1	0.9737086687			2
HR+/HER2-	14.291581109	1	0.9737086687			2
HR+/HER2-	14.291581109	1	0.9737086687			2
HR+/HER2-	14.324435318	1	0.9737086687			2
HR+/HER2-	14.324435318	1	0.9737086687			2
HR+/HER2-	14.422997947	1	0.9737086687			2
HR+/HER2-	14.488706366	1	0.9737086687			2
HR+/HER2-	14.488706366	1	0.9737086687			2
HR+/HER2-	14.718685832	1	0.9737086687			2
HR+/HER2-	14.751540041	1	0.9737086687			2
HR+/HER2-	14.784394251	1	0.9737086687			2
HR+/HER2-	14.850102669	1	0.9737086687			2
HR+/HER2-	14.981519507	0	0.9724103904	0.9587567869	0.981587289	2
HR+/HER2-	15.277207392	1	0.9724103904			2
HR+/HER2-	15.277207392	1	0.9724103904			2
HR+/HER2-	15.342915811	1	0.9724103904			2
HR+/HER2-	15.375770021	1	0.9724103904			2
HR+/HER2-	15.441478439	0	0.9711051416	0.9571795596	0.9805479485	2
HR+/HER2-	15.441478439	1	0.9711051416			2
HR+/HER2-	15.540041068	1	0.9711051416			2
HR+/HER2-	15.770020534	1	0.9711051416			2
HR+/HER2-	15.868583162	0	0.9697946083	0.9556019896	0.979498898	2
HR+/HER2-	15.868583162	1	0.9697946083			2
HR+/HER2-	15.934291581	1	0.9697946083			2
HR+/HER2-	15.967145791	1	0.9697946083			2
HR+/HER2-	16.229979466	1	0.9697946083			2
HR+/HER2-	16.229979466	1	0.9697946083			2
HR+/HER2-	16.295687885	1	0.9697946083			2
HR+/HER2-	16.328542094	1	0.9697946083			2
HR+/HER2-	16.328542094	1	0.9697946083			2
HR+/HER2-	16.361396304	1	0.9697946083			2
HR+/HER2-	16.361396304	1	0.9697946083			2
HR+/HER2-	16.459958932	0	0.9684661226	0.9540046682	0.9784320577	2
HR+/HER2-	16.459958932	1	0.9684661226			2
HR+/HER2-	16.492813142	1	0.9684661226			2
HR+/HER2-	16.492813142	1	0.9684661226			2
HR+/HER2-	16.492813142	1	0.9684661226			2
HR+/HER2-	16.492813142	1	0.9684661226			2
HR+/HER2-	16.525667351	0	0.9671284622	0.95240088	0.9773533094	2
HR+/HER2-	16.525667351	1	0.9671284622			2
HR+/HER2-	16.657084189	1	0.9671284622			2
HR+/HER2-	16.689938398	1	0.9671284622			2
HR+/HER2-	16.755646817	0	0.9657852282	0.9507958171	0.9762652369	2
HR+/HER2-	16.854209446	1	0.9657852282			2
HR+/HER2-	16.854209446	1	0.9657852282			2
HR+/HER2-	16.887063655	1	0.9657852282			2
HR+/HER2-	16.952772074	1	0.9657852282			2
HR+/HER2-	17.084188912	1	0.9657852282			2
HR+/HER2-	17.084188912	1	0.9657852282			2
HR+/HER2-	17.084188912	1	0.9657852282			2

HR+/HER2-	17.149897331	1	0.9657852282			2
HR+/HER2-	17.248459959	1	0.9657852282			2
HR+/HER2-	17.445585216	1	0.9657852282			2
HR+/HER2-	17.478439425	1	0.9657852282			2
HR+/HER2-	17.577002053	1	0.9657852282			2
HR+/HER2-	17.642710472	1	0.9657852282			2
HR+/HER2-	17.839835729	1	0.9657852282			2
HR+/HER2-	17.839835729	1	0.9657852282			2
HR+/HER2-	17.872689938	1	0.9657852282			2
HR+/HER2-	18.004106776	1	0.9657852282			2
HR+/HER2-	18.036960986	1	0.9657852282			2
HR+/HER2-	18.036960986	1	0.9657852282			2
HR+/HER2-	18.036960986	1	0.9657852282			2
HR+/HER2-	18.069815195	1	0.9657852282			2
HR+/HER2-	18.102669405	1	0.9657852282			2
HR+/HER2-	18.201232033	1	0.9657852282			2
HR+/HER2-	18.234086242	0	0.9643976058	0.9491315884	0.9751420963	2
HR+/HER2-	18.234086242	1	0.9643976058			2
HR+/HER2-	18.299794661	1	0.9643976058			2
HR+/HER2-	18.36550308	0	0.9630059786	0.9474688173	0.9740106716	2
HR+/HER2-	18.36550308	1	0.9630059786			2
HR+/HER2-	18.529774127	1	0.9630059786			2
HR+/HER2-	18.661190965	1	0.9630059786			2
HR+/HER2-	18.694045175	1	0.9630059786			2
HR+/HER2-	18.694045175	1	0.9630059786			2
HR+/HER2-	18.726899384	1	0.9630059786			2
HR+/HER2-	18.825462012	0	0.9616021798	0.9457951284	0.9728656906	2
HR+/HER2-	18.858316222	0	0.960198381	0.9441282653	0.9717155052	2
HR+/HER2-	18.858316222	1	0.960198381			2
HR+/HER2-	18.924024641	1	0.960198381			2
HR+/HER2-	19.022587269	1	0.960198381			2
HR+/HER2-	19.022587269	1	0.960198381			2
HR+/HER2-	19.121149897	0	0.9587863246	0.9424558041	0.970554735	2
HR+/HER2-	19.154004107	0	0.9573742681	0.9407895264	0.9693891802	2
HR+/HER2-	19.154004107	1	0.9573742681			2
HR+/HER2-	19.186858316	0	0.9559601259	0.939126077	0.9682176248	2
HR+/HER2-	19.186858316	1	0.9559601259			2
HR+/HER2-	19.219712526	1	0.9559601259			2
HR+/HER2-	19.252566735	1	0.9559601259			2
HR+/HER2-	19.351129363	1	0.9559601259			2
HR+/HER2-	19.351129363	1	0.9559601259			2
HR+/HER2-	19.383983573	1	0.9559601259			2
HR+/HER2-	19.416837782	1	0.9559601259			2
HR+/HER2-	19.482546201	1	0.9559601259			2
HR+/HER2-	19.482546201	1	0.9559601259			2
HR+/HER2-	19.54825462	0	0.9545269024	0.9374409142	0.9670284352	2
HR+/HER2-	19.646817248	1	0.9545269024			2
HR+/HER2-	19.843942505	1	0.9545269024			2
HR+/HER2-	20.041067762	1	0.9545269024			2
HR+/HER2-	20.1724846	1	0.9545269024			2

HR+/HER2-	20.271047228	1	0.9545269024			2
HR+/HER2-	20.271047228	1	0.9545269024			2
HR+/HER2-	20.271047228	1	0.9545269024			2
HR+/HER2-	20.303901437	1	0.9545269024			2
HR+/HER2-	20.336755647	0	0.9530762536	0.9357365129	0.9658227553	2
HR+/HER2-	20.501026694	0	0.9516256048	0.9340377192	0.9646127518	2
HR+/HER2-	20.599589322	1	0.9516256048			2
HR+/HER2-	20.632443532	0	0.9501727413	0.9323411073	0.9633970543	2
HR+/HER2-	20.632443532	1	0.9501727413			2
HR+/HER2-	20.862422998	0	0.9487176528	0.9306464424	0.9621758023	2
HR+/HER2-	20.862422998	1	0.9487176528			2
HR+/HER2-	20.895277207	1	0.9487176528			2
HR+/HER2-	20.928131417	0	0.9472580872	0.9289503767	0.960947531	2
HR+/HER2-	20.993839836	1	0.9472580872			2
HR+/HER2-	21.158110883	1	0.9472580872			2
HR+/HER2-	21.158110883	1	0.9472580872			2
HR+/HER2-	21.190965092	0	0.9457917434	0.9272495711	0.9597107007	2
HR+/HER2-	21.190965092	1	0.9457917434			2
HR+/HER2-	21.256673511	1	0.9457917434			2
HR+/HER2-	21.256673511	1	0.9457917434			2
HR+/HER2-	21.256673511	1	0.9457917434			2
HR+/HER2-	21.420944559	0	0.9443162493	0.9255406514	0.9584636861	2
HR+/HER2-	21.486652977	0	0.9428407551	0.9238362197	0.9572131092	2
HR+/HER2-	21.585215606	1	0.9428407551			2
HR+/HER2-	21.650924025	1	0.9428407551			2
HR+/HER2-	21.683778234	1	0.9428407551			2
HR+/HER2-	21.683778234	1	0.9428407551			2
HR+/HER2-	21.880903491	1	0.9428407551			2
HR+/HER2-	21.880903491	1	0.9428407551			2
HR+/HER2-	21.880903491	1	0.9428407551			2
HR+/HER2-	21.9137577	1	0.9428407551			2
HR+/HER2-	21.9137577	1	0.9428407551			2
HR+/HER2-	21.9137577	1	0.9428407551			2
HR+/HER2-	21.979466119	1	0.9428407551			2
HR+/HER2-	22.078028747	1	0.9428407551			2
HR+/HER2-	22.143737166	1	0.9428407551			2
HR+/HER2-	22.308008214	1	0.9428407551			2
HR+/HER2-	22.308008214	1	0.9428407551			2
HR+/HER2-	22.308008214	1	0.9428407551			2
HR+/HER2-	22.340862423	1	0.9428407551			2
HR+/HER2-	22.373716632	1	0.9428407551			2
HR+/HER2-	22.439425051	1	0.9428407551			2
HR+/HER2-	22.439425051	1	0.9428407551			2
HR+/HER2-	22.570841889	1	0.9428407551			2
HR+/HER2-	22.603696099	1	0.9428407551			2
HR+/HER2-	22.603696099	1	0.9428407551			2
HR+/HER2-	22.669404517	1	0.9428407551			2
HR+/HER2-	22.767967146	1	0.9428407551			2
HR+/HER2-	22.800821355	0	0.9413051839	0.9220530391	0.9559159786	2
HR+/HER2-	22.899383984	0	0.9397696126	0.9202748144	0.95461508	2

HR+/HER2-	23.12936345	1	0.9397696126			2
HR+/HER2-	23.162217659	0	0.9382315282	0.9184978529	0.9533087458	2
HR+/HER2-	23.227926078	0	0.9366934437	0.916725437	0.9519988997	2
HR+/HER2-	23.359342916	1	0.9366934437			2
HR+/HER2-	23.392197125	0	0.9351528295	0.9149539138	0.9506838287	2
HR+/HER2-	23.490759754	0	0.9336122152	0.9131865838	0.9493654712	2
HR+/HER2-	23.523613963	1	0.9336122152			2
HR+/HER2-	23.655030801	1	0.9336122152			2
HR+/HER2-	23.917864476	0	0.9320664997	0.9114163656	0.948040201	2
HR+/HER2-	24.246406571	1	0.9320664997			2
HR+/HER2-	24.443531828	1	0.9320664997			2
HR+/HER2-	24.542094456	1	0.9320664997			2
HR+/HER2-	24.640657084	0	0.9305130555	0.9096396313	0.9467061479	2
HR+/HER2-	25.297741273	0	0.9289596113	0.9078667508	0.945369041	2
HR+/HER2-	25.297741273	1	0.9289596113			2
HR+/HER2-	25.49486653	0	0.9274035651	0.9060940862	0.944027052	2
HR+/HER2-	25.527720739	1	0.9274035651			2
HR+/HER2-	25.560574949	1	0.9274035651			2
HR+/HER2-	25.626283368	1	0.9274035651			2
HR+/HER2-	25.823408624	0	0.9258396468	0.9043144909	0.9426763403	2
HR+/HER2-	25.856262834	1	0.9258396468			2
HR+/HER2-	25.889117043	0	0.9242730822	0.9025349123	0.9413208187	2
HR+/HER2-	26.217659138	0	0.9227065177	0.9007587775	0.939962526	2
HR+/HER2-	26.447638604	0	0.9211399531	0.8989859649	0.9386015435	2
HR+/HER2-	27.071868583	1	0.9211399531			2
HR+/HER2-	27.301848049	0	0.9195707198	0.8972128376	0.9372359418	2
HR+/HER2-	27.531827515	1	0.9195707198			2
HR+/HER2-	28.123203285	1	0.9195707198			2
HR+/HER2-	28.45174538	1	0.9195707198			2
HR+/HER2-	28.484599589	0	0.9179934115	0.8954322018	0.9358617013	2
HR+/HER2-	28.714579055	0	0.9164161033	0.8936546624	0.9344849336	2
HR+/HER2-	28.878850103	0	0.914838795	0.8918801189	0.9331057073	2
HR+/HER2-	29.207392197	0	0.9132614867	0.8901084768	0.9317240877	2
HR+/HER2-	29.404517454	1	0.9132614867			2
HR+/HER2-	29.535934292	1	0.9132614867			2
HR+/HER2-	29.60164271	1	0.9132614867			2
HR+/HER2-	29.897330595	1	0.9132614867			2
HR+/HER2-	30.751540041	1	0.9132614867			2
HR+/HER2-	30.850102669	0	0.9116704388	0.888321742	0.9303296737	2
HR+/HER2-	32.098562628	0	0.9084883431	0.8847567068	0.9275338862	2
HR+/HER2-	32.394250513	0	0.9068972952	0.8829782401	0.9261326285	2
HR+/HER2-	32.755646817	1	0.9068972952			2
HR+/HER2-	33.741273101	1	0.9068972952			2
HR+/HER2-	33.872689938	1	0.9068972952			2
HR+/HER2-	33.938398357	1	0.9068972952			2
HR+/HER2-	33.938398357	1	0.9068972952			2
HR+/HER2-	34.135523614	1	0.9068972952			2
HR+/HER2-	34.135523614	1	0.9068972952			2
HR+/HER2-	34.39835729	1	0.9068972952			2
HR+/HER2-	34.39835729	1	0.9068972952			2

HR+/HER2-	34.496919918	1	0.9068972952			2
HR+/HER2-	34.562628337	1	0.9068972952			2
HR+/HER2-	34.562628337	1	0.9068972952			2
HR+/HER2-	34.595482546	0	0.9052720312	0.8811581059	0.9247029583	2
HR+/HER2-	34.759753593	1	0.9052720312			2
HR+/HER2-	34.891170431	0	0.9036438441	0.8793369747	0.9232687699	2
HR+/HER2-	35.022587269	1	0.9036438441			2
HR+/HER2-	35.219712526	0	0.902012718	0.8775147763	0.9218300874	2
HR+/HER2-	35.219712526	1	0.902012718			2
HR+/HER2-	35.252566735	1	0.902012718			2
HR+/HER2-	35.515400411	0	0.9003756714	0.875687621	0.920384651	2
HR+/HER2-	35.515400411	1	0.9003756714			2
HR+/HER2-	35.515400411	1	0.9003756714			2
HR+/HER2-	35.515400411	1	0.9003756714			2
HR+/HER2-	35.58110883	1	0.9003756714			2
HR+/HER2-	35.679671458	1	0.9003756714			2
HR+/HER2-	35.712525667	1	0.9003756714			2
HR+/HER2-	35.909650924	1	0.9003756714			2
HR+/HER2-	36.008213552	1	0.9003756714			2
HR+/HER2-	36.008213552	1	0.9003756714			2
HR+/HER2-	36.041067762	1	0.9003756714			2
HR+/HER2-	36.205338809	0	0.898708309	0.8738240321	0.9189137175	2
HR+/HER2-	36.435318275	1	0.898708309			2
HR+/HER2-	36.501026694	0	0.8970378475	0.8719592419	0.9174381401	2
HR+/HER2-	36.665297741	1	0.8970378475			2
HR+/HER2-	36.698151951	1	0.8970378475			2
HR+/HER2-	36.862422998	0	0.8953611412	0.8700891558	0.9159555286	2
HR+/HER2-	37.158110883	1	0.8953611412			2
HR+/HER2-	37.519507187	1	0.8953611412			2
HR+/HER2-	37.585215606	1	0.8953611412			2
HR+/HER2-	37.618069815	0	0.893674962	0.8682096078	0.9144634084	2
HR+/HER2-	37.815195072	1	0.893674962			2
HR+/HER2-	37.9137577	1	0.893674962			2
HR+/HER2-	38.373716632	0	0.8919823958	0.8663245673	0.9129641625	2
HR+/HER2-	38.702258727	1	0.8919823958			2
HR+/HER2-	38.702258727	1	0.8919823958			2
HR+/HER2-	38.932238193	1	0.8919823958			2
HR+/HER2-	39.162217659	1	0.8919823958			2
HR+/HER2-	39.195071869	1	0.8919823958			2
HR+/HER2-	39.227926078	1	0.8919823958			2
HR+/HER2-	39.359342916	1	0.8919823958			2
HR+/HER2-	39.392197125	1	0.8919823958			2
HR+/HER2-	39.490759754	1	0.8919823958			2
HR+/HER2-	39.589322382	1	0.8919823958			2
HR+/HER2-	39.622176591	1	0.8919823958			2
HR+/HER2-	39.68788501	1	0.8919823958			2
HR+/HER2-	39.72073922	1	0.8919823958			2
HR+/HER2-	39.852156057	1	0.8919823958			2
HR+/HER2-	39.917864476	1	0.8919823958			2
HR+/HER2-	40.114989733	1	0.8919823958			2

HR+/HER2-	40.180698152	1	0.8919823958			2
HR+/HER2-	40.180698152	1	0.8919823958			2
HR+/HER2-	40.344969199	1	0.8919823958			2
HR+/HER2-	40.509240246	1	0.8919823958			2
HR+/HER2-	40.574948665	1	0.8919823958			2
HR+/HER2-	40.772073922	1	0.8919823958			2
HR+/HER2-	40.804928131	1	0.8919823958			2
HR+/HER2-	40.837782341	1	0.8919823958			2
HR+/HER2-	40.837782341	1	0.8919823958			2
HR+/HER2-	40.837782341	1	0.8919823958			2
HR+/HER2-	40.87063655	1	0.8919823958			2
HR+/HER2-	40.936344969	0	0.890198431	0.8643243674	0.9113925218	2
HR+/HER2-	41.133470226	0	0.8884144662	0.8623274587	0.9098183307	2
HR+/HER2-	41.264887064	1	0.8884144662			2
HR+/HER2-	41.330595483	1	0.8884144662			2
HR+/HER2-	41.527720739	1	0.8884144662			2
HR+/HER2-	41.593429158	0	0.8866196895	0.8603197765	0.9082333672	2
HR+/HER2-	41.691991786	1	0.8866196895			2
HR+/HER2-	41.691991786	1	0.8866196895			2
HR+/HER2-	41.757700205	1	0.8866196895			2
HR+/HER2-	41.790554415	1	0.8866196895			2
HR+/HER2-	41.823408624	0	0.8848102616	0.8582963696	0.9066346878	2
HR+/HER2-	41.954825462	1	0.8848102616			2
HR+/HER2-	42.020533881	1	0.8848102616			2
HR+/HER2-	42.151950719	1	0.8848102616			2
HR+/HER2-	42.184804928	1	0.8848102616			2
HR+/HER2-	42.184804928	1	0.8848102616			2
HR+/HER2-	42.381930185	1	0.8848102616			2
HR+/HER2-	42.381930185	1	0.8848102616			2
HR+/HER2-	42.414784394	1	0.8848102616			2
HR+/HER2-	42.447638604	1	0.8848102616			2
HR+/HER2-	42.480492813	1	0.8848102616			2
HR+/HER2-	42.546201232	0	0.8829630586	0.8562273619	0.9050045923	2
HR+/HER2-	42.611909651	0	0.8811158555	0.8541617659	0.9033718753	2
HR+/HER2-	43.104722793	1	0.8811158555			2
HR+/HER2-	43.104722793	1	0.8811158555			2
HR+/HER2-	43.137577002	1	0.8811158555			2
HR+/HER2-	43.170431211	0	0.8792569613	0.8520844071	0.9017276325	2
HR+/HER2-	43.367556468	1	0.8792569613			2
HR+/HER2-	43.400410678	1	0.8792569613			2
HR+/HER2-	43.400410678	1	0.8792569613			2
HR+/HER2-	43.400410678	1	0.8792569613			2
HR+/HER2-	43.400410678	1	0.8792569613			2
HR+/HER2-	43.531827515	1	0.8792569613			2
HR+/HER2-	43.597535934	1	0.8792569613			2
HR+/HER2-	43.630390144	1	0.8792569613			2
HR+/HER2-	44.090349076	1	0.8792569613			2
HR+/HER2-	44.714579055	1	0.8792569613			2
HR+/HER2-	44.911704312	1	0.8792569613			2
HR+/HER2-	44.911704312	1	0.8792569613			2

HR+/HER2-	44.911704312	1	0.8792569613			2
HR+/HER2-	45.108829569	1	0.8792569613			2
HR+/HER2-	45.667351129	1	0.8792569613			2
HR+/HER2-	45.831622177	1	0.8792569613			2
HR+/HER2-	45.963039014	1	0.8792569613			2
HR+/HER2-	46.061601643	1	0.8792569613			2
HR+/HER2-	46.127310062	1	0.8792569613			2
HR+/HER2-	46.258726899	1	0.8792569613			2
HR+/HER2-	46.357289528	1	0.8792569613			2
HR+/HER2-	46.357289528	1	0.8792569613			2
HR+/HER2-	46.915811088	0	0.8773073893	0.849892909	0.9000116592	2
HR+/HER2-	46.981519507	1	0.8773073893			2
HR+/HER2-	47.145790554	1	0.8773073893			2
HR+/HER2-	47.277207392	0	0.8753491139	0.8476940448	0.8982861158	2
HR+/HER2-	47.441478439	1	0.8753491139			2
HR+/HER2-	47.507186858	1	0.8753491139			2
HR+/HER2-	47.704312115	1	0.8753491139			2
HR+/HER2-	47.737166324	1	0.8753491139			2
HR+/HER2-	47.967145791	1	0.8753491139			2
HR+/HER2-	48	1	0.8753491139			2
HR+/HER2-	48.131416838	1	0.8753491139			2
HR+/HER2-	48.131416838	1	0.8753491139			2
HR+/HER2-	48.262833676	0	0.8733551524	0.8454526818	0.8965305549	2
HR+/HER2-	48.459958932	1	0.8733551524			2
HR+/HER2-	48.689938398	1	0.8733551524			2
HR+/HER2-	48.788501027	1	0.8733551524			2
HR+/HER2-	49.18275154	0	0.8713474394	0.843197523	0.8947615095	2
HR+/HER2-	49.347022587	1	0.8713474394			2
HR+/HER2-	49.478439425	1	0.8713474394			2
HR+/HER2-	50.234086242	1	0.8713474394			2
HR+/HER2-	50.234086242	1	0.8713474394			2
HR+/HER2-	50.332648871	0	0.86932105	0.8409221767	0.8929752365	2
HR+/HER2-	50.39835729	1	0.86932105			2
HR+/HER2-	50.759753593	1	0.86932105			2
HR+/HER2-	51.383983573	1	0.86932105			2
HR+/HER2-	52.041067762	1	0.86932105			2
HR+/HER2-	52.205338809	0	0.8672755887	0.8386261773	0.8911714059	2
HR+/HER2-	52.238193018	1	0.8672755887			2
HR+/HER2-	52.566735113	0	0.8652252918	0.8363280638	0.8893607936	2
HR+/HER2-	52.73100616	0	0.8631749949	0.8340340242	0.8875471272	2
HR+/HER2-	52.928131417	1	0.8631749949			2
HR+/HER2-	52.960985626	1	0.8631749949			2
HR+/HER2-	52.993839836	1	0.8631749949			2
HR+/HER2-	52.993839836	1	0.8631749949			2
HR+/HER2-	53.388090349	1	0.8631749949			2
HR+/HER2-	53.453798768	1	0.8631749949			2
HR+/HER2-	53.519507187	1	0.8631749949			2
HR+/HER2-	53.618069815	1	0.8631749949			2
HR+/HER2-	53.683778234	0	0.8610849828	0.8316925769	0.8857002012	2
HR+/HER2-	53.979466119	0	0.8569049586	0.8270220583	0.8819970693	2

HR+/HER2-	53.979466119	1	0.8569049586			2
HR+/HER2-	54.439425051	1	0.8569049586			2
HR+/HER2-	54.439425051	1	0.8569049586			2
HR+/HER2-	54.53798768	1	0.8569049586			2
HR+/HER2-	54.636550308	1	0.8569049586			2
HR+/HER2-	54.669404517	1	0.8569049586			2
HR+/HER2-	55.063655031	1	0.8569049586			2
HR+/HER2-	55.359342916	1	0.8569049586			2
HR+/HER2-	55.589322382	1	0.8569049586			2
HR+/HER2-	55.72073922	1	0.8569049586			2
HR+/HER2-	55.917864476	1	0.8569049586			2
HR+/HER2-	55.950718686	1	0.8569049586			2
HR+/HER2-	56.27926078	1	0.8569049586			2
HR+/HER2-	56.607802875	1	0.8569049586			2
HR+/HER2-	56.837782341	0	0.8547410572	0.824597328	0.8800846317	2
HR+/HER2-	56.87063655	1	0.8547410572			2
HR+/HER2-	56.87063655	1	0.8547410572			2
HR+/HER2-	57.330595483	1	0.8547410572			2
HR+/HER2-	57.330595483	1	0.8547410572			2
HR+/HER2-	57.823408624	1	0.8547410572			2
HR+/HER2-	57.856262834	1	0.8547410572			2
HR+/HER2-	58.0862423	1	0.8547410572			2
HR+/HER2-	58.119096509	1	0.8547410572			2
HR+/HER2-	58.151950719	0	0.852532424	0.8221190023	0.8781349559	2
HR+/HER2-	58.480492813	1	0.852532424			2
HR+/HER2-	58.480492813	1	0.852532424			2
HR+/HER2-	58.513347023	1	0.852532424			2
HR+/HER2-	58.743326489	1	0.852532424			2
HR+/HER2-	58.841889117	1	0.852532424			2
HR+/HER2-	59.137577002	1	0.852532424			2
HR+/HER2-	59.203285421	1	0.852532424			2
HR+/HER2-	59.630390144	1	0.852532424			2
HR+/HER2-	59.630390144	1	0.852532424			2
HR+/HER2-	59.696098563	1	0.852532424			2
HR+/HER2-	60.090349076	1	0.852532424			2
HR+/HER2-	60.287474333	1	0.852532424			2
HR+/HER2-	60.353182752	1	0.852532424			2
HR+/HER2-	60.353182752	1	0.852532424			2
HR+/HER2-	60.616016427	1	0.852532424			2
HR+/HER2-	60.780287474	1	0.852532424			2
HR+/HER2-	61.437371663	1	0.852532424			2
HR+/HER2-	61.535934292	1	0.852532424			2
HR+/HER2-	61.700205339	1	0.852532424			2
HR+/HER2-	62.028747433	1	0.852532424			2
HR+/HER2-	62.160164271	1	0.852532424			2
HR+/HER2-	62.22587269	1	0.852532424			2
HR+/HER2-	62.422997947	1	0.852532424			2
HR+/HER2-	62.587268994	1	0.852532424			2
HR+/HER2-	62.587268994	1	0.852532424			2
HR+/HER2-	62.718685832	1	0.852532424			2

HR+/HER2-	63.047227926	1	0.852532424			2
HR+/HER2-	63.310061602	1	0.852532424			2
HR+/HER2-	63.967145791	1	0.852532424			2
HR+/HER2-	64.164271047	1	0.852532424			2
HR+/HER2-	64.197125257	0	0.85013767	0.8194013175	0.8760424993	2
HR+/HER2-	64.295687885	1	0.85013767			2
HR+/HER2-	64.328542094	1	0.85013767			2
HR+/HER2-	64.525667351	1	0.85013767			2
HR+/HER2-	64.887063655	0	0.8477225061	0.8166629928	0.8739303682	2
HR+/HER2-	64.919917864	1	0.8477225061			2
HR+/HER2-	64.952772074	1	0.8477225061			2
HR+/HER2-	64.985626283	1	0.8477225061			2
HR+/HER2-	65.248459959	1	0.8477225061			2
HR+/HER2-	65.379876797	1	0.8477225061			2
HR+/HER2-	66.036960986	1	0.8477225061			2
HR+/HER2-	66.102669405	1	0.8477225061			2
HR+/HER2-	66.135523614	1	0.8477225061			2
HR+/HER2-	66.135523614	1	0.8477225061			2
HR+/HER2-	66.266940452	1	0.8477225061			2
HR+/HER2-	66.825462012	1	0.8477225061			2
HR+/HER2-	67.055441478	0	0.8452292046	0.8138274804	0.8717559933	2
HR+/HER2-	67.285420945	1	0.8452292046			2
HR+/HER2-	67.449691992	1	0.8452292046			2
HR+/HER2-	67.449691992	1	0.8452292046			2
HR+/HER2-	67.876796715	0	0.8427136415	0.810969403	0.8695602314	2
HR+/HER2-	67.876796715	1	0.8427136415			2
HR+/HER2-	68.106776181	1	0.8427136415			2
HR+/HER2-	68.13963039	1	0.8427136415			2
HR+/HER2-	68.13963039	1	0.8427136415			2
HR+/HER2-	68.566735113	1	0.8427136415			2
HR+/HER2-	68.665297741	1	0.8427136415			2
HR+/HER2-	68.665297741	1	0.8427136415			2
HR+/HER2-	68.698151951	1	0.8427136415			2
HR+/HER2-	69.519507187	0	0.8401365356	0.8080365469	0.8673142298	2
HR+/HER2-	69.683778234	0	0.8375594297	0.8051111349	0.865062905	2
HR+/HER2-	69.749486653	1	0.8375594297			2
HR+/HER2-	69.815195072	1	0.8375594297			2
HR+/HER2-	70.242299795	1	0.8375594297			2
HR+/HER2-	70.275154004	1	0.8375594297			2
HR+/HER2-	70.439425051	1	0.8375594297			2
HR+/HER2-	70.472279261	1	0.8375594297			2
HR+/HER2-	71.425051335	1	0.8375594297			2
HR+/HER2-	71.457905544	1	0.8375594297			2
HR+/HER2-	71.655030801	1	0.8375594297			2
HR+/HER2-	71.655030801	1	0.8375594297			2
HR+/HER2-	72.049281314	1	0.8375594297			2
HR+/HER2-	72.344969199	1	0.8375594297			2
HR+/HER2-	72.377823409	1	0.8375594297			2
HR+/HER2-	72.377823409	1	0.8375594297			2
HR+/HER2-	72.509240246	1	0.8375594297			2

HR+/HER2-	72.542094456	1	0.8375594297			2
HR+/HER2-	73.002053388	1	0.8375594297			2
HR+/HER2-	73.034907598	1	0.8375594297			2
HR+/HER2-	73.034907598	1	0.8375594297			2
HR+/HER2-	73.199178645	0	0.8348223073	0.8019793789	0.8626895926	2
HR+/HER2-	73.396303901	1	0.8348223073			2
HR+/HER2-	73.46201232	1	0.8348223073			2
HR+/HER2-	73.757700205	1	0.8348223073			2
HR+/HER2-	74.283367556	0	0.8320579951	0.7988199834	0.8602903066	2
HR+/HER2-	74.316221766	1	0.8320579951			2
HR+/HER2-	74.874743326	1	0.8320579951			2
HR+/HER2-	74.907597536	0	0.8292751924	0.79564468	0.8578713024	2
HR+/HER2-	75.137577002	1	0.8292751924			2
HR+/HER2-	75.498973306	1	0.8292751924			2
HR+/HER2-	75.564681725	1	0.8292751924			2
HR+/HER2-	75.696098563	1	0.8292751924			2
HR+/HER2-	75.728952772	1	0.8292751924			2
HR+/HER2-	75.8275154	1	0.8292751924			2
HR+/HER2-	75.893223819	1	0.8292751924			2
HR+/HER2-	75.893223819	1	0.8292751924			2
HR+/HER2-	75.893223819	1	0.8292751924			2
HR+/HER2-	76.353182752	0	0.8264057281	0.7923619213	0.8553832849	2
HR+/HER2-	76.484599589	1	0.8264057281			2
HR+/HER2-	76.484599589	1	0.8264057281			2
HR+/HER2-	76.648870637	1	0.8264057281			2
HR+/HER2-	76.747433265	1	0.8264057281			2
HR+/HER2-	76.977412731	1	0.8264057281			2
HR+/HER2-	77.141683778	1	0.8264057281			2
HR+/HER2-	77.305954825	0	0.8234752113	0.7890065814	0.85284447	2
HR+/HER2-	77.371663244	1	0.8234752113			2
HR+/HER2-	77.503080082	0	0.8205342284	0.7856471646	0.8502910108	2
HR+/HER2-	77.63449692	1	0.8205342284			2
HR+/HER2-	77.63449692	1	0.8205342284			2
HR+/HER2-	77.995893224	1	0.8205342284			2
HR+/HER2-	78.061601643	1	0.8205342284			2
HR+/HER2-	78.324435318	1	0.8205342284			2
HR+/HER2-	78.324435318	1	0.8205342284			2
HR+/HER2-	78.488706366	1	0.8205342284			2
HR+/HER2-	78.81724846	1	0.8205342284			2
HR+/HER2-	78.981519507	0	0.8175064268	0.7821807715	0.8476680341	2
HR+/HER2-	79.047227926	1	0.8175064268			2
HR+/HER2-	79.178644764	1	0.8175064268			2
HR+/HER2-	79.474332649	1	0.8175064268			2
HR+/HER2-	79.507186858	1	0.8175064268			2
HR+/HER2-	79.638603696	0	0.8144330944	0.7786635887	0.8450048509	2
HR+/HER2-	79.868583162	1	0.8144330944			2
HR+/HER2-	79.967145791	1	0.8144330944			2
HR+/HER2-	80.164271047	1	0.8144330944			2
HR+/HER2-	80.164271047	1	0.8144330944			2
HR+/HER2-	80.197125257	1	0.8144330944			2

HR+/HER2-	80.262833676	0	0.8113006594	0.7750775213	0.8422915943	2
HR+/HER2-	80.328542094	1	0.8113006594			2
HR+/HER2-	80.394250513	1	0.8113006594			2
HR+/HER2-	80.788501027	1	0.8113006594			2
HR+/HER2-	81.018480493	1	0.8113006594			2
HR+/HER2-	81.314168378	1	0.8113006594			2
HR+/HER2-	81.80698152	1	0.8113006594			2
HR+/HER2-	81.80698152	1	0.8113006594			2
HR+/HER2-	81.938398357	1	0.8113006594			2
HR+/HER2-	81.971252567	1	0.8113006594			2
HR+/HER2-	82.135523614	0	0.8080554568	0.7713501465	0.8394898765	2
HR+/HER2-	82.464065708	1	0.8080554568			2
HR+/HER2-	82.595482546	1	0.8080554568			2
HR+/HER2-	82.661190965	1	0.8080554568			2
HR+/HER2-	83.121149897	1	0.8080554568			2
HR+/HER2-	83.154004107	1	0.8080554568			2
HR+/HER2-	83.318275154	1	0.8080554568			2
HR+/HER2-	83.646817248	1	0.8080554568			2
HR+/HER2-	84.041067762	1	0.8080554568			2
HR+/HER2-	84.238193018	1	0.8080554568			2
HR+/HER2-	84.336755647	1	0.8080554568			2
HR+/HER2-	84.336755647	1	0.8080554568			2
HR+/HER2-	84.501026694	1	0.8080554568			2
HR+/HER2-	84.501026694	1	0.8080554568			2
HR+/HER2-	84.566735113	1	0.8080554568			2
HR+/HER2-	84.895277207	1	0.8080554568			2
HR+/HER2-	84.895277207	1	0.8080554568			2
HR+/HER2-	84.928131417	1	0.8080554568			2
HR+/HER2-	85.125256674	1	0.8080554568			2
HR+/HER2-	85.223819302	1	0.8080554568			2
HR+/HER2-	85.256673511	1	0.8080554568			2
HR+/HER2-	85.388090349	1	0.8080554568			2
HR+/HER2-	86.308008214	1	0.8080554568			2
HR+/HER2-	86.340862423	1	0.8080554568			2
HR+/HER2-	86.340862423	1	0.8080554568			2
HR+/HER2-	86.406570842	1	0.8080554568			2
HR+/HER2-	86.50513347	1	0.8080554568			2
HR+/HER2-	86.636550308	1	0.8080554568			2
HR+/HER2-	86.735112936	1	0.8080554568			2
HR+/HER2-	87.030800821	1	0.8080554568			2
HR+/HER2-	87.063655031	1	0.8080554568			2
HR+/HER2-	87.293634497	1	0.8080554568			2
HR+/HER2-	87.425051335	1	0.8080554568			2
HR+/HER2-	87.753593429	1	0.8080554568			2
HR+/HER2-	88.114989733	1	0.8080554568			2
HR+/HER2-	88.410677618	1	0.8080554568			2
HR+/HER2-	88.410677618	1	0.8080554568			2
HR+/HER2-	88.673511294	1	0.8080554568			2
HR+/HER2-	88.837782341	1	0.8080554568			2
HR+/HER2-	89.034907598	1	0.8080554568			2

HR+/HER2-	89.034907598	1	0.8080554568			2
HR+/HER2-	89.166324435	1	0.8080554568			2
HR+/HER2-	89.199178645	0	0.8041705748	0.7667447183	0.836241625	2
HR+/HER2-	89.560574949	1	0.8041705748			2
HR+/HER2-	89.790554415	1	0.8041705748			2
HR+/HER2-	90.316221766	1	0.8041705748			2
HR+/HER2-	90.414784394	0	0.8002285621	0.7620820089	0.8329387827	2
HR+/HER2-	90.64476386	1	0.8002285621			2
HR+/HER2-	90.67761807	1	0.8002285621			2
HR+/HER2-	90.973305955	1	0.8002285621			2
HR+/HER2-	91.301848049	1	0.8002285621			2
HR+/HER2-	91.433264887	1	0.8002285621			2
HR+/HER2-	91.498973306	1	0.8002285621			2
HR+/HER2-	91.630390144	1	0.8002285621			2
HR+/HER2-	91.8275154	1	0.8002285621			2
HR+/HER2-	92.123203285	1	0.8002285621			2
HR+/HER2-	92.156057495	1	0.8002285621			2
HR+/HER2-	92.254620123	1	0.8002285621			2
HR+/HER2-	92.287474333	1	0.8002285621			2
HR+/HER2-	92.287474333	1	0.8002285621			2
HR+/HER2-	92.287474333	1	0.8002285621			2
HR+/HER2-	92.386036961	0	0.7959945486	0.7570247268	0.8294284584	2
HR+/HER2-	92.484599589	1	0.7959945486			2
HR+/HER2-	92.714579055	1	0.7959945486			2
HR+/HER2-	93.503080082	0	0.791715008	0.7519318621	0.8258676585	2
HR+/HER2-	93.63449692	1	0.791715008			2
HR+/HER2-	93.963039014	1	0.791715008			2
HR+/HER2-	93.963039014	1	0.791715008			2
HR+/HER2-	93.963039014	1	0.791715008			2
HR+/HER2-	94.127310062	0	0.7873408919	0.7467325205	0.8222248416	2
HR+/HER2-	94.422997947	1	0.7873408919			2
HR+/HER2-	94.554414784	1	0.7873408919			2
HR+/HER2-	94.784394251	1	0.7873408919			2
HR+/HER2-	94.784394251	1	0.7873408919			2
HR+/HER2-	94.882956879	1	0.7873408919			2
HR+/HER2-	94.915811088	1	0.7873408919			2
HR+/HER2-	94.915811088	1	0.7873408919			2
HR+/HER2-	94.915811088	1	0.7873408919			2
HR+/HER2-	94.981519507	1	0.7873408919			2
HR+/HER2-	95.277207392	1	0.7873408919			2
HR+/HER2-	95.310061602	1	0.7873408919			2
HR+/HER2-	95.507186858	1	0.7873408919			2
HR+/HER2-	95.605749487	1	0.7873408919			2
HR+/HER2-	95.704312115	1	0.7873408919			2
HR+/HER2-	95.802874743	1	0.7873408919			2
HR+/HER2-	96.065708419	1	0.7873408919			2
HR+/HER2-	96.755646817	1	0.7873408919			2
HR+/HER2-	96.854209446	1	0.7873408919			2
HR+/HER2-	96.985626283	1	0.7873408919			2
HR+/HER2-	97.117043121	1	0.7873408919			2

HR+/HER2-	97.577002053	1	0.7873408919			2
HR+/HER2-	98.135523614	1	0.7873408919			2
HR+/HER2-	98.464065708	0	0.7823577217	0.7406721142	0.8181775278	2
HR+/HER2-	98.628336756	0	0.7773745515	0.7346586354	0.8140973251	2
HR+/HER2-	98.759753593	1	0.7773745515			2
HR+/HER2-	98.825462012	0	0.7723592318	0.7286416175	0.8099665086	2
HR+/HER2-	99.186858316	1	0.7723592318			2
HR+/HER2-	99.252566735	1	0.7723592318			2
HR+/HER2-	99.613963039	0	0.7672779211	0.722570145	0.8057648491	2
HR+/HER2-	99.646817248	0	0.7621966104	0.7165397133	0.8015345571	2
HR+/HER2-	99.942505133	1	0.7621966104			2
HR+/HER2-	100.10677618	1	0.7621966104			2
HR+/HER2-	100.33675565	1	0.7621966104			2
HR+/HER2-	100.56673511	1	0.7621966104			2
HR+/HER2-	100.89527721	1	0.7621966104			2
HR+/HER2-	100.89527721	1	0.7621966104			2
HR+/HER2-	101.09240246	1	0.7621966104			2
HR+/HER2-	101.25667351	1	0.7621966104			2
HR+/HER2-	101.28952772	1	0.7621966104			2
HR+/HER2-	101.32238193	0	0.7567909607	0.7100776973	0.7970709059	2
HR+/HER2-	101.35523614	0	0.7513853109	0.7036616362	0.7925752132	2
HR+/HER2-	101.68377823	1	0.7513853109			2
HR+/HER2-	102.04517454	1	0.7513853109			2
HR+/HER2-	102.83367556	1	0.7513853109			2
HR+/HER2-	102.89938398	1	0.7513853109			2
HR+/HER2-	103.12936345	1	0.7513853109			2
HR+/HER2-	103.16221766	1	0.7513853109			2
HR+/HER2-	103.26078029	0	0.7457357973	0.6969355039	0.7878943786	2
HR+/HER2-	103.2936345	1	0.7457357973			2
HR+/HER2-	103.32648871	1	0.7457357973			2
HR+/HER2-	103.78644764	1	0.7457357973			2
HR+/HER2-	103.88501027	1	0.7457357973			2
HR+/HER2-	104.04928131	1	0.7457357973			2
HR+/HER2-	104.24640657	1	0.7457357973			2
HR+/HER2-	104.3449692	1	0.7457357973			2
HR+/HER2-	104.50924025	1	0.7457357973			2
HR+/HER2-	104.67351129	1	0.7457357973			2
HR+/HER2-	104.7063655	1	0.7457357973			2
HR+/HER2-	104.73921971	1	0.7457357973			2
HR+/HER2-	104.93634497	1	0.7457357973			2
HR+/HER2-	105.00205339	1	0.7457357973			2
HR+/HER2-	105.00205339	1	0.7457357973			2
HR+/HER2-	105.00205339	1	0.7457357973			2
HR+/HER2-	105.0349076	1	0.7457357973			2
HR+/HER2-	105.0349076	1	0.7457357973			2
HR+/HER2-	105.13347023	1	0.7457357973			2
HR+/HER2-	105.19917864	1	0.7457357973			2
HR+/HER2-	105.62628337	0	0.7391363655	0.688845256	0.7826039367	2
HR+/HER2-	106.18480493	1	0.7391363655			2
HR+/HER2-	106.41478439	1	0.7391363655			2

HR+/HER2-	107.00616016	1	0.7391363655			2
HR+/HER2-	107.00616016	1	0.7391363655			2
HR+/HER2-	107.20328542	1	0.7391363655			2
HR+/HER2-	107.20328542	1	0.7391363655			2
HR+/HER2-	107.53182752	1	0.7391363655			2
HR+/HER2-	107.99178645	1	0.7391363655			2
HR+/HER2-	108.15605749	1	0.7391363655			2
HR+/HER2-	108.22176591	1	0.7391363655			2
HR+/HER2-	108.45174538	1	0.7391363655			2
HR+/HER2-	109.04312115	1	0.7391363655			2
HR+/HER2-	109.10882957	1	0.7391363655			2
HR+/HER2-	109.73305955	1	0.7391363655			2
HR+/HER2-	109.76591376	0	0.7315941577	0.6793784337	0.7767277927	2
HR+/HER2-	109.83162218	1	0.7315941577			2
HR+/HER2-	110.52156057	1	0.7315941577			2
HR+/HER2-	110.55441478	1	0.7315941577			2
HR+/HER2-	111.14579055	1	0.7315941577			2
HR+/HER2-	111.17864476	1	0.7315941577			2
HR+/HER2-	111.40862423	1	0.7315941577			2
HR+/HER2-	111.44147844	1	0.7315941577			2
HR+/HER2-	111.67145791	1	0.7315941577			2
HR+/HER2-	111.80287474	1	0.7315941577			2
HR+/HER2-	111.86858316	1	0.7315941577			2
HR+/HER2-	111.90143737	1	0.7315941577			2
HR+/HER2-	111.93429158	1	0.7315941577			2
HR+/HER2-	111.93429158	1	0.7315941577			2
HR+/HER2-	112.13141684	1	0.7315941577			2
HR+/HER2-	112.59137577	1	0.7315941577			2
HR+/HER2-	113.08418891	1	0.7315941577			2
HR+/HER2-	113.51129363	1	0.7315941577			2
HR+/HER2-	113.54414784	1	0.7315941577			2
HR+/HER2-	113.67556468	1	0.7315941577			2
HR+/HER2-	113.93839836	1	0.7315941577			2
HR+/HER2-	114.03696099	0	0.7220929349	0.6668345679	0.7697951524	2
HR+/HER2-	114.13552361	0	0.712591712	0.6545540109	0.7626759922	2
HR+/HER2-	114.20123203	1				2
HR+/HER2-	114.46406571	1				2
HR+/HER2-	114.92402464	1				2
HR+/HER2-	115.05544148	1				2
HR+/HER2-	115.35112936	1				2
HR+/HER2-	115.4825462	1				2
HR+/HER2-	116.40246407	1				2
HR+/HER2-	116.43531828	1				2
HR+/HER2-	116.50102669	1				2
HR+/HER2-	116.66529774	1				2
HR+/HER2-	116.66529774	1				2
HR+/HER2-	116.66529774	1				2
HR+/HER2-	116.89527721	1				2
HR+/HER2-	117.38809035	1				2
HR+/HER2-	117.88090349	1				2

HR+/HER2-	117.88090349	1				2
HR+/HER2-	117.9137577	1				2
HR+/HER2-	118.04517454	1				2
HR+/HER2-	118.07802875	1				2
HR+/HER2-	118.07802875	1				2
HR+/HER2-	118.14373717	1				2
HR+/HER2-	118.14373717	1				2
HR+/HER2-	118.50513347	1				2
HR+/HER2-	118.50513347	1				2
HR+/HER2-	118.83367556	1				2
HR+/HER2-	118.9650924	1				2
HR+/HER2-	119.26078029	1				2
HR+/HER2-	119.42505133	1				2
HR+/HER2-	119.75359343	1				2
HR+/HER2-	119.95071869	1				2
HR+/HER2-	120.08213552	1				2
HR+/HER2-	120.31211499	1				2
HR+/HER2-	120.41067762	1				2
HR+/HER2-	120.41067762	1				2
HR+/HER2-	120.44353183	1				2
HR+/HER2-	121.10061602	1				2
HR+/HER2-	121.13347023	1				2
HR+/HER2-	121.13347023	1				2
HR+/HER2-	121.75770021	1				2
HR+/HER2-	122.02053388	1				2
HR+/HER2-	122.18480493	1				2
HR+/HER2-	122.64476386	1				2
HR+/HER2-	122.71047228	1				2
HR+/HER2-	122.71047228	1				2
HR+/HER2-	122.84188912	1				2
HR+/HER2-	123.33470226	1				2
HR+/HER2-	123.33470226	1				2
HR+/HER2-	123.33470226	1				2
HR+/HER2-	123.76180698	1				2
HR+/HER2-	124.25462012	1				2
HR+/HER2-	124.28747433	1				2
HR+/HER2-	124.48459959	1				2
HR+/HER2-	124.97741273	1				2
HR+/HER2-	125.37166324	1				2
HR+/HER2-	125.73305955	1				2
HR+/HER2-	125.73305955	1				2
HR+/HER2-	125.73305955	1				2
HR+/HER2-	125.83162218	1				2
HR+/HER2-	126.19301848	1				2
HR+/HER2-	126.55441478	1				2
HR+/HER2-	126.58726899	1				2
HR+/HER2-	126.88295688	1				2
HR+/HER2-	127.11293634	1				2
HR+/HER2-	127.34291581	1				2
HR+/HER2-	127.54004107	1				2

HR+/HER2-	127.70431211	1				2
HR+/HER2-	127.90143737	1				2
HR+/HER2-	127.93429158	1				2
HR+/HER2-	128.13141684	1				2
HR+/HER2-	128.13141684	1				2
HR+/HER2-	128.42710472	1				2
HR+/HER2-	128.45995893	1				2
HR+/HER2-	128.85420945	1				2
HR+/HER2-	129.0513347	1				2
HR+/HER2-	129.08418891	1				2
HR-/HER2+	0		1	1	1	3
HR-/HER2+	1.2484599589	0	0.992248062	0.9462521106	0.9989043866	3
HR-/HER2+	2.8911704312	1	0.992248062			3
HR-/HER2+	3.2525667351	0	0.9844350851	0.9391992059	0.9960845049	3
HR-/HER2+	3.6796714579	0	0.9766221083	0.9292758213	0.9924000614	3
HR-/HER2+	5.0595482546	0	0.9688091314	0.9190316617	0.988178309	3
HR-/HER2+	5.8809034908	0	0.9609961546	0.9088344232	0.983578147	3
HR-/HER2+	6.1437371663	0	0.9531831777	0.8987614958	0.9786912394	3
HR-/HER2+	6.2094455852	0	0.9453702008	0.8888250626	0.9735763005	3
HR-/HER2+	6.4722792608	0	0.937557224	0.8790197106	0.9682736893	3
HR-/HER2+	6.5051334702	1	0.937557224			3
HR-/HER2+	6.5708418891	0	0.9296785918	0.8692145952	0.9627780872	3
HR-/HER2+	10.184804928	1	0.9296785918			3
HR-/HER2+	10.283367556	1	0.9296785918			3
HR-/HER2+	10.349075975	0	0.9136496506	0.8494810349	0.9512348536	3
HR-/HER2+	10.447638604	1	0.9136496506			3
HR-/HER2+	12.254620123	1	0.9136496506			3
HR-/HER2+	12.944558522	1	0.9136496506			3
HR-/HER2+	12.977412731	1	0.9136496506			3
HR-/HER2+	13.01026694	1	0.9136496506			3
HR-/HER2+	14.784394251	1	0.9136496506			3
HR-/HER2+	14.882956879	1	0.9136496506			3
HR-/HER2+	15.112936345	1	0.9136496506			3
HR-/HER2+	15.310061602	1	0.9136496506			3
HR-/HER2+	15.934291581	1	0.9136496506			3
HR-/HER2+	17.117043121	1	0.9136496506			3
HR-/HER2+	17.347022587	1	0.9136496506			3
HR-/HER2+	17.839835729	1	0.9136496506			3
HR-/HER2+	18.036960986	1	0.9136496506			3
HR-/HER2+	18.924024641	1	0.9136496506			3
HR-/HER2+	19.416837782	0	0.9044208662	0.8376493823	0.9446237554	3
HR-/HER2+	19.58110883	1	0.9044208662			3
HR-/HER2+	19.942505133	0	0.8950969398	0.8258850324	0.9378156877	3
HR-/HER2+	20.106776181	1	0.8950969398			3
HR-/HER2+	20.369609856	1	0.8950969398			3
HR-/HER2+	20.369609856	1	0.8950969398			3
HR-/HER2+	20.402464066	1	0.8950969398			3
HR-/HER2+	20.796714579	1	0.8950969398			3
HR-/HER2+	20.862422998	1	0.8950969398			3
HR-/HER2+	22.340862423	1	0.8950969398			3

HR-/HER2+	23.490759754	1	0.8950969398			3
HR-/HER2+	24.640657084	1	0.8950969398			3
HR-/HER2+	25.856262834	0	0.8848084692	0.8126294459	0.9303546648	3
HR-/HER2+	26.184804928	1	0.8848084692			3
HR-/HER2+	27.23613963	0	0.8743989578	0.7994521658	0.9226696845	3
HR-/HER2+	27.893223819	1	0.8743989578			3
HR-/HER2+	28.156057495	1	0.8743989578			3
HR-/HER2+	29.700205339	0	0.8637355559	0.7860966529	0.9146981161	3
HR-/HER2+	30.652977413	0	0.853072154	0.7729857751	0.9065845467	3
HR-/HER2+	30.652977413	1	0.853072154			3
HR-/HER2+	30.981519507	0	0.8422737723	0.7598642107	0.8982642183	3
HR-/HER2+	31.901437372	0	0.8314753906	0.7469376572	0.8898214618	3
HR-/HER2+	35.219712526	1	0.8314753906			3
HR-/HER2+	35.909650924	1	0.8314753906			3
HR-/HER2+	36.468172485	1	0.8314753906			3
HR-/HER2+	36.862422998	1	0.8314753906			3
HR-/HER2+	36.895277207	1	0.8314753906			3
HR-/HER2+	39.917864476	1	0.8314753906			3
HR-/HER2+	40.082135524	1	0.8314753906			3
HR-/HER2+	43.23613963	0	0.8195971707	0.7324506086	0.8806335299	3
HR-/HER2+	45.930184805	1	0.8195971707			3
HR-/HER2+	45.963039014	0	0.8075442711	0.7179234181	0.871203636	3
HR-/HER2+	46.127310062	0	0.7954913716	0.7036221842	0.8616384024	3
HR-/HER2+	46.160164271	1	0.7954913716			3
HR-/HER2+	46.488706366	1	0.7954913716			3
HR-/HER2+	46.751540041	1	0.7954913716			3
HR-/HER2+	46.850102669	1	0.7954913716			3
HR-/HER2+	46.948665298	1	0.7954913716			3
HR-/HER2+	47.507186858	1	0.7954913716			3
HR-/HER2+	48.558521561	1	0.7954913716			3
HR-/HER2+	50.299794661	1	0.7954913716			3
HR-/HER2+	50.464065708	0	0.7817760031	0.6868798524	0.8509813645	3
HR-/HER2+	50.891170431	0	0.7680606346	0.6704560406	0.8401467706	3
HR-/HER2+	52.533880903	1	0.7680606346			3
HR-/HER2+	52.76386037	1	0.7680606346			3
HR-/HER2+	55.293634497	0	0.7538372895	0.6535152657	0.828853292	3
HR-/HER2+	55.556468172	1	0.7538372895			3
HR-/HER2+	56.180698152	1	0.7538372895			3
HR-/HER2+	56.640657084	1	0.7538372895			3
HR-/HER2+	58.67761807	1	0.7538372895			3
HR-/HER2+	60.41889117	1	0.7538372895			3
HR-/HER2+	60.813141684	0	0.738132346	0.6345242321	0.816544091	3
HR-/HER2+	61.273100616	1	0.738132346			3
HR-/HER2+	61.667351129	0	0.7220859906	0.6153893827	0.8038215073	3
HR-/HER2+	63.540041068	1	0.7220859906			3
HR-/HER2+	63.835728953	1	0.7220859906			3
HR-/HER2+	64	1	0.7220859906			3
HR-/HER2+	65.642710472	1	0.7220859906			3
HR-/HER2+	65.839835729	1	0.7220859906			3
HR-/HER2+	68.369609856	1	0.7220859906			3

HR-/HER2+	69.519507187	1	0.7220859906			3
HR-/HER2+	69.782340862	1	0.7220859906			3
HR-/HER2+	70.702258727	1	0.7220859906			3
HR-/HER2+	71.655030801	1	0.7220859906			3
HR-/HER2+	71.786447639	1	0.7220859906			3
HR-/HER2+	72.772073922	1	0.7220859906			3
HR-/HER2+	74.579055441	1	0.7220859906			3
HR-/HER2+	75.531827515	1	0.7220859906			3
HR-/HER2+	76.386036961	0	0.6754997977	0.5547574657	0.7701323513	3
HR-/HER2+	76.944558522	1	0.6754997977			3
HR-/HER2+	77.864476386	1	0.6754997977			3
HR-/HER2+	78.554414784	0	0.6504812867	0.5234296148	0.7515018463	3
HR-/HER2+	80.131416838	1	0.6504812867			3
HR-/HER2+	80.755646817	1	0.6504812867			3
HR-/HER2+	81.051334702	1	0.6504812867			3
HR-/HER2+	83.186858316	1	0.6504812867			3
HR-/HER2+	83.285420945	1	0.6504812867			3
HR-/HER2+	84.106776181	1	0.6504812867			3
HR-/HER2+	85.453798768	1	0.6504812867			3
HR-/HER2+	92.845995893	1	0.6504812867			3
HR-/HER2+	101.25667351	1	0.6504812867			3
HR-/HER2+	103.78644764	1	0.6504812867			3
HR-/HER2+	106.54620123	1	0.6504812867			3
HR-/HER2+	106.61190965	1	0.6504812867			3
HR-/HER2+	106.87474333	0	0.6040183376	0.4526431309	0.7256732341	3
HR-/HER2+	106.87474333	1				3
HR-/HER2+	107.66324435	1				3
HR-/HER2+	109.73305955	1				3
HR-/HER2+	110.55441478	1				3
HR-/HER2+	117.81519507	1				3
HR-/HER2+	117.84804928	1				3
HR-/HER2+	118.11088296	1				3
HR-/HER2+	118.34086242	1				3
HR-/HER2+	121.00205339	1				3
HR-/HER2+	122.74332649	1				3
HR-/HER2+	123.56468172	1				3
HR-/HER2+	123.89322382	1				3
HR-/HER2+	127.01437372	1				3
TNBC	0		1	1	1	4
TNBC	0.295687885	0	0.9969604863	0.9786212356	0.9995712837	4
TNBC	0.5585215606	0	0.9939209726	0.9759139637	0.9984761751	4
TNBC	0.7885010267	1	0.9939209726			4
TNBC	1.0841889117	0	0.9908721353	0.9719685978	0.9970469445	4
TNBC	1.3798767967	0	0.987823298	0.9678838002	0.9954124236	4
TNBC	1.5441478439	0	0.9847744606	0.9638094305	0.9936344068	4
TNBC	1.5770020534	0	0.9817256233	0.9597777624	0.9917484728	4
TNBC	1.7412731006	1	0.9817256233			4
TNBC	2.726899384	0	0.978667288	0.9557748582	0.9897728293	4
TNBC	2.8583162218	0	0.9756089527	0.9518206363	0.9877271641	4
TNBC	3.318275154	0	0.9725506175	0.9479109353	0.9856228411	4

TNBC	3.4496919918	0	0.9694922822	0.9440415194	0.9834683877	4
TNBC	3.4825462012	1	0.9694922822			4
TNBC	4.0410677618	0	0.9664242686	0.9401913116	0.9812650208	4
TNBC	4.2381930185	0	0.9633562551	0.9363749869	0.9790230592	4
TNBC	5.0924024641	0	0.9602882415	0.9325893705	0.9767467751	4
TNBC	5.3552361396	1	0.9602882415			4
TNBC	5.5852156057	1	0.9602882415			4
TNBC	5.9137577002	0	0.957200498	0.928798938	0.9744279594	4
TNBC	6.1437371663	0	0.9541127544	0.9250353759	0.9720808114	4
TNBC	6.5051334702	0	0.9510250109	0.9212964274	0.9697078626	4
TNBC	6.5708418891	0	0.9479372674	0.9175801421	0.9673112742	4
TNBC	6.5708418891	1	0.9479372674			4
TNBC	6.6694045175	1	0.9479372674			4
TNBC	6.8336755647	0	0.9448292763	0.9138531902	0.9648801115	4
TNBC	7.6550308008	0	0.9417212853	0.9101465817	0.9624284356	4
TNBC	8.0492813142	1	0.9417212853			4
TNBC	8.1149897331	1	0.9417212853			4
TNBC	8.2792607803	1	0.9417212853			4
TNBC	8.772073922	0	0.9385822143	0.9064113825	0.9599376244	4
TNBC	9.5934291581	0	0.9354431434	0.9026949916	0.9574285389	4
TNBC	10.05338809	0	0.9323040724	0.8989961555	0.9549023563	4
TNBC	10.184804928	0	0.9291650015	0.8953137635	0.9523601256	4
TNBC	10.283367556	1	0.9291650015			4
TNBC	10.349075975	1	0.9291650015			4
TNBC	10.414784394	1	0.9291650015			4
TNBC	10.743326489	1	0.9291650015			4
TNBC	10.973305955	1	0.9291650015			4
TNBC	11.466119097	0	0.9259719946	0.8915673335	0.9497666682	4
TNBC	11.531827515	0	0.9227789877	0.8878370162	0.9471582365	4
TNBC	11.696098563	0	0.9195859808	0.8841218579	0.9445356469	4
TNBC	11.761806982	0	0.9100069602	0.8730591972	0.936590001	4
TNBC	11.761806982	1	0.9100069602			4
TNBC	11.893223819	0	0.9068027104	0.8693810632	0.9339096781	4
TNBC	11.926078029	0	0.9035984605	0.865714784	0.9312181858	4
TNBC	12.024640657	1	0.9035984605			4
TNBC	12.747433265	0	0.9003828076	0.8620439245	0.9285079322	4
TNBC	12.813141684	1	0.9003828076			4
TNBC	13.108829569	0	0.8971556291	0.858368104	0.9257791269	4
TNBC	13.568788501	0	0.8939284506	0.8547030324	0.9230402803	4
TNBC	14.19301848	1	0.8939284506			4
TNBC	14.258726899	0	0.8906895794	0.8510322098	0.9202833818	4
TNBC	14.291581109	1	0.8906895794			4
TNBC	14.422997947	0	0.8874388875	0.8473553036	0.9175085671	4
TNBC	14.521560575	0	0.8841881956	0.8436882531	0.9147246125	4
TNBC	14.521560575	1	0.8841881956			4
TNBC	14.521560575	1	0.8841881956			4
TNBC	14.718685832	1	0.8841881956			4
TNBC	14.850102669	1	0.8841881956			4
TNBC	15.014373717	1	0.8841881956			4
TNBC	15.145790554	1	0.8841881956			4

TNBC	15.178644764	0	0.8808641798	0.8399310942	0.9118786187	4
TNBC	15.244353183	1	0.8808641798			4
TNBC	15.310061602	0	0.8775275731	0.8361670919	0.9090143648	4
TNBC	15.310061602	1	0.8775275731			4
TNBC	15.310061602	1	0.8775275731			4
TNBC	15.40862423	1	0.8775275731			4
TNBC	15.474332649	0	0.874152467	0.8323610155	0.9061131791	4
TNBC	15.474332649	1	0.874152467			4
TNBC	15.671457906	0	0.8707642792	0.8285475005	0.9031935292	4
TNBC	15.704312115	1	0.8707642792			4
TNBC	16.295687885	1	0.8707642792			4
TNBC	16.459958932	0	0.8673495173	0.8247082383	0.9002457064	4
TNBC	16.657084189	1	0.8673495173			4
TNBC	16.689938398	1	0.8673495173			4
TNBC	16.722792608	0	0.8639076541	0.8208426074	0.8972693557	4
TNBC	16.887063655	1	0.8639076541			4
TNBC	17.084188912	0	0.8604520235	0.8169685753	0.8942742895	4
TNBC	17.347022587	1	0.8604520235			4
TNBC	17.379876797	1	0.8604520235			4
TNBC	17.379876797	1	0.8604520235			4
TNBC	17.412731006	0	0.856954251	0.8130480833	0.8912397534	4
TNBC	17.412731006	1	0.856954251			4
TNBC	17.445585216	0	0.8534421435	0.8091185504	0.888186141	4
TNBC	17.511293634	1	0.8534421435			4
TNBC	17.741273101	1	0.8534421435			4
TNBC	17.77412731	0	0.8499008897	0.8051601521	0.8851026216	4
TNBC	17.839835729	1	0.8499008897			4
TNBC	18.069815195	1	0.8499008897			4
TNBC	18.299794661	1	0.8499008897			4
TNBC	18.464065708	1	0.8499008897			4
TNBC	18.496919918	1	0.8499008897			4
TNBC	18.661190965	1	0.8499008897			4
TNBC	18.792607803	1	0.8499008897			4
TNBC	18.891170431	0	0.8462532464	0.8010699651	0.8819322671	4
TNBC	19.055441478	0	0.8426056031	0.7969908191	0.8787530031	4
TNBC	19.121149897	0	0.8389579598	0.7929223034	0.8755650965	4
TNBC	19.219712526	1	0.8389579598			4
TNBC	19.58110883	1	0.8389579598			4
TNBC	19.778234086	0	0.8352783197	0.7888217291	0.8723450237	4
TNBC	19.811088296	1	0.8352783197			4
TNBC	20.008213552	1	0.8352783197			4
TNBC	20.271047228	1	0.8352783197			4
TNBC	20.369609856	1	0.8352783197			4
TNBC	20.369609856	1	0.8352783197			4
TNBC	20.435318275	1	0.8352783197			4
TNBC	20.468172485	0	0.8314987798	0.7845992142	0.8690424684	4
TNBC	20.533880903	1	0.8314987798			4
TNBC	20.599589322	1	0.8314987798			4
TNBC	20.599589322	1	0.8314987798			4
TNBC	20.632443532	1	0.8314987798			4

TNBC	20.632443532	1	0.8314987798			4
TNBC	20.829568789	1	0.8314987798			4
TNBC	20.862422998	1	0.8314987798			4
TNBC	20.895277207	0	0.8275950296	0.7802227676	0.8656394208	4
TNBC	21.256673511	0	0.8236912795	0.7758589228	0.8622266895	4
TNBC	21.420944559	1	0.8236912795			4
TNBC	22.242299795	0	0.81976894	0.7714825684	0.8587908179	4
TNBC	22.570841889	0	0.8158466006	0.7671181558	0.8553456676	4
TNBC	22.735112936	0	0.8119242612	0.7627652606	0.8518914981	4
TNBC	22.866529774	1	0.8119242612			4
TNBC	22.965092402	1	0.8119242612			4
TNBC	23.359342916	1	0.8119242612			4
TNBC	23.457905544	1	0.8119242612			4
TNBC	23.655030801	0	0.8079246343	0.7583219943	0.8483709054	4
TNBC	23.917864476	0	0.8039250074	0.7538904719	0.844841176	4
TNBC	24.049281314	0	0.7999253805	0.749470296	0.8413025532	4
TNBC	25.363449692	1	0.7999253805			4
TNBC	26.874743326	0	0.795905655	0.7450349295	0.8377401493	4
TNBC	27.26899384	0	0.7918859294	0.7406104334	0.8341691507	4
TNBC	27.26899384	1	0.7918859294			4
TNBC	27.400410678	0	0.7878456951	0.7361699356	0.8305742481	4
TNBC	27.433264887	0	0.7838054608	0.7317398902	0.8269710162	4
TNBC	27.433264887	1	0.7838054608			4
TNBC	28.747433265	0	0.7756831244	0.7228563191	0.8197083241	4
TNBC	29.273100616	0	0.7716219562	0.718429377	0.8160650186	4
TNBC	29.765913758	0	0.767560788	0.7140119681	0.8124139718	4
TNBC	29.963039014	1	0.767560788			4
TNBC	30.521560575	0	0.7634780179	0.7095763916	0.8087387048	4
TNBC	31.441478439	0	0.7593952477	0.7051500764	0.8050558765	4
TNBC	32.427104723	1	0.7593952477			4
TNBC	32.985626283	0	0.7552904085	0.7007048831	0.8013485575	4
TNBC	34.036960986	1	0.7552904085			4
TNBC	34.299794661	0	0.7511631385	0.6962403942	0.7976164515	4
TNBC	34.529774127	1	0.7511631385			4
TNBC	34.726899384	0	0.7470130659	0.6917561851	0.7938592503	4
TNBC	34.792607803	0	0.7428629934	0.6872810023	0.79009465	4
TNBC	34.858316222	1	0.7428629934			4
TNBC	36.205338809	1	0.7428629934			4
TNBC	36.271047228	1	0.7428629934			4
TNBC	36.829568789	1	0.7428629934			4
TNBC	37.092402464	0	0.738618062	0.6826953218	0.7862490618	4
TNBC	37.650924025	1	0.738618062			4
TNBC	37.782340862	1	0.738618062			4
TNBC	37.815195072	1	0.738618062			4
TNBC	38.078028747	0	0.7342986581	0.6780252637	0.7823380745	4
TNBC	38.899383984	1	0.7342986581			4
TNBC	39.392197125	1	0.7342986581			4
TNBC	39.490759754	1	0.7342986581			4
TNBC	39.72073922	1	0.7342986581			4
TNBC	39.852156057	1	0.7342986581			4

TNBC	40.147843943	1	0.7342986581			4
TNBC	41.100616016	0	0.7298212273	0.6731651191	0.7782973455	4
TNBC	41.49486653	0	0.7253437964	0.6683164412	0.7742476368	4
TNBC	42.151950719	1	0.7253437964			4
TNBC	42.447638604	1	0.7253437964			4
TNBC	42.710472279	1	0.7253437964			4
TNBC	42.874743326	1	0.7253437964			4
TNBC	43.203285421	0	0.7207530129	0.6633351191	0.7701021474	4
TNBC	43.761806982	1	0.7207530129			4
TNBC	44.320328542	1	0.7207530129			4
TNBC	45.04312115	0	0.7161029935	0.6582906371	0.7659019017	4
TNBC	45.174537988	0	0.711452974	0.6532586475	0.7616919724	4
TNBC	45.404517454	1	0.711452974			4
TNBC	45.667351129	1	0.711452974			4
TNBC	45.831622177	1	0.711452974			4
TNBC	45.930184805	1	0.711452974			4
TNBC	45.930184805	1	0.711452974			4
TNBC	45.930184805	1	0.711452974			4
TNBC	46.390143737	1	0.711452974			4
TNBC	46.718685832	1	0.711452974			4
TNBC	47.211498973	1	0.711452974			4
TNBC	47.441478439	1	0.711452974			4
TNBC	47.967145791	1	0.711452974			4
TNBC	48.361396304	1	0.711452974			4
TNBC	48.459958932	1	0.711452974			4
TNBC	48.624229979	1	0.711452974			4
TNBC	49.117043121	1	0.711452974			4
TNBC	49.609856263	1	0.711452974			4
TNBC	49.80698152	1	0.711452974			4
TNBC	49.971252567	0	0.7062217021	0.6474907447	0.7570354244	4
TNBC	50.858316222	1	0.7062217021			4
TNBC	51.613963039	1	0.7062217021			4
TNBC	52.369609856	1	0.7062217021			4
TNBC	52.599589322	1	0.7062217021			4
TNBC	52.928131417	1	0.7062217021			4
TNBC	54.702258727	0	0.7007892275	0.6414786017	0.7522170038	4
TNBC	56.049281314	1	0.7007892275			4
TNBC	56.049281314	1	0.7007892275			4
TNBC	56.27926078	1	0.7007892275			4
TNBC	57.067761807	1	0.7007892275			4
TNBC	57.199178645	1	0.7007892275			4
TNBC	58.513347023	1	0.7007892275			4
TNBC	58.940451745	1	0.7007892275			4
TNBC	58.973305955	1	0.7007892275			4
TNBC	60.090349076	1	0.7007892275			4
TNBC	60.878850103	1	0.7007892275			4
TNBC	61.273100616	1	0.7007892275			4
TNBC	62.620123203	0	0.6948503358	0.6348146669	0.7470191339	4
TNBC	63.047227926	1	0.6948503358			4
TNBC	64.032854209	1	0.6948503358			4

TNBC	64.689938398	1	0.6948503358			4
TNBC	65.215605749	1	0.6948503358			4
TNBC	65.609856263	1	0.6948503358			4
TNBC	66.858316222	1	0.6948503358			4
TNBC	66.98973306	1	0.6948503358			4
TNBC	67.646817248	0	0.6885335145	0.6276694112	0.7415355641	4
TNBC	67.646817248	1	0.6885335145			4
TNBC	67.778234086	1	0.6885335145			4
TNBC	68.698151951	1	0.6885335145			4
TNBC	69.289527721	1	0.6885335145			4
TNBC	69.552361396	1	0.6885335145			4
TNBC	71.425051335	1	0.6885335145			4
TNBC	71.425051335	1	0.6885335145			4
TNBC	72.246406571	1	0.6885335145			4
TNBC	72.27926078	1	0.6885335145			4
TNBC	72.509240246	1	0.6885335145			4
TNBC	72.574948665	1	0.6885335145			4
TNBC	72.739219713	1	0.6885335145			4
TNBC	72.772073922	1	0.6885335145			4
TNBC	72.969199179	1	0.6885335145			4
TNBC	73.297741273	1	0.6885335145			4
TNBC	73.691991786	1	0.6885335145			4
TNBC	74.414784394	1	0.6885335145			4
TNBC	74.67761807	1	0.6885335145			4
TNBC	75.301848049	1	0.6885335145			4
TNBC	75.696098563	1	0.6885335145			4
TNBC	76.287474333	1	0.6885335145			4
TNBC	76.484599589	1	0.6885335145			4
TNBC	77.535934292	1	0.6885335145			4
TNBC	77.765913758	1	0.6885335145			4
TNBC	79.40862423	1	0.6885335145			4
TNBC	79.507186858	1	0.6885335145			4
TNBC	79.967145791	1	0.6885335145			4
TNBC	80.197125257	1	0.6885335145			4
TNBC	80.558521561	1	0.6885335145			4
TNBC	81.117043121	1	0.6885335145			4
TNBC	81.708418891	1	0.6885335145			4
TNBC	81.77412731	1	0.6885335145			4
TNBC	82.924024641	1	0.6885335145			4
TNBC	85.158110883	0	0.679473863	0.6165731216	0.7343212721	4
TNBC	85.158110883	1	0.679473863			4
TNBC	86.800821355	1	0.679473863			4
TNBC	87.030800821	1	0.679473863			4
TNBC	87.260780287	1	0.679473863			4
TNBC	88.607802875	1	0.679473863			4
TNBC	88.706365503	1	0.679473863			4
TNBC	89.034907598	1	0.679473863			4
TNBC	90.447638604	1	0.679473863			4
TNBC	90.743326489	1	0.679473863			4
TNBC	91.86036961	1	0.679473863			4

TNBC	93.04312115	1	0.679473863			4
TNBC	93.174537988	1	0.679473863			4
TNBC	93.207392197	1	0.679473863			4
TNBC	93.930184805	1	0.679473863			4
TNBC	95.014373717	1	0.679473863			4
TNBC	95.540041068	1	0.679473863			4
TNBC	95.835728953	1	0.679473863			4
TNBC	96.525667351	1	0.679473863			4
TNBC	97.77412731	1	0.679473863			4
TNBC	98.825462012	1	0.679473863			4
TNBC	100.07392197	0	0.6671197928	0.6002713279	0.7253926573	4
TNBC	100.07392197	1				4
TNBC	100.56673511	1				4
TNBC	100.76386037	1				4
TNBC	101.25667351	1				4
TNBC	102.89938398	1				4
TNBC	102.9650924	1				4
TNBC	103.19507187	1				4
TNBC	104.7063655	1				4
TNBC	105.26488706	1				4
TNBC	105.65913758	1				4
TNBC	105.95482546	1				4
TNBC	106.34907598	1				4
TNBC	106.34907598	1				4
TNBC	106.80903491	1				4
TNBC	107.30184805	1				4
TNBC	107.79466119	1				4
TNBC	108.81314168	1				4
TNBC	109.07597536	1				4
TNBC	109.14168378	1				4
TNBC	110.91581109	1				4
TNBC	111.44147844	1				4
TNBC	112.13141684	1				4
TNBC	112.62422998	1				4
TNBC	112.85420945	1				4
TNBC	113.11704312	1				4
TNBC	113.21560575	1				4
TNBC	114.13552361	1				4
TNBC	114.46406571	1				4
TNBC	114.82546201	1				4
TNBC	115.25256674	1				4
TNBC	115.84394251	1				4
TNBC	116.04106776	1				4
TNBC	116.5338809	1				4
TNBC	117.09240246	1				4
TNBC	118.11088296	1				4
TNBC	118.275154	1				4
TNBC	119.52361396	1				4
TNBC	119.85215606	1				4
TNBC	119.85215606	1				4

TNBC	119.9835729	1				4
TNBC	120.11498973	1				4
TNBC	121.0349076	1				4
TNBC	121.13347023	1				4
TNBC	121.33059548	1				4
TNBC	121.82340862	1				4
TNBC	122.41478439	1				4
TNBC	122.84188912	1				4
TNBC	123.43326489	1				4
TNBC	123.89322382	1				4
TNBC	126.55441478	1				4
TNBC	126.81724846	1				4
TNBC	127.70431211	1				4
TNBC	128.82135524	1				4
TNBC	129.18275154	1				4
