# Supplementary material for: Overall survival of triple negative breast cancer in French Caribbean women
Source: PLoS One. 2022 Aug 24;17(8):e0271966. doi: 10.1371/journal.pone.0271966 (PMC9401158; doi:10.1371/journal.pone.0271966)
Supplement: S1 Data — (ZIP) [file pone.0271966.s001.zip › survie_recept_stade.pdf]

RECEPTEUR	stade2	Time(month)	CENSOR	SURVIVAL	Survival LCL	Survival UCL	STRATUM
	Localized/ local spread	0		1	1	1	1
	Localized/ local spread	0	1	1			1
	Localized/ local spread	0.2628336756	1	1			1
	Localized/ local spread	0.295687885	1	1			1
	Localized/ local spread	1.4127310062	1	1			1
	Localized/ local spread	2.3983572895	1	1			1
	Localized/ local spread	2.8583162218	1	1			1
	Localized/ local spread	3.1211498973	0	0.9836065574	0.8892780611	0.9976743852	1
	Localized/ local spread	4.7967145791	0	0.9508196721	0.8552314819	0.9838684498	1
	Localized/ local spread	5.0266940452	0	0.9344262295	0.8346514782	0.974870877	1
	Localized/ local spread	5.8151950719	1	0.9344262295			1
	Localized/ local spread	7.8521560575	0	0.9177400468	0.8135736154	0.9649151778	1
	Localized/ local spread	8.9691991786	1	0.9177400468			1
	Localized/ local spread	10.973305955	0	0.9007448608	0.7922748251	0.9541554563	1
	Localized/ local spread	11.039014374	1	0.9007448608			1
	Localized/ local spread	12.024640657	0	0.8834228442	0.7708233327	0.9426833675	1
	Localized/ local spread	12.057494867	0	0.8661008277	0.7498669188	0.9307270976	1
	Localized/ local spread	22.702258727	0	0.8487788111	0.7293305861	0.918356926	1
	Localized/ local spread	28.090349076	0	0.8314567946	0.7091599842	0.9056253327	1
	Localized/ local spread	41.889117043	1	0.8314567946			1
	Localized/ local spread	44.320328542	1	0.8314567946			1
	Localized/ local spread	44.911704312	0	0.8133816469	0.6880647949	0.8921542258	1
	Localized/ local spread	45.240246407	0	0.7953064992	0.6673566082	0.878361019	1
	Localized/ local spread	47.178644764	1	0.7953064992			1
	Localized/ local spread	47.868583162	1	0.7953064992			1
	Localized/ local spread	49.379876797	1	0.7953064992			1
	Localized/ local spread	50.891170431	1	0.7953064992			1
	Localized/ local spread	50.924024641	1	0.7953064992			1
	Localized/ local spread	51.876796715	1	0.7953064992			1
	Localized/ local spread	52.468172485	1	0.7953064992			1
	Localized/ local spread	52.566735113	1	0.7953064992			1
	Localized/ local spread	52.960985626	1	0.7953064992			1
	Localized/ local spread	53.453798768	1	0.7953064992			1
	Localized/ local spread	55.950718686	1	0.7953064992			1
	Localized/ local spread	56.772073922	0	0.7712063022	0.6367390027	0.8611153707	1
	Localized/ local spread	58.907597536	1	0.7712063022			1
	Localized/ local spread	58.940451745	1	0.7712063022			1
	Localized/ local spread	62.981519507	0	0.7454994255	0.6045699227	0.8424757333	1
	Localized/ local spread	65.117043121	1	0.7454994255			1
	Localized/ local spread	65.708418891	1	0.7454994255			1
	Localized/ local spread	68.1724846	1	0.7454994255			1
	Localized/ local spread	68.435318275	0	0.7168263706	0.5685880348	0.8217569309	1
	Localized/ local spread	70.176591376	1	0.7168263706			1
	Localized/ local spread	72.114989733	0	0.6869586052	0.5323056777	0.7996374514	1
	Localized/ local spread	72.147843943	1	0.6869586052			1
	Localized/ local spread	73.297741273	1	0.6869586052			1
	Localized/ local spread	74.316221766	1	0.6869586052			1
	Localized/ local spread	75.696098563	1	0.6869586052			1
	Localized/ local spread	75.794661191	0	0.6508028891	0.4869552355	0.7738207794	1

	Localized/ local spread	76.648870637	1				1
	Localized/ local spread	77.108829569	1				1
	Localized/ local spread	77.798767967	1				1
	Localized/ local spread	82.496919918	1				1
	Localized/ local spread	83.318275154	1				1
	Localized/ local spread	86.110882957	1				1
	Localized/ local spread	89.527720739	1				1
	Localized/ local spread	89.527720739	1				1
	Localized/ local spread	96.394250513	1				1
	Localized/ local spread	98.102669405	1				1
	Localized/ local spread	101.35523614	1				1
	Localized/ local spread	110.68583162	1				1
	Localized/ local spread	110.91581109	1				1
	Localized/ local spread	112.59137577	1				1
	Localized/ local spread	112.85420945	1				1
	Localized/ local spread	115.02258727	1				1
	Localized/ local spread	115.38398357	1				1
	Localized/ local spread	116.23819302	1				1
	Metastatic	0		1	1	1	2
	Metastatic	0.0328542094	0	0.875	0.387000014	0.9813929659	2
	Metastatic	0.4599589322	1	0.875			2
	Metastatic	0.9856262834	0	0.7291666667	0.2763902203	0.9253531813	2
	Metastatic	2.6283367556	0	0.5833333333	0.180188891	0.8440686917	2
	Metastatic	5.3223819302	1	0.5833333333			2
	Metastatic	27.794661191	0	0.3888888889	0.0630028115	0.7242227555	2
	Metastatic	54.669404517	1	0.3888888889			2
	Metastatic	79.638603696	0	0			2
	Regional	0		1	1	1	3
	Regional	6.1765913758	0	0.8888888889	0.4329650902	0.9835640285	3
	Regional	15.047227926	1	0.8888888889			3
	Regional	23.293634497	0	0.7619047619	0.3321725136	0.9351042487	3
	Regional	35.679671458	1	0.7619047619			3
	Regional	48.919917864	1	0.7619047619			3
	Regional	63.310061602	0	0.5714285714	0.1506812928	0.8474938362	3
	Regional	93.437371663	1	0.5714285714			3
	Regional	111.44147844	0	0.2857142857	0.0135884171	0.6941241662	3
	Regional	114.16837782	1				3
HR+/HER2-	Localized/ local spread	0		1	1	1	1
HR+/HER2-	Localized/ local spread	0	1	1			1
HR+/HER2-	Localized/ local spread	0	1	1			1
HR+/HER2-	Localized/ local spread	0	1	1			1
HR+/HER2-	Localized/ local spread	0.3613963039	1	1			1
HR+/HER2-	Localized/ local spread	0.3942505133	1	1			1
HR+/HER2-	Localized/ local spread	0.4599589322	1	1			1
HR+/HER2-	Localized/ local spread	0.6899383984	1	1			1
HR+/HER2-	Localized/ local spread	0.6899383984	1	1			1
HR+/HER2-	Localized/ local spread	0.7885010267	1	1			1
HR+/HER2-	Localized/ local spread	1.0841889117	1	1			1
HR+/HER2-	Localized/ local spread	1.4127310062	1	1			1
HR+/HER2-	Localized/ local spread	2.0698151951	1	1			1

HR+/HER2-	Localized/ local spread	2.4640657084	1	1			1
HR+/HER2-	Localized/ local spread	2.5626283368	0	0.9981060606	0.9866321903	0.9997329959	1
HR+/HER2-	Localized/ local spread	2.6283367556	1	0.9981060606			1
HR+/HER2-	Localized/ local spread	2.6611909651	1	0.9981060606			1
HR+/HER2-	Localized/ local spread	3.022587269	1	0.9981060606			1
HR+/HER2-	Localized/ local spread	3.7125256674	1	0.9981060606			1
HR+/HER2-	Localized/ local spread	4.8952772074	0	0.9942892114	0.9823990366	0.9981546003	1
HR+/HER2-	Localized/ local spread	5.158110883	1	0.9942892114			1
HR+/HER2-	Localized/ local spread	5.4209445585	0	0.9923771168	0.979817843	0.9971322004	1
HR+/HER2-	Localized/ local spread	5.8151950719	1	0.9923771168			1
HR+/HER2-	Localized/ local spread	5.8809034908	1	0.9923771168			1
HR+/HER2-	Localized/ local spread	6.0780287474	1	0.9923771168			1
HR+/HER2-	Localized/ local spread	6.4722792608	1	0.9923771168			1
HR+/HER2-	Localized/ local spread	6.5708418891	1	0.9923771168			1
HR+/HER2-	Localized/ local spread	6.7679671458	0	0.990446422	0.9772000568	0.9960125241	1
HR+/HER2-	Localized/ local spread	6.9650924025	1	0.990446422			1
HR+/HER2-	Localized/ local spread	7.3264887064	1	0.990446422			1
HR+/HER2-	Localized/ local spread	7.3264887064	1	0.990446422			1
HR+/HER2-	Localized/ local spread	7.5893223819	1	0.990446422			1
HR+/HER2-	Localized/ local spread	8.0492813142	1	0.990446422			1
HR+/HER2-	Localized/ local spread	8.0492813142	1	0.990446422			1
HR+/HER2-	Localized/ local spread	9.2320328542	1	0.990446422			1
HR+/HER2-	Localized/ local spread	9.7577002053	1	0.990446422			1
HR+/HER2-	Localized/ local spread	9.8234086242	1	0.990446422			1
HR+/HER2-	Localized/ local spread	9.954825462	1	0.990446422			1
HR+/HER2-	Localized/ local spread	10.020533881	1	0.990446422			1
HR+/HER2-	Localized/ local spread	10.480492813	1	0.990446422			1
HR+/HER2-	Localized/ local spread	10.480492813	1	0.990446422			1
HR+/HER2-	Localized/ local spread	10.480492813	1	0.990446422			1
HR+/HER2-	Localized/ local spread	10.513347023	1	0.990446422			1
HR+/HER2-	Localized/ local spread	10.513347023	1	0.990446422			1
HR+/HER2-	Localized/ local spread	10.710472279	1	0.990446422			1
HR+/HER2-	Localized/ local spread	10.907597536	1	0.990446422			1
HR+/HER2-	Localized/ local spread	11.071868583	1	0.990446422			1
HR+/HER2-	Localized/ local spread	11.071868583	1	0.990446422			1
HR+/HER2-	Localized/ local spread	11.170431211	1	0.990446422			1
HR+/HER2-	Localized/ local spread	11.170431211	1	0.990446422			1
HR+/HER2-	Localized/ local spread	11.433264887	1	0.990446422			1
HR+/HER2-	Localized/ local spread	11.761806982	1	0.990446422			1
HR+/HER2-	Localized/ local spread	11.794661191	1	0.990446422			1
HR+/HER2-	Localized/ local spread	11.893223819	1	0.990446422			1
HR+/HER2-	Localized/ local spread	12.221765914	0	0.9884126511	0.9743835094	0.994779089	1
HR+/HER2-	Localized/ local spread	12.353182752	1	0.9884126511			1
HR+/HER2-	Localized/ local spread	12.681724846	1	0.9884126511			1
HR+/HER2-	Localized/ local spread	12.714579055	1	0.9884126511			1
HR+/HER2-	Localized/ local spread	13.01026694	1	0.9884126511			1
HR+/HER2-	Localized/ local spread	13.01026694	1	0.9884126511			1
HR+/HER2-	Localized/ local spread	13.04312115	1	0.9884126511			1
HR+/HER2-	Localized/ local spread	13.108829569	1	0.9884126511			1
HR+/HER2-	Localized/ local spread	13.207392197	1	0.9884126511			1

HR+/HER2-	Localized/ local spread	13.273100616	1	0.9884126511			1
HR+/HER2-	Localized/ local spread	13.765913758	0	0.9863405072	0.9715479987	0.9934679796	1
HR+/HER2-	Localized/ local spread	14.028747433	1	0.9863405072			1
HR+/HER2-	Localized/ local spread	14.061601643	0	0.9842640009	0.9687628553	0.9921040693	1
HR+/HER2-	Localized/ local spread	14.094455852	1	0.9842640009			1
HR+/HER2-	Localized/ local spread	14.094455852	1	0.9842640009			1
HR+/HER2-	Localized/ local spread	14.291581109	1	0.9842640009			1
HR+/HER2-	Localized/ local spread	14.324435318	1	0.9842640009			1
HR+/HER2-	Localized/ local spread	14.324435318	1	0.9842640009			1
HR+/HER2-	Localized/ local spread	14.422997947	1	0.9842640009			1
HR+/HER2-	Localized/ local spread	14.488706366	1	0.9842640009			1
HR+/HER2-	Localized/ local spread	14.751540041	1	0.9842640009			1
HR+/HER2-	Localized/ local spread	14.784394251	1	0.9842640009			1
HR+/HER2-	Localized/ local spread	14.850102669	1	0.9842640009			1
HR+/HER2-	Localized/ local spread	15.277207392	1	0.9842640009			1
HR+/HER2-	Localized/ local spread	15.277207392	1	0.9842640009			1
HR+/HER2-	Localized/ local spread	15.342915811	1	0.9842640009			1
HR+/HER2-	Localized/ local spread	15.375770021	1	0.9842640009			1
HR+/HER2-	Localized/ local spread	15.441478439	0	0.9821242965	0.9659008056	0.9906662266	1
HR+/HER2-	Localized/ local spread	15.441478439	1	0.9821242965			1
HR+/HER2-	Localized/ local spread	15.540041068	1	0.9821242965			1
HR+/HER2-	Localized/ local spread	15.770020534	1	0.9821242965			1
HR+/HER2-	Localized/ local spread	15.868583162	1	0.9821242965			1
HR+/HER2-	Localized/ local spread	15.934291581	1	0.9821242965			1
HR+/HER2-	Localized/ local spread	16.229979466	1	0.9821242965			1
HR+/HER2-	Localized/ local spread	16.295687885	1	0.9821242965			1
HR+/HER2-	Localized/ local spread	16.328542094	1	0.9821242965			1
HR+/HER2-	Localized/ local spread	16.328542094	1	0.9821242965			1
HR+/HER2-	Localized/ local spread	16.361396304	1	0.9821242965			1
HR+/HER2-	Localized/ local spread	16.361396304	1	0.9821242965			1
HR+/HER2-	Localized/ local spread	16.459958932	0	0.9799320548	0.9629870268	0.9891629015	1
HR+/HER2-	Localized/ local spread	16.459958932	1	0.9799320548			1
HR+/HER2-	Localized/ local spread	16.492813142	1	0.9799320548			1
HR+/HER2-	Localized/ local spread	16.492813142	1	0.9799320548			1
HR+/HER2-	Localized/ local spread	16.525667351	0	0.9777250006	0.9600934421	0.9876166026	1
HR+/HER2-	Localized/ local spread	16.525667351	1	0.9777250006			1
HR+/HER2-	Localized/ local spread	16.657084189	1	0.9777250006			1
HR+/HER2-	Localized/ local spread	16.689938398	1	0.9777250006			1
HR+/HER2-	Localized/ local spread	16.755646817	0	0.9755028983	0.9572139337	0.9860310358	1
HR+/HER2-	Localized/ local spread	16.854209446	1	0.9755028983			1
HR+/HER2-	Localized/ local spread	16.854209446	1	0.9755028983			1
HR+/HER2-	Localized/ local spread	16.952772074	1	0.9755028983			1
HR+/HER2-	Localized/ local spread	17.084188912	1	0.9755028983			1
HR+/HER2-	Localized/ local spread	17.149897331	1	0.9755028983			1
HR+/HER2-	Localized/ local spread	17.248459959	1	0.9755028983			1
HR+/HER2-	Localized/ local spread	17.445585216	1	0.9755028983			1
HR+/HER2-	Localized/ local spread	17.478439425	1	0.9755028983			1
HR+/HER2-	Localized/ local spread	17.642710472	1	0.9755028983			1
HR+/HER2-	Localized/ local spread	17.839835729	1	0.9755028983			1
HR+/HER2-	Localized/ local spread	17.839835729	1	0.9755028983			1

HR+/HER2-	Localized/ local spread	17.872689938	1	0.9755028983			1
HR+/HER2-	Localized/ local spread	18.036960986	1	0.9755028983			1
HR+/HER2-	Localized/ local spread	18.036960986	1	0.9755028983			1
HR+/HER2-	Localized/ local spread	18.102669405	1	0.9755028983			1
HR+/HER2-	Localized/ local spread	18.201232033	1	0.9755028983			1
HR+/HER2-	Localized/ local spread	18.234086242	1	0.9755028983			1
HR+/HER2-	Localized/ local spread	18.299794661	1	0.9755028983			1
HR+/HER2-	Localized/ local spread	18.36550308	0	0.9731857893	0.954200673	0.9843652585	1
HR+/HER2-	Localized/ local spread	18.36550308	1	0.9731857893			1
HR+/HER2-	Localized/ local spread	18.529774127	1	0.9731857893			1
HR+/HER2-	Localized/ local spread	18.661190965	1	0.9731857893			1
HR+/HER2-	Localized/ local spread	18.694045175	1	0.9731857893			1
HR+/HER2-	Localized/ local spread	18.726899384	1	0.9731857893			1
HR+/HER2-	Localized/ local spread	18.858316222	1	0.9731857893			1
HR+/HER2-	Localized/ local spread	18.924024641	1	0.9731857893			1
HR+/HER2-	Localized/ local spread	19.022587269	1	0.9731857893			1
HR+/HER2-	Localized/ local spread	19.154004107	1	0.9731857893			1
HR+/HER2-	Localized/ local spread	19.186858316	1	0.9731857893			1
HR+/HER2-	Localized/ local spread	19.219712526	1	0.9731857893			1
HR+/HER2-	Localized/ local spread	19.351129363	1	0.9731857893			1
HR+/HER2-	Localized/ local spread	19.416837782	1	0.9731857893			1
HR+/HER2-	Localized/ local spread	19.482546201	1	0.9731857893			1
HR+/HER2-	Localized/ local spread	19.482546201	1	0.9731857893			1
HR+/HER2-	Localized/ local spread	19.646817248	1	0.9731857893			1
HR+/HER2-	Localized/ local spread	19.843942505	1	0.9731857893			1
HR+/HER2-	Localized/ local spread	20.041067762	1	0.9731857893			1
HR+/HER2-	Localized/ local spread	20.1724846	1	0.9731857893			1
HR+/HER2-	Localized/ local spread	20.271047228	1	0.9731857893			1
HR+/HER2-	Localized/ local spread	20.271047228	1	0.9731857893			1
HR+/HER2-	Localized/ local spread	20.271047228	1	0.9731857893			1
HR+/HER2-	Localized/ local spread	20.303901437	1	0.9731857893			1
HR+/HER2-	Localized/ local spread	20.599589322	1	0.9731857893			1
HR+/HER2-	Localized/ local spread	20.632443532	1	0.9731857893			1
HR+/HER2-	Localized/ local spread	20.862422998	1	0.9731857893			1
HR+/HER2-	Localized/ local spread	20.895277207	1	0.9731857893			1
HR+/HER2-	Localized/ local spread	20.928131417	0	0.9707094896	0.9509422668	0.9825843968	1
HR+/HER2-	Localized/ local spread	20.993839836	1	0.9707094896			1
HR+/HER2-	Localized/ local spread	21.158110883	1	0.9707094896			1
HR+/HER2-	Localized/ local spread	21.158110883	1	0.9707094896			1
HR+/HER2-	Localized/ local spread	21.256673511	1	0.9707094896			1
HR+/HER2-	Localized/ local spread	21.486652977	0	0.968207661	0.9476880151	0.9807597571	1
HR+/HER2-	Localized/ local spread	21.585215606	1	0.968207661			1
HR+/HER2-	Localized/ local spread	21.650924025	1	0.968207661			1
HR+/HER2-	Localized/ local spread	21.683778234	1	0.968207661			1
HR+/HER2-	Localized/ local spread	21.683778234	1	0.968207661			1
HR+/HER2-	Localized/ local spread	21.880903491	1	0.968207661			1
HR+/HER2-	Localized/ local spread	21.880903491	1	0.968207661			1
HR+/HER2-	Localized/ local spread	21.9137577	1	0.968207661			1
HR+/HER2-	Localized/ local spread	21.9137577	1	0.968207661			1
HR+/HER2-	Localized/ local spread	21.979466119	1	0.968207661			1

HR+/HER2-	Localized/ local spread	22.078028747	1	0.968207661			1
HR+/HER2-	Localized/ local spread	22.143737166	1	0.968207661			1
HR+/HER2-	Localized/ local spread	22.308008214	1	0.968207661			1
HR+/HER2-	Localized/ local spread	22.308008214	1	0.968207661			1
HR+/HER2-	Localized/ local spread	22.308008214	1	0.968207661			1
HR+/HER2-	Localized/ local spread	22.373716632	1	0.968207661			1
HR+/HER2-	Localized/ local spread	22.570841889	1	0.968207661			1
HR+/HER2-	Localized/ local spread	22.603696099	1	0.968207661			1
HR+/HER2-	Localized/ local spread	22.669404517	1	0.968207661			1
HR+/HER2-	Localized/ local spread	22.767967146	1	0.968207661			1
HR+/HER2-	Localized/ local spread	22.800821355	0	0.9655766619	0.944250724	0.9788357944	1
HR+/HER2-	Localized/ local spread	23.12936345	1	0.9655766619			1
HR+/HER2-	Localized/ local spread	23.227926078	0	0.9629384743	0.9408497839	0.9768794639	1
HR+/HER2-	Localized/ local spread	23.359342916	1	0.9629384743			1
HR+/HER2-	Localized/ local spread	23.392197125	0	0.960293039	0.9374788561	0.974893302	1
HR+/HER2-	Localized/ local spread	23.490759754	0	0.9576476036	0.934145147	0.9728837274	1
HR+/HER2-	Localized/ local spread	23.655030801	1	0.9576476036			1
HR+/HER2-	Localized/ local spread	24.443531828	1	0.9576476036			1
HR+/HER2-	Localized/ local spread	24.542094456	1	0.9576476036			1
HR+/HER2-	Localized/ local spread	25.49486653	0	0.9549800615	0.9308078228	0.9708395436	1
HR+/HER2-	Localized/ local spread	25.527720739	1	0.9549800615			1
HR+/HER2-	Localized/ local spread	25.560574949	1	0.9549800615			1
HR+/HER2-	Localized/ local spread	25.626283368	1	0.9549800615			1
HR+/HER2-	Localized/ local spread	25.889117043	0	0.9522899768	0.9274642065	0.9687615809	1
HR+/HER2-	Localized/ local spread	26.217659138	0	0.9495998922	0.9241487352	0.9666646584	1
HR+/HER2-	Localized/ local spread	27.071868583	1	0.9495998922			1
HR+/HER2-	Localized/ local spread	27.531827515	1	0.9495998922			1
HR+/HER2-	Localized/ local spread	28.45174538	1	0.9495998922			1
HR+/HER2-	Localized/ local spread	28.878850103	0	0.9468867496	0.9208222031	0.9645357897	1
HR+/HER2-	Localized/ local spread	29.207392197	0	0.9441736071	0.9175195801	0.962390208	1
HR+/HER2-	Localized/ local spread	29.535934292	1	0.9441736071			1
HR+/HER2-	Localized/ local spread	29.897330595	1	0.9441736071			1
HR+/HER2-	Localized/ local spread	30.751540041	1	0.9441736071			1
HR+/HER2-	Localized/ local spread	32.755646817	1	0.9441736071			1
HR+/HER2-	Localized/ local spread	33.741273101	1	0.9441736071			1
HR+/HER2-	Localized/ local spread	33.872689938	1	0.9441736071			1
HR+/HER2-	Localized/ local spread	33.938398357	1	0.9441736071			1
HR+/HER2-	Localized/ local spread	33.938398357	1	0.9441736071			1
HR+/HER2-	Localized/ local spread	34.135523614	1	0.9441736071			1
HR+/HER2-	Localized/ local spread	34.135523614	1	0.9441736071			1
HR+/HER2-	Localized/ local spread	34.39835729	1	0.9441736071			1
HR+/HER2-	Localized/ local spread	34.39835729	1	0.9441736071			1
HR+/HER2-	Localized/ local spread	34.562628337	1	0.9441736071			1
HR+/HER2-	Localized/ local spread	34.595482546	0	0.9413551784	0.9140754188	0.9601621868	1
HR+/HER2-	Localized/ local spread	35.219712526	0	0.9385367497	0.9106553169	0.9579177935	1
HR+/HER2-	Localized/ local spread	35.219712526	1	0.9385367497			1
HR+/HER2-	Localized/ local spread	35.252566735	1	0.9385367497			1
HR+/HER2-	Localized/ local spread	35.515400411	0	0.9357012912	0.907231172	0.9556471155	1
HR+/HER2-	Localized/ local spread	35.515400411	1	0.9357012912			1
HR+/HER2-	Localized/ local spread	35.515400411	1	0.9357012912			1

HR+/HER2-	Localized/ local spread	35.515400411	1	0.9357012912			1
HR+/HER2-	Localized/ local spread	35.58110883	1	0.9357012912			1
HR+/HER2-	Localized/ local spread	35.679671458	1	0.9357012912			1
HR+/HER2-	Localized/ local spread	35.712525667	1	0.9357012912			1
HR+/HER2-	Localized/ local spread	35.909650924	1	0.9357012912			1
HR+/HER2-	Localized/ local spread	36.008213552	1	0.9357012912			1
HR+/HER2-	Localized/ local spread	36.008213552	1	0.9357012912			1
HR+/HER2-	Localized/ local spread	36.435318275	1	0.9357012912			1
HR+/HER2-	Localized/ local spread	36.698151951	1	0.9357012912			1
HR+/HER2-	Localized/ local spread	37.158110883	1	0.9357012912			1
HR+/HER2-	Localized/ local spread	37.519507187	1	0.9357012912			1
HR+/HER2-	Localized/ local spread	37.618069815	0	0.9327495522	0.9036505905	0.9532867552	1
HR+/HER2-	Localized/ local spread	37.815195072	1	0.9327495522			1
HR+/HER2-	Localized/ local spread	38.702258727	1	0.9327495522			1
HR+/HER2-	Localized/ local spread	38.702258727	1	0.9327495522			1
HR+/HER2-	Localized/ local spread	39.162217659	1	0.9327495522			1
HR+/HER2-	Localized/ local spread	39.195071869	1	0.9327495522			1
HR+/HER2-	Localized/ local spread	39.227926078	1	0.9327495522			1
HR+/HER2-	Localized/ local spread	39.359342916	1	0.9327495522			1
HR+/HER2-	Localized/ local spread	39.392197125	1	0.9327495522			1
HR+/HER2-	Localized/ local spread	39.490759754	1	0.9327495522			1
HR+/HER2-	Localized/ local spread	39.589322382	1	0.9327495522			1
HR+/HER2-	Localized/ local spread	39.68788501	1	0.9327495522			1
HR+/HER2-	Localized/ local spread	39.72073922	1	0.9327495522			1
HR+/HER2-	Localized/ local spread	39.852156057	1	0.9327495522			1
HR+/HER2-	Localized/ local spread	40.114989733	1	0.9327495522			1
HR+/HER2-	Localized/ local spread	40.180698152	1	0.9327495522			1
HR+/HER2-	Localized/ local spread	40.180698152	1	0.9327495522			1
HR+/HER2-	Localized/ local spread	40.344969199	1	0.9327495522			1
HR+/HER2-	Localized/ local spread	40.509240246	1	0.9327495522			1
HR+/HER2-	Localized/ local spread	40.574948665	1	0.9327495522			1
HR+/HER2-	Localized/ local spread	40.772073922	1	0.9327495522			1
HR+/HER2-	Localized/ local spread	40.804928131	1	0.9327495522			1
HR+/HER2-	Localized/ local spread	40.837782341	1	0.9327495522			1
HR+/HER2-	Localized/ local spread	40.837782341	1	0.9327495522			1
HR+/HER2-	Localized/ local spread	40.936344969	0	0.9295661066	0.8997359849	0.9507635336	1
HR+/HER2-	Localized/ local spread	41.264887064	1	0.9295661066			1
HR+/HER2-	Localized/ local spread	41.330595483	1	0.9295661066			1
HR+/HER2-	Localized/ local spread	41.527720739	1	0.9295661066			1
HR+/HER2-	Localized/ local spread	41.691991786	1	0.9295661066			1
HR+/HER2-	Localized/ local spread	41.691991786	1	0.9295661066			1
HR+/HER2-	Localized/ local spread	41.757700205	1	0.9295661066			1
HR+/HER2-	Localized/ local spread	41.954825462	1	0.9295661066			1
HR+/HER2-	Localized/ local spread	42.184804928	1	0.9295661066			1
HR+/HER2-	Localized/ local spread	42.184804928	1	0.9295661066			1
HR+/HER2-	Localized/ local spread	42.381930185	1	0.9295661066			1
HR+/HER2-	Localized/ local spread	42.414784394	1	0.9295661066			1
HR+/HER2-	Localized/ local spread	42.480492813	1	0.9295661066			1
HR+/HER2-	Localized/ local spread	43.104722793	1	0.9295661066			1
HR+/HER2-	Localized/ local spread	43.137577002	1	0.9295661066			1

HR+/HER2-	Localized/ local spread	43.367556468	1	0.9295661066			1
HR+/HER2-	Localized/ local spread	43.400410678	1	0.9295661066			1
HR+/HER2-	Localized/ local spread	43.400410678	1	0.9295661066			1
HR+/HER2-	Localized/ local spread	43.597535934	1	0.9295661066			1
HR+/HER2-	Localized/ local spread	43.630390144	1	0.9295661066			1
HR+/HER2-	Localized/ local spread	44.714579055	1	0.9295661066			1
HR+/HER2-	Localized/ local spread	44.911704312	1	0.9295661066			1
HR+/HER2-	Localized/ local spread	44.911704312	1	0.9295661066			1
HR+/HER2-	Localized/ local spread	45.108829569	1	0.9295661066			1
HR+/HER2-	Localized/ local spread	45.667351129	1	0.9295661066			1
HR+/HER2-	Localized/ local spread	45.831622177	1	0.9295661066			1
HR+/HER2-	Localized/ local spread	46.061601643	1	0.9295661066			1
HR+/HER2-	Localized/ local spread	46.127310062	1	0.9295661066			1
HR+/HER2-	Localized/ local spread	46.258726899	1	0.9295661066			1
HR+/HER2-	Localized/ local spread	46.357289528	1	0.9295661066			1
HR+/HER2-	Localized/ local spread	46.357289528	1	0.9295661066			1
HR+/HER2-	Localized/ local spread	46.915811088	0	0.9260181444	0.8952756518	0.9479974682	1
HR+/HER2-	Localized/ local spread	46.981519507	1	0.9260181444			1
HR+/HER2-	Localized/ local spread	47.441478439	1	0.9260181444			1
HR+/HER2-	Localized/ local spread	47.507186858	1	0.9260181444			1
HR+/HER2-	Localized/ local spread	47.704312115	1	0.9260181444			1
HR+/HER2-	Localized/ local spread	47.737166324	1	0.9260181444			1
HR+/HER2-	Localized/ local spread	48	1	0.9260181444			1
HR+/HER2-	Localized/ local spread	48.131416838	1	0.9260181444			1
HR+/HER2-	Localized/ local spread	48.131416838	1	0.9260181444			1
HR+/HER2-	Localized/ local spread	48.459958932	1	0.9260181444			1
HR+/HER2-	Localized/ local spread	48.689938398	1	0.9260181444			1
HR+/HER2-	Localized/ local spread	48.788501027	1	0.9260181444			1
HR+/HER2-	Localized/ local spread	49.478439425	1	0.9260181444			1
HR+/HER2-	Localized/ local spread	50.234086242	1	0.9260181444			1
HR+/HER2-	Localized/ local spread	50.234086242	1	0.9260181444			1
HR+/HER2-	Localized/ local spread	50.39835729	1	0.9260181444			1
HR+/HER2-	Localized/ local spread	50.759753593	1	0.9260181444			1
HR+/HER2-	Localized/ local spread	51.383983573	1	0.9260181444			1
HR+/HER2-	Localized/ local spread	52.041067762	1	0.9260181444			1
HR+/HER2-	Localized/ local spread	52.205338809	0	0.9222073701	0.8904353888	0.9450496281	1
HR+/HER2-	Localized/ local spread	52.238193018	1	0.9222073701			1
HR+/HER2-	Localized/ local spread	52.566735113	0	0.9183807835	0.8856233903	0.9420626903	1
HR+/HER2-	Localized/ local spread	52.960985626	1	0.9183807835			1
HR+/HER2-	Localized/ local spread	52.993839836	1	0.9183807835			1
HR+/HER2-	Localized/ local spread	52.993839836	1	0.9183807835			1
HR+/HER2-	Localized/ local spread	53.388090349	1	0.9183807835			1
HR+/HER2-	Localized/ local spread	53.519507187	1	0.9183807835			1
HR+/HER2-	Localized/ local spread	53.618069815	1	0.9183807835			1
HR+/HER2-	Localized/ local spread	53.979466119	1	0.9183807835			1
HR+/HER2-	Localized/ local spread	54.439425051	1	0.9183807835			1
HR+/HER2-	Localized/ local spread	54.53798768	1	0.9183807835			1
HR+/HER2-	Localized/ local spread	54.636550308	1	0.9183807835			1
HR+/HER2-	Localized/ local spread	54.669404517	1	0.9183807835			1
HR+/HER2-	Localized/ local spread	55.063655031	1	0.9183807835			1

HR+/HER2-	Localized/ local spread	55.359342916	1	0.9183807835			1
HR+/HER2-	Localized/ local spread	55.589322382	1	0.9183807835			1
HR+/HER2-	Localized/ local spread	55.72073922	1	0.9183807835			1
HR+/HER2-	Localized/ local spread	55.950718686	1	0.9183807835			1
HR+/HER2-	Localized/ local spread	56.27926078	1	0.9183807835			1
HR+/HER2-	Localized/ local spread	56.607802875	1	0.9183807835			1
HR+/HER2-	Localized/ local spread	56.87063655	1	0.9183807835			1
HR+/HER2-	Localized/ local spread	56.87063655	1	0.9183807835			1
HR+/HER2-	Localized/ local spread	57.330595483	1	0.9183807835			1
HR+/HER2-	Localized/ local spread	57.823408624	1	0.9183807835			1
HR+/HER2-	Localized/ local spread	57.856262834	1	0.9183807835			1
HR+/HER2-	Localized/ local spread	58.0862423	1	0.9183807835			1
HR+/HER2-	Localized/ local spread	58.119096509	1	0.9183807835			1
HR+/HER2-	Localized/ local spread	58.151950719	0	0.914109245	0.880136851	0.9387870936	1
HR+/HER2-	Localized/ local spread	58.480492813	1	0.914109245			1
HR+/HER2-	Localized/ local spread	58.480492813	1	0.914109245			1
HR+/HER2-	Localized/ local spread	58.513347023	1	0.914109245			1
HR+/HER2-	Localized/ local spread	58.743326489	1	0.914109245			1
HR+/HER2-	Localized/ local spread	58.841889117	1	0.914109245			1
HR+/HER2-	Localized/ local spread	59.630390144	1	0.914109245			1
HR+/HER2-	Localized/ local spread	59.696098563	1	0.914109245			1
HR+/HER2-	Localized/ local spread	60.090349076	1	0.914109245			1
HR+/HER2-	Localized/ local spread	60.287474333	1	0.914109245			1
HR+/HER2-	Localized/ local spread	60.353182752	1	0.914109245			1
HR+/HER2-	Localized/ local spread	60.616016427	1	0.914109245			1
HR+/HER2-	Localized/ local spread	60.780287474	1	0.914109245			1
HR+/HER2-	Localized/ local spread	61.437371663	1	0.914109245			1
HR+/HER2-	Localized/ local spread	61.535934292	1	0.914109245			1
HR+/HER2-	Localized/ local spread	62.160164271	1	0.914109245			1
HR+/HER2-	Localized/ local spread	62.422997947	1	0.914109245			1
HR+/HER2-	Localized/ local spread	62.587268994	1	0.914109245			1
HR+/HER2-	Localized/ local spread	62.587268994	1	0.914109245			1
HR+/HER2-	Localized/ local spread	62.718685832	1	0.914109245			1
HR+/HER2-	Localized/ local spread	63.047227926	1	0.914109245			1
HR+/HER2-	Localized/ local spread	63.310061602	1	0.914109245			1
HR+/HER2-	Localized/ local spread	64.328542094	1	0.914109245			1
HR+/HER2-	Localized/ local spread	64.525667351	1	0.914109245			1
HR+/HER2-	Localized/ local spread	64.985626283	1	0.914109245			1
HR+/HER2-	Localized/ local spread	65.248459959	1	0.914109245			1
HR+/HER2-	Localized/ local spread	65.379876797	1	0.914109245			1
HR+/HER2-	Localized/ local spread	66.135523614	1	0.914109245			1
HR+/HER2-	Localized/ local spread	66.135523614	1	0.914109245			1
HR+/HER2-	Localized/ local spread	66.266940452	1	0.914109245			1
HR+/HER2-	Localized/ local spread	66.825462012	1	0.914109245			1
HR+/HER2-	Localized/ local spread	67.285420945	1	0.914109245			1
HR+/HER2-	Localized/ local spread	67.449691992	1	0.914109245			1
HR+/HER2-	Localized/ local spread	67.449691992	1	0.914109245			1
HR+/HER2-	Localized/ local spread	68.106776181	1	0.914109245			1
HR+/HER2-	Localized/ local spread	68.13963039	1	0.914109245			1
HR+/HER2-	Localized/ local spread	68.13963039	1	0.914109245			1

HR+/HER2-	Localized/ local spread	68.566735113	1	0.914109245			1
HR+/HER2-	Localized/ local spread	68.665297741	1	0.914109245			1
HR+/HER2-	Localized/ local spread	68.698151951	1	0.914109245			1
HR+/HER2-	Localized/ local spread	69.815195072	1	0.914109245			1
HR+/HER2-	Localized/ local spread	70.242299795	1	0.914109245			1
HR+/HER2-	Localized/ local spread	70.472279261	1	0.914109245			1
HR+/HER2-	Localized/ local spread	71.655030801	1	0.914109245			1
HR+/HER2-	Localized/ local spread	72.049281314	1	0.914109245			1
HR+/HER2-	Localized/ local spread	72.344969199	1	0.914109245			1
HR+/HER2-	Localized/ local spread	72.509240246	1	0.914109245			1
HR+/HER2-	Localized/ local spread	73.002053388	1	0.914109245			1
HR+/HER2-	Localized/ local spread	74.874743326	1	0.914109245			1
HR+/HER2-	Localized/ local spread	75.498973306	1	0.914109245			1
HR+/HER2-	Localized/ local spread	75.728952772	1	0.914109245			1
HR+/HER2-	Localized/ local spread	75.893223819	1	0.914109245			1
HR+/HER2-	Localized/ local spread	75.893223819	1	0.914109245			1
HR+/HER2-	Localized/ local spread	75.893223819	1	0.914109245			1
HR+/HER2-	Localized/ local spread	77.141683778	1	0.914109245			1
HR+/HER2-	Localized/ local spread	77.503080082	0	0.9083960622	0.8722034963	0.9347214099	1
HR+/HER2-	Localized/ local spread	77.63449692	1	0.9083960622			1
HR+/HER2-	Localized/ local spread	77.63449692	1	0.9083960622			1
HR+/HER2-	Localized/ local spread	77.995893224	1	0.9083960622			1
HR+/HER2-	Localized/ local spread	78.81724846	1	0.9083960622			1
HR+/HER2-	Localized/ local spread	79.507186858	1	0.9083960622			1
HR+/HER2-	Localized/ local spread	79.638603696	0	0.9024973865	0.8641226373	0.9304692152	1
HR+/HER2-	Localized/ local spread	80.164271047	1	0.9024973865			1
HR+/HER2-	Localized/ local spread	80.197125257	1	0.9024973865			1
HR+/HER2-	Localized/ local spread	81.018480493	1	0.9024973865			1
HR+/HER2-	Localized/ local spread	81.314168378	1	0.9024973865			1
HR+/HER2-	Localized/ local spread	81.80698152	1	0.9024973865			1
HR+/HER2-	Localized/ local spread	83.154004107	1	0.9024973865			1
HR+/HER2-	Localized/ local spread	83.646817248	1	0.9024973865			1
HR+/HER2-	Localized/ local spread	84.041067762	1	0.9024973865			1
HR+/HER2-	Localized/ local spread	84.238193018	1	0.9024973865			1
HR+/HER2-	Localized/ local spread	84.336755647	1	0.9024973865			1
HR+/HER2-	Localized/ local spread	84.501026694	1	0.9024973865			1
HR+/HER2-	Localized/ local spread	84.566735113	1	0.9024973865			1
HR+/HER2-	Localized/ local spread	84.895277207	1	0.9024973865			1
HR+/HER2-	Localized/ local spread	84.895277207	1	0.9024973865			1
HR+/HER2-	Localized/ local spread	85.125256674	1	0.9024973865			1
HR+/HER2-	Localized/ local spread	85.388090349	1	0.9024973865			1
HR+/HER2-	Localized/ local spread	86.340862423	1	0.9024973865			1
HR+/HER2-	Localized/ local spread	86.636550308	1	0.9024973865			1
HR+/HER2-	Localized/ local spread	86.735112936	1	0.9024973865			1
HR+/HER2-	Localized/ local spread	87.030800821	1	0.9024973865			1
HR+/HER2-	Localized/ local spread	87.425051335	1	0.9024973865			1
HR+/HER2-	Localized/ local spread	88.673511294	1	0.9024973865			1
HR+/HER2-	Localized/ local spread	88.837782341	1	0.9024973865			1
HR+/HER2-	Localized/ local spread	89.034907598	1	0.9024973865			1
HR+/HER2-	Localized/ local spread	89.199178645	0	0.8955012827	0.8541986325	0.9256117112	1

HR+/HER2-	Localized/ local spread	89.560574949	1	0.8955012827			1
HR+/HER2-	Localized/ local spread	89.790554415	1	0.8955012827			1
HR+/HER2-	Localized/ local spread	90.316221766	1	0.8955012827			1
HR+/HER2-	Localized/ local spread	90.414784394	0	0.8883372724	0.844247614	0.9205317726	1
HR+/HER2-	Localized/ local spread	90.64476386	1	0.8883372724			1
HR+/HER2-	Localized/ local spread	90.67761807	1	0.8883372724			1
HR+/HER2-	Localized/ local spread	91.301848049	1	0.8883372724			1
HR+/HER2-	Localized/ local spread	91.498973306	1	0.8883372724			1
HR+/HER2-	Localized/ local spread	92.123203285	1	0.8883372724			1
HR+/HER2-	Localized/ local spread	92.156057495	1	0.8883372724			1
HR+/HER2-	Localized/ local spread	92.287474333	1	0.8883372724			1
HR+/HER2-	Localized/ local spread	92.287474333	1	0.8883372724			1
HR+/HER2-	Localized/ local spread	92.714579055	1	0.8883372724			1
HR+/HER2-	Localized/ local spread	93.503080082	0	0.8806126005	0.8335243674	0.9150580184	1
HR+/HER2-	Localized/ local spread	93.63449692	1	0.8806126005			1
HR+/HER2-	Localized/ local spread	93.963039014	1	0.8806126005			1
HR+/HER2-	Localized/ local spread	94.422997947	1	0.8806126005			1
HR+/HER2-	Localized/ local spread	94.554414784	1	0.8806126005			1
HR+/HER2-	Localized/ local spread	94.784394251	1	0.8806126005			1
HR+/HER2-	Localized/ local spread	94.882956879	1	0.8806126005			1
HR+/HER2-	Localized/ local spread	94.915811088	1	0.8806126005			1
HR+/HER2-	Localized/ local spread	94.981519507	1	0.8806126005			1
HR+/HER2-	Localized/ local spread	95.277207392	1	0.8806126005			1
HR+/HER2-	Localized/ local spread	95.507186858	1	0.8806126005			1
HR+/HER2-	Localized/ local spread	95.704312115	1	0.8806126005			1
HR+/HER2-	Localized/ local spread	95.802874743	1	0.8806126005			1
HR+/HER2-	Localized/ local spread	96.854209446	1	0.8806126005			1
HR+/HER2-	Localized/ local spread	97.577002053	1	0.8806126005			1
HR+/HER2-	Localized/ local spread	98.759753593	1	0.8806126005			1
HR+/HER2-	Localized/ local spread	98.825462012	0	0.8717175237	0.8209039056	0.9089107544	1
HR+/HER2-	Localized/ local spread	99.252566735	1	0.8717175237			1
HR+/HER2-	Localized/ local spread	99.613963039	0	0.8627307451	0.8085095324	0.9025203498	1
HR+/HER2-	Localized/ local spread	100.10677618	1	0.8627307451			1
HR+/HER2-	Localized/ local spread	100.33675565	1	0.8627307451			1
HR+/HER2-	Localized/ local spread	100.89527721	1	0.8627307451			1
HR+/HER2-	Localized/ local spread	100.89527721	1	0.8627307451			1
HR+/HER2-	Localized/ local spread	101.25667351	1	0.8627307451			1
HR+/HER2-	Localized/ local spread	101.28952772	1	0.8627307451			1
HR+/HER2-	Localized/ local spread	101.32238193	0	0.853144848	0.7953621171	0.8956763802	1
HR+/HER2-	Localized/ local spread	101.35523614	0	0.8435589508	0.7825720912	0.8886499698	1
HR+/HER2-	Localized/ local spread	102.89938398	1	0.8435589508			1
HR+/HER2-	Localized/ local spread	103.12936345	1	0.8435589508			1
HR+/HER2-	Localized/ local spread	103.26078029	0	0.8337501258	0.76969382	0.8813555605	1
HR+/HER2-	Localized/ local spread	103.2936345	1	0.8337501258			1
HR+/HER2-	Localized/ local spread	103.88501027	1	0.8337501258			1
HR+/HER2-	Localized/ local spread	104.04928131	1	0.8337501258			1
HR+/HER2-	Localized/ local spread	104.24640657	1	0.8337501258			1
HR+/HER2-	Localized/ local spread	104.3449692	1	0.8337501258			1
HR+/HER2-	Localized/ local spread	104.73921971	1	0.8337501258			1
HR+/HER2-	Localized/ local spread	105.00205339	1	0.8337501258			1

HR+/HER2-	Localized/ local spread	105.0349076	1	0.8337501258			1
HR+/HER2-	Localized/ local spread	105.13347023	1	0.8337501258			1
HR+/HER2-	Localized/ local spread	105.19917864	1	0.8337501258			1
HR+/HER2-	Localized/ local spread	105.62628337	0	0.8226334574	0.7548096849	0.8732615003	1
HR+/HER2-	Localized/ local spread	106.18480493	1	0.8226334574			1
HR+/HER2-	Localized/ local spread	106.41478439	1	0.8226334574			1
HR+/HER2-	Localized/ local spread	107.00616016	1	0.8226334574			1
HR+/HER2-	Localized/ local spread	107.20328542	1	0.8226334574			1
HR+/HER2-	Localized/ local spread	107.99178645	1	0.8226334574			1
HR+/HER2-	Localized/ local spread	108.45174538	1	0.8226334574			1
HR+/HER2-	Localized/ local spread	109.10882957	1	0.8226334574			1
HR+/HER2-	Localized/ local spread	110.55441478	1	0.8226334574			1
HR+/HER2-	Localized/ local spread	111.14579055	1	0.8226334574			1
HR+/HER2-	Localized/ local spread	111.17864476	1	0.8226334574			1
HR+/HER2-	Localized/ local spread	111.40862423	1	0.8226334574			1
HR+/HER2-	Localized/ local spread	111.44147844	1	0.8226334574			1
HR+/HER2-	Localized/ local spread	111.67145791	1	0.8226334574			1
HR+/HER2-	Localized/ local spread	111.80287474	1	0.8226334574			1
HR+/HER2-	Localized/ local spread	111.86858316	1	0.8226334574			1
HR+/HER2-	Localized/ local spread	111.90143737	1	0.8226334574			1
HR+/HER2-	Localized/ local spread	111.93429158	1	0.8226334574			1
HR+/HER2-	Localized/ local spread	113.08418891	1	0.8226334574			1
HR+/HER2-	Localized/ local spread	113.51129363	1	0.8226334574			1
HR+/HER2-	Localized/ local spread	113.54414784	1	0.8226334574			1
HR+/HER2-	Localized/ local spread	113.67556468	1	0.8226334574			1
HR+/HER2-	Localized/ local spread	114.03696099	0	0.8071120714	0.7320101838	0.8631192787	1
HR+/HER2-	Localized/ local spread	114.46406571	1				1
HR+/HER2-	Localized/ local spread	114.92402464	1				1
HR+/HER2-	Localized/ local spread	115.05544148	1				1
HR+/HER2-	Localized/ local spread	115.35112936	1				1
HR+/HER2-	Localized/ local spread	115.4825462	1				1
HR+/HER2-	Localized/ local spread	116.40246407	1				1
HR+/HER2-	Localized/ local spread	116.43531828	1				1
HR+/HER2-	Localized/ local spread	116.50102669	1				1
HR+/HER2-	Localized/ local spread	116.66529774	1				1
HR+/HER2-	Localized/ local spread	116.66529774	1				1
HR+/HER2-	Localized/ local spread	117.38809035	1				1
HR+/HER2-	Localized/ local spread	117.88090349	1				1
HR+/HER2-	Localized/ local spread	117.88090349	1				1
HR+/HER2-	Localized/ local spread	117.9137577	1				1
HR+/HER2-	Localized/ local spread	118.07802875	1				1
HR+/HER2-	Localized/ local spread	118.14373717	1				1
HR+/HER2-	Localized/ local spread	118.14373717	1				1
HR+/HER2-	Localized/ local spread	118.50513347	1				1
HR+/HER2-	Localized/ local spread	118.9650924	1				1
HR+/HER2-	Localized/ local spread	119.26078029	1				1
HR+/HER2-	Localized/ local spread	119.42505133	1				1
HR+/HER2-	Localized/ local spread	119.95071869	1				1
HR+/HER2-	Localized/ local spread	120.31211499	1				1
HR+/HER2-	Localized/ local spread	120.44353183	1				1

HR+/HER2-	Localized/ local spread	121.10061602	1				1
HR+/HER2-	Localized/ local spread	121.13347023	1				1
HR+/HER2-	Localized/ local spread	121.13347023	1				1
HR+/HER2-	Localized/ local spread	122.02053388	1				1
HR+/HER2-	Localized/ local spread	122.18480493	1				1
HR+/HER2-	Localized/ local spread	122.64476386	1				1
HR+/HER2-	Localized/ local spread	122.71047228	1				1
HR+/HER2-	Localized/ local spread	122.71047228	1				1
HR+/HER2-	Localized/ local spread	122.84188912	1				1
HR+/HER2-	Localized/ local spread	123.76180698	1				1
HR+/HER2-	Localized/ local spread	124.25462012	1				1
HR+/HER2-	Localized/ local spread	124.48459959	1				1
HR+/HER2-	Localized/ local spread	125.37166324	1				1
HR+/HER2-	Localized/ local spread	125.73305955	1				1
HR+/HER2-	Localized/ local spread	125.73305955	1				1
HR+/HER2-	Localized/ local spread	125.73305955	1				1
HR+/HER2-	Localized/ local spread	126.55441478	1				1
HR+/HER2-	Localized/ local spread	126.58726899	1				1
HR+/HER2-	Localized/ local spread	127.11293634	1				1
HR+/HER2-	Localized/ local spread	127.34291581	1				1
HR+/HER2-	Localized/ local spread	127.70431211	1				1
HR+/HER2-	Localized/ local spread	127.90143737	1				1
HR+/HER2-	Localized/ local spread	127.93429158	1				1
HR+/HER2-	Localized/ local spread	128.13141684	1				1
HR+/HER2-	Localized/ local spread	128.13141684	1				1
HR+/HER2-	Localized/ local spread	128.42710472	1				1
HR+/HER2-	Localized/ local spread	128.85420945	1				1
HR+/HER2-	Localized/ local spread	129.08418891	1				1
HR+/HER2-	Metastatic	0		1	1	1	2
HR+/HER2-	Metastatic	0.2628336756	0	0.9821428571	0.8799254875	0.9974651359	2
HR+/HER2-	Metastatic	0.7227926078	0	0.9642857143	0.8646548381	0.9909464645	2
HR+/HER2-	Metastatic	0.887063655	0	0.9464285714	0.8430398434	0.9824012795	2
HR+/HER2-	Metastatic	2.0041067762	0	0.9285714286	0.8207816339	0.9725752698	2
HR+/HER2-	Metastatic	2.5297741273	1	0.9285714286			2
HR+/HER2-	Metastatic	4.1396303901	1	0.9285714286			2
HR+/HER2-	Metastatic	6.932238193	0	0.91	0.7971235988	0.9615325977	2
HR+/HER2-	Metastatic	8.5749486653	1	0.91			2
HR+/HER2-	Metastatic	10.184804928	1	0.91			2
HR+/HER2-	Metastatic	10.349075975	1	0.91			2
HR+/HER2-	Metastatic	10.743326489	1	0.91			2
HR+/HER2-	Metastatic	12.024640657	1	0.91			2
HR+/HER2-	Metastatic	13.995893224	0	0.8893181818	0.7696071706	0.9488148437	2
HR+/HER2-	Metastatic	14.718685832	1	0.8893181818			2
HR+/HER2-	Metastatic	14.981519507	0	0.8681439394	0.7423483698	0.9350961167	2
HR+/HER2-	Metastatic	15.868583162	0	0.846969697	0.7161485493	0.9206958122	2
HR+/HER2-	Metastatic	16.229979466	1	0.846969697			2
HR+/HER2-	Metastatic	17.577002053	1	0.846969697			2
HR+/HER2-	Metastatic	18.004106776	1	0.846969697			2
HR+/HER2-	Metastatic	18.694045175	1	0.846969697			2
HR+/HER2-	Metastatic	18.858316222	0	0.8234427609	0.6863985352	0.9045783794	2

HR+/HER2-	Metastatic	19.022587269	1	0.8234427609			2
HR+/HER2-	Metastatic	19.121149897	0	0.7992238562	0.6565848876	0.8874628685	2
HR+/HER2-	Metastatic	19.351129363	1	0.7992238562			2
HR+/HER2-	Metastatic	19.54825462	0	0.7742481107	0.6265357265	0.8693416936	2
HR+/HER2-	Metastatic	20.336755647	0	0.7492723652	0.5975104528	0.8506178109	2
HR+/HER2-	Metastatic	20.632443532	0	0.7242966197	0.5693331795	0.831351149	2
HR+/HER2-	Metastatic	20.862422998	0	0.6993208742	0.5418824399	0.8115875769	2
HR+/HER2-	Metastatic	21.190965092	1	0.6993208742			2
HR+/HER2-	Metastatic	21.420944559	0	0.6734201011	0.5136792413	0.7908280534	2
HR+/HER2-	Metastatic	22.340862423	1	0.6734201011			2
HR+/HER2-	Metastatic	22.899383984	0	0.646483297	0.4846320959	0.7689867818	2
HR+/HER2-	Metastatic	23.523613963	1	0.646483297			2
HR+/HER2-	Metastatic	24.640657084	0	0.6183753276	0.4546300257	0.7459556773	2
HR+/HER2-	Metastatic	25.297741273	0	0.5902673582	0.4254650644	0.7223731636	2
HR+/HER2-	Metastatic	25.823408624	0	0.5621593887	0.3970624124	0.6982634469	2
HR+/HER2-	Metastatic	25.856262834	1	0.5621593887			2
HR+/HER2-	Metastatic	26.447638604	0	0.5325720525	0.3673659317	0.6727422387	2
HR+/HER2-	Metastatic	28.714579055	0	0.5029847162	0.3385150621	0.6466366794	2
HR+/HER2-	Metastatic	29.60164271	1	0.5029847162			2
HR+/HER2-	Metastatic	30.850102669	0	0.4715481715	0.3080600949	0.6188238387	2
HR+/HER2-	Metastatic	32.098562628	0	0.4401116267	0.2786097568	0.5903195917	2
HR+/HER2-	Metastatic	32.394250513	0	0.4086750819	0.2501239458	0.561125451	2
HR+/HER2-	Metastatic	36.041067762	1	0.4086750819			2
HR+/HER2-	Metastatic	38.373716632	0	0.3746188251	0.2194372591	0.5296125407	2
HR+/HER2-	Metastatic	41.823408624	0	0.3405625683	0.1900805507	0.4971686362	2
HR+/HER2-	Metastatic	42.546201232	0	0.3065063114	0.1620483062	0.4637633365	2
HR+/HER2-	Metastatic	42.611909651	0	0.2724500546	0.1353684581	0.4293453732	2
HR+/HER2-	Metastatic	43.170431211	0	0.2383937978	0.1101065084	0.3938378348	2
HR+/HER2-	Metastatic	44.090349076	1	0.2383937978			2
HR+/HER2-	Metastatic	47.277207392	0	0.1986614982	0.080942424	0.3538243027	2
HR+/HER2-	Metastatic	53.979466119	0	0.1589291985	0.0549925445	0.3115076419	2
HR+/HER2-	Metastatic	54.439425051	1	0.1589291985			2
HR+/HER2-	Metastatic	56.837782341	0	0.105952799	0.0232585244	0.2619081563	2
HR+/HER2-	Metastatic	69.519507187	0	0.0529763995	0.0043687182	0.2042116712	2
HR+/HER2-	Metastatic	114.13552361	0	0			2
HR+/HER2-	Regional	0		1	1	1	3
HR+/HER2-	Regional	0.887063655	0	0.990990991	0.9377744118	0.9987260293	3
HR+/HER2-	Regional	1.0841889117	1	0.990990991			3
HR+/HER2-	Regional	1.7741273101	0	0.9818993305	0.9295617817	0.9954422976	3
HR+/HER2-	Regional	7.2936344969	1	0.9818993305			3
HR+/HER2-	Regional	8.6078028747	1	0.9818993305			3
HR+/HER2-	Regional	9.9219712526	1	0.9818993305			3
HR+/HER2-	Regional	10.05338809	1	0.9818993305			3
HR+/HER2-	Regional	10.743326489	1	0.9818993305			3
HR+/HER2-	Regional	10.907597536	0	0.9723663273	0.9167341374	0.9910081211	3
HR+/HER2-	Regional	11.071868583	1	0.9723663273			3
HR+/HER2-	Regional	11.104722793	1	0.9723663273			3
HR+/HER2-	Regional	11.498973306	1	0.9723663273			3
HR+/HER2-	Regional	12.254620123	1	0.9723663273			3
HR+/HER2-	Regional	12.583162218	1	0.9723663273			3

HR+/HER2-	Regional	12.813141684	1	0.9723663273			3
HR+/HER2-	Regional	13.273100616	1	0.9723663273			3
HR+/HER2-	Regional	13.700205339	1	0.9723663273			3
HR+/HER2-	Regional	13.733059548	1	0.9723663273			3
HR+/HER2-	Regional	13.733059548	1	0.9723663273			3
HR+/HER2-	Regional	14.061601643	1	0.9723663273			3
HR+/HER2-	Regional	14.291581109	1	0.9723663273			3
HR+/HER2-	Regional	14.488706366	1	0.9723663273			3
HR+/HER2-	Regional	15.967145791	1	0.9723663273			3
HR+/HER2-	Regional	16.492813142	1	0.9723663273			3
HR+/HER2-	Regional	16.492813142	1	0.9723663273			3
HR+/HER2-	Regional	16.887063655	1	0.9723663273			3
HR+/HER2-	Regional	17.084188912	1	0.9723663273			3
HR+/HER2-	Regional	18.036960986	1	0.9723663273			3
HR+/HER2-	Regional	18.069815195	1	0.9723663273			3
HR+/HER2-	Regional	18.825462012	0	0.9605082014	0.8974246195	0.9851109012	3
HR+/HER2-	Regional	19.154004107	0	0.9486500754	0.8800638164	0.9784840449	3
HR+/HER2-	Regional	19.186858316	0	0.9367919495	0.8637007086	0.9713238272	3
HR+/HER2-	Regional	19.252566735	1	0.9367919495			3
HR+/HER2-	Regional	19.383983573	1	0.9367919495			3
HR+/HER2-	Regional	20.501026694	0	0.9246258203	0.8473197707	0.9636109994	3
HR+/HER2-	Regional	21.256673511	1	0.9246258203			3
HR+/HER2-	Regional	21.256673511	1	0.9246258203			3
HR+/HER2-	Regional	21.880903491	1	0.9246258203			3
HR+/HER2-	Regional	21.9137577	1	0.9246258203			3
HR+/HER2-	Regional	22.439425051	1	0.9246258203			3
HR+/HER2-	Regional	22.439425051	1	0.9246258203			3
HR+/HER2-	Regional	22.603696099	1	0.9246258203			3
HR+/HER2-	Regional	23.162217659	0	0.9112254461	0.8289384231	0.9549785983	3
HR+/HER2-	Regional	23.917864476	0	0.8978250719	0.8112926243	0.94596672	3
HR+/HER2-	Regional	24.246406571	1	0.8978250719			3
HR+/HER2-	Regional	27.301848049	0	0.8842216617	0.7938074526	0.936534975	3
HR+/HER2-	Regional	28.484599589	0	0.8706182515	0.7767868399	0.9268170044	3
HR+/HER2-	Regional	32.098562628	0	0.8570148413	0.760142203	0.916848332	3
HR+/HER2-	Regional	34.759753593	1	0.8570148413			3
HR+/HER2-	Regional	34.891170431	0	0.8431920213	0.7434404582	0.9065352103	3
HR+/HER2-	Regional	36.501026694	0	0.8293692013	0.7270339625	0.8960123512	3
HR+/HER2-	Regional	36.665297741	1	0.8293692013			3
HR+/HER2-	Regional	38.932238193	1	0.8293692013			3
HR+/HER2-	Regional	39.917864476	1	0.8293692013			3
HR+/HER2-	Regional	40.837782341	1	0.8293692013			3
HR+/HER2-	Regional	40.87063655	1	0.8293692013			3
HR+/HER2-	Regional	41.790554415	1	0.8293692013			3
HR+/HER2-	Regional	42.020533881	1	0.8293692013			3
HR+/HER2-	Regional	42.151950719	1	0.8293692013			3
HR+/HER2-	Regional	42.381930185	1	0.8293692013			3
HR+/HER2-	Regional	43.104722793	1	0.8293692013			3
HR+/HER2-	Regional	43.400410678	1	0.8293692013			3
HR+/HER2-	Regional	43.400410678	1	0.8293692013			3
HR+/HER2-	Regional	43.531827515	1	0.8293692013			3

HR+/HER2-	Regional	44.911704312	1	0.8293692013			3
HR+/HER2-	Regional	45.963039014	1	0.8293692013			3
HR+/HER2-	Regional	49.18275154	0	0.8109387746	0.7029032543	0.8828736459	3
HR+/HER2-	Regional	50.332648871	0	0.7925083479	0.6796499688	0.869322863	3
HR+/HER2-	Regional	53.453798768	1	0.7925083479			3
HR+/HER2-	Regional	53.683778234	0	0.7736391015	0.6563572551	0.8551827322	3
HR+/HER2-	Regional	53.979466119	0	0.7547698551	0.6337196239	0.8406954835	3
HR+/HER2-	Regional	55.917864476	1	0.7547698551			3
HR+/HER2-	Regional	59.203285421	1	0.7547698551			3
HR+/HER2-	Regional	59.630390144	1	0.7547698551			3
HR+/HER2-	Regional	60.353182752	1	0.7547698551			3
HR+/HER2-	Regional	61.700205339	1	0.7547698551			3
HR+/HER2-	Regional	63.967145791	1	0.7547698551			3
HR+/HER2-	Regional	64.197125257	0	0.7325707417	0.606092455	0.8241476736	3
HR+/HER2-	Regional	64.295687885	1	0.7325707417			3
HR+/HER2-	Regional	67.876796715	0	0.7096779061	0.5782683001	0.8067602824	3
HR+/HER2-	Regional	69.683778234	0	0.6867850704	0.5513604658	0.788893097	3
HR+/HER2-	Regional	70.275154004	1	0.6867850704			3
HR+/HER2-	Regional	72.542094456	1	0.6867850704			3
HR+/HER2-	Regional	73.199178645	0	0.6622570322	0.522648898	0.7697124424	3
HR+/HER2-	Regional	76.484599589	1	0.6622570322			3
HR+/HER2-	Regional	77.305954825	0	0.6367856078	0.493377666	0.7495223888	3
HR+/HER2-	Regional	78.324435318	1	0.6367856078			3
HR+/HER2-	Regional	78.488706366	1	0.6367856078			3
HR+/HER2-	Regional	78.981519507	0	0.6090992771	0.4616175584	0.7276291668	3
HR+/HER2-	Regional	80.262833676	0	0.5814129463	0.4310133554	0.7050925083	3
HR+/HER2-	Regional	80.328542094	1	0.5814129463			3
HR+/HER2-	Regional	82.135523614	0	0.552342299	0.3993947729	0.6811991091	3
HR+/HER2-	Regional	87.063655031	1	0.552342299			3
HR+/HER2-	Regional	89.034907598	1	0.552342299			3
HR+/HER2-	Regional	93.963039014	1	0.552342299			3
HR+/HER2-	Regional	94.127310062	0	0.5178209053	0.3608183005	0.653838545	3
HR+/HER2-	Regional	95.605749487	1	0.5178209053			3
HR+/HER2-	Regional	98.464065708	0	0.4808336978	0.3206203683	0.6241552964	3
HR+/HER2-	Regional	98.628336756	0	0.4438464902	0.2827409466	0.5931494854	3
HR+/HER2-	Regional	104.7063655	1	0.4438464902			3
HR+/HER2-	Regional	107.20328542	1	0.4438464902			3
HR+/HER2-	Regional	109.76591376	0	0.3994618412	0.2367615763	0.5573970149	3
HR+/HER2-	Regional	112.59137577	1				3
HR+/HER2-	Regional	113.93839836	1				3
HR+/HER2-	Regional	114.20123203	1				3
HR+/HER2-	Regional	120.41067762	1				3
HR+/HER2-	Regional	123.33470226	1				3
HR+/HER2-	Regional	125.83162218	1				3
HR+/HER2-	Regional	126.88295688	1				3
HR+/HER2-	Regional	127.54004107	1				3
HR+/HER2-	Regional	129.0513347	1				3
TNBC	Localized/ local spread	0		1	1	1	1
TNBC	Localized/ local spread	1.3798767967	0	0.9945355191	0.961847645	0.999228443	1
TNBC	Localized/ local spread	1.5770020534	0	0.9890710383	0.9570117311	0.9972554389	1

TNBC	Localized/ local spread	1.7412731006	1	0.9890710383			1
TNBC	Localized/ local spread	2.726899384	0	0.9835761991	0.9499485992	0.9946733803	1
TNBC	Localized/ local spread	3.318275154	0	0.97808136	0.9426577165	0.9917168662	1
TNBC	Localized/ local spread	5.0924024641	0	0.9725865209	0.9353967672	0.9884976459	1
TNBC	Localized/ local spread	5.5852156057	1	0.9725865209			1
TNBC	Localized/ local spread	6.6694045175	1	0.9725865209			1
TNBC	Localized/ local spread	8.0492813142	1	0.9725865209			1
TNBC	Localized/ local spread	8.1149897331	1	0.9725865209			1
TNBC	Localized/ local spread	8.2792607803	1	0.9725865209			1
TNBC	Localized/ local spread	10.283367556	1	0.9725865209			1
TNBC	Localized/ local spread	10.414784394	1	0.9725865209			1
TNBC	Localized/ local spread	10.743326489	1	0.9725865209			1
TNBC	Localized/ local spread	10.973305955	1	0.9725865209			1
TNBC	Localized/ local spread	11.696098563	0	0.9667973155	0.927571985	0.9849495197	1
TNBC	Localized/ local spread	11.761806982	1	0.9667973155			1
TNBC	Localized/ local spread	11.893223819	0	0.9609732353	0.919849981	0.9812100713	1
TNBC	Localized/ local spread	13.108829569	0	0.955149155	0.9122778163	0.9773259173	1
TNBC	Localized/ local spread	14.19301848	1	0.955149155			1
TNBC	Localized/ local spread	14.258726899	0	0.9492893443	0.9047613156	0.9733023014	1
TNBC	Localized/ local spread	14.422997947	0	0.9434295335	0.8973550558	0.9691736088	1
TNBC	Localized/ local spread	14.521560575	1	0.9434295335			1
TNBC	Localized/ local spread	14.521560575	1	0.9434295335			1
TNBC	Localized/ local spread	14.718685832	1	0.9434295335			1
TNBC	Localized/ local spread	14.850102669	1	0.9434295335			1
TNBC	Localized/ local spread	15.014373717	1	0.9434295335			1
TNBC	Localized/ local spread	15.310061602	0	0.9373819083	0.8897069093	0.9648514484	1
TNBC	Localized/ local spread	15.310061602	1	0.9373819083			1
TNBC	Localized/ local spread	15.40862423	1	0.9373819083			1
TNBC	Localized/ local spread	15.474332649	1	0.9373819083			1
TNBC	Localized/ local spread	16.657084189	1	0.9373819083			1
TNBC	Localized/ local spread	16.887063655	1	0.9373819083			1
TNBC	Localized/ local spread	17.347022587	1	0.9373819083			1
TNBC	Localized/ local spread	17.379876797	1	0.9373819083			1
TNBC	Localized/ local spread	17.379876797	1	0.9373819083			1
TNBC	Localized/ local spread	17.412731006	0	0.9310051606	0.88157722	0.960262255	1
TNBC	Localized/ local spread	17.412731006	1	0.9310051606			1
TNBC	Localized/ local spread	17.445585216	0	0.9245844354	0.8734953214	0.9555600103	1
TNBC	Localized/ local spread	17.741273101	1	0.9245844354			1
TNBC	Localized/ local spread	18.464065708	1	0.9245844354			1
TNBC	Localized/ local spread	18.661190965	1	0.9245844354			1
TNBC	Localized/ local spread	19.219712526	1	0.9245844354			1
TNBC	Localized/ local spread	19.58110883	1	0.9245844354			1
TNBC	Localized/ local spread	19.811088296	1	0.9245844354			1
TNBC	Localized/ local spread	20.008213552	1	0.9245844354			1
TNBC	Localized/ local spread	20.271047228	1	0.9245844354			1
TNBC	Localized/ local spread	20.369609856	1	0.9245844354			1
TNBC	Localized/ local spread	20.599589322	1	0.9245844354			1
TNBC	Localized/ local spread	20.599589322	1	0.9245844354			1
TNBC	Localized/ local spread	20.829568789	1	0.9245844354			1
TNBC	Localized/ local spread	20.862422998	1	0.9245844354			1

TNBC	Localized/ local spread	21.256673511	0	0.9175265389	0.8644093647	0.9504251296	1
TNBC	Localized/ local spread	21.420944559	1	0.9175265389			1
TNBC	Localized/ local spread	22.242299795	0	0.9104139301	0.8553879197	0.9451655878	1
TNBC	Localized/ local spread	22.735112936	0	0.9033013213	0.8465070719	0.9398195567	1
TNBC	Localized/ local spread	22.965092402	1	0.9033013213			1
TNBC	Localized/ local spread	23.359342916	1	0.9033013213			1
TNBC	Localized/ local spread	23.457905544	1	0.9033013213			1
TNBC	Localized/ local spread	23.655030801	0	0.8960166332	0.8374600164	0.9342959289	1
TNBC	Localized/ local spread	24.049281314	0	0.8887319451	0.8285320645	0.9286959381	1
TNBC	Localized/ local spread	27.26899384	1	0.8887319451			1
TNBC	Localized/ local spread	27.400410678	0	0.881387053	0.8196117291	0.9229900239	1
TNBC	Localized/ local spread	27.433264887	0	0.8740421609	0.8107882273	0.9172181771	1
TNBC	Localized/ local spread	29.765913758	0	0.8666972688	0.8020514491	0.9113853056	1
TNBC	Localized/ local spread	29.963039014	1	0.8666972688			1
TNBC	Localized/ local spread	32.427104723	1	0.8666972688			1
TNBC	Localized/ local spread	32.985626283	0	0.8592257406	0.7931983964	0.9054161684	1
TNBC	Localized/ local spread	34.036960986	1	0.8592257406			1
TNBC	Localized/ local spread	34.299794661	0	0.8516886727	0.7843234727	0.8993496406	1
TNBC	Localized/ local spread	34.529774127	1	0.8516886727			1
TNBC	Localized/ local spread	34.726899384	0	0.8440843096	0.7754215645	0.8931862753	1
TNBC	Localized/ local spread	34.792607803	0	0.8364799464	0.7665910713	0.8869709391	1
TNBC	Localized/ local spread	34.858316222	1	0.8364799464			1
TNBC	Localized/ local spread	36.205338809	1	0.8364799464			1
TNBC	Localized/ local spread	36.271047228	1	0.8364799464			1
TNBC	Localized/ local spread	36.829568789	1	0.8364799464			1
TNBC	Localized/ local spread	37.650924025	1	0.8364799464			1
TNBC	Localized/ local spread	37.782340862	1	0.8364799464			1
TNBC	Localized/ local spread	37.815195072	1	0.8364799464			1
TNBC	Localized/ local spread	38.078028747	0	0.8283587819	0.7570559526	0.8803734889	1
TNBC	Localized/ local spread	39.392197125	1	0.8283587819			1
TNBC	Localized/ local spread	39.490759754	1	0.8283587819			1
TNBC	Localized/ local spread	39.72073922	1	0.8283587819			1
TNBC	Localized/ local spread	39.852156057	1	0.8283587819			1
TNBC	Localized/ local spread	40.147843943	1	0.8283587819			1
TNBC	Localized/ local spread	41.100616016	0	0.8198190006	0.7469743353	0.8734534658	1
TNBC	Localized/ local spread	42.151950719	1	0.8198190006			1
TNBC	Localized/ local spread	42.447638604	1	0.8198190006			1
TNBC	Localized/ local spread	42.874743326	1	0.8198190006			1
TNBC	Localized/ local spread	43.761806982	1	0.8198190006			1
TNBC	Localized/ local spread	44.320328542	1	0.8198190006			1
TNBC	Localized/ local spread	45.04312115	0	0.8108100006	0.7362785851	0.8661763079	1
TNBC	Localized/ local spread	45.174537988	0	0.8018010006	0.7256962633	0.8588282973	1
TNBC	Localized/ local spread	45.667351129	1	0.8018010006			1
TNBC	Localized/ local spread	45.831622177	1	0.8018010006			1
TNBC	Localized/ local spread	45.930184805	1	0.8018010006			1
TNBC	Localized/ local spread	45.930184805	1	0.8018010006			1
TNBC	Localized/ local spread	45.930184805	1	0.8018010006			1
TNBC	Localized/ local spread	46.390143737	1	0.8018010006			1
TNBC	Localized/ local spread	46.718685832	1	0.8018010006			1
TNBC	Localized/ local spread	47.441478439	1	0.8018010006			1

TNBC	Localized/ local spread	47.967145791	1	0.8018010006			1
TNBC	Localized/ local spread	48.361396304	1	0.8018010006			1
TNBC	Localized/ local spread	48.459958932	1	0.8018010006			1
TNBC	Localized/ local spread	48.624229979	1	0.8018010006			1
TNBC	Localized/ local spread	49.117043121	1	0.8018010006			1
TNBC	Localized/ local spread	49.609856263	1	0.8018010006			1
TNBC	Localized/ local spread	49.80698152	1	0.8018010006			1
TNBC	Localized/ local spread	50.858316222	1	0.8018010006			1
TNBC	Localized/ local spread	51.613963039	1	0.8018010006			1
TNBC	Localized/ local spread	52.599589322	1	0.8018010006			1
TNBC	Localized/ local spread	52.928131417	1	0.8018010006			1
TNBC	Localized/ local spread	56.049281314	1	0.8018010006			1
TNBC	Localized/ local spread	56.049281314	1	0.8018010006			1
TNBC	Localized/ local spread	56.27926078	1	0.8018010006			1
TNBC	Localized/ local spread	57.199178645	1	0.8018010006			1
TNBC	Localized/ local spread	58.940451745	1	0.8018010006			1
TNBC	Localized/ local spread	60.090349076	1	0.8018010006			1
TNBC	Localized/ local spread	60.878850103	1	0.8018010006			1
TNBC	Localized/ local spread	61.273100616	1	0.8018010006			1
TNBC	Localized/ local spread	62.620123203	0	0.7888687264	0.7088751217	0.8492004316	1
TNBC	Localized/ local spread	63.047227926	1	0.7888687264			1
TNBC	Localized/ local spread	64.689938398	1	0.7888687264			1
TNBC	Localized/ local spread	67.646817248	0	0.77549807	0.6917267945	0.8391286105	1
TNBC	Localized/ local spread	67.646817248	1	0.77549807			1
TNBC	Localized/ local spread	67.778234086	1	0.77549807			1
TNBC	Localized/ local spread	68.698151951	1	0.77549807			1
TNBC	Localized/ local spread	69.289527721	1	0.77549807			1
TNBC	Localized/ local spread	72.246406571	1	0.77549807			1
TNBC	Localized/ local spread	72.27926078	1	0.77549807			1
TNBC	Localized/ local spread	72.509240246	1	0.77549807			1
TNBC	Localized/ local spread	73.691991786	1	0.77549807			1
TNBC	Localized/ local spread	75.696098563	1	0.77549807			1
TNBC	Localized/ local spread	77.535934292	1	0.77549807			1
TNBC	Localized/ local spread	77.765913758	1	0.77549807			1
TNBC	Localized/ local spread	79.40862423	1	0.77549807			1
TNBC	Localized/ local spread	79.507186858	1	0.77549807			1
TNBC	Localized/ local spread	80.558521561	1	0.77549807			1
TNBC	Localized/ local spread	81.117043121	1	0.77549807			1
TNBC	Localized/ local spread	81.708418891	1	0.77549807			1
TNBC	Localized/ local spread	81.77412731	1	0.77549807			1
TNBC	Localized/ local spread	85.158110883	0	0.756583483	0.6649648039	0.8263820351	1
TNBC	Localized/ local spread	85.158110883	1				1
TNBC	Localized/ local spread	86.800821355	1				1
TNBC	Localized/ local spread	87.260780287	1				1
TNBC	Localized/ local spread	88.607802875	1				1
TNBC	Localized/ local spread	90.447638604	1				1
TNBC	Localized/ local spread	93.04312115	1				1
TNBC	Localized/ local spread	95.014373717	1				1
TNBC	Localized/ local spread	95.540041068	1				1
TNBC	Localized/ local spread	98.825462012	1				1

TNBC	Localized/ local spread	100.56673511	1				1
TNBC	Localized/ local spread	100.76386037	1				1
TNBC	Localized/ local spread	103.19507187	1				1
TNBC	Localized/ local spread	104.7063655	1				1
TNBC	Localized/ local spread	106.34907598	1				1
TNBC	Localized/ local spread	106.80903491	1				1
TNBC	Localized/ local spread	107.79466119	1				1
TNBC	Localized/ local spread	109.07597536	1				1
TNBC	Localized/ local spread	109.14168378	1				1
TNBC	Localized/ local spread	111.44147844	1				1
TNBC	Localized/ local spread	112.62422998	1				1
TNBC	Localized/ local spread	113.11704312	1				1
TNBC	Localized/ local spread	113.21560575	1				1
TNBC	Localized/ local spread	114.13552361	1				1
TNBC	Localized/ local spread	114.46406571	1				1
TNBC	Localized/ local spread	114.82546201	1				1
TNBC	Localized/ local spread	116.04106776	1				1
TNBC	Localized/ local spread	116.5338809	1				1
TNBC	Localized/ local spread	117.09240246	1				1
TNBC	Localized/ local spread	118.275154	1				1
TNBC	Localized/ local spread	119.52361396	1				1
TNBC	Localized/ local spread	119.85215606	1				1
TNBC	Localized/ local spread	119.9835729	1				1
TNBC	Localized/ local spread	121.0349076	1				1
TNBC	Localized/ local spread	121.13347023	1				1
TNBC	Localized/ local spread	121.82340862	1				1
TNBC	Localized/ local spread	122.41478439	1				1
TNBC	Localized/ local spread	122.84188912	1				1
TNBC	Localized/ local spread	123.89322382	1				1
TNBC	Localized/ local spread	128.82135524	1				1
TNBC	Localized/ local spread	129.18275154	1				1
TNBC	Metastatic	0		1	1	1	2
TNBC	Metastatic	1.0841889117	0	0.9375	0.6323454417	0.9909531417	2
TNBC	Metastatic	1.5441478439	0	0.875	0.5859814368	0.967188846	2
TNBC	Metastatic	2.8583162218	0	0.8125	0.5246043108	0.935352258	2
TNBC	Metastatic	4.0410677618	0	0.75	0.4634300427	0.8979799097	2
TNBC	Metastatic	5.3552361396	1	0.75			2
TNBC	Metastatic	5.9137577002	0	0.6818181818	0.3952553101	0.853827914	2
TNBC	Metastatic	6.5051334702	0	0.6136363636	0.3325129427	0.8052554742	2
TNBC	Metastatic	6.5708418891	0	0.5454545455	0.2743775683	0.752698211	2
TNBC	Metastatic	6.8336755647	0	0.4772727273	0.2205108475	0.6963593211	2
TNBC	Metastatic	11.761806982	0	0.4090909091	0.1708551286	0.6362627572	2
TNBC	Metastatic	11.926078029	0	0.3409090909	0.1255815349	0.5722594887	2
TNBC	Metastatic	18.891170431	0	0.2727272727	0.0851163862	0.5039925791	2
TNBC	Metastatic	19.121149897	0	0.2045454545	0.0502526011	0.430805525	2
TNBC	Metastatic	19.778234086	0	0.1363636364	0.0224255797	0.3515682014	2
TNBC	Metastatic	27.26899384	0	0.0681818182	0.0043894731	0.2648497713	2
TNBC	Metastatic	28.747433265	0	0			2
TNBC	Regional	0		1	1	1	3
TNBC	Regional	0.7885010267	1	1			3

TNBC	Regional	3.4825462012	1	1			3
TNBC	Regional	6.1437371663	0	0.9873417722	0.9135323853	0.9982071691	3
TNBC	Regional	6.5708418891	1	0.9873417722			3
TNBC	Regional	8.772073922	0	0.9745191517	0.9019312171	0.993566295	3
TNBC	Regional	10.05338809	0	0.9616965313	0.8859314712	0.9874844313	3
TNBC	Regional	10.349075975	1	0.9616965313			3
TNBC	Regional	11.466119097	0	0.9487006322	0.8690516058	0.9804345497	3
TNBC	Regional	11.531827515	0	0.9357047332	0.8523842394	0.9727280989	3
TNBC	Regional	11.761806982	0	0.9227088341	0.8359989775	0.9645205393	3
TNBC	Regional	12.024640657	1	0.9227088341			3
TNBC	Regional	12.747433265	0	0.9095272793	0.8195263777	0.9558222141	3
TNBC	Regional	14.291581109	1	0.9095272793			3
TNBC	Regional	14.521560575	0	0.8961518782	0.8029859798	0.9466830922	3
TNBC	Regional	15.145790554	1	0.8961518782			3
TNBC	Regional	15.178644764	0	0.8825738194	0.7863745004	0.9371365595	3
TNBC	Regional	15.244353183	1	0.8825738194			3
TNBC	Regional	15.310061602	1	0.8825738194			3
TNBC	Regional	15.474332649	0	0.8685647112	0.7692912011	0.9270889971	3
TNBC	Regional	15.671457906	0	0.8545556029	0.7525203357	0.9167831626	3
TNBC	Regional	15.704312115	1	0.8545556029			3
TNBC	Regional	16.459958932	0	0.8403130095	0.7356291295	0.9061161446	3
TNBC	Regional	16.689938398	1	0.8403130095			3
TNBC	Regional	16.722792608	0	0.8258248542	0.7185995149	0.8950920743	3
TNBC	Regional	17.084188912	0	0.8113366989	0.7018279069	0.8838618776	3
TNBC	Regional	17.511293634	1	0.8113366989			3
TNBC	Regional	17.77412731	0	0.7965851225	0.6848736816	0.8722856902	3
TNBC	Regional	17.839835729	1	0.7965851225			3
TNBC	Regional	18.069815195	1	0.7965851225			3
TNBC	Regional	18.299794661	1	0.7965851225			3
TNBC	Regional	18.496919918	1	0.7965851225			3
TNBC	Regional	18.792607803	1	0.7965851225			3
TNBC	Regional	19.055441478	0	0.7803282833	0.665780937	0.8596402778	3
TNBC	Regional	20.369609856	1	0.7803282833			3
TNBC	Regional	20.435318275	1	0.7803282833			3
TNBC	Regional	20.468172485	0	0.7633646249	0.6459152598	0.8463603214	3
TNBC	Regional	20.533880903	1	0.7633646249			3
TNBC	Regional	20.632443532	1	0.7633646249			3
TNBC	Regional	20.632443532	1	0.7633646249			3
TNBC	Regional	22.866529774	1	0.7633646249			3
TNBC	Regional	23.917864476	0	0.7447459756	0.6237917693	0.8319031899	3
TNBC	Regional	26.874743326	0	0.7261273262	0.6021790491	0.8171518871	3
TNBC	Regional	27.433264887	1	0.7261273262			3
TNBC	Regional	28.747433265	0	0.7070187123	0.5802573193	0.8018449505	3
TNBC	Regional	30.521560575	0	0.6879100985	0.558782144	0.7862634043	3
TNBC	Regional	31.441478439	0	0.6688014846	0.537707054	0.7704245224	3
TNBC	Regional	37.092402464	0	0.6496928708	0.5169954074	0.7543423639	3
TNBC	Regional	38.899383984	1	0.6496928708			3
TNBC	Regional	45.404517454	1	0.6496928708			3
TNBC	Regional	47.211498973	1	0.6496928708			3
TNBC	Regional	49.971252567	0	0.6287350362	0.4939570458	0.7368939618	3

TNBC	Regional	52.369609856	1				3
TNBC	Regional	57.067761807	1				3
TNBC	Regional	58.513347023	1				3
TNBC	Regional	58.973305955	1				3
TNBC	Regional	65.215605749	1				3
TNBC	Regional	66.98973306	1				3
TNBC	Regional	69.552361396	1				3
TNBC	Regional	71.425051335	1				3
TNBC	Regional	71.425051335	1				3
TNBC	Regional	72.574948665	1				3
TNBC	Regional	72.969199179	1				3
TNBC	Regional	75.301848049	1				3
TNBC	Regional	80.197125257	1				3
TNBC	Regional	87.030800821	1				3
TNBC	Regional	88.706365503	1				3
TNBC	Regional	89.034907598	1				3
TNBC	Regional	90.743326489	1				3
TNBC	Regional	93.174537988	1				3
TNBC	Regional	93.207392197	1				3
TNBC	Regional	102.89938398	1				3
TNBC	Regional	105.95482546	1				3
TNBC	Regional	106.34907598	1				3
TNBC	Regional	107.30184805	1				3
TNBC	Regional	110.91581109	1				3
TNBC	Regional	115.25256674	1				3
TNBC	Regional	115.84394251	1				3
TNBC	Regional	118.11088296	1				3
TNBC	Regional	126.55441478	1				3
TNBC	Regional	126.81724846	1				3
TNBC	Regional	127.70431211	1				3
HR-/HER2+	Localized/ local spread	0		1	1	1	1
HR-/HER2+	Localized/ local spread	5.0595482546	0	0.9850746269	0.8987440984	0.9978839931	1
HR-/HER2+	Localized/ local spread	10.184804928	1	0.9850746269			1
HR-/HER2+	Localized/ local spread	12.254620123	1	0.9850746269			1
HR-/HER2+	Localized/ local spread	12.944558522	1	0.9850746269			1
HR-/HER2+	Localized/ local spread	12.977412731	1	0.9850746269			1
HR-/HER2+	Localized/ local spread	13.01026694	1	0.9850746269			1
HR-/HER2+	Localized/ local spread	14.882956879	1	0.9850746269			1
HR-/HER2+	Localized/ local spread	15.934291581	1	0.9850746269			1
HR-/HER2+	Localized/ local spread	17.839835729	1	0.9850746269			1
HR-/HER2+	Localized/ local spread	18.036960986	1	0.9850746269			1
HR-/HER2+	Localized/ local spread	18.924024641	1	0.9850746269			1
HR-/HER2+	Localized/ local spread	19.942505133	0	0.9674840085	0.8755339665	0.9918128628	1
HR-/HER2+	Localized/ local spread	20.796714579	1	0.9674840085			1
HR-/HER2+	Localized/ local spread	20.862422998	1	0.9674840085			1
HR-/HER2+	Localized/ local spread	22.340862423	1	0.9674840085			1
HR-/HER2+	Localized/ local spread	23.490759754	1	0.9674840085			1
HR-/HER2+	Localized/ local spread	24.640657084	1	0.9674840085			1
HR-/HER2+	Localized/ local spread	27.23613963	0	0.9481343284	0.8466522638	0.9831045761	1
HR-/HER2+	Localized/ local spread	27.893223819	1	0.9481343284			1

HR-/HER2+	Localized/ local spread	29.700205339	0	0.9283815299	0.8191233718	0.9727014422	1
HR-/HER2+	Localized/ local spread	31.901437372	0	0.9086287313	0.7931250813	0.9611615552	1
HR-/HER2+	Localized/ local spread	35.219712526	1	0.9086287313			1
HR-/HER2+	Localized/ local spread	35.909650924	1	0.9086287313			1
HR-/HER2+	Localized/ local spread	36.895277207	1	0.9086287313			1
HR-/HER2+	Localized/ local spread	40.082135524	1	0.9086287313			1
HR-/HER2+	Localized/ local spread	45.930184805	1	0.9086287313			1
HR-/HER2+	Localized/ local spread	45.963039014	0	0.886467055	0.7628934667	0.9477521783	1
HR-/HER2+	Localized/ local spread	46.160164271	1	0.886467055			1
HR-/HER2+	Localized/ local spread	46.488706366	1	0.886467055			1
HR-/HER2+	Localized/ local spread	46.751540041	1	0.886467055			1
HR-/HER2+	Localized/ local spread	46.850102669	1	0.886467055			1
HR-/HER2+	Localized/ local spread	46.948665298	1	0.886467055			1
HR-/HER2+	Localized/ local spread	47.507186858	1	0.886467055			1
HR-/HER2+	Localized/ local spread	48.558521561	1	0.886467055			1
HR-/HER2+	Localized/ local spread	50.299794661	1	0.886467055			1
HR-/HER2+	Localized/ local spread	50.464065708	0	0.8587649595	0.7221948858	0.931246025	1
HR-/HER2+	Localized/ local spread	55.293634497	0	0.831062864	0.6850265402	0.9134562212	1
HR-/HER2+	Localized/ local spread	56.180698152	1	0.831062864			1
HR-/HER2+	Localized/ local spread	58.67761807	1	0.831062864			1
HR-/HER2+	Localized/ local spread	60.41889117	1	0.831062864			1
HR-/HER2+	Localized/ local spread	61.273100616	1	0.831062864			1
HR-/HER2+	Localized/ local spread	61.667351129	0	0.7990989077	0.641641111	0.8928362583	1
HR-/HER2+	Localized/ local spread	63.540041068	1	0.7990989077			1
HR-/HER2+	Localized/ local spread	65.642710472	1	0.7990989077			1
HR-/HER2+	Localized/ local spread	65.839835729	1	0.7990989077			1
HR-/HER2+	Localized/ local spread	68.369609856	1	0.7990989077			1
HR-/HER2+	Localized/ local spread	69.519507187	1	0.7990989077			1
HR-/HER2+	Localized/ local spread	69.782340862	1	0.7990989077			1
HR-/HER2+	Localized/ local spread	70.702258727	1	0.7990989077			1
HR-/HER2+	Localized/ local spread	72.772073922	1	0.7990989077			1
HR-/HER2+	Localized/ local spread	76.386036961	0	0.7520930896	0.5698373987	0.865612121	1
HR-/HER2+	Localized/ local spread	76.944558522	1	0.7520930896			1
HR-/HER2+	Localized/ local spread	81.051334702	1	0.7520930896			1
HR-/HER2+	Localized/ local spread	83.186858316	1	0.7520930896			1
HR-/HER2+	Localized/ local spread	84.106776181	1	0.7520930896			1
HR-/HER2+	Localized/ local spread	92.845995893	1	0.7520930896			1
HR-/HER2+	Localized/ local spread	106.54620123	1	0.7520930896			1
HR-/HER2+	Localized/ local spread	106.61190965	1	0.7520930896			1
HR-/HER2+	Localized/ local spread	106.87474333	0	0.6685271908	0.4268299399	0.826580875	1
HR-/HER2+	Localized/ local spread	107.66324435	1				1
HR-/HER2+	Localized/ local spread	110.55441478	1				1
HR-/HER2+	Localized/ local spread	117.81519507	1				1
HR-/HER2+	Localized/ local spread	118.34086242	1				1
HR-/HER2+	Localized/ local spread	121.00205339	1				1
HR-/HER2+	Localized/ local spread	123.56468172	1				1
HR-/HER2+	Localized/ local spread	123.89322382	1				1
HR-/HER2+	Localized/ local spread	127.01437372	1				1
HR-/HER2+	Metastatic	0		1	1	1	2
HR-/HER2+	Metastatic	1.2484599589	0	0.9230769231	0.5663594661	0.9887940623	2

HR-/HER2+	Metastatic	5.8809034908	0	0.8461538462	0.5122035907	0.9591454605	2
HR-/HER2+	Metastatic	6.2094455852	0	0.7692307692	0.4421414979	0.9191153262	2
HR-/HER2+	Metastatic	6.4722792608	0	0.6923076923	0.3733634842	0.8717501165	2
HR-/HER2+	Metastatic	6.5051334702	1	0.6923076923			2
HR-/HER2+	Metastatic	10.349075975	0	0.5192307692	0.2245847741	0.7500480179	2
HR-/HER2+	Metastatic	17.117043121	1	0.5192307692			2
HR-/HER2+	Metastatic	17.347022587	1	0.5192307692			2
HR-/HER2+	Metastatic	30.981519507	0	0.3894230769	0.1152054393	0.6626096945	2
HR-/HER2+	Metastatic	43.23613963	0	0.2596153846	0.0454308817	0.5553007336	2
HR-/HER2+	Metastatic	60.813141684	0	0.1298076923	0.0075591912	0.4259910066	2
HR-/HER2+	Metastatic	78.554414784	0	0			2
HR-/HER2+	Regional	0		1	1	1	3
HR-/HER2+	Regional	2.8911704312	1	1			3
HR-/HER2+	Regional	10.283367556	1	1			3
HR-/HER2+	Regional	10.447638604	1	1			3
HR-/HER2+	Regional	14.784394251	1	1			3
HR-/HER2+	Regional	15.112936345	1	1			3
HR-/HER2+	Regional	15.310061602	1	1			3
HR-/HER2+	Regional	19.416837782	0	0.9545454545	0.7187028534	0.9934696024	3
HR-/HER2+	Regional	19.58110883	1	0.9545454545			3
HR-/HER2+	Regional	20.106776181	1	0.9545454545			3
HR-/HER2+	Regional	20.369609856	1	0.9545454545			3
HR-/HER2+	Regional	20.369609856	1	0.9545454545			3
HR-/HER2+	Regional	20.402464066	1	0.9545454545			3
HR-/HER2+	Regional	25.856262834	0	0.8948863636	0.6361801992	0.9730973887	3
HR-/HER2+	Regional	26.184804928	1	0.8948863636			3
HR-/HER2+	Regional	28.156057495	1	0.8948863636			3
HR-/HER2+	Regional	36.468172485	1	0.8948863636			3
HR-/HER2+	Regional	36.862422998	1	0.8948863636			3
HR-/HER2+	Regional	39.917864476	1	0.8948863636			3
HR-/HER2+	Regional	50.891170431	0	0.8053977273	0.4888431601	0.9366540538	3
HR-/HER2+	Regional	52.533880903	1	0.8053977273			3
HR-/HER2+	Regional	55.556468172	1	0.8053977273			3
HR-/HER2+	Regional	56.640657084	1	0.8053977273			3
HR-/HER2+	Regional	76.386036961	0	0.6711647727	0.2990846739	0.8765833023	3
HR-/HER2+	Regional	80.131416838	1				3
HR-/HER2+	Regional	83.285420945	1				3
HR-/HER2+	Regional	85.453798768	1				3
HR-/HER2+	Regional	109.73305955	1				3
HR-/HER2+	Regional	118.11088296	1				3
HR+/HER2+	Localized/ local spread	0		1	1	1	1
HR+/HER2+	Localized/ local spread	0	1	1			1
HR+/HER2+	Localized/ local spread	0	1	1			1
HR+/HER2+	Localized/ local spread	0.59137577	1	1			1
HR+/HER2+	Localized/ local spread	0.7885010267	1	1			1
HR+/HER2+	Localized/ local spread	1.2813141684	1	1			1
HR+/HER2+	Localized/ local spread	1.839835729	1	1			1
HR+/HER2+	Localized/ local spread	2.8583162218	1	1			1
HR+/HER2+	Localized/ local spread	3.9753593429	1	1			1
HR+/HER2+	Localized/ local spread	4.3367556468	1	1			1

HR+/HER2+	Localized/ local spread	10.184804928	1	1				1
HR+/HER2+	Localized/ local spread	10.710472279	1	1				1
HR+/HER2+	Localized/ local spread	10.776180698	1	1				1
HR+/HER2+	Localized/ local spread	11.531827515	1	1				1
HR+/HER2+	Localized/ local spread	11.564681725	1	1				1
HR+/HER2+	Localized/ local spread	11.86036961	1	1				1
HR+/HER2+	Localized/ local spread	12.320328542	1	1				1
HR+/HER2+	Localized/ local spread	12.517453799	1	1				1
HR+/HER2+	Localized/ local spread	14.784394251	1	1				1
HR+/HER2+	Localized/ local spread	16.164271047	1	1				1
HR+/HER2+	Localized/ local spread	16.854209446	1	1				1
HR+/HER2+	Localized/ local spread	16.952772074	1	1				1
HR+/HER2+	Localized/ local spread	17.347022587	1	1				1
HR+/HER2+	Localized/ local spread	17.741273101	0	0.9919354839	0.9441379583	0.998860055		1
HR+/HER2+	Localized/ local spread	17.80698152	1	0.9919354839				1
HR+/HER2+	Localized/ local spread	17.905544148	0	0.9838048652	0.9367970625	0.9959250079		1
HR+/HER2+	Localized/ local spread	17.905544148	1	0.9838048652				1
HR+/HER2+	Localized/ local spread	18.496919918	1	0.9838048652				1
HR+/HER2+	Localized/ local spread	18.661190965	1	0.9838048652				1
HR+/HER2+	Localized/ local spread	19.712525667	1	0.9838048652				1
HR+/HER2+	Localized/ local spread	19.843942505	1	0.9838048652				1
HR+/HER2+	Localized/ local spread	19.942505133	1	0.9838048652				1
HR+/HER2+	Localized/ local spread	20.566735113	0	0.9752500402	0.9251937018	0.9919546501		1
HR+/HER2+	Localized/ local spread	21.420944559	1	0.9752500402				1
HR+/HER2+	Localized/ local spread	23.425051335	1	0.9752500402				1
HR+/HER2+	Localized/ local spread	24.344969199	1	0.9752500402				1
HR+/HER2+	Localized/ local spread	24.574948665	1	0.9752500402				1
HR+/HER2+	Localized/ local spread	25.954825462	1	0.9752500402				1
HR+/HER2+	Localized/ local spread	26.414784394	1	0.9752500402				1
HR+/HER2+	Localized/ local spread	27.466119097	1	0.9752500402				1
HR+/HER2+	Localized/ local spread	28.123203285	1	0.9752500402				1
HR+/HER2+	Localized/ local spread	28.221765914	0	0.9660495682	0.9119164814	0.9871448189		1
HR+/HER2+	Localized/ local spread	32.952772074	0	0.9568490961	0.899246178	0.9818458676		1
HR+/HER2+	Localized/ local spread	33.412731006	1	0.9568490961				1
HR+/HER2+	Localized/ local spread	33.905544148	1	0.9568490961				1
HR+/HER2+	Localized/ local spread	34.332648871	1	0.9568490961				1
HR+/HER2+	Localized/ local spread	35.186858316	1	0.9568490961				1
HR+/HER2+	Localized/ local spread	35.515400411	1	0.9568490961				1
HR+/HER2+	Localized/ local spread	35.515400411	1	0.9568490961				1
HR+/HER2+	Localized/ local spread	35.942505133	0	0.9470853298	0.885710455	0.9759406201		1
HR+/HER2+	Localized/ local spread	36.1724846	1	0.9470853298				1
HR+/HER2+	Localized/ local spread	36.402464066	1	0.9470853298				1
HR+/HER2+	Localized/ local spread	36.402464066	1	0.9470853298				1
HR+/HER2+	Localized/ local spread	37.190965092	1	0.9470853298				1
HR+/HER2+	Localized/ local spread	37.782340862	1	0.9470853298				1
HR+/HER2+	Localized/ local spread	37.848049281	1	0.9470853298				1
HR+/HER2+	Localized/ local spread	37.880903491	1	0.9470853298				1
HR+/HER2+	Localized/ local spread	38.50513347	1	0.9470853298				1
HR+/HER2+	Localized/ local spread	38.899383984	1	0.9470853298				1
HR+/HER2+	Localized/ local spread	39.753593429	0	0.9363229965	0.8704846809	0.9692722395		1

HR+/HER2+	Localized/ local spread	39.852156057	1	0.9363229965			1
HR+/HER2+	Localized/ local spread	41.297741273	1	0.9363229965			1
HR+/HER2+	Localized/ local spread	41.527720739	1	0.9363229965			1
HR+/HER2+	Localized/ local spread	41.691991786	1	0.9363229965			1
HR+/HER2+	Localized/ local spread	41.790554415	1	0.9363229965			1
HR+/HER2+	Localized/ local spread	42.217659138	1	0.9363229965			1
HR+/HER2+	Localized/ local spread	42.940451745	1	0.9363229965			1
HR+/HER2+	Localized/ local spread	43.597535934	1	0.9363229965			1
HR+/HER2+	Localized/ local spread	44.090349076	1	0.9363229965			1
HR+/HER2+	Localized/ local spread	44.911704312	1	0.9363229965			1
HR+/HER2+	Localized/ local spread	45.174537988	1	0.9363229965			1
HR+/HER2+	Localized/ local spread	45.207392197	1	0.9363229965			1
HR+/HER2+	Localized/ local spread	45.568788501	1	0.9363229965			1
HR+/HER2+	Localized/ local spread	46.127310062	1	0.9363229965			1
HR+/HER2+	Localized/ local spread	46.291581109	1	0.9363229965			1
HR+/HER2+	Localized/ local spread	46.291581109	1	0.9363229965			1
HR+/HER2+	Localized/ local spread	46.718685832	1	0.9363229965			1
HR+/HER2+	Localized/ local spread	47.014373717	1	0.9363229965			1
HR+/HER2+	Localized/ local spread	47.014373717	1	0.9363229965			1
HR+/HER2+	Localized/ local spread	47.211498973	1	0.9363229965			1
HR+/HER2+	Localized/ local spread	47.474332649	1	0.9363229965			1
HR+/HER2+	Localized/ local spread	47.967145791	1	0.9363229965			1
HR+/HER2+	Localized/ local spread	48.164271047	1	0.9363229965			1
HR+/HER2+	Localized/ local spread	48.229979466	1	0.9363229965			1
HR+/HER2+	Localized/ local spread	50.266940452	1	0.9363229965			1
HR+/HER2+	Localized/ local spread	51.449691992	1	0.9363229965			1
HR+/HER2+	Localized/ local spread	52.271047228	1	0.9363229965			1
HR+/HER2+	Localized/ local spread	52.533880903	0	0.9207176132	0.8445603399	0.9604169441	1
HR+/HER2+	Localized/ local spread	54.110882957	1	0.9207176132			1
HR+/HER2+	Localized/ local spread	55.950718686	1	0.9207176132			1
HR+/HER2+	Localized/ local spread	57.100616016	1	0.9207176132			1
HR+/HER2+	Localized/ local spread	57.297741273	1	0.9207176132			1
HR+/HER2+	Localized/ local spread	57.429158111	1	0.9207176132			1
HR+/HER2+	Localized/ local spread	57.560574949	0	0.9036672871	0.8177147628	0.9502921099	1
HR+/HER2+	Localized/ local spread	58.119096509	1	0.9036672871			1
HR+/HER2+	Localized/ local spread	58.316221766	1	0.9036672871			1
HR+/HER2+	Localized/ local spread	58.447638604	1	0.9036672871			1
HR+/HER2+	Localized/ local spread	60.188911704	1	0.9036672871			1
HR+/HER2+	Localized/ local spread	60.813141684	1	0.9036672871			1
HR+/HER2+	Localized/ local spread	61.963039014	1	0.9036672871			1
HR+/HER2+	Localized/ local spread	62.915811088	1	0.9036672871			1
HR+/HER2+	Localized/ local spread	64.229979466	1	0.9036672871			1
HR+/HER2+	Localized/ local spread	65.708418891	1	0.9036672871			1
HR+/HER2+	Localized/ local spread	66.529774127	1	0.9036672871			1
HR+/HER2+	Localized/ local spread	66.759753593	1	0.9036672871			1
HR+/HER2+	Localized/ local spread	66.759753593	1	0.9036672871			1
HR+/HER2+	Localized/ local spread	67.318275154	1	0.9036672871			1
HR+/HER2+	Localized/ local spread	67.54825462	1	0.9036672871			1
HR+/HER2+	Localized/ local spread	67.646817248	1	0.9036672871			1
HR+/HER2+	Localized/ local spread	68.13963039	1	0.9036672871			1

HR+/HER2+	Localized/ local spread	70.275154004	1	0.9036672871			1
HR+/HER2+	Localized/ local spread	70.669404517	1	0.9036672871			1
HR+/HER2+	Localized/ local spread	72.016427105	1	0.9036672871			1
HR+/HER2+	Localized/ local spread	74.874743326	0	0.8770888374	0.7693230694	0.9365185713	1
HR+/HER2+	Localized/ local spread	78.81724846	1	0.8770888374			1
HR+/HER2+	Localized/ local spread	78.948665298	1	0.8770888374			1
HR+/HER2+	Localized/ local spread	79.014373717	1	0.8770888374			1
HR+/HER2+	Localized/ local spread	79.047227926	1	0.8770888374			1
HR+/HER2+	Localized/ local spread	81.018480493	1	0.8770888374			1
HR+/HER2+	Localized/ local spread	81.971252567	1	0.8770888374			1
HR+/HER2+	Localized/ local spread	82.464065708	1	0.8770888374			1
HR+/HER2+	Localized/ local spread	83.58110883	1	0.8770888374			1
HR+/HER2+	Localized/ local spread	83.811088296	1	0.8770888374			1
HR+/HER2+	Localized/ local spread	85.125256674	1	0.8770888374			1
HR+/HER2+	Localized/ local spread	90.184804928	0	0.8389545402	0.6979087623	0.9178402787	1
HR+/HER2+	Localized/ local spread	90.349075975	0	0.8008202429	0.6393954995	0.8955513066	1
HR+/HER2+	Localized/ local spread	91.23613963	1	0.8008202429			1
HR+/HER2+	Localized/ local spread	92.648870637	0	0.7607792307	0.5838369722	0.8703030611	1
HR+/HER2+	Localized/ local spread	93.207392197	1				1
HR+/HER2+	Localized/ local spread	93.273100616	1				1
HR+/HER2+	Localized/ local spread	94.160164271	1				1
HR+/HER2+	Localized/ local spread	95.802874743	1				1
HR+/HER2+	Localized/ local spread	96.032854209	1				1
HR+/HER2+	Localized/ local spread	96.295687885	1				1
HR+/HER2+	Localized/ local spread	96.854209446	1				1
HR+/HER2+	Localized/ local spread	100.82956879	1				1
HR+/HER2+	Localized/ local spread	101.12525667	1				1
HR+/HER2+	Localized/ local spread	108.94455852	1				1
HR+/HER2+	Localized/ local spread	110.19301848	1				1
HR+/HER2+	Localized/ local spread	110.9486653	1				1
HR+/HER2+	Localized/ local spread	112.95277207	1				1
HR+/HER2+	Localized/ local spread	113.54414784	1				1
HR+/HER2+	Localized/ local spread	114.66119097	1				1
HR+/HER2+	Localized/ local spread	123.40041068	1				1
HR+/HER2+	Localized/ local spread	126.12731006	1				1
HR+/HER2+	Localized/ local spread	127.34291581	1				1
HR+/HER2+	Localized/ local spread	129.14989733	1				1
HR+/HER2+	Metastatic	0		1	1	1	2
HR+/HER2+	Metastatic	0.3285420945	0	0.9523809524	0.7072067656	0.9931521372	2
HR+/HER2+	Metastatic	0.8213552361	0	0.9047619048	0.6700458822	0.975294149	2
HR+/HER2+	Metastatic	1.1498973306	0	0.8571428571	0.6197179553	0.9515517476	2
HR+/HER2+	Metastatic	5.9794661191	0	0.8095238095	0.5689050599	0.9238888286	2
HR+/HER2+	Metastatic	14.587268994	1	0.8095238095			2
HR+/HER2+	Metastatic	18.39835729	1	0.8095238095			2
HR+/HER2+	Metastatic	20.599589322	0	0.7555555556	0.5075423525	0.8906053846	2
HR+/HER2+	Metastatic	21.453798768	1	0.7555555556			2
HR+/HER2+	Metastatic	25.297741273	1	0.7555555556			2
HR+/HER2+	Metastatic	25.987679671	0	0.6925925926	0.4347794067	0.8504548658	2
HR+/HER2+	Metastatic	27.564681725	0	0.6296296296	0.3699067433	0.806377564	2
HR+/HER2+	Metastatic	31.474332649	0	0.5666666667	0.3107084037	0.7588198429	2

HR+/HER2+	Metastatic	34.694045175	0	0.5037037037	0.2561619547	0.7080096179	2
HR+/HER2+	Metastatic	36.435318275	0	0.4407407407	0.2057962833	0.6540258824	2
HR+/HER2+	Metastatic	42.64476386	1	0.4407407407			2
HR+/HER2+	Metastatic	49.248459959	1	0.4407407407			2
HR+/HER2+	Metastatic	56.016427105	1	0.4407407407			2
HR+/HER2+	Metastatic	68.238193018	1	0.4407407407			2
HR+/HER2+	Metastatic	79.704312115	0	0.2938271605	0.0677540832	0.5727818492	2
HR+/HER2+	Metastatic	111.27720739	0	0.1469135802	0.0097975459	0.4514825356	2
HR+/HER2+	Metastatic	127.27720739	1				2
HR+/HER2+	Regional	0		1	1	1	3
HR+/HER2+	Regional	3.318275154	0	0.976744186	0.8461554119	0.9966910485	3
HR+/HER2+	Regional	10.710472279	1	0.976744186			3
HR+/HER2+	Regional	10.940451745	1	0.976744186			3
HR+/HER2+	Regional	12.353182752	1	0.976744186			3
HR+/HER2+	Regional	12.583162218	1	0.976744186			3
HR+/HER2+	Regional	13.437371663	1	0.976744186			3
HR+/HER2+	Regional	14.488706366	1	0.976744186			3
HR+/HER2+	Regional	15.277207392	1	0.976744186			3
HR+/HER2+	Regional	16.229979466	1	0.976744186			3
HR+/HER2+	Regional	20.501026694	1	0.976744186			3
HR+/HER2+	Regional	20.632443532	1	0.976744186			3
HR+/HER2+	Regional	20.698151951	1	0.976744186			3
HR+/HER2+	Regional	21.388090349	1	0.976744186			3
HR+/HER2+	Regional	25.889117043	1	0.976744186			3
HR+/HER2+	Regional	27.86036961	1	0.976744186			3
HR+/HER2+	Regional	36.205338809	1	0.976744186			3
HR+/HER2+	Regional	37.519507187	1	0.976744186			3
HR+/HER2+	Regional	40.772073922	1	0.976744186			3
HR+/HER2+	Regional	41.691991786	1	0.976744186			3
HR+/HER2+	Regional	42.447638604	0	0.9360465116	0.7560951136	0.9844987651	3
HR+/HER2+	Regional	44.977412731	1	0.9360465116			3
HR+/HER2+	Regional	47.835728953	0	0.8934989429	0.6953482729	0.9657009466	3
HR+/HER2+	Regional	58.0862423	1	0.8934989429			3
HR+/HER2+	Regional	62.620123203	1	0.8934989429			3
HR+/HER2+	Regional	62.718685832	1	0.8934989429			3
HR+/HER2+	Regional	65.642710472	1	0.8934989429			3
HR+/HER2+	Regional	67.022587269	1	0.8934989429			3
HR+/HER2+	Regional	67.646817248	1	0.8934989429			3
HR+/HER2+	Regional	72.27926078	1	0.8934989429			3
HR+/HER2+	Regional	73.856262834	1	0.8934989429			3
HR+/HER2+	Regional	81.77412731	1	0.8934989429			3
HR+/HER2+	Regional	83.515400411	1	0.8934989429			3
HR+/HER2+	Regional	83.843942505	1	0.8934989429			3
HR+/HER2+	Regional	84.106776181	0	0.8041490486	0.5111465177	0.9316518238	3
HR+/HER2+	Regional	85.486652977	1	0.8041490486			3
HR+/HER2+	Regional	88.410677618	1	0.8041490486			3
HR+/HER2+	Regional	92.254620123	1	0.8041490486			3
HR+/HER2+	Regional	96.229979466	0	0.6701242072	0.306376168	0.873319578	3
HR+/HER2+	Regional	109.99589322	1				3
HR+/HER2+	Regional	114.66119097	1				3

HR+/HER2+	Regional	123.56468172	1				3
HR+/HER2+	Regional	124.09034908	1				3
HR+/HER2+	Regional	125.63449692	1				3
